# A 1,681-locus consensus genetic map of cultivated cucumber including 67 NB-LRR resistance gene homolog and ten gene loci

**DOI:** 10.1186/1471-2229-13-53

**Published:** 2013-03-25

**Authors:** Luming Yang, Dawei Li, Yuhong Li, Xingfang Gu, Sanwen Huang, Jordi Garcia-Mas, Yiqun Weng

**Affiliations:** 1 Horticulture Department, University of Wisconsin, Madison, WI 53706, USA; 2 Horticulture College, Northwest A&F University, Yangling, 712100, China; 3 Institute of Vegetables and Flowers, Chinese Academy of Agricultural Sciences, Beijing, 100018, China; 4 IRTA, Center for Research in Agricultural Genomics CSIC-IRTA-UAB-UB, Bellaterra, Barcelona, 08193, Spain; 5 USDA-ARS Vegetable Crops Research Unit, Horticulture Department, University of Wisconsin, Madison, WI, 53706, USA

**Keywords:** Cucumber, *Cucumis sativus*, NB-LRR, Resistance gene homolog, Genetic mapping, Comparative mapping, Map integration

## Abstract

**Background:**

Cucumber is an important vegetable crop that is susceptible to many pathogens, but no disease resistance (R) genes have been cloned. The availability of whole genome sequences provides an excellent opportunity for systematic identification and characterization of the nucleotide binding and leucine-rich repeat (NB-LRR) type R gene homolog (RGH) sequences in the genome. Cucumber has a very narrow genetic base making it difficult to construct high-density genetic maps. Development of a consensus map by synthesizing information from multiple segregating populations is a method of choice to increase marker density. As such, the objectives of the present study were to identify and characterize NB-LRR type RGHs, and to develop a high-density, integrated cucumber genetic-physical map anchored with RGH loci.

**Results:**

From the Gy14 draft genome, 70 NB-containing RGHs were identified and characterized. Most RGHs were in clusters with uneven distribution across seven chromosomes. *In silico* analysis indicated that all 70 RGHs had EST support for gene expression. Phylogenetic analysis classified 58 RGHs into two clades: CNL and TNL. Comparative analysis revealed high-degree sequence homology and synteny in chromosomal locations of these RGH members between the cucumber and melon genomes.

Fifty-four molecular markers were developed to delimit 67 of the 70 RGHs, which were integrated into a genetic map through linkage analysis. A 1,681-locus cucumber consensus map including 10 gene loci and spanning 730.0 cM in seven linkage groups was developed by integrating three component maps with a bin-mapping strategy. Physically, 308 scaffolds with 193.2 Mbp total DNA sequences were anchored onto this consensus map that covered 52.6% of the 367 Mbp cucumber genome.

**Conclusions:**

**C**ucumber contains relatively few NB-LRR RGHs that are clustered and unevenly distributed in the genome. All RGHs seem to be transcribed and shared significant sequence homology and synteny with the melon genome suggesting conservation of these RGHs in the *Cucumis* lineage. The 1,681-locus consensus genetic-physical map developed and the RGHs identified and characterized herein are valuable genomics resources that may have many applications such as quantitative trait loci identification, map-based gene cloning, association mapping, marker-assisted selection, as well as assembly of a more complete cucumber genome.

## Background

Over the last decade, many plant pathogen resistance (R) genes or quantitative trait loci (QTL) have been cloned. The largest class of known R genes encodes proteins with a central nucleotide binding (NB) domain and a C-terminal leucine-rich repeat (LRR) domain [1]. Based on the amino-terminal domain feature, the NB-LRR proteins can be divided into two classes: TNL (TIR-NB-LRR) and CNL (CC-NB-LRR) in which the R proteins possess, respectively, either the Toll/Interleukin-1 Receptor (TIR) domain or a coiled-coil (CC) domain [2]. The NB domain seems to have NTP-hydrolyzing activity for regulating signal transduction through conformational changes [2]. The LRR domain contains tandemly arrayed repeats that is involved in the specific recognition of pathogen effectors [3]. Both TIR and CC domains are assumed to be involved in protein-protein interactions and signal transduction [4,5].

Due to the availability of whole genome sequences, NB-encoding resistance gene homolog (RGH) sequences have been annotated and mapped in a number of plant species such as *Arabidopsis thaliana*[6], poplar (*Populus trichocarpa*) [7], potato (*Solanum tuberosum*) [8,9], rice (*Oryza sativa*) [10], sorghum (*Sorghum bicolor*) [11], grapevine (*Vitis vinifera*) [12], coffee tree (*Coffea arabica*) [13], *Medicago truncatula*[14], and papaya (*Carica papaya*) [15]. While NB-LRR genes are widely distributed among plant genomes, their numbers vary greatly in different species. For example, the papaya and grapevine genome contains 55 and 535 NB-LRR RGHs representing 0.2% and 1.8% of their total genes, respectively [12,15]. A lack of recent genome duplication was believed to be the reason of the overall low NB-LRR gene numbers in papaya [16]. NB-encoding genes are unevenly distributed in the plant genome and are mainly organized in multi-gene clusters. The clustered distribution of R-genes is assumed to provide a reservoir of genetic variations from which new pathogen specificity can evolve via gene duplication, unequal crossing-over, ectopic recombination or diversifying selection [17,18]. In addition, nucleotide polymorphism analyses demonstrated extremely high level of inter- and intra-specific variations of NB-LRR genes, which presumably evolve rapidly in response to changes in pathogen populations [12,19]. Nevertheless, conservation of synteny for NB–LRR disease resistance genes among phylogenetically related species was also observed [20,21]. However, the extent of genome-wide conservation and synteny of NB-LRR RGHs between different species is not well documented.

Cucumber, *Cucumis sativus* L. (2n = 2*x* =14) is an economically important vegetable crop and a system of choice for studying several important biological processes [22]. In recent years, application of next generation sequencing technologies enabled release of draft genomes of three cucumber lines (‘9930’, ‘Gy14’ and ‘B10’) [23-25] providing powerful tools for understanding the structure and organization of R genes in the cucumber genome. In the 9930 draft genome, 61 NB-containing RGHs were identified [23], but no details were given for these RGHs, and the RGH numbers seem to be underestimated as compared with an improved annotation of the 9930 genome (Version 2.0) [26]. Thus, one objective of the present study was to conduct genome wide identification and characterization of NB-LRR type RGHs in the Gy14 draft genome assembly (Version 1.0) [27]. Since the ratio of genetic to physical distances varies along the chromosomes (for example, [28]), the information of genetic map locations of RGHs, especially on a high-density reference genetic map, is very useful for map-based cloning of R genes or association mapping through the candidate gene approach. The association of RGHs with candidate disease resistance genes has been well established in a number of crops such as melon (*Cucumis melo*) [29,30], wheat (*Triticum aestivum*) [31], cucumber [32], sunflower (*Helianthus annuus*) [33], and potato [34]. The information of genetic and physical locations of RGHs also allows for quick map-based cloning of several R genes or QTL in rice [35-37], poplar [38] and common bean (*Phaseolus vulgaris*) [39].

Cultivated cucumber has a very narrow genetic base [28,40,41] making it difficult to develop high-density genetic maps. From whole genome sequences, tens of thousands of simple sequence repeat (SSR) markers have been developed [24,42]. Among all SSR-based cucumber genetic maps constructed thus far [27,28,42-46], the one by Ren *et al.*[42] with 995 SSR loci has the highest marker density. However, this map was developed with a limited number of recombinant inbred lines (RILs) from an inter-subspecific cross between Gy14 and the wild cucumber (*C. sativus* var. *hardwickii*) accession PI 183967 (CSH-RIL map hereinafter). Strong recombination suppression was found in this mapping population, and more than one quarter of mapped loci were clustered across five chromosomes (3, 4, 5, 6 and 7). As a result, the total genetic distance of this map is only 572.9 cM, which is shorter than the expected ~750 cM map length for the cucumber genome [27]. The most recent intra-varietal linkage map of cultivated cucumber was developed with an F_2_ population of Gy14 × 9930 (CSS-F2 map hereinafter) containing 735 marker loci with a total map length of 707.8 cM, which allowed for integration of the genetic and physical maps to develop a chromosome-level draft genome assembly of Gy14 (Version 1.0) [27]. While such maps are a significant improvement as compared with those AFLP- or RAPD-based maps developed early, marker density on this map is still far from being satisfactory for many molecular marker-based applications such as marker-assisted breeding, map-based gene cloning or assembly of a more complete cucumber genome.

For cultivated crops like cucumber with limited genetic diversity, development of a dense consensus map is a method of choice to increase marker density, which is usually achieved through map integration by synthesizing the information from multiple segregating populations of diverse genetic backgrounds. This allows for mapping a larger number of loci than in most single crosses to saturate the map, thus providing a genomic framework for QTL identification, map-based gene cloning, assessment of genetic diversity, association mapping, as well as marker-assisted selection in molecular breeding [47]. Consensus maps have been constructed in a number of crop species such as lettuce (*Lactuca sativa*) [48], grapevine [49], cowpea (*Vigna unguiculata*) [50], red clover (*Trifolium pratense*) [51], sorghum [52], soybean (*Glycine max*) [53,54], melon [47], and the oilseed rape, *Brassica napus*[55]. In cucumber, a consensus map with 1,369 mapped loci was also developed by integrating the CSH-RIL map (Gy14 × PI 183967 RIL) and the S94 × S06 RIL map [44]. A major drawback associated with this consensus map is that marker orders in the recombination suppressed regions were not well resolved, which greatly affect the accuracy of the order of loci and the quality of the resulting integrated cucumber map. Thus, the second objective of the present study was to develop a high-density consensus map for cultivated cucumber by integrating several individual maps and to anchor all NB-LRR type RGHs identified herein onto this integrated map.

We first scanned the Gy14 draft genome and bioinformatically identified and characterized 70 NB-containing RGH sequences. *In silico* expression in cucumber transcriptome and conservation in sequence homology and colinearity between cucumber and melon genomes were investigated. Through comparison between the Gy14 and 9930 draft genome sequences, we identified DNA polymorphisms in the regions harbouring the RGHs, and genetically mapped these RGH loci on the Gy14 × 9930 F_2_ linkage map (CSS-F2 map) [27]. By integrating three component maps, we developed a cucumber consensus map that contained 1,681 loci and anchored 67 RGH loci and 10 cucumber genes.

## Results

### Characterization of NB-containing R gene sequences in Gy14 cucumber genome

Seventy non-redundant NB-encoding RGHs were identified in the cucumber Gy14 draft genome. It seems all RGHs presented in the cucumber genome as single copy because BLAST alignment against the Gy14 draft genome assembly didn’t find any paralogs for each RGH sequence. The nucleotide and peptide sequences of all 70 RGHs are provided in Additional file 1. Based on the C- and N-terminal domain features, the 70 RGHs could be classified into six subgroups [6]: N (NB), CN (CC-NB), NL (NB-LRR), TN (TIR-NB), TNL (TIR-NB-LRR), and CNL (CC-NB-LRR) with 3, 1, 17, 5, 19, 25 members in each category, respectively (Table 1). The names, protein domain features, scaffold and Gy14 draft genome positions, and chromosome locations of all 70 RGHs are presented in Additional file 2: Table S1. The annotated NB-encoding RGHs in the Gy14 and 9930 (Version2.0) [26] were largely consistent. As shown in Additional file 2: Table S1, only two RGHs in the Gy14 genome, Cucsa.237070 and Cucsa.249360, were missing from the 9930 genome; whereas each of the three RGHs, Csa5P647620, Csa5P647590, and Csa5P647550 in the 9930 genome was corresponding to two RGHs in the Gy14 suggesting different annotations of these sequences in the two genomes.

**Table 1:** Number of NB domain-containing RGHs with homology to plant resistance proteins in the Gy14 cucumber genome

**Predicted protein domains**	**RGH numbers**	# in clusters*
CC-NB-LRR (CNL)	25	10 (3)
CC-NB (CN)	1	0
TIR-NB-LRR (TNL)	19	14 (2)
TIR-NB (TN)	5	2 (1)
NB-LRR (NL)	17	7 (3)
NB (N)	3	3 (1)
Total	70	36 (10)

* Numbers in parentheses were number of clusters.

We defined a RGH cluster as a genome DNA region less than 1Mbp that contained two or more RGH members. Clustering of RGHs in the cucumber genome was obvious. Among the 70 RGHs, 52 (74%) were located in nine clusters, which were consistent with the Gy14 scaffolds. Characteristics of these clusters are summarized in Table 2 and more details are presented in Additional file 2: Table S1. The two scaffolds, scaffold00894 and scaffold02023, had the most RGH loci, which were 11 and 12, respectively, whereas scaffold00919 had the highest RGH density: ~ 11 RGHs per 100 kb genomic DNA sequences (Table 2). RGH members in some clusters seemed to be heterogeneous in the classes to which they belonged (Additional file 2: Table S1). That is, except for the cluster in scaffold02229, no other clusters contained RGHs that were annotated in the same class. Consistent with the clustering of RGHs, their chromosomal distribution was clearly uneven. There were 4, 24, 11, 6, 16, 2 and 7 RGHs in cucumber Chromosomes 1 to 7, respectively (Additional file 3: Figure S1).

**Table 2:** Characteristics of NB-containing RGH sequence clusters in the Gy14 genome

**Cluster Chr #**	**Location **^**a**^	**Gy14 scaffolds**	**# RGHs in cluster**	**Physical span of cluster (kb)**	**RGH Density (#RGHs/100 kb)**	**Genetic span of cluster (cM)**	**Syntenic cluster in melon **^**b**^
1	Chr2	scaffold01037	2	857.3	0.2	1.4	No
2	Chr2	scaffold00894	11	288.8	3.8	1.8	Yes (10)
3	Chr2	scaffold01227	3	413.4	0.7	2.0	Yes (2)
4	Chr2	scaffold00245	4	109.5	3.6	2.5	No
5	Chr3	scaffold02229	2	59.6	3.4	0.0	No
6	Chr3	scaffold03356	7	1143.1	0.6	5.9	Yes (3)
7	Chr4	scaffold00919	5	43.4	11.5	0.6	Yes (4)
8	Chr5	scaffold02023	12	258.3	4.6	0.5	Yes (7)
9	Chr7	scaffold01024	6	94.4	6.4	0.0	Yes (6)

^a^ Chr = chromosome or linkage group.

^b^ Yes/No = there is (no) corresponding NB-LRR type RGHs annotated in syntenic blocks of the melon genome. Numbers in parentheses are number of RGHs annotated in the corresponding cluster. Melon annotation data were from Garcia-Mas *et al.* (2012) [56]. Cucumber and melon chromosome synteny is based on Li *et al.* (2011) [57].

*In silico* analysis of RGH gene expression was conducted with BLAST against EST contig assembly from 220 million Illumina/GA reads from 10 tissues of cucumber inbred line 9930 [26], as well as 2.3 million Roche/454 raw reads of the Gy14 root and leaf tissues (Weng *et al.* unpublished data; assembly is available at http://cucumber.vcru.wisc.edu/). The BLAST alignment result is summarized in Additional file 2: Table S2. As compared with the Gy14 leaf and root transcriptome, the 9930 RNA-Seq dataset had more depth coverage of the cucumber transcriptome, and showed more BLAST hits with better query sequence coverage. Thus, while 65 of the 70 NB-LRR sequences had hits in the Gy14 leaf and root transcriptome, all of them seemed to have EST representations in the 9930 EST data set. From the number of reads with BLAST hits of the Gy14 leaf and root EST collection (Additional file 2: Table S2), the expression level of different RGHs varied significantly; tissue-specific expression of some RGH genes was also very clear.

**Figure 1:**
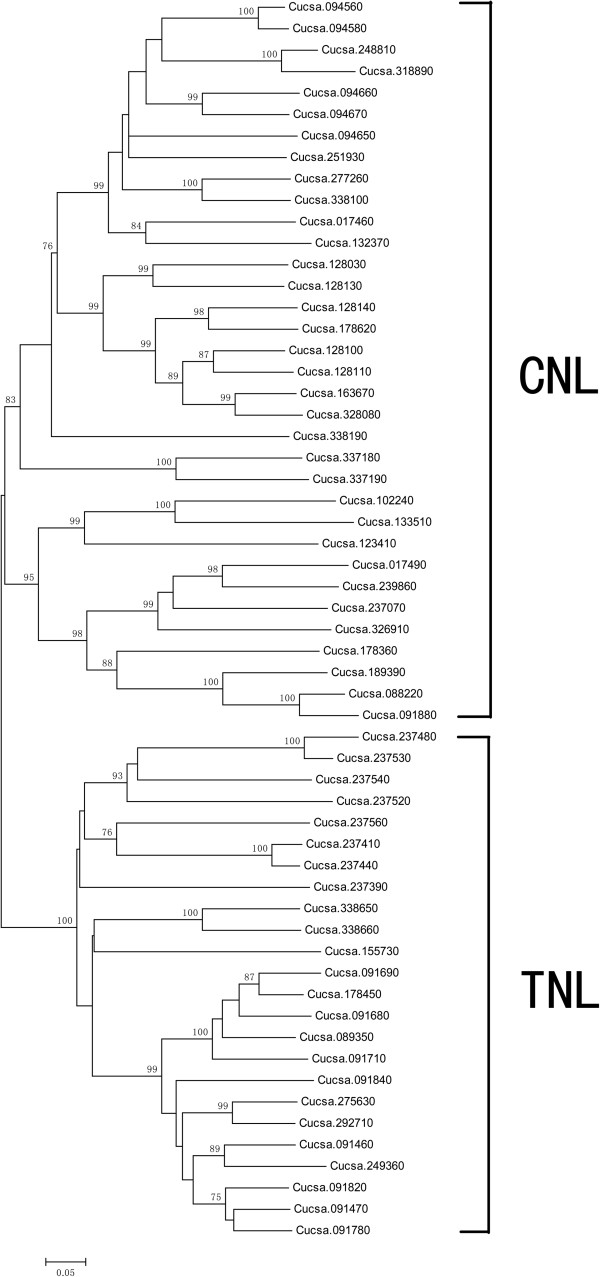
**Phylogram of the NB-LRR proteins in the Gy14 cucumber genome.** The neighbor-joining tree was constructed with 58 NB-containing protein sequences using MEGA5 software. Sequences were trimmed to extract just the NB domain. One thousand bootstrap repetitions were used, and support value (in percentages) for each node is indicated on the branch.

The amino acid sequence of the NB domain (~120 amino acids from the P-loop motif to the Kin3 motif) of each predicted NB resistance protein was extracted and used to perform a phylogenetic analysis. Proteins with incomplete NB domains were excluded. The NB domain sequences of 58 NB RGHs (Additional file 2: Table S1) were aligned and the resulting neighbor-joining phylogenetic tree is shown in Figure 1. Two clades, CC-NB-LRR (CNL), and TIR-NB-LRR (TNL) were evident, which contained 34 and 24 members, respectively. All CNL and CN class RGHs were grouped into the CNL clade, and all TNL and TN RGHs into the TNL clade. Meanwhile, all N and NL type RGHs were dispersed in the two groups (Figure 1).

### Homology and synteny of NB-LRR RGH sequences between the cucumber and melon genomes

The annotated melon draft genome has been released [56]. Sequences of the 70 cucumber RGHs identified herein were BLAST-aligned against the melon draft genome. Information on chromosome locations and draft genome scaffold positions in cucumber and melon as well as alignment scores of these RGHs is presented in Additional file 2: Table S3. Of the 70 cucumber RGH sequences, two (Cucsa.326910 and Cucsa.017500) had no BLAST hits in the melon draft genome, and four had low alignment scores (Additional file 2: Table S3). For the remaining 64 RGH sequences, each had a match sequence in the melon genome with a minimum 90% sequence identity and 90% sequence coverage of its whole length suggesting high degree of homology of these NB-LRR type RGHs between the two genomes.

Garcia-Mas *et al.*[56] identified 81 NB-LRR type RGHs in the melon draft genome, 37 (45%) of which were in six clusters. The locations and classes of these RGH clusters in the cucumber and melon genomes were highly conserved. Based on their scaffold positions and linkage map locations, five RGH clusters in the Gy14 genome, scaffold00894 + scaffold01227 (Chr2), scaffold03356 (Chr3), scaffold00919 (Chr4), scaffold02023 (Chr5), and scaffold 01024 (Chr7) (Table 2) corresponded very well with the five RGH clusters in melon chromosomes V, IV, VII, IX, and I, respectively (Additional file 2: Table S3). The number and class of RGHs in corresponding cucumber and melon clusters were largely consistent (Additional file 2: Table S3). Meanwhile, the melon ortholog sequences of cucumber RGHs in cucumber scaffold00245 (Chr2), scaffold01037 (Chr2), and part of scaffold03356 (Chr3) were not annotated as RGHs in the melon genome (Table 2, last column). Conversely, the only melon RGH cluster without its ortholog in cucumber was Cluster #13 (with four RGH members) located in CM3.5_scaffold00079 of melon Chromosome IX [56].

The syntenic relationships of cucumber and melon chromosomes have been largely established [56,57]. Among the 65 RGHs in cucumber with orthologs in the melon genome, 57 were located in syntenic blocks (Additional file 2: Table S3). We randomly selected six NB-LRR sequences from the melon genome (MELO3C010346T1, MELO3C004289T1, MELO3C004292T1, MELO3C009694T1, MELO3C022146T1, MELO3C023579T1) to verify their orthologous relationships in both cucumber and melon genomes by BLAST alignment, and all of them had single copy in the melon or the cucumber genome (Additional file 2: Table S3).

### Genetic mapping of RGH loci in cucumber

Using 92 F_2_ plants of Gy14 × 9930, Yang *et al.*[27] developed a high-density linkage map of cultivated cucumber with 735 marker loci (CSS-F2 map). To anchor the 70 RGHs identified herein onto this genetic map, markers for all RGH or RGH clusters were developed and used in linkage mapping of the RGH loci.

DNA sequences of the 70 RGHs between Gy14 and 9930 (Version 2.0) were aligned to identify polymorphisms. Eighteen RGHs showed no polymorphisms (Additional file 2: Table S1). For these RGHs, flanking markers were developed, with which 15 RGHs were successfully anchored. Three RGHs, Cucsa.189390, Cucsa.318890, and Cucsa.328080 (Additional file 2: Table S1) failed to be anchored to the genetic map because the scaffolds in which they resided were relatively short and no polymorphic flanking markers were identified. For the remaining 52 RGHs, single nucleotide, insertion/deletion (indel) or SSR polymorphisms were identified. When possible, SSR markers were preferred to SNPs to tag these RGHs. Seventeen RGHs had SNP or indel polymorphisms within the target RGH sequence. SNP-derived dCAPS or indel-derived STS markers were developed as molecular tags for these 17 RGHs without development of additional flanking markers (Additional file 2: Table S1). Since a number of SSR markers on the Gy14 × 9930 F_2_ high-density map [27] were physically very close to many of these 35 RGHs left, new markers were developed only for those RGHs without close flanking markers. However, for a cluster with multiple RGH members, only markers flanking the cluster were developed. Eventually 54 markers were employed to delimit and anchor 67 RGHs, of which 28 were newly developed from the present study (Additional file 2: Table S1) and 26 were from Yang *et al.* (2012) [27]. Meanwhile, during the polymorphism screen stage of this study, 20 additional new markers (48 in total) were developed but not used to delimit the RGHs because these markers were less close to target RGHs than other ones listed in Additional file 2: Table S1.

**Figure 2:**
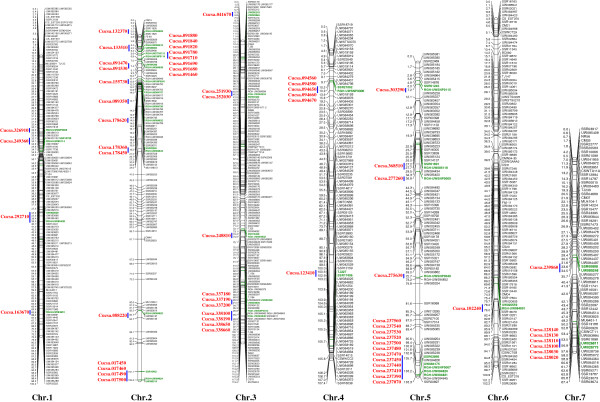
**Graphic view of high-density genetic map of cultivated cucumber developed with the Gy14 × 9930 F**_**2 **_**mapping population showing locations of 67 RGHs and molecular tags on the map.** Numbers on top of the map are linkage groups (LG) (1 through 7) which also correspond to chromosomes numbers. Cumulative map distance (cM) is shown to the left of each linkage group and marker designation is on the right. RGH loci are in red. Markers in green are molecular tags for corresponding RGHs.

For linkage analysis, genotypic data from the 48 new RGH markers for the 92 F_2_ plants of the CSS-F2 population were combined with the data of previously mapped 735 markers [27]. The resulting linkage map contained 783 loci in seven linkage groups. Brief statistics of this map is presented in Table 3 and graphically presented in Figure 2, in which 54 molecular marker loci delimiting 67 RGHs were also highlighted. The genetic map and physical scaffold locations of all RGHs were highly consistent (Additional file 2: Table S1 and Additional file 2: Table S4) suggesting high reliability of the mapping data. The addition of 48 new markers slightly shortened the map length of this high-density genetic map by 1.1 cM, and provided a road map for 67 NB-LRR RGHs. Details of all markers, their genetic and physical locations in the cucumber genome are presented in Additional file 2: Table S4.

**Table 3:** Summary of the cucumber consensus map and its three component genetic maps used for map integration

	**# of mapped loci **^**a**^				**# shared markers**			**Map length (cM)**				**Marker density of consensus map **^**e**^	**Gy14**		**9930**	
**Chr**^**b**^	**Map A**	**Map B**	**Map C**	**Consensus**	**A vs. B**	**A vs. C**	**B vs. C**	**Map A**	**Map B**	**Map C**	**Consensus**		**# scaffolds anchored**	**Physical length (Mb)**	**# scaffolds anchored**	**Physical length (Mb)**
**Chr1**	118	155	17	241	36	13	10	96.2	100.4	115.7	102.8	0.43	61	29.3	45	32.4
**Chr2**	126	93	33	214	18	21	9	100.2	103.6	88.0	108.9	0.51	33	25.9	35	24.8
**Chr3**	187	167	54	316	50	31	18	112.7	119.4	137.5	121.9	0.39	45	41.5	63	43.1
**Chr4**^**c**^	114	104	15	n/a	13	9	2	37.3	107.4	50.5	n/a	n/a	n/a	n/a	n/a	n/a
**Chr4**^**d**^	41	105	37	220	15	9	13	96.0	107.4	107.9	106.3	0.49	43	22.0	36	24.0
**Chr5**	160	84	57	250	12	33	9	59.9	106.3	118.3	101.0	0.40	50	25.9	38	28.1
**Chr6**	203	113	54	295	38	33	19	106.5	102.2	111.4	110.7	0.38	43	30.4	36	30.1
**Chr7**	87	67	25	145	18	14	5	60.1	67.4	90.1	78.4	0.54	33	18.4	22	19.8
**Total**	**995**	**783**	**255**	**1681**	**185**	**152**	**72**	**572.9**	**706.7**	**711.5**	**730.0**	**0.44**	**308**	**193.4**	**275**	**202.3**

^**a**^ MapA = Gy14 × PI 183967 RIL (CSH-RIL) [42]; MapB = Gy14 × 9930 F_2_ (CSS-F2 map) (Additional file 2: Table S4, Figure 2); MapC = 9110Gt × 9930 RIL (CSS_RIL) [45].

^**b**^ Chr = Chromosome or linkage group.

^**c**^ Marker loci of linkage group (Chromosome) 4 from the Gy14 × PI 183967 RIL (MapA) and 9110Gt × 9930 RIL (MapC) maps were not used in map integration. Numbers in this row were listed for comparison purpose only. n/a = not applicable.

^**d**^ The consensus map for Chromosome 4 was integrated from two intra-varietal cucumber component genetic maps: the PI 249561 × PI 308915 F_2_ map (CSS-PI-F2 Map - MapA) [28] and the Gy14 × 9930 F_2_ map (CSS-F2 map - MapB) (Additional file 2: Table S4, Figure 2).

^**e**^ Average genetic distance between adjacent markers (marker interval) in centimorgan.

It is known that the ratios of genetic to physical distances varies significantly in different regions of the cucumber chromosomes (e.g., Li *et al.*[28]). This was further evidenced in the RGH cluster regions in the present study. The genetic coverage (in cM) of each RGH cluster is presented in Table 2, which, when compared with respective physical span, clearly indicated very different recombination rate among these clusters. Thus, this genetic mapping effort provided good approximation of genetic recombination in each cluster, which should be helpful in map-based cloning of R genes in cucumber.

### Consensus map construction

Two steps were taken to integrate the intra-varietal CSS-F2 (783 loci with 92 F_2_ plants of Gy14 × 9930) (Additional file 2: Table S4 and Figure 2), the CSS-RIL (255 loci with 148 RILs of 9110Gt × 9930) [45] maps, as well as the inter-subspecific CSH-RIL map (995 loci with 77 RILs from Gy14 × PI 183967) [42]. First, common markers were identified and their orders were compared for each linkage group (Additional file 3: Figure S2). The CSS-F2 map shared 72 and 185 markers with the CSS-RIL and CSH-RIL maps, respectively (Table 3). In selection of markers for bin mapping, chromosomal locations of three markers, SSR02693, SSR04454 and SSR04905 were inconsistent between the CSS-F2 and CSH-RIL maps. Map locations of SSR01981 did not agree between the CSS-F2 and the CSS-RIL maps. The CSH-RIL and CSS-RIL maps shared 152 markers with four (SSR01981, SSR19728, SSR21834, and CMCT160a) inconsistencies in chromosomal locations. Based on the scaffolds with which these markers were associated and their map locations on different genetic maps, the chromosomal locations of SSR01981, SSR02693, SSR04454, and SSR04905 on the CSS-F2 map, and SSR19728, SSR21834, as we all as CMCT160a on the CSS-RIL map were considered correct. Details (map locations, scaffold information etc.) of these and other markers (see below) with discrepancies in chromosome locations among the component maps are summarized in Additional file 2: Table S5.

In total, 419 and 387 markers were selected from the CSH-RIL and CSS-F2 maps, respectively for bin mapping; all the 255 markers on the CSS-RIL map were employed for map integration. To reduce the effects of marker clustering on map integration, markers in the four clusters in Chromosomes 5 and 7 of the CSH-RIL map were excluded from bin mapping.

The orders of shared markers among the three maps in respective linkage groups were quantitatively assessed with Spearman’s rank correlation coefficients (r), and the result is shown in Additional file 2: Table S6. The orders of common markers in Chromosomes 1, 2, 3 and 6 between each pair of maps were strongly positively correlated (average r > 0.96). In contrast, marker orders in Chromosomes 4, 5 and 7 showed low correlations between the CSH-RIL and the other two maps, which was mainly due to the clustering of markers on the CSH-RIL map.

Reliable map integration for Chromosome 4 among the three component maps was difficult. On the CSH-RIL map, 144 loci were mapped in LG4, but only covered 37.3 cM due to recombination suppression and marker clustering [42]. Only 15 markers were placed in LG4 on the CSS-RIL map [45] with a map length of 50.5 cM. LG4 of the CSS-F2 map shared 13 and 2 markers with the above two maps, respectively. The consensus map of Chromosome 4 integrated from the three individual maps shrank to 83.7 cM and many markers were out of order when compared with their orders on individual maps and in the Gy14 draft genome assembly (data not shown). In addition, using the Gy14 draft genome assembly (Version 1.0) [27] as the reference, this map integration resulted in a large inversion in the distal region of Chromosome 4 that could not be resolved. For these reasons, map integration for Chromosome 4 was performed using the CSS-F2 map (104 loci spanning 107.4 cM) and an intra-varietal F_2_ genetic map developed from PI 249561 × PI 308915 (42 markers in 96.0 cM, CSS-PI-F2 map hereinafter) [28]. All markers selected for bin mapping are indicated in Additional file 2: Table S7 (in the 'Bin' column).

The resulting consensus skeleton map contained 487 bins in seven linkage groups (chromosomes) (Additional file 3: Figure S3). Next, all the remaining markers including all molecular tags for 67 RGHs on the component maps were assigned to the consensus bin map based on their original bin positions. For Chromosome 4, refilled markers included not only those residual markers from the CSS-F2 and CSS-PI-F2 maps, but also those from the CSH-RIL and CSS-RIL maps. There were 33 markers with conflicting chromosome locations among the CSS-F2, CSS-RIL and CSH-RIL maps (listed in Additional file 2: Table S5). Based on the scaffolds with which these markers were associated, and the chromosome locations of other mapped markers from the same scaffold, the chromosome locations of these 33 markers on the CSS-F2 map constructed herein were considered correct and were assigned to the consensus map (Additional file 2: Table S7).

The final cucumber consensus map contained 1,681 marker loci including markers delimiting 67 NB-LRR type RGHs and ten gene loci, which is the densest genetic map of cucumber ever constructed. Among the markers mapped, 1,640 were developed from the cucumber genome, and 41 from melon. Nearly all (1,656 of 1,671) of the mapped markers were co-dominant SSRs. The cucumber genes on this consensus map included *bi* for bitterfree, *Ccu* for scab resistance, *cp* for compact plant growth habit, *d* for dull fruit skin, *F* for gynoecy, *fr* for fruit ribbing, *H* for heavy netting of mature fruit, *m* for bi-sexual flower, *u* for uniform immature fruit colour, and *v-1* for virescent leaf [28,32,45,58]. Details of this cucumber consensus genetic map are presented in Additional file 2: Table S7 and summarized in Table 3. The total genetic length of this integrated map was 730 cM in seven linkage groups (Chromosomes 1 to 7) with an average distance of 0.44 cM between adjacent markers. On average, there were 240 markers per chromosome, and Chromosome 3 had the largest (316) number of markers. Comparison of the marker orders between three component maps and the consensus map indicated high degree of accordance across all seven chromosomes as evidenced from the Spearman’s rank correlation coefficient (r) of marker orders (Additional file 2: Table S6). The Chromosome 4 consensus map was significantly improved in both marker numbers and map length as compared with the individual maps.

Physically, 308 and 275 scaffolds of the Gy14 and 9930 draft genome assemblies were anchored onto the integrated map covering 95% (193.2 Mb) and 83% (202.4 Mb) of the two draft genome sequences, respectively (Table 3). Locations of all mapped loci on this consensus map in the Gy14 draft genome assembly Version 1.0 are provided in Additional file 2: Table S7. Of the 1,681 markers, only 102 did not have any *in silico* PCR product or BLAST hits in the Gy14 and/or 9930 draft genome scaffolds. The order of loci on the consensus linkage map was largely consistent with their physical locations in the Gy14 draft genome assembly indicating that this integrated genetic map is highly reliable (Additional file 2: Table S7).

## Discussion

### Number and distribution of NB-LRR type RGHs in the cucumber genome

We identified 70 NB-LRR type RGHs in the Gy14 draft genome (Additional file 2: Table S1). The number of NB-LRR type disease resistance genes in the cucumber genome was significantly lower than in species such as *Arabidopsis thaliana* (212) [6], rice (535) [10], grapevine (459) [12] and potato (435) [8], but was simiar to that in papaya (55) [16] and melon (81) [56]. The low number in papaya was believed to be due to lack of whole genome duplication (WGD) during evolution of its genome [16]. In the grapevine genome which didn’t undergo WGD either [59], the recent expansion by tandem duplications was proposed to be the reason for the large numbers of NBS-encoding genes [12]. Huang *et al.*[23] noticed that the lipoxygenase (LOX) gene family has been notably expanded in the cucumber genome and proposed that this might be a complementary mechanism to deal with biotic stresses. On the other hand, while the melon genome has small set of NB-LRR type RGHs, the total number of resistance genes including the RLK (receptor-like kinases), KIN-GNK2 (receptor-like kinases-ginkbilobin-2 domain), RLP (receptor-like proteins), Pto-like or MLO-like RGHs was not significantly fewer than *Arabidopsis* or the grapevine [56]. Therefore, the reason why cucurbit crops have fewer NB-LRR type resistance genes merits further investigation. Nevertheless, these genetically mapped NB-LRR RGHs from present study should be useful in map-based cloning or association mapping studies of disease resistance genes in the cucumber genome. For example, the single dominant scab (*Cladosporium cucumerinum*) resistance gene, *Ccu*, has been fine mapped [32] which is co-localized with the large NB-LRR cluster (in Gy14 scaffold00894, Additional file 2: Table S1) in the short arm of cucumber chromosome 2 (Figure 2).

Expression studies of cloned R genes detected low levels of transcripts in unchallenged plants (i.e., constitutive expression) [60,61]. Tan *et al.*[62] analyzed the expression patterns of ~170 NB-LRR and related genes in *Arabidopsis*, and found that most of these genes were expressed at low levels with a variety of tissue specificities. It is known that disease resistance is the predominant function for plant NB-LRR-encoding genes [1], but their other biological roles should not be precluded. In this study, by examining the large collection of RNA-Seq data, we found EST representations of all 70 RGHs in the cucumber transcriptome; we also found that expression of a number of these genes was tissue-specific (Additional file 2: Table S2). These observations may suggest that all RGHs identified herein might be expressed in the cucumber genome. However, further investigations are needed to understand the functions of these RGHs and their tissue or organ specificities.

### Conservation of NB-LRR RGHs in the cucumber and melon genomes

Previous studies found that NB-LRR genes exhibited high level of inter- and intra-specific variation that presumably evolved rapidly in response to changes in pathogen populations [12,19]. Sequence conservation and synteny was found in several NB-LRR genes among Solanaceae species (tomato, potato, and pepper) [20,63]. Genome-wide comparison of NB-LRR-encoding genes between *Arabidopsis thaliana* and *A. lyrata* found that both species have similar numbers of NB-LRR genes [64]. In the present study, of the 70 cucumber RGHs, 65 had homologs and 57 were located in syntenic blocks of the melon genome (Additional file 2: Table S3). Among the nine RGH clusters in cucumber, six were also annotated to encode NB-LRR type RGHs in the melon genome, and all were located in syntenic blocks (Table 2, Additional file 2: Table S3). However, in the syntenic regions of the six cucumber clusters with 44 RGH members, only 32 RGHs were annotated in the melon genome (Table 2, Additional file 2: Table S3). It seems that annotation of the melon genome need to be refined in these regions to resolve these discrepancies. Nevertheless, it is clear that these RGHs are highly conserved in the cucumber and melon genomes in both sequence homology and chromosome locations suggesting a similar evolution history of these RGH ortholog in the two species, which were diverged approximately ten million years ago [65].

### The high-density consensus map of cucumber

We presented herein an SSR-based integrated genetic map of cucumber that was constructed using the segregation data from three populations. The aim of this map was to fill the large gaps on each individual map with markers that have been mapped in other mapping experiments and resolve marker orders in the recombination suppression regions on the CSH-RIL map. The cucumber consensus map consisted of 1,681 marker loci and the majority of these markers were anchored to the Gy14 and/or 9930 draft genome scaffolds (Additional file 2: Table S7). Among linkage maps constructed so far in cucumber, this integrated map has the highest marker density. As compared with the CSS-F2 map (Additional file 2: Table S4, Figure 2), this consensus map significantly increased the number of marker loci (from 783 to 1,681), marker density (0.96 cM to 0.44 cM), anchored Gy14 draft genome sequences (233 scaffolds, 173.1 Mbp versus 308 scaffolds, 193.4 Mbp), as well as total map length (706.7 cM vs. 730.0 cM) (Table 3). Similarly, this integrated map also showed several significant improvements over the cucumber consensus map by Zhang *et al.*[44] in number of mapped loci (1,681 vs. 1,369 loci), anchored genome sequences (193.4 Mbp vs. 172.5 Mbp of the 9930 draft genome), and map length (730.0 cM vs. 700.4 cM). The accuracy of marker orders on the present consensus map is also improved over the previous one [44]. This is especially true for markers in Chromosomes 4, 5 and 7, which could be seen from comparisons of the marker order on the consensus map and in the Gy14 draft genome assembly (Additional file 2: Table S7). Considering the very narrow genetic base of cultivated cucumber, map integration has allowed us to place a larger number of markers than possible on any individual maps, thus obtaining a more complete coverage of the cucumber genome. This consensus map integrated with 67 NB-LRR RGH loci should have broad potential uses in molecular mapping, gene cloning, quantitative trait loci (QTL) analysis, marker-assisted selection, comparative genomics, as well as whole genome assembly studies.

## Conclusions

**C**ucumber contains relatively few NB-LRR type RGHs that are clustered and unevenly distributed in the genome. As evidenced from their presence in the ESTs, all RGHs seem to be transcribed. These NB-LRR type RGHs shared significant homology in nucleotide sequences and high degree synteny in chromosomal location with the melon genome suggesting these RGHs are highly conserved in genome organization and functions in the *Cucumis* lineage. The high quality, 1,681-locus consensus map is the densest genetic map of cucumber ever constructed. The RGHs identified and characterized, and the high-density consensus genetic-physical map developed herein provide valuable genomics resources for many molecular marker-based studies such as mapping of quantitative trait loci, map-based gene cloning, assessment of genetic diversity, association mapping, as well as marker-assisted selection in molecular breeding in cucumber.

## Methods

### Identification and characterization of NB-LRR type RGHs in the cucumber genome

The Gy14 (Version 1.0) and 9930 (Version 2.0) cucumber draft genome assembly and annotation were employed in the present study [26,27] (Gy14 annotation is available at http://www.phytozome.net/cucumber.php#A).

The NB-ARC protein domain (accession ID: PF00931) sequence downloaded from the Pfam database (http://pfam.sanger.ac.uk) was used as the seed to extract all NB sequence homologs in the Gy14 draft genome by HMMER V.3 using the raw Hidden Markov Model (HMM) [66]. A total of 80 candidate sequences was identified from which a high quality protein set (<1e-60) was aligned with Clustal X (V2.0) [67] and used to construct a cucumber-specific NB HMM using the module “hmmbuild”, which was then used to identified proteins in cucumber genome with “hmmsearch” at a lower threshold (<1e-5) resulting in 70 proteins.

These NB domain-containing proteins were searched for the presence of signature domains of plant R proteins: the TIR or CC domains in the N-terminal region and LRR domain in the C-terminal region. To detect these conserved domains, NB-encoding proteins were characterized using Pfam version 26.0. SMART (http://smart.embl-heidelberg.de/) was used to confirm the identity of the TIR and LRR domains. Prediction of the CC domain was conducted using the COIL program (http://embnet.vital-it.ch/software/COILS_form.html) with default settings and a stringency of 90% threshold. RGHs with no TIR or LRR domains were verified by manual annotation. RGH DNA sequences including additional 2,000 bp up- and down-stream sequences were extracted from the Gy14 draft genome scaffolds and re-annotated with Augustus (http://bioinf.uni-greifswald.de/augustus/) using 9930 CDS (Version 2.0) [26] as reference sequences. NB-containing proteins were aligned using Clustal X with default parameters. Subsequently, Jalview (Version 2.0) [68] was used to trim at both ends to eliminate regions of poor alignment. For RGHs with complete NB domains, the amino acid sequence of each NB domain (approximately 120 AA from P-loop motif to Kin3 motif) was extracted and used to perform a phylogenetic analysis. Fifty-eight NB domain sequences (Additional file 2: Table S1) were aligned to construct a phylogenetic tree using the neighbor-joining method in MEGA 5 with a bootstrap of 1,000 replicates [69].

To describe the distribution of RGHs in the genome, a cluster of RGH was defined loosely as a chromosome region with a maximum 1Mbp sequences that had two or more RGHs. *In Silico* expression analysis of NB-LRR RGHs was conducted with BLAST alignment against two cucumber EST datasets. The first was the 9930 transcript assembly (Version 2.0) from nearly 220 million Illumina sequencing reads derived from 10 different tissues of cucumber line 9930 [26]; the second set included 2.3 million Roche/454 reads of Gy14 leaf and root tissues (available at http://cucumber.vcru.wisc.edu/) (Weng *et al.* unpublished data). BLAST hits with 95% sequence identity and 90% coverage of the query length were regarded as evidence of EST support of RGH gene expression in the genome.

### Comparative analysis of RGH sequence homology and chromosomal location synteny in the cucumber and melon genomes

From the draft genome sequence of melon, *Cucumis melo*, 81 NB-containing RGHs were identified [56]. To investigate sequence conservation and colinearity of RGH loci between cucumber and melon, the 70 cucumber RGH sequences were BLAST aligned with both the cucumber and melon genome assembly. The cucumber RGH query sequence and that of melon hits were considered orthologs when the sequence identify was >90%, the coverage was >95%, and no paralogous sequences in either genome. The synteny of chromosomal blocks of these cucumber RGHs in melon and cucumber were compared with current understanding of the melon-cucumber chromosome synteny [56,57].

### Genetic and physical mapping of NB-LRR RGHs in the cucumber genome

Since the scaffold information to which the 70 NB-LRR type RGHs belonged were known, it was relatively straight forward to map these RGHs to their physical locations in the Gy14 draft genome assembly (version 1.0) [27]. Three RGHs Cucsa.189390, Cucsa.318890, and Cucsa.328080) (Additional file 2: Table S1) were not anchored onto the map because their associated scaffolds were too small and no markers from these scaffolds were mapped on the high-density linkage map of cucumber.

For linkage mapping of RGHs, DNA polymorphisms (SNPs and Indels) within RGH and its surrounding DNA sequences between the Gy14 and 9930 cucumber lines were identified through alignment of the Gy14 [27] and 9930 [26] draft genome sequences. Indel-derived STS, SNP-based dCAPS or SSR markers were designed from target regions. In case there was no polymorphism within the RGH sequences, flanking markers of the target RGH locus were developed. Previously, a mapping population with 92 F_2_ plants from Gy14 × 9930 was used to develop a 735-locus high-density genetic map for cultivated cucumber [27]. This population was employed in this study to integrate RGH loci on the genetic map. Molecular marker analysis and linkage map construction followed Li *et al.* (2011) [57].

### Component genetic maps for consensus map construction

Three SSR-based cucumber linkage maps were used for consensus map construction in the present study. Two maps were developed with mapping populations from intra-varietal crosses including the Gy14 × 9930 F_2_ map from this study (CSS-F2 map, 783 loci) (Additional file 2: Table S4, Figure 2), and the 9110Gt × 9930 RIL map (248 SSR markers plus seven genes constructed with 148 RILs, CSS-RIL map) [45]. The third was the inter-subspecific Gy14 × PI 183967 RIL map (995 SSR loci mapped with 77 RILs, CSH-RIL map) [42]. The three maps shared a significant number of SSR markers. To give a quantitative assessment of the colinearity of these shared markers, Spearman’s rank correlation coefficients were calculated for marker orders between pairs of maps using the PROC CORR procedure in the statistical software SAS 9.3. The comparison of shared marker orders between individual maps was displayed graphically using the Circos program (http://circos.ca/) [70].

### Development of consensus cucumber linkage map

For all markers on three individual maps, *in silico* PCR was implemented using the Gy14 and 9930 draft genome scaffolds as templates to assign markers to scaffolds, reveal polymorphisms between the two parental genomes, as well as estimate copy numbers of expected PCR products. This was performed with a custom Perl script that used the NCBI BLASTN program as a search engine [24]. If no *in silico* PCR products were available, the primer sequences of each marker were employed in BLAST searching against the two draft genomes to identify their scaffold locations. If there were no or multiple *in silico* PCR products or BLAST hits, the marker was labeled ‘no hit’ or ‘multi-copy’ in the genome, respectively.

The quality of raw mapping data for individual maps was double checked. Markers showing unlikely local double crossovers were eliminated. Prior to map integration, population-specific bin maps were generated for each linkage group of the CSS-F2 and CSH-RIL maps using the procedure described previously [55]. A bin was defined as a unique location on the genetic map where one or more markers were within 1 cM genetic distance. In each bin, one or more markers were selected to provide a bridge to other population-specific map(s) (shared loci), or maximize the information content. All 255 mapped loci on the CSS-RIL map were employed in map integration. JoinMap 3.0 was used to generate pairwise recombination frequencies and LOD scores for the selected sets of representative loci for each linkage group, which were then combined into a single group node in the navigation tree. Within JoinMap3.0, the “Combine Groups for Map Integration” function carries out map calculations based on mean recombination frequencies and combined LOD scores.

On the CSH-RIL map, there were 247 markers in 10 clusters. Map integration for markers in the largest four clusters in Chromosomes 5 and 7 was conducted only between the CSS-F2 and the CSS-RIL maps. For map integration of markers in Chromosome 4, the CSS-F2 map and another intra-varietal cucumber F_2_ genetic map developed from PI 249561 × PI 308915 (CSS-PI-F2 map) [28] were employed. Bin map construction for Chromosome 4 followed the same procedure as other chromosomes.

After the consensus bin map (skeleton map) was constructed, residual markers in each original bin of individual maps including all molecular tags of 70 RGH loci were re-introduced and assigned to their respective bin positions on the integrated map. In addition, three cucumber genes, the *m* gene for bi-sexual flower expression [58], *cp* for compact plant growth habit [28], and *Ccu*[32] for scab resistance have been cloned or fine mapped. They were placed on this consensus genetic map based on their scaffold locations. Finally, based on marker-scaffold associations, the Gy14 and 9930 draft genome scaffolds were aligned onto the consensus genetic map to develop an integrated genetic-physical map.

## Abbreviations

AFLP: Amplified fragment length polymorphism; ARC: APAF-1, R proteins, and CED-4; BLAST: Basic local alignment search tool; CC: Coiled-coil; dCAPS: Derived cleaved amplified polymorphic sequence; EST: Expressed sequence tag; FISH: Fluorescence in situ hybridization; Mbp: Million base pairs; LG: Linkage groups; PCR: Polymerase chain reaction; NB-LRR: Nucleotide binding – leucine-rich repeats; RAPD: Random amplified polymorphic DNA; QTL: Quantitative trait loci; RGH: Resistance gene homolog; RIL: Recombinant inbred line; SNP: Single nucleotide polymorphism; SSR: Simple sequence repeats; STS: Sequence tagged sites; TIR: Toll, interleukin-1, and R proteins

## Competing interests

The authors declare that they have no competing interests.

## Authors’ contributions

LY conducted high-density genetic mapping, map integration and comparative analysis of RGHs between melon and cucumber. DL identified, characterized and mapped RGH loci. YL conducted genetic mapping in cucumber. XF and SH provide mapping data for integration of component maps. JG-M provided the sequence of the melon genome for NB-LRR comparison with cucumber. YW conceived the study, designed the experiments, conducted partial data analysis and wrote the manuscript. All authors read and approved the final version of the manuscript.

## Supplementary Material

Additional file 1 DNA and peptide sequences of 70 NB-containing R gene homologs in the Gy14 genome (in fasta format). Click here for file

Additional file 2 **Including seven supplemental MS Excel tables (Table S1 to Table S7). Table S1**. Information of 70 NB-containing RGH sequences in the Gy14 cucumber draft genome. Left and right markers are the closest flanking markers for each RGH. Map location indicates their positions on the high-density cucumber linkage map developed in the present study (Figure 2). Gy14V1.0 is the Gy14 draft genome assembly Version 1.0 [27]. Corresponding cucumber 9930 CDS data are from Li *et al.* (2011) [26]. **Table S2**. Expressed sequence tag (EST) representation of RGHs based on BLAST search of Gy14 leaf and root transcriptome assembly (//cucumber.vcru.wisc.edu/, Weng *et al.* unpublished data) and 9930 V2.0 transcript assembly derived from 10 tissues [26]. **Table S3**. BLAST alignment of cucumber RGH sequences and the melon draft genome showing sequence homology and syntenic blocks of RGH loci in the two genomes. Melon draft genome assembly and RGH annotation are from Garcia-Mas *et al.* (2012) [56]. Chr = chromosome. The melon scaffold positions of annotated RHG are approximations. **Table S4**. Information of 783 cucumber and melon markers placed on the high-resolution cultivated cucumber genetic map. Marker loci are arranged by increasing order of map locations in each linkage group (LG). The physical location of each marker in the new Gy14 draft genome assembly (Gy14_V1.0) [27], and the Gy14 scaffolds are also shown. The Gy14 V1.0 or Gy14 scaffold position is the first nucleotide-binding site of the left primer of each marker. **Table S5**. Markers with discrepancies in chromosomal locations between the Gy14 × 9930 F_2_ (CSS-F2) (**Table S4**), and the Gy14 × PI 183967 RIL (CSH-RIL) [42] or the 9110Gt × 9930 RIL (CSS-RIL) [45] maps. **Table S6**. Spearman’s rank of correlation (r) of marker orders among the three component maps and the final integrated map. **Table S7**. Information of 1,681 cucumber and melon markers on the consensus cucumber genetic map. Marker loci were arranged by increasing order of map locations in each chromosome (CHR). The physical location of each marker in the new Gy14 whole genome assembly (Gy14_Chr_V1.0), and their positions in the original 9930 and Gy14 scaffolds are also shown. Bins indicate marker loci that were selected from component maps for bin map construction prior to map integration. Chr_source, cM_source and Map_source were LG, map position (in cM) and reference of three component maps from which each marker was employed. Click here for file

Additional file 3 **Supplemental data file including three supplemental figures (Figure S1 to Figure S3). Figure S1**. Distribution of 70 NB domain-containing RGHs across seven cucumber chromosomes in the Gy14 genome. **Figure S2**. Graphic view of colinearity of common markers among three cucumber linkages used for consensus map construction. MapA = Gy14 × PI 183967 RIL (CSH-RIL) [42]; MapB = Gy14 × 9930 F_2_ (CSS-F2 map) (Additional file 2: Table S4, this study); MapC = 9110Gt × 9930 RIL (CSS-RIL) [45]. The graphs were drawn with the Circos software package (http://circos.ca/) [70]. **Figure S3**. Graphic view of consensus bin map of cucumber, which was developed from integration of three individual maps of Gy14 × 9930 F_2_ (CSS-F2) (Additional file 2: Table S4, this study), Gy14 × PI 183967 RIL (CSH-RIL) [42] and 9110Gt × 9930 RIL (CSS-RIL) [45] with JoinMap 3.0. Numbers on top of the map are linkage groups (LG) (1 through 7) which also correspond to chromosomes numbers. Cumulative map distance (cM) is shown to the left of each linkage group and marker designation is on the right. Click here for file
