# Supplementary material for: A 1,681-locus consensus genetic map of cultivated cucumber including 67 NB-LRR resistance gene homolog and ten gene loci
Source: BMC Plant Biol. 2013 Mar 25;13:53. doi: 10.1186/1471-2229-13-53 (PMC3626583; doi:10.1186/1471-2229-13-53)
Supplement: Additional file 1 — DNA and peptide sequences of 70 NB-containing R gene homologs in the Gy14 genome (in fasta format). [file 1471-2229-13-53-S1.pdf]

Additional file 1

Seventy NBS-containing R gene DNA and protein sequences from the Gy14 cucumber draft genome (fasta format)

```
>Cucsa.017450
ATGCTTGGGCAACGGTATTTTCTTGTGCTTGACGATGTTTGGAACGAAAATTCTTTTCTATGGAATGAGTTGAAATGTTG
TTTACTCAAGATCACTGAAAACTCTAAGAATAGTATTGTTGTGACTACAAGAAGTGCTGAAGTTGCAAAAATCATGGAAA
CATGTCATAGTTATTTTTTAAGTAAATTATCTAATGATCATTGTTGGTCCTTATTTAAAGAAAGTGCAAATGCATATGGA
TTATCCAATGACTTCAAACTTGGGGATCGTTCAAAAAGACAATGTTTTTCTTATTGTTCAATTTTTCCGAAGGATTTTGT
GTTTGAAAAACAAGGACTAGTTAAAATATGGATGGCACAAGGTTTTCTTCAACCACAAGAAGGAAGAAATGTGACGATGG
AAAATGTAGGAGACATGTACTTCAAGATCTTGTTGTCACAGTGCTTATTTCAAATTTCAAGTAATTCAATGAGATTAAAA
TTTCCAGAATCTATTGTTTCGCTTCATAATTTGCAAACATTGAAGTTCCTACACTCAAAGATTGAAGAATTTCCGATGAA
CTTTACAAATTTGGCAAGTTTAAGGCACTTGGAATTATGGGTAAGTTCTGACAAAACGCCTCTACATTTAAGTCGTTTGA
CTCAACTTCAAATATTGTCTCATTTTGTAGTCGGGTTTGAAAAAGGTTGTAAGATTACTGAATTGGGTCGATTGAAAAAC
TTGCAAGGTAGTTTGAGTCTTTTGTGTTCGGAGAAAGTTGAAAGTAAAGAGGAAGCCAATGGAGCAAACTTGGCAGAAAA
GGAGAATTTAAAAGAGCTGCACTTGAATTGGGATATGGAAAGAAAAGATAACAACAGTTACAATGATTTGGAGGTGTTGG
AAGGACTTCAACCAAACCAAAATCTCCAATCATTAATAATCCACAGCTTTGCAGAAAGACGTTTGCCTAACAAGATTTTT
GTTGAGAATTTAAGAGTGATACATTTGTATTCATCTTTTAATTGTGTAAAGCTTCCAATGCTTGGACAATTAAACAACCT
AAAGGAACTTGAGATTTACAGCTTCCTTGGTGTCCGAATTATAGACAACGAGTTCTATGGTAATGATCCAAACCAAAGAA
GGTTCTTCCCAAAGCTTGAGAAATTTGTAATGTATGAGATGATCAACTTAGAGCAATGGAAAGAAATAAGTAGATGTCCT
AAATTATTAAACATTCCAAAAGTTTTCTATGAAAATGATATGAGAAATAAGTCGAAATTGTGTTATTTAAACATTGGTCC
GTTGAGGAAGCTGCCAAAAGATTTATGTCATCTCATGAATTTGAGGGAAATGGAAATCGTTGGAAATATGCATAATTATG
ATTTTGGCATCCTTCAGCACCTTCCTTCCCTTAAACAAATTACTTTAGTCGAGGATGAGTTGAGCAACAATAGTGTAACG
CAAATTCCTCAACAACTCCAACACCTCACTGCCTTGGAATTTCTGTCCATTAAAAATTTTGGAGGCATTGAAGCTTTGCC
AGAATGGTTAGGAAACTTTGTATGTTTGCAAACACTCGGTCTTTCTAATTGCAAAAAATTGAAAAAATTGCCTTCTACAG
GAGCAATTCTACGTCTCACCAAATTAAAAAAATTGTATGCTTATAAATGTCCACAGCTACTACTTGACGAAGAAAAAAAA
AGATAG

>Cucsa.017460
ATGGCTGAGTTCTTATGGACTTTTGCTGTGCAAGAAATTTTGAAGAAGGTCCTAACTCTTGTGGCTGAGCAGATCATTCT
AGCAAGGGAGGTCAAGGATGTGCTACAACAACTACAAAAAGAGCTAGTTGAGTCTCAAAAAATTGTTAGTGCTATCACTA
CCCAAAGACAAAATCATTATTCACCAGATAGCTTAGTGACTCAGTGGGTGAATGATCTTCAACTTATTGTTCATGAGGCT
GACGACTTGCTGGATTTTATATTGAATAAAAATCAACCCATCGAGAGGTTATGGTCTGTGATCTCTTTATCTTGCCTGCT
ATATTCTTCAAACCCTGAGACTAAAAAAATGAAGGAAATTATAGCGTTGTTAAACAAACATTGCACCAAATTACCTCACT
TACTTCAACTTGAGCCTACACCCTCAAACATTGCAGAGACTGAAGTTGCGCAAATTCAAGAGACAGTCTCAAAGCCTGAA
GATTATGTGGTGGGAAGGAACAGGGAAGTTGAAACCATAGTTGATCGAGTGATTGATGCCAGCAAACAGGAACTCAATTC
TATTTTACCCGTTTTTGGAATGGGTGGATTAGGAAAAACCACTTTGGCAAAGTCGGTCTTCAACCATGATAGGATCAAAA
ATCATTTTGGTATCACTATTTGGATATATGTGTCACAACCTTTTGTCATCAACAACATTTTGCAAGCAATCTTACAAAAG
GTGGAGGTTCATTCTAGTGATTGCTCCAACAATAGGGAGGCCTTACTTGAAAAGCTTACAGAAAACATGGGAGAGAAAAC
ATATTTTCTTGTTCTTGACGATGTTTGGAATGAAAACAAAATGTTGTGGGAGAAGTTGAAGGAATGTTTGATGAGTATTA
CTCATATGTCAGGAAATAGTATTCTTGTCACTACAAGGAGCAGTGGAATTGCAAAAATGATGGAAGAAAATATTGGAAGT
CATGAATTAAGAAAATTATCTGATGACCAATGTTGGTCAATATTTAGGAACTTTGCCAATGCCAAGGATGTACCAATGAC
TTCCAATTTGGAGTTTGTGCAAAAAGAGTTTGATAAAAGAATCGGTGGTCTTCCATTAATCGCTAAAGTTTTGGGAGCAG
CAGTTCCATTTTCAGGAGACCATGACCAGTGGGTAGCAAATATAAAAAGCGTTCTAACAACTCCAATAAAAGAGGAAGAG
TTTGTTAAATTCACATTGAAGTTAAGCGTTGATCGTCTACCAAATGCTTCAGTAAAGCAGTGTTTTGCTTATTGTTCAAA
TTTTTCCAAGGGTTGTGAGTTTGACAAAAAGCAAGTGATTCGAATGTGGATGGCACAAGGATTTACTCAACCAGATGAAA
GAAACAATGAAACAATGGAAGATACAGGAGAAAGGTACTTTAACATCTTGTTGTCTTTCTGCTTATTTCAAGATGTTGTT
AAGAATGAAAGAGGAATAATTGAGAAGGTTCGAATGCATGATCTTATACATGATATTGCTTGTCAAGTTTCAAATGATAA
AAAGTTGCGAATAGATCACATCATTTCATCAAATTGGAAAGATTGGACGAAAGATGACAAAATACTTGTGAGCAAGTTAC
GAACAATAAATTTTTATGATCGTCATCATGTGGTGGTTCAGGATAAGATTGGGGACTTTACTGGTTTGCGTGTTTTGACA
ATTGAAAATTATATTGTTGAGGAGTTACCAAACTCAATATTCAAGTTGAAGCACTTGAGATATCTAGACATTTCGTATTG
TTATTCAATAAAGAAGCTTCCTGAATCTATTGTTCTGCTTTATAATTTGCAAACACTGAGATTTCATCTCTTAAGCAAGG
GATTTCTACCAAAAAACGTTGGACAAATGATTAGTTTGAGGCATTTGGAGTTCTCATCTATCGATAAACAAATGTCTCCC
TATTTGAGTCAATTGATTCAACTTGAAACATTGCCTAAATTTGCAGTAGGTTTTGAGAAGGGTTGTAAGATTACAGAACT
TGGGGTTCTAAGAAACCTGAAAGGGTTGTTGAAGCTTCAACGTTTAGAACATGTTGAAAGTAAAGAAGAAGCCGAAACTG
CAAAATTAGTGGAAAAGGAGAATCTAGAAGAAGTACATTTTGTGTGGACAAAGGAAAGGAAGAGAAAAGTAGAGAATAAG
AATGATTTGGAAGTGTTGGAAGGACTTCAACCACCCAAAAATGTAGAATATTTGAGAATCAAATACTTTTTAGGTGGGTG
TTTACCAAACCAGACGTTTGTTGAGAATTTAGTGAAAATAGAGCTAAGAGATTGTGGAAATTGTGAGAAGCTTCCAAGGC
TTGGGCAATTAGGAAATCTAGAGATACTTGATATTTCATGGTTTGAAAGAGTAAAGAGTATAGGGAATGAATTCTATGGA
AACAGCTCCAACAACCAAAGGAGTTTATTCCCCAGGTTGAAGGAATTGTATGTTGATGAGATGAGGAGGATAGGAGAATG
GGAAGAAGTGGGAAGTAATGTTAAAGCTTTCCCACGTCTTGAACGTTTGTATATTGGTTGTTGTAGAGATTTAGTGAAAA
```

TTCCAGATGTTTTTGGGTATTGTGATGAGTATGGTGAGAAGCATCTGGAAGTTGTGGAAATTATTGAACATTTGTG

>Cucsa.017490
ATGGACATTTTAGTTTCAGTCATTGCAGCAACAATTAAACCTATTGGACATCAATTAGGT
TACCTTGTTTGCTATAACAGAAACAAGAAGGAGCTTAGAGAACAACTTGAAAATCTTGAG
ACTACTAAAAAGGATGTGAATCAAAGGGTTGAAGAGGCAAAAGGCAAATCGTATACAATC
TCTGAGGAAGTTTCAAAGTGGTTGGCCGATGTGGATAATGCAATAACCCATGATGAGCTA
TCCAACTCCAACCCATCTTGCTTTAACTTGGCTCAACGATACCAGCTAAGTAGAAAAAGG
GAGAAGCAAGTGAATTATATTCTTCAACTCATGAACAAAAGAAACAGCTTTGTCGAAGTT
GGATATCGTGCACCTCTTCCGGATACTGAGAATACTGTTGTTCCCGGAGATTACCAAGTT
TTGGAATCAAAAACATTATTGGCTAAAGATATCAAGAATGCGCTTTCGAAACCTGAGGTC
AATAAGATTGGTGTTTATGGTATGGCAGGTGTTGGAAAAACTTATTTTCTTAACGAAGTT
AAGAAATTGGTGTTGAAAGGGGAAGACAGATTGTTTGATCGAGTTATTGATGTGCGTGTA
GGTCGATTTAATGATGTAACAGACATACAAGAACAAATTGGTGATCAATTGAACGTAGAA
TTGCCAAAAAGTAAAGAGGGAAGAGCGTCTTTTCTACGGAATAATTTGGCGAAAATGGAA
GGTAATATCCTCATTTTATTAGATGATTTGTGGAAGGAATATGATCTTTTAAAAGAGATT
GGGATTCCATTAAGTAAAGATGGATGTAAGGTACTCATTACAAGTCGATCACAAGATATA
TTAACCAATAATATGAATACACAGGAGTGTTTTCAGGTGAGTTCGTTATCTGAAGAAGAG
TCTTGGAAGTTTTTTATGGCAATCATTGGTGATAAGTTTGATACAATTTATAAGAAAAAC
ATTGCAAAGAATGTTGCAAAAGAATGTGGAGGGTTACCGCTTGCACTTGATACCATTGCA
AAAGCATTGAAGGGGAAAGATATGCACCATTGGGAGGATGCTTTAACCAAATTGAGAAAT
TCTATTGGAATGGATATTAAAGGGGTGAGTGACAAAGTTTATGCTTCACTTAGATTGAGT
TATGATCATCTAGATGGAGAAGAAACAAAATTAATATTTCTTCTTTGCAGCGTATTTCCA
GATGATTATAAGATTTCTATAAAAAATTTGCAAATGTATGCCATGTGTATGAGATTATTG
AATAAAGTAAAAACTTGGGAGGATTCAAAAAATAGGGTCATGAAGTTGGTTAATGATCTA
ATATCGTCTTCTTTACTTCTCGAGGCTGAGAGCGATTCAAAAGACAAGTATGTTAAAATG
CACGATGTGGTTCGTGATGTTGCGATACACATTGCATCCAAGGAAGGTAACATGTCTACA
TTGAACATTGGATATAATAAAGTTAATGAATGGGAAGATGAATGCAGAAGTGGTTCTCAT
CGTGCCATTTTTGCAAACTGTGATAACTTAAACAATCTTCCCCTAAAGATGAATTTTCCA
CAACTTGAGTTGTTGATATTAAGAGTTTCTTATTGGTTGGTGGAAGATAATCTTCAAATT
CCATATGCATTTTTTGATGGAATGGTAAAGCTCAAGGTTTTGGACTTGACAGGAATGTGT
TGCCTCAGACCATTGTGGACAACACCATCATTAAACAACCTTCAAGCATTGTGTATGTTG
CGTTGCGAATTTAACGACATTGATACAATCGGAGAGCTAAAGAAACTGGAAGTTTTGAGA
ATCGTTAAGTGTAACATGCTAGATCACTTACCTCCAACTATGAGTCAATTGACACACCTT
AAGGTACTAGAAGTTTTAAATTGCCCTAAATTGGAGGTGGTTCCTGCAAACATTTTTTCA
AGTATGACAAAACTCGAAGAATTGAAATTACAAGACAGCTTTTGTAGATGGGGAGAAGAA
GTATGGTACAAGGATCGATTGGTCAAGAATGTCACAGTTTCAGAATTGAATTGTCTGCCA
TGTCTATCTAATTTAAGTTTAGAAAGTTGGAATGTTAAGATTCTATCTGAAATAAGTTCA
CAAACTTGTAAGAAGTTAAAAGAATTTTGGATTTGTAGTAATGAATCAGATGATTTTATT
CAACCCAAGGTTTCTAATGAATATGCAAGAACCTTGATGCTTAACATTGAATCCCAAGTT
GGTTCAATTGATGAAGGACTTGAAATACTATTGCAAAGAAGTGAGAGATTGATTGTAAGT
GATTCAAAGGGTAATTTTATAAATGCAATGTTCAAGCCAAATGGAAATGGCTATCCCTGT
TTGAAGTATCTATGGATGATTGATGAAAATGGTAATTCAGAAATGGCACATTTAATTGGA
AGTGACTTTACTTCTCTAAAGTATTTGATTATTTTTGGGATGAAGAGATTGGAGAACATT
GTTCCTAGGCATATTTCACTAAGCCCTTTCAAGAAGGTTAAAACTATTGCAATTCAATTT
TGTGGGCAGATAAGGAATCTTTTCTCATTCTCTATTTTTAAAGACCTTTTAGATCTTCAA
GAGATTGAGGTGATTAATTGTGGTAAGATGGAAGGGATTATATTCATGGAAATTGGAGAT
CAACTCAACATTTGCTCTTGTCCTTTAACTTCTTTACAACTTGAAAATGTGGATAAACTT
ACAAGTTTTTGCACCAAAGACTTAATCCAAGAAAGTTCACAAAGTATCATTCCCTTTTTT
GATGGTCAGGTTTCATTTCCTGAGTTGAATGATTTATCAATTGTTGGAGGTAACAATTTG
GAGACGTTATGGCATAAAAATAATAACCCAACTACAGTTTTGTGGTCATTGAATGAGTTG
CATTTACTCAATCTACCAAATCTCAAACACGTATGGAGAAAAGACATCATCAAAATTTTG
ACATTTCCATCTCTAAAGAGAGTAAAAATCCATGGCTGTACTAAGCTAACACACGTCTGG
AAGGACAACAATAAAGTAACCAGAAGCTTTGATAGCTTGGAGAGGATTGAAGTAGAAAAA
TGCAAGAATTTGAAGTATTTACTGCCATCATCAATTGCATTCTTAAACCTGAAGGAGCTT
CACATCAAGAAATGTAATGGAATGATCAATTTGTTCAGCTCTACAGTGACAAAAAAGCTA
GTGAATCTCAGCTCCATTAAAGTATCTTATTGTAAAGGAATGAGATGCATGGTTGAAGTA
GATCAAGCAGAAAATGATGAAATTATTACTTTCAAGAAATTGAGTACGTTGGAATTAGAT
TATTTACCACGATTGGATAGCTTTTACTCTGGCAAATGCATGCTTGAGTTTCCCTGTTTG
GAGAGTTTGGTTATAAAAAGATGTCCTGAAATGAAGACATTTTCGTATGGAGTAATAATC
GCGCCAAGATTACAAACCTTGTGGATGAACGATAAAGAATTTGGAGTATCATCACCAGCA
TGTGGGATAAATGAAACCATACAAAATTTTCCGAGGCGAGTGGTATGTATGTTCAATTCT
AATTAA

>Cucsa.017500
TTACCCACGTCTATGAGTGAATTGAAAAAACTTAAGGTATTAGTTGTGTTGGATTGCTCCAATTTGGTGGTGATTCACAA
AAACATTATTTCAAGCATGACAAAATTAGAAGAGTTGGATATACAAGGCCGCTTTACGAAATGGAGAGAAAAAATTGTTT
CAGTCAACCCGACGAAACTTAGCATATTATTAGAAGGTGCTGAAGAGTTGGTGATTGTAAATGACTCCAAGGGTTTTGCA
AATAATATTTTTAAAGCAATTGGAAATGGTTATCCCCTGTTGAAGTATCTTAAAATTCTTGAGAATTCAGAGACACCACA
TTTGAGAGGAAATGATTTCACATCTTTGGAGTGGTTGGTTCTTAAAGGAATGGTGATGTTGGAGAGTATAGTTCCAAGGC
ATTCTCCAACAAATCCTTTCAACAAACTTAAAGTTGTAGAAATAACAAGGTGCAAGCAGCTGAGGAATTTTTTTTCACTC
TCTATTTTTAAAGGGCTTTCAAATCTTCGAGAGATTAAGATCTCTGAATGTGATATGATGGAGGAGATTGTATCAATAGA
AATTGAAGATCAGATCACGATTTGCACTTCTCCTTTGACATCTTTACACATTTGTCATATGAATAAACTTGCAAGTTTCT
GCAGTACCAAATCATCCATTCAACAAACAATTGTTCCTTTCTTTGATGAACGACGGGTTTCATTTCCTCAATTGGAGGAT
TTATCAATTTTTAGAGCAAACAATTTGGAGATGCTATGGCATAAGAATGGAACTTCCTTTTCCAAACTTCAAACAGTAGA
GATTAGTTATTGCAAGAAGTTGAGATGCGTTTTTCCTTCAAATATAGTGACGTCACTTGTCTCTTTAGATACATTGGAAA
TCAATTGTTGTGGGTTATTGGAAATGATATTTGAAATTGAAAAGCCGAAGACTTCGTGTGATACAAAAGTAGTGGTGCCA
TTGAGACACTTATATTTACAAGTTCTACCAAGTTTAAAGTACGTGTGGGACAAAGATGGTGACGATGTTGTGGCGTTTCC
AAATCTAAAGAAAGTTGAGGTTAGTCGTTGTCCTAAGTTGAGAAGTATTTTTCCACCTTCCTTCACCAAATACATGAAAG
AAATTGAAGAATTAATAGTGGAGGATGAGCCAATATTTCCAGTGGAGGATGAAGCATCAAAGTTAAAAGAGGTTGCATTG
TTCCAAAGCTTGAAAACATTGAAAATGAGTTGTAAGGAGGTTGTAGACGAGAGGTTTTGGGTTATGTCTAAGTTTTCCAA
ACTCAAACGTCTTGAATTGGTTGGTTGTGAAGATGATGATGATGATGATGATGATGATGATAAAATGATTAGCTTGCCGA
TGGAAATGAGTGAAGTATCATACAGCATTGAAGAATTGACAATTAGAGGATGCCTCCAGCTGGTACAAGTATTTGGAAAT
GACTCTTATATCCAAAGATGTGCAAATTTGAAGAACATGAATCAAATGACTGCAACCACATTCTCCAAGTTGGTTGATCT
TCAAGTACATGATTGCAATGGAATGATAAATTTATTTAGTCCTTCAGTGGCAAAGAATCTAGCGAATCTCAAGTCCGTTG
AAATATGTAACTGCAGAGAAATGACATCCATAGTTGCAGCAAAAGCAGAGGAAGAAGAGGAAAATGTTGAAATTGTCTTC
AACAATCTAACAAATATGGAATTTGATAATTTAGAAAGATTAGAATGGTTTTACTCTCGAAAATGCAGATTTGAATTCCC
CATATTAGATACGTTGAGGATAGACAAATGCTATGACATGAAAATCTTTTCATACGGAATTAACAAACACTCCCACTTTG
GAAAAGATCTTGATTGGAGGGTGGAACTCCTCTATTCCAATACTACCAACACAAGGGATAAATGA

>Cucsa.041670
ATGGATTCTGATGGGGTTGAATCAGAATCTACTCCGGCTATTTCCACCTGTCTCACCATTAAAATAGCTCCAACTTCCAG
TAAACCGCCGGGGACGTCTTCCGATTTGGCATTGCCGGAGCTCAAAAGCTCAATCGAATCATCTCCTTATAACTCCCCTT
CCCTTTTATCGCCGCCGTCATCGGCGTTTGTTTCGGCTTTGCAGTCGCCGTACATTTCGCCGAGGGCGGTGGTCCTTAAA
CCAGAAGAGAAGCCTATTCCGGCGGAGAGCACGGCGGCGCTGACTTGTCATTCACCGCTGGTTTCCCAATCTGAGGATAT
TCCGAGTAGTTCCTACACTCCTCCCTCGGACCAATATGAATATTCCGATGACCCTTCTGATTCTAAGGTTCAGTTTGTCG
CCTGTGTTCCGGTCCCCGACTCCGCCCCGCCTCGCATTTCGTTCTCCTTCCCCGTTCCTCGGACCTCCTTCGCCAAATGC
GGCGGCCCCCTCTCTCCTGTTTCCACCTCTAAACTCAGAAGCTGTGATGTCTACATTGGCTTTCATGGCCAAGCTAATGG
CTTGATACGCTTCTGTAAGTGGCTCAAATCAGAACTTGAACTTCAAGGAATTGCCTGCTTCATTGCTGACCGGTCCAAGT
ACTCCGATAACCAAAGCCACGAGATCGCTGACCGCGTTATCAGCTCAGTAACTTTCGGAGTCGTCGTCCTCACAAGCTCT
AGTTTCCATAACCATTTCACCTTGGAGGAGGTGAGATTCTTTGCACAGAAGAAGAACTTGATTCCATTCTTTTTCGACAT
GGAGTCGTCGGAGATCTCAAGCTTTCTCAACTATAATTCCATGGATAAAGAGTACAAAGAGACAGTGCAGGGATTGTTGA
GGTTCCATGAATACAAGTTGGAAGCCAATGAAGGTAATTGGAGAAGCTGTATAGCTAAAGCGGCTGGGATTTTGAGGGGA
AAGCTTGGGAGGATGAGTACTGAAAGTGATGTTGAAAGATATGAGGAGCTGCCATTTCCAAGAAACAGATGCTTTTTAGG
GAGGGAAAAGGAGATTATGGAAATGGAAGCTACTCTGTTTGGTAATCGAAGCTATCACAAGCAAGACGGTACGGTTTCTA
CTCTGATCGTTGAAGGGAATTCCAGCCAGCAATCTGAAGGCTTAGCAGATGAAGAAAGTGAGCCCGTTAGTGTGAGGGGA
AGTAGGTTCATCAATTTGGAGATAGGGAGGTCTGATAATCCAACTTTGGAGACATGGATTGAACCAGTTAAAGGAAGAAA
TTCATTCAAGAGATCGAAGCACAAAGAAATGGTCAAGAGTGGGAATCACAAGAGCATGAGCAGCAGCATAGTGTGCATCA
ATGGAAACCCTGGAATTGGAAAGACAGAACTTGCTCTGGAATTTGCTTATAGATACTCCCAAAGATACAAGATGGTTTTA
TGGGTCGGTGGTGAAGCTCGGTACTTTCGACAGAATATATTGAACTTATCTTTGAACTTAGGGTTAGACATAAGTGCTGA
TGCTGAAAAGGATAGAGGACGGTTTCGGAGTTTCGAGGAGCAAGAACAAGAGGCATTCAAAAGAGTCAAGAGGGAGCTGT
TTGGAGACATGCCATATTTGTTAATCATTGATAATCTTGAGGCAGAAGAGGACTGGTGGGAAGGGAAAGATTTGAACGAT
TTGTTGCCGAGGAACACCGGAGGATCCCATGTGATCATCACTACGAGACTCTCTAAGGTGATGAGTTTTCGGATGATTAA
CATTCATCCATTGGCTTTGGCTGATGCAATGGTTTTGATGAGAGGAAGAAGGAAGAAAGAATACCCAGCAGATGAATTGG
AATATCTGAAGAAGTTCGATGAGAGGCTTGGGAGATTGACATATGGACTTTGGGTGATTGGATCGCTGCTTTGCGAGCTT
GCAATCACCCCGTCTTCTCTTTTTGAAGCTATTGAACAAGTACCCATCGATGAATGTTCTCCCTGCCCTTATATAAGCAT
AAACGAAGAGCACTACTGCAAAAGCAATCCCTTCCTGATGAAGATCATTTACTTTTCTTTTTCCATATTGGAGCAAACTA
ATGGACCGTTAGCATCCGGAATATTTCTGGTCGGTGCCTGGCTCGCCCCAGCACCCATCTCAGTGTCTGTACTAGCCACA
GCAGCAAAGGATATGGCTGTCTCAAGAAAAGGGTTTAAAATTTGGAGTAAATACTTGAGTTTCATGTTCGGTTGTTGCTC
CACTTGTTTAGCTTCACAAGCTTGGAAGAGTGAGGAAGAATCAGCTCTTCTTCTGATCAAGTTCGGGCTCGCCCGGAAGG
CGAACAAGCAAACTGGTAGTTGGATCCAATTCCATCCCATAACTCAAGTGTTTGCCAAAAGAAAGGAGGGTTTATCAGCT
GCCAAGTCTATAGTTCAAGGGATAAGGAAATGTAGTAGTAACACAATGGCAAACTTGGATCATTTATGGGCATCTGCATT
TCTCGTTTTTGGTTTCAAATCTGAACCTCCATTTGTACAACTAAAGGCTGTTGATATGGTCCTATACATAAAAAAGGCTG
CCCTTCCTTTGGCGATTCGGGCCTTCACAACCTTTTCGAGATGTAACTCAGCACTAGAATTGCTAAAGGTATGCACAAAT
GCACTTGAGGAAGTAGAGAAGTCATTTGTATCACAAATACAAGACTGGTGTGAAGGGTCCTTGTGTTGGAAGAAAAAGTT
CCAAGGCTATCAACGAGTTGATGAATACGTGTGGCAAGATGTGACGTTGCTAAAAGCTACATTGCTTGAAACTCGAGCGA

AACTACTACTAAGAGGTGGACACTTCGACAGCGCCGAAGAACTATGTAGAACTTGCATAAGTATAAGAACAGTCATGTTG
GGACATAACCATGCTCAAACCTTGGCAGCACAAGAAACGTTAGCGAAGATTGTTCGGCTTCGGAGCAAGATCTGA

>Cucsa.088220.1
ATGGACATCCTTGTTTCAGTCACTGCAAAAATTGCTGAATACACAGTTGTGCCCGTTGGACGTCAGCTTGGTTATGTAAT
TCACATTCATGCCAACTTTCAAAAACTTAAGACTCAAGTAGAAAAGTTGAAAGATACAAGAGAATCTGTGCAACAAAACA
TCTATACTGCAAGAAGAAATGCTGAAGACATAAAACCTGCTGTTGAGAAATGGTTGAAAAACGTTGATGACTTTGTTCGA
GAATCTGACAAGATATTAGCCAATGAAGGTGGACATGGTAGACTGTGTTCCACCAATTTAGTCCAACGACACAAGTTAAG
TAGAAAAGCAAGCAAAATGGCGTATGAGGTTAATGAGATGAAAAATGAGGGGGAAGGTTTTAATACGGTCTCCTATAAAA
ATGCTATTCCATCGGTTGATTGTTCACTGCAAAAAGTATCTGACTTTCTTGACTTAGACTCAAGAAAATTGACTGCGGAA
CAAATCATGGATGCACTCTCTGATGATAATGTCCATAGGATTGGAGTGTACGGGATGGGGGGTGTTGGTAAAACAATGTT
AGTGAAAGAAATTTTAAGAAAAATTGTTGAGAGTAAGTCTTTCGATGAGGTGGTAACATCCACGATCAGCCAAACACCAG
ATTTTAAAAGTATTCAAGGACAACTAGCTGACAAGCTAGGTTTGAAATTCGAACGAGAAACAATAGAAGGAAGGGCACCT
AGTCTACGAAAGAGGTTGAAGATGGAGAGACGTATCCTAGTTGTGTTGGATGATATCTGGGAGTATATTGATTTGGAAAC
AATAGGAATTCCAAGTGTTGAAGATCATACAGGATGCAAGATATTGTTTACCTCTAGGAATAAACATTTGATCTCAAATC
AAATGTGCGCCAATCAAATTTTTGAGATAAAAGTTTTAGGAGAAAATGAGTCATGGAATTTATTTAAGGCAATGGCTGGT
AAAATTGTTGAAGCAAGTGATTTGAAGCCTATAGCCATTCAAGTTGTGAGAGAATGTGCAGGTTTGCCTATTGCTATTAC
TACTGTTGCTAAGGCATTACGAAATAAACCTTCCGACATTTGGAATGATGCCTTGGATCAACTTAAAAGTGTTGATGTGT
TTATGACAAACATTGGAGAAATGGACAAGAAAGTGTATTTGTCACTAAAATTGAGTTACGATTGCTTGGGATATGAAGAG
GTCAAGTTATTATTCTTGTTATGCAGCATGTTTCCAGAAGACTTTAGCATTGACATGGAAGAGTTGCATGTATATGCCAT
GGGCATGGGTTTCTTACATGGTGTTGATACTGTGGTAAAAGGACGACGTAGGATTAAAAAATTGGTTGACGATCTTATAT
CTTCTTCTTTGCTTCAACAATATTCTGAGTATGGGTACAATTATGTGAAAATGCATGATATGGTTCGTGATGTAGCTATA
TTTATTGCGTCTAAGAATGATCACATACGTACATTGAGCTATGTGAAAAGATTAGATGAAGAATGGAAAGAAGAGAGACT
ATTGGGTAATCATACCGTGGTGTCCATTCATGGTTTACATTATCCTCTCCCAAAGTTAATGTTACCCAAAGTTCAATTAT
TAAGGTTGGATGGACAATGGTTGAATAATACGTATGTGTCAGTGGTACAAACTTTTTTTGAAGAAATGAAAGAGCTCAAA
GGTTTAGTATTAGAAAAAATGAATATATCCTTGTTGCAACGACCATTTGATCTTTACTTCTTAGCAAACATTAGAGTGTT
ACGTTTACGGGGATGTGAATTAGGGAGCATAGATATGATTGGTGAATTAAAAAGGCTTGAAATTCTCGATCTTAGTGGAT
CTAACATCATCCAGATTCCTACAACAATGGGTCAATTGACACAACTGAAAGTGTTAAATTTATCTAATTGTTTTAATAAG
CTCGAGATAATTCCACCAAATATTCTTTCAAAGTTGACAAAACTGGAGGAATTACGTATGGGAACTTTTGGTAGTTGGGA
AGGAGAAGAATGGTATGAAGGAAGGAAAAATGCTAGTCTTTCCGAGCTTAGGTTCTTGCCACACCTTTTTGATTTAGATT
TAACCATTCAAGATGAAAAGATTATGCCAAAACACTTGTTTTCAGCAGAGGAGTTGAATCTTGAAAAATTCCACATTACT
ATTGGTTGTAAGAGAGAAAGAGTTAAAAATTATGATGGAATCATTAAGATGAACTACTCTAGAATATTGGAAGTCAAGAT
GGAATCAGAAATGTGCTTGGATGATTGGATAAAATTTTTGTTAAAGAGGTCAGAAGAAGTGCATTTAGAAGGATCAATTT
GTTCAAAGGTTCTTAACTCTGAACTGTTAGATGCAAATGGCTTCTTACATTTGAAGAATCTCTGGATTTTTTATAATTCA
GACATTCAACATTTCATCCACGAAAAGAACAAGCCTTTGCGAAAATGCTTGTCCAAATTGGAGTTCTTATATCTTAAGAA
CTTGGAGAATTTGGAAAGTGTAATTCATGGTTATAATCATGGTGAATCTCCTTTAAACAATTTGAAGAATGTAATCGTAT
GGAATTGCAATAAATTGAAAACCCTATTTTTGAATTGCATGTTGGATGACGTTTTGAATCTCGAGGAAATTGAGATCAAT
TATTGTAAAAAGATGGAAGTGATGATCACTGTGAAGGAAAATGAGGAGACAACCAACCACGTTGAGTTTACTCATTTGAA
ATCTTTATGTCTATGGACTTTACCACAACTTCATAAATTTTGCTCCAAAGTTAGCAATACCATCAACACATGTGAATCAT
TTTTCAGTGAAGAGGTATCGCTTCCTAATTTGGAGAAGTTGAAAATTTGGTGTACAAAGGATTTGAAGAAGATATGGAGC
AATAATGTACTCATTCCCAATTCCTTTTCCAAACTTAAGGAAATAGACATTTATTCATGCAACAATCTTCAAAAAGCATT
GTTCTCTCCAAATATGATGAGCATTCTTACTTGCCTTAAAGTCCTAAGGATTGAAGATTGTAAATTGTTGGAAGGAATAT
TTGAAGTGCAAGAGCCAATTAGTGTTGTTGAAGCAAGTCCTATCGCCCTCCAAACTTTGAGTGAGTTGAAACTATATAAA
CTTCCAAACCTTGAGTACGTATGGAGCAAAGATTCCTGTGAGCTTCAGAGTTTGGTAAATATAAAACGTTTAACCATGGA
TGAATGTCCAAGACTTAGAAGAGAATATTCAGTCAAAATTCTCAAGCAACTTGAAGCACTAAGCATAGATATCAAACAAT
TGATGGAGGTTATTGGGAAGAAAAAGTCGACGGATTATAACAGGTTGGAATCAAAGCAATTGGAAACTTCTTCTTCCAAG
GTTGAGGTTCTACAGTTGGGAGATGGTTCTGAGTTGTTTCCGAAGCTTAAAACTTTGAAGCTATATGGTTTTGTTGAGGA
TAACTCAACCCATCTGCCAATGGAAATTGTACAAAACTTATACCAATTTGAGAAGTTTGAATTAGAAGGAGCATTTATTG
AAGAAATTCTCCCCAGCAACATATTGATTCCTATGAAAAAACAATACAATGCAAGACGATCTAAAACTTCACAGCGTAGT
TGGGTTCTATCCAAGCTACCGAAACTTAGGCATTTGGGGAGTGAATGCTCACAAAAGAATAATGATTCAATTCTACAAGA
TCTGACCTCTCTGTCCATTTCAGAATGTGGTGGATTGAGTAGTTTAGTGTCATCATCGGTGTCTTTTACGAACTTGACGT
TTCTTAAATTGAATAAATGTGATGGACTAACCCATTTGCTGGATCCTTCGATGGCTACAACGCTTGTGCAACTTAAACAG
TTGAGAATAGGAGAATGCAAAAGGATGAGTCGTATAATTGAGGGAGGATCATCAGGTGAAGAAGATGGAAATGATGCATC
ATTCAATTTCCATGTTTGA

>Cucsa.089350
ATGAATCGAGCAAGTGGATCATCTTCTTCCTCACCTTTTAGATGCAGTTTTGATGTATTT
TTAAGTTTTCGTGGTGAAGATACTCGTTCCAACTTCACCAGTCATCTTAATATGGCTTTA
CGTCAAAGAGGAATCAATGTTTTTATAGATAACAAGATTTCAAGGGGTGAAGAAATTTCT
GCATCTCTTTTGGAAGCTATTGAAAAATCCAAGATTTTGATCGTTATAATCTCTGAAAAT
TATGCATCTTCAAGTTGGTGTTTGAATGAACTAGAGAAAATCATTATGTGTAACGAATTG
AGATCAGGACAACTTGTTTTACCAATTTTCTACAGAGTGGATCCATCTGAAGTAAGAAAA
CAAAGTGGAAGATTTGGAGAAGAATTTGGGAGACTCGAAGTTAGATTCTCGTCGGACAAG

ATGCAAGCATGGAGGGAGGCCATGATTTATGTTTCTCAGATGTCTGGATGGCCGGTTCTT
CAAGAAGAGTATTTTTCTTCTTCACATATATCTTTTTACTCATTTGCTCTTCATATTATA
TCGTTACTATACTTCCACTATATTTCATCAAGTGATGAGGCCAATTTGATTCAAGAAATT
GTTCAAGAAGTCTTTAAGAAATTAAATCGTGGAATAATAATGCAGTTGCGTATACCTAAA
TATCCAGTTGGAATAGACATACAAGTTAATAATATACTCTTCCAAATTATGTCTGATAAA
AAAATTGTTATGCTTGGATTATATGGAATTGGAGGTATCGGCAAGACAACTTTGGCCAAA
GCATTGTACAATAGAATTGCTCATGACTTTGAAGGTTGTTGCTTTTTGGAAAAAATTAGA
GAAGCTTCAAATCAATATGACGGCCTTGTTCAACTCCAAAAGAAAATACTTTGTGATATT
CTAATGGATAATTCGATCAATGTTAGCAATCTTGATATAGGGGTTAACATCATAAGGAAT
CGACTATGCTCAAAAAAAATTCTTTTAATTCTTGATGATGTTGATACGAGAGAACAACTA
GAAGCATTAGCGGGAGGGCATGATTGGTTTGGACATGGAAGTAAGATCATTGCGACAACA
AGAAACATGCAATTACTTGCTAGTCATGGATTTAATAAATTGGAAAAAGTTAACGGATTG
AATGCCATTGAAGGTCTTGAGCTTTTTAGTTGGCATGCATTCAACAATTGTCATCCCTCA
AGTGATTATTTAGACCTTTCAAAACGTGCTGTACATTATTGTAAAGATCTTCCATTAGCT
CTTGAAGTGTTAGGTTCCTTCCTTAATTCTATTCACGATCAATCCAAATTTGAACGTATA
TTGGACGAGTATAAGAACTTCTATCTAGACAAAGACATCCAAGATATTCTTCGAATAAGT
TATGATGAACTTGAACAAGATGTAAAAGATATTTTCCTTTACATTTCTTGTTGCTTTGTA
GGAGAAGATATCAACGAAGTTAAAATGAAGTTAGAAGCATGTGGTTGTTTATGTTTGGAA
AAGGGAACAACAAAACTAATGAATTTATCACTTCTAACCATTGAATCCAATCGAATTAAA
ATGCATGACTTAATACAACAAATGGGTCGCTCAATTCATCTCTCGAAAACTTTTACATCT
CATAAAAGAAAAAGATTGTTGATTAAAGATGATGCTATGGATGTCTTAAATGGGAATAAG
GAAGCAAGAGCAGTTAAAGTTATAAAATTGGATTTTCCTCGACCTACCCAACTTGACATT
GATTCAAGAGCTTTTGAAAAAGTGAAAAATTTGGTAGTACTCGATGTTCGCAATGTCACA
TCTTCAAAAGGTACTGATCTTGAGTATCTACCTAGTAGCATAAGGTGGATGAATTGGCCT
CAATTTCCTTTTTCATATTTGCATACAAGCTTCACAATAGAGAACCTTGTCAAGTTCAAC
TTGCCATATAGCTCCATAAAAAAATTTGGGAAAGCATTAATGTGTGGTGAATGGTTGAAG
GAAATTAATCTTAGTTACTCTAAGTTTTTGGTGGAAATTCCAGATTTAACTACTGCAATA
AACCTCGAAAAGTTGAATCTTGAAGGGTGTGAAAAATTAGTAAAGGTTCATGAATCAGTT
GGATCTCTCAGTAAGCTTGTTGAGTTTTATCTTTCCAGCAGTGTTGAGGGTTTTGAGAAG
TTTCCATCCTGCCTTAAGTTGAATTCTCTTGAAGCTTTGGTAGTGAGATATTGTAGAATA
GAAGAATGTTGTCCTCAATTTAGTGAAGAAATGAATAGCCTAGAAATATTGGAGATCGAT
GATAGTATAATTAATCAATTATCTCCAACAATTGAATATCTTACTGGCCTAAAAGAACTC
TGGATCACAGAGTGCACGAAGCTCGAAACTCTTCCAAAAATTCTAAAGGTTCCAAAAGGA
GTAGTTCGTATGGATACTAGAGGGTGTGTATCATTGGCCAAATTTCCTAACAACATTCCT
GATTTCATATCTTGTGATGATAATGTGGAATATGATACAAAAGACGGAGTAATCAAACAA
CTCATATTAATGAATTGTGATATTCCAGATTGGTGCAAGTACAAGAGTATGAACAATTCA
GTAACGTTTGATTTTCTAGCTGATTATTTAAGTTGGAAAAGGAAGGCTTTTATTGCTCTT
TGTGTCAAATTTCATGTTACCAATGATCACGAGTTGGTTAAGCTTAATTGTGGAGTACTG
TTTATCAACGATATTGAAGTATGGAGTCGAATGTCAATCTCCAATTTTAACTTTTGGCTA
TCACGAGGTGAATGTCTATGGATGGCAGTACTTCATCCTTGCATGCATCGTCTAATCAAC
CCATATGGTGATGATATTATGGACATCTCACCGAATTTCTCAATAGGCATTTTGGACAAT
AAAATTACATTGTTATTTGAGGTTAATCCAGAGTGTAAAGACACA

>Cucsa.091460
ATGGGTTCTTCTGTTGTTGGAGATGAATCATTTTCTTCTTCTCCCAATTTCAATTACGATTATGATGTGTTTTTTAGTTT
CAGAGGAGAAGATACTCGCTCCAATTTTATCAGTCATCTTCATATGGCCTTGCGTCTAAAGGAAGTCAACGTTTTCATAG
ACGACAAACTCAAAAGGGGTGAACAAATTTATGAGTCTCTTCTCAAATTTATAGAGCGATCTAGACTTTCCCTCGTTATT
TTCTCTAAAGATTATGCATCTTCAACTTGGTGTTTGGATGAACTGGTGAAAATAATTGAGTGTAAGAAATCCAAAGGACA
AGCAGTTTGGCCAGTGTTCTACAAGGTGGATCCATCCGAGGTTCGAAAACAAACCGGTGGGTTTGGGGAAGCATTGGCCA
AACATGAAGCTAATAAGTTATTGGCCAACAAGATTCAACCATGGAGGGAAGCTTTGACTTTTGCTGCTGGTTTGTCTGGT
TGGGATCTAGCAAATAGCAAGGATGAGGCTGAACTTATCCAAAAAATTGTTAAACGAGTGTTGTCTGCAGTAAATCCAAT
GCAATTACTACATGTAGCCAAGCACCAAGTAGGAGTTGATTCTCGACTAAGGAAAATTGAGGAGTTGGTCTCTCATATTG
GGTCCGAGGGTGTTAATCTGGTGGGGTTGTATGGCATTGGAGGCATTGGTAAGACCACTTTGGCTAAGGCTTTGTACAAC
AAAATTGCTACCCAATTTGAAGGATGCTGCTTTCTACAAGATGTTAGACGAGAAGCTTCGAAGCATGGGCTCGTTCAACT
ACAGGAAACCTTACTCAATGAGATCTTAAAAGAGGATTTGAAGGTTATTGTCAGTCGTGATAGAGGAATTAACATCATAA
GAAGTAGACTGTGTTCAAAGAAAGTTCTTATAGTTCTTGATGATGTGAATGATCTTGAGCAATTAGAAGCACTGGTTGGT
GGGCGTGATTGGTTTGGTCAAGGTAGTAAAATCATTGTGACGACAAGGAATGAACATTTACTTTCTAGCCATGGATTTGA
TGAAAAGCATAAAATTCAAGAATTGAATCAAGACCATGCTCTTGAGCTTTTCAGTTGGCATGCTTTTAAGAAAAGTCATC
CATCAAGTAATTATTTAGATTTTTCAAAGCGTGCTACAAGTTATTGTAAAGGTCTCTCTTTGGCCCTCGTTGTTTTGGGT
TCTTTCCTTTGCGGCAGAGCTAAAGAAGAATGGAATGGTATACTAGATGAATTTGAAAACTCTTTGAGAAAAGATATTAA
AGATGTACTTCAATTAAGTTTTGATGGACTTGAAGACAAAATAAAGGATATTTTCCTCGACATTTCTTGTTTATTCGTGG
GAGAAGAATACAAGTGTGCTAAAAAAATGTTGAGTGCATGTCATTTGAACATAGATTTTGGAATTATGATACTCATGGAT
CTTTCACTTATTACGGTTGAAATGGATAGAGTGCAAATGCATGAGTTAATACAACAAATGGGTCGTAGCATAGTTCATAA

TGAATCATCTGAGCCTGGAAAGAGGAGTAGGCTGTGGTTGGAACACGACATTTGGGAGGTGTTTGTTAATAATTCTGGAA
CAGATGCAGTTAAAGCCATAAAGTTGGACTTGCCTAAATCCACAAGGCTAAATGTAGATCCACGGGCATTTGGAAGCATG
AAAAATTTGAGATTGCTTATCATTCGAAATGCACGATTTTGTACAAAGATTAGGTACTTACCTAATAGCTTAAAGTGGAT
TGAATGGCATGGATTTGCTCATCGAACTTTGCCGTCCTGCTTCATTACCAAAAATCTTGTTGGACTTGATTTGCAACATA
GCCTCATCAAAAGATTTGGGAAAAGACTTAAGGTCTCTTCAAGAAATTCCGAACCTTCCTCAAAACATACAAATTTTGAA
TGCGAATGGCTGTAA

>Cucsa.091470
ATGGATTCTTCCACTGTTGCAACAGAATCACCGACTTTCAAATGGACTTATGATGTGTTTTTGAGTTTCAGAGGAGAGGA
TACTCGCACAAATTTCACCAGTCATCTTGATATGGCCTTGCGTCAAAAGGGTGTCAATGTCTTCATAGACAACAAGCTCG
AAAGGGGTGAGCAAATTTCTGAATCCCTTTTCAAATCTATACAGGAAGCTTCCATTTCTATTGTTATATTCTCTCAAAAT
TATGCATCTTCTTCCTGGTGTCTGGATGAATTGGTGAACATAATTGAGTGTAAGAAATCCAAGGGCCAGAATGTTTTCCC
AGTTTTCTATAAGGTGGATCCGTCCGATATACGAAAACAAACTGGTAGCTTCGGAGAAGCACTGGCCAAACATCAGCCTA
AGTTCCAAACAAAGACCCAAATTTGGAGGGAAGCTTTAACTACTGCTGCTAACTTGTCTGGTTGGAATCTAGGAACTAGG
AAGGAGGCTGATCTTATTGGAGATCTTGTTAAAAAAGTGTTGTCTGTATTAAACCGCACTTGCACGCCCTTATATGTAGC
TAAGTATCCGGTTGGAATTGATTCTAAACTAGAATATATGAAGCTTCGTTCACATAATCTTTTTGAGAAGAGCAACAAAT
TCCATTATCGAAAACAACATGAGTATGAGTCTGATACTGGTGTTTACATGGTGGGCTTATATGGCATTGGAGGCATTGGT
AAGACAACTTTGGCTAAAGCTTTATACAACAAAATTGCTAGCCAATTTGAAGCTTGCTGCTTTCTATCAAATGTTAGAGA
AGCTTCAAAGCAATTCAATGGCCTTGCTCAACTACAGGAAACCCTACTCTATGAGATCCTAACGGTTGATTTGAAGGTTA
TCAACCTTGATAGAGGAATTAACATCATAAGGAATAGATTGTGTTTGAAGAAAGTCCTTATAGTTCTTGATGATGTAGAT
AAGCTTGAGCAGTTAGAAGCATTGGTTGGCGGGCGTGATTGGTTTGGCCAAGGCAGTAGAATCATTGTGACGACAAGGAA
CAAACATTTACTTTCTAGCCATGGCTTTGATGAAATGGAAAATATTCTAGGATTGGATGAAGACGAAGCTATTGAGCTTT
TTAGTTGGCATGCTTTCAAGAAAAATCATCCATCAAGTAATTATTTAGACCTTTCAAAACGTGCTACAAGTTATTGTAAA
GGCCATTCTTTGGCTCTCGTTGTTTTGGGTTCTTTCCTCTGTACCAGAGATCAAGTAGAATGGTGTAGTATATTAGATGA
ATTTGAAAACTCTTTGAACAAAGATATCAAAGATATCCTTCAATTAAGTTTTGATGGTCTGGAAGACAAAGTAAAGGATA
TCTTTCTTGATATTTCTTGTTTACTTGTGGGAGAGAAAGTTAAGTACGTTAAGAATATGTTGAGTGCATGTCATGTAAAT
CTAGATTTTGGAATTATAGTACTCACGGATCTTTCATTTATTACGATTGAAAATGACATAATGCAAATGCATGATTTAAT
AAAACAGATGGGTCATAAAATAGTTTGTGGTGAATCTCTTGAGCTTGGAAAGAGGAGTAGGTTGTGGTTGGTACAGGATG
TTTGGGAGGTGCTTGTTAATAATTCAGGAACAGACGCAGTTAAAGGCATAAAGTTGGACTTTCCTAATTCCACGAGGCTG
GATGTGGATCCACAAGCTTTTAGAAAAATGAAAAATTTGAGATTGCTTATTGTTCAAAATGCAAGATTTTCTACAAAGAT
TGAGTACCTACCTGATAGCTTAAAGTGGATTAAGTGGCATGGATTTCGTCAACCAACTTTTCCTTCGTTCTTCACTATGA
AAAATCTTGTTGGACTAGATTTGCAACATAGCTTCATCAAAACATTTGGGAAAAGACTTGAGGATTGTGAAAGGTTGAAG
TATGTTGATCTTAGCTACTCTACTTTCTTAGAGAAAATTCCTAATTTCTCTGCAGCATCAAACCTTGAAGAGTTGTATCT
CACCAATTGCACAAATTTAGGAATGATAGATAAGTCTGTTTTCTCTCTCGATAAGCTTACTGTCCTAAACCTTGATGGTT
GTTCTAACCTTAAAAAGCTTCCAAGAGGCTACTTCATGTTAAGTTCTCTTAAAAAATTGAATCTCTCTTACTGCAAAAAA
CTTGAGAAAATTCCAGACTTATCTTCAGCATCAAACCTTACGAGCTTGCATATCTACGAATGCACAAATTTAAGAGTAAT
TCATGAATCTGTTGGATCTTTGGATAAGCTTGAAGGTTTGTACCTTAAACAATGCACTAACCTGGTAAAGCTTCCAAGCT
ATCTCAGCTTAAAGTCTCTTCTATGTTTATCACTTTCTGGGTGTTGTAAGCTTGAAAGCTTCCCAACAATTGCTAAAAAC
ATGAAATCTTTAAGGACCTTGGATTTGGATTTTACTGCCATAAAGGAGTTACCTTCATCAATTAGATATCTCACTGAGCT
TTGGACATTAAAACTTAATGGTTGCACAAACCTCATCTCCCTTCCCAATACAATTTATTTGTTAAGGAGTCTTGAGAATC
TTCTTCTTAGTGGCTGTTCTATATTTGGAATTTTACCCTCATGTCTCCACAAGTTCATGTCGTTGTGGAATCTTGAATTA
AGGAATTGTAAGTTTCTTCAAGAAATTCCAAGCCTTCCTGAGAGTATACAAAAAATGGATGCCTGTGGTTGTGAATCATT
GTCTCGAATTCCAGATAACATTGTGGATATAATATCAAAAAAACAGGACCTCACAATGGGTGAGATTTCAAGAGAGTTTT
TATTAACGGGGATTGAGATTCCAGAATGGTTCAGCTATAAGACTACATCCAATTTGGTGAGTGCTAGCTTTCGTCACTAT
CCAGACATGGAAAGAACTTTGGCTGCCTGTGTTAGTTTCAAAGTAAATGGAAATTCATCTGAAAGAGGTGCCCGGATTTC
ATGCAATATATTCGTCTGCAATAGACTCTATTTTTCATTGTCAAGACCATTTCTTCCCTCAAAATCAGAATATATGTGGT
TAGTAACAACTTCTCTAGCGTTGGGTTCCATGGAGGTGAATGACTGGAATAAAGTTTTGGTCTGGTTTGAGGTTCATGAA
GCACATAGTGAGGTTAATGCAACTATAACAAGGTATGGTGTCCATGTCACTGAAGAGCTCCATGCGATACAAACGGATGT
CAAGTGGCCGATGGTAAATTATGCTGATTTTTATCAACTGGAGAAATTGCAAAGTCTGGATATTGAGGAACTTCTTCTCA
AACGCCTTTTTGAAGAAATGTCGTGCTGGTCCAATTCCCAAGCAATGTTATATGCGGCAAATTATGATCCAGAAGCAATA
ATCGATTCGAATATACAACCTATGATATTTCCATTGCACGTAACATATAATGGTGAGACATTTATATGTGGAATGGAAGG
CATGGGAGACACTACACTCGCCAACTCTTTATGCAATAAATTTAATAGGTCAAATGACAACGGTTGGCCAAGAGAAGCTT
TAGATGATTCTACAAGCTTTTTGCATTTTCGAGGAGGAAAGTTTTATGGAGGTTCCTGGTCATTGTCCCACCACCGTAAG
CGTGGAGATGGTGAAAGAGGAACCAATATCACAACCCGCACAATATCCTCCAAACGCTATTTGATACTCTTTCATAAAGC
GGGGAGCTATAATCATTTATTTAACTTTGCTGGTAGCCACCGTTTGATTGCAGGTTCTGGCAGTTATGACAGTCTTAACG
GAAGAGGTGATGTTCGGCTTCTGATTGAAAGGGTTGATACATCCTTGCTCTGA

>Cucsa.091530
CAGAATCATCTACTTTCAAGGAGCTATGATGTGTTTTTGAGTTTCAGAGGAGAGGATACTCGAGACAATTTCACCAGTCA
TCTTGACATGGCCTTGCGTCAAAAGGGTGTCAACGTCTTCATTGACGACCAGCTCGAAAGGGGTGAGCAAATTTCTGAAA
CCCTTTTCAAATCTATACACAAAACTTCCATTTCTATTGTTATATTCTCTGAAAATTATGCATCTTCGACATGGTGTCTG
GATGAATTGGTGGAAATAATTGAATGTAAGAAATCCAAGGGTCAGGAAGTTTTGCCGATTTTCTACAAGGTGGATCCTTC
GGATGTACGAAAACAAACTGGTTGGTTTGGAGGAGCATTGGCCAAACATGAGGCTAATTTCATGGAGAAGATTCCAATAT

GGAGGGATGCTTTAACTACTGCTGCCAACTTAGCTGGTTGGGATCTCGGAACCATAAGAAAGGAGGCTGATCTTATTCAA
GTTATTGTTGAACGAGTGTTGTCTATATTAAATCAAACCCACACGCCCTTAAAGGTAGCTGAGTATCCAGTTGGAATTGA
TTACAAAATAGAATCCCTTTACTGGACACAAGAAATGTACAAGTCTGAATGTGTTGACATGGTGGGGATATATGGCATTC
GAGGCATTGGTAAAACAACTTTGGCTAAAGCTTTATACAACAAAATTGCTAGCCAATTTGAAGGCTGCTGCTTTCTATCA
AATGACAATAATTCTAGTGCCTTTAGGGAGTTCGTTCTAATGAATAGTGGGATTCCAGAATGGTTGAGCTATCAGACTAC
ATCAGATTCAATAAGGGTTAGTTTTCAACACAATCGCAATACAGAAAGAACTTTGGCTACATCTGTTACTTTTCGAGTGG
ATGGGGATTCATATCAAGGAATGGCCTTAGTTTCATGTAACATATTCATCGGCTGTAGACTCAAAAGTTGTTATATGAGA
AAATTTCCAACATCAACATCAGAATATACATGGTTAGTAGAAACTTTTTCTGCAACATCTAGTAGCTCCTTGGAAATGAA
TGATTGGAATGATGTTGTAATCTGTTTTGAGGCTGTGAACTGTCCTGCGGTCGTAACTATAAGAAGCTATGGTGTCTATT
TCACTGAAAAGGTCTCTGGGATACAAAATGATATCAAGGGACCAGGGGCAACTTATACAGATTTTGATCAACCGGACGAA
TTGCGCTCGAGCCGGTGA

>Cucsa.091680
ATGAATCAAGCAGGTGGATCATCTTCTTCCTCGTGTTTTAGATGGAGTTTCGATGTATTC
TTAAGTTTTCGAGGGGAAGATACTCGATCCAACTTCACCAGTCATCTTAATATGACTTTG
CGTCAAAGAGGAATCAATGTCTTTATAGATAAAAAGCTTTCAAGAGGTGAAGAAATTTGT
GCATCTCTTTTGGAAGCTATTGAAGGGTCCAAGATCTCCATCGTTGTAATTTCTGAAAGT
TATGCATCTTCGAGTTGGTGTTTGAATGAATTGGTGAAAATCATTATGTGTAACAAGTTG
AGAGGACAAGTTGTTTTACCAATTTTCTACAAGGTGGATCCATCTGAAGTAGGAAAACAA
AGTGGAAGATTTGGAGAAGAATTTGCCAAACTTGAAGTTAGATTCTTCAACAAGATGCAA
GCATGGAAGGAGGCTCTCATTACTGTTTCCCATATGTCTGGATGGCCGGTTCTCCAAAGA
GACGACGAGGCTAATTTGATTCAGAACATTGTTCAAGAAGTCTGGAAGAAATTAGATCGT
GCAACGATGCAGTTGGATGTAGCTAAATATCCAGTTGGAATTGACATACAAGTTAGAAAT
TTACTCCCACATGTTATGTCTAATGGAATTACCATGTTTGGATTATATGGAGTTGGAGGT
ATGGGCAAGACAACTATAGCGAAAGCTTTATACAATAAAATTGCTGATGAGTTTGAAGGT
TGTTGCTTTCTGTCAAATATTAGAGAAGCTTCGAATCAATATGGAGGCCTTGTTCAATTT
CAAAAGGAGCTACTTTGTGAGATTCTAATGGATGATTCGATTAAAGTTAGCAATCTTCCT
AGAGGAATTACTATCATAAGGAATCGACTATACTCAAAAAAGATTCTTTTGATTCTTGAT
GATGTTGATACGCGTGAACAACTACAGGCATTGGCGGGAGGACATGATTGGTTTGGACAT
GGAAGTAAGGTGATTGCGACAACAAGAAACAAGCAATTACTTGTTACTCATGGATTTGAT
AAAATGCAAAATGTTGGGGGATTGGATTACGATGAAGCTCTTGAGCTCTTTAGTTGGCAT
TGTTTTAGGAATAGTCATCCCTTAAATGTTTATTTAGAACTTTCAAAACGTGCCGTAGAT
TATTGTAAAGGCCTTCCCTTAGCTCTTGAAGTTTTAGGTTCCTTCCTTCATTCTATTGGT
GATCCCTCCAATTTTAAACGTATATTGGATGAATATGAAAAACATTACCTCGATAAGGAC
ATCCAAGACTCTCTTCGAATAAGTTACGATGGACTCGAAGATGAAGGAATAACTAAACTT
ATGAATCTATCACTTCTTACCATTGGTAGATTCAACAGAGTTGAAATGCATAACATAATA
CAACAAATGGGTCGGACAATTCATCTTTCAGAAACTTCTAAATCTCACAAAAGAAAAAGA
TTGTTGATTAAAGATGATGCTATGGATGTTTTAAATGGGAATAAGGAAGCAAGAGCAGTG
AAAGTCATAAAACTAAATTTTCCTAAACCTACGAAGTTGGACATCGATTCAAGAGCTTTT
GATAAAGTGAAAAAATTTGGTAGTACTGGAAGTTGGCAATGCCACATCTTCAGAAAGTTCT
ACTCTTGAGTATCTACCCAGTAGCTTAAGGTGGATGAATTGGCCTCAATTTCCTTTTTCA
TCTTTGCCTACAACCTACACAATGGAGAACCTTATTGAATTGAAATTGCCATATAGCTCC
ATCAAACATTTTGGTCAAGGATATATGAGTTGTGAAAGGTTGAAGGAAATTAATCTTAGT
GACTCCAATCTTTTGGTGGAAATCCCAGATTTATCTACTGCAATAAACCTCAAATACTTG
AATCTTGTAGGATGTGAAAATTTAGTAAAAGTTCATGAATCAATTGGATCTCTCAGTAAG
CTTGTCGCCCTTCATTTTTCTAGTAGTGTTAAGGGCTTTGAGCAGTTTCCATCCTGCCTC
AAGTTGAAATCTCTTAAGTTTTTGTCAATGAAAAATTGTAGAATAGATGAATGGTGTCCC
CAATTCAGTGAAGAAATGAAGTCTATAGAATATTTATCGATTGGGTACAGTACTGTAACA
TATCAGCTATCTCCAACAATTGGATATCTTACTAGCCTAAAACATTTGAGCCTCTATTAT
TGCAAAGAGCTCACAACTCTTCCAAAAATTTCAAAGGTTCCCGAAGGCGTAATTTGTATG
AGTGCTGCTGGGAGCATATCATTGGCTAGATTTCCTAACAACTTGGCTGATTTCATGTCT
TGTGATGATTCTGTGGAATATTGTAAGGGTGGAGAATTGAAACAACTGGTATTAATGAAT
TGTCATATTCCAGATTGGTATAGGTACAAGAGCATGAGCGATTCATTAACATTTTTTTTG
CCAGCTGATTATCTAAGTTGGAAATGGAAGCCTTTGTTTGCTCCTTGTGTCAAATTTGAA
GTTACGAATGATGATTGGTTCCAGAAGCTTGAATGTAAAGTTTTTATCAACGATATTCAA
GTATGGAGTTCTGAAGAGGTGTATGCCAATCAGAAGGAACGGAGTGGGATGTTTGGAAAA
GTATCACCAGGTGAGTATATGTGGCTGATAGTACTTGATCCTCATACACGTTTCCAATCA
TATTCGGATGATATCATGGACAGGAGGTCACTGAAGATTATTGATCTAAATCAACTAAGT
TCTGAGATTAATTCCTCACAAAGTATTTTGGGTAAAATTACGGTGTCATTTGAGGTTACT
CCATGGTATAAAGACGTAGTTATAAAAATGTGTGGTGTTCATGTCATCATGGGGGAATGA

>Cucsa.091690
ATGAATCGAGCAACTGGATCGTCTTCCTCGCATCTTAGGTTGCCTTTTGATGTATTCTTA

AGTTTTAGAGGGGAAGATACTCGTTCCAACTTCACGAGTCATCTTCATATGGCTTTGTGT
CAAAAGGGTATCAATGTTTTCATAGATGATGACAAGCTTCCAAGGGGTGAAGAAATTTGT
ACATCTCTTTTGAAAGCCATCGAAGAATCGAAAATCTCGATTGTTATAATTTCAGAAAAT
TATGCATCTTCCCATTGGTGTTTGGATGAACTGATGAAAATCATTATGTGTAACAAATCC
AATAATCGACAAGTCGTTTTTCCTGTTTTTTACAAAGTGGATCCATCTCATGTACGACGA
CAAAGGGGAGTATTTGGAGAAGAATTTGCCAAACTCCAAGTTAGGTTCTCCAACAAGATG
CAAGCATGGAGTGAGGCTCTAACTTTCATCTCCACTATGTCTGGATGGGATCTAAAAAAC
TATGAAAATGAAGCAAGTTTGATTCAAATAATTGTTCAAGAAGTCAGAAAGAAATTAAAG
AATAGTGCAACAACGGAGTTAGATGTAGCTAAATATCCAGTTGGAATTGACATACAAGTT
AGTAATTTACTGCCACATGTTATGTCTAATGAAATTACTATGGTTGGATTGTATGGAATT
GGAGGTATGGGCAAGACAACTTTGGCCAAAGCTTTATACAATAAAATTTCTGATGACTTT
GAAGGTTGTTGCTTTTTGGCAAATGTTAGAGAAGCTTCAAATCAATACTGGGGTCTTGTT
GAACTCCAAAAGACGCTAATTCGTGAGATTCTGATGGATGATTCAATCAAAGTTAGCAAT
GTTGGTATAGGAATTAGCATCATAAGGGATCGATTATGCTCAAAAAAGATAATTTTGATT
CTTGATGACATTGATACACATGAACAACTACAGGCATTGGCTGGAGGACATGATTGGTTT
GGACATGGAAGTAAGGTCATTGCAACAACAAGAAACAAGCAATTACTTGCTAGTCATGGG
TTTAATATATTGAAAAGAGTTAACGGATTAAATGCGATTGAAGGTCTTGAGCTTTTTAGT
TGGCATGCATTTAAAAATAGTCATCCCTCAAGTGATTATTTAGATGTTTCAAAACGTGCT
GTGCATTATTGTAAAGGTCTTCCCTTAGCACTTGAAGTGTTAGGTTCCTTCCTTAATTCT
ATCGATGATCAATCCAAGTTTGAACGTATATTGGATGAATATGAGAACTCATACCTGGAC
AAAGGCATCCAAGATATTCTTCGAATAAGTTATGATGAGCTTGAACAAGATGTAAAAGAA
ATTTTCCTTTACATTTCTTGTTGCTTTGTACATGAAGACAAAAACGAGGTTCAAATGATG
TTAAAAGAATGTGACTCTCGTTTCAGATTGGAAATGGGTATTAAAAAACTCACTGATCTA
TCACTTCTTACAATTGATAAATTCAACCGGGTCGAAATGCATGACTTGATACAACAAATG
GGTCACACAATTCATCTCTTGGAGACTTCTAATTCTCATAAAAGAAAAAGATTGTTGTTT
GAAAAAGACGTCATGGATGTCTTAAATGGAGATATGGAAGCAAGGGCTGTGAAAGTCATA
AAGCTAAATTTTCATCAGCCCACTGAGCTAGACATTGATTCAAGAGGTTTTGAAAAAGTG
AAAAACTTGGTAGTGCTCAAAGTTCACAACGTCACATCTTCAAAAAGTCTCGAGTATCTA
CCGAGTAGCTTAAGGTGGATGATTTGGCCTAAATTTCCGTTTTCATCTTTGCCTTCAACC
TACTCACTGGAGAAACTTACTGAACTCAGCATGCCAAGTAGCTTCATCAAACATTTTGGA
AATGGATATCTGAATTGCAAATGGTTGAAGCGTATAAATCTTAACTACTCAAAGTTTTTA
GAAGAAATTTCTGATCTATCCAGTGCGATAAATCTTGAAGAGTTGAATCTTTCCGAGTGT
AAAAAGCTGGTAAGAGTTCATGAATCAGTTGGATCACTAGGTAAACTTGCTAAATTGGAA
CTTTCTAGTCATCCTAATGGCTTTACGCAATTTCCATCCAACCTCAAGTTGAAGTCCCTA
CAAAAATTGGTAATGTACGAGTGCAGGATTGTTGAAAGTTATCCTCATTTCAGTGAAGAA
ATGAAGTCTAGTTTAAAAGAATTACGGATTCAGTCTTGTAGTGTGACAAAACTATCCCCA
ACGATTGGAAATCTTACTGGTCTTCAACATTTGTGGATCGATGTATGCAAAGAGCTCACT
ACTCTTCCAAAAATTTTAAAGGTTCCAGAAGGCGTAATTTATATGAATGCTCAAGGGTGC
AGATCATTGGCTAGATTTCCAGACAATATAGCTGAATTCATATCTTGTGATTCGGAATAT
GTAGATGGAAAATACAAACAACTCATATTAATGAATAATTGTGATATTCCAGAATGGTTT
CATTTCAAGAGTACGAACAATTCAATAACGTTTCCTACGACATTTAATTATCCGGGTTGG
AAATTGAAAGTTCTTGCTGCTTGTGTTAAAGTTCAAGTTCATGATCCTGTTAATGGGTAT
CATAGAGGGGGGGATCTTGAATGTGAAGTGTTCTTTAAGGACATTCTAGTATGGAGTTCT
GGAGACTGGACAAATTATCTTGGATACGATTCAAGATGGTTGCCCCTAGGAGCATCACCA
AGTGAGTATACATGGTTTATTGTACTCAATCCTCATAGAGATTTCTCCCTAGATGATTGG
GATGATATGATGGAGAGATCACCAGAGACTGATCTAAGTCAGCTATGTTTTGGAATTAAT
TCCATGGAAATGGACCGTAATAGATCAAATGATAAATGGAATTCTATTGGGGGAAGTATT
TGGAAGAACTTTACGGTGTTGTTTGAGCCTCGTCCCCTGTCTCCAGACACTACAATAAGT
ATAAAAGGTTGTGGTGTTCATGTCATCATGGAGTAA

>Cucsa.091710
ATGGATAGAGCAAGTGGATCATCTTCTTCCTCATGTTTTAGATGGACTTTTGATGTCTTTTTAAGCTTTCGAGGGGAAGA
TACTCGATCCAACTTCACCAGTCATCTTAATATGGCTTTGCGTCAAAGAGGAATCAATGTTTTTATAGATAACAGGATTT
CAAGGGGTCAAGAAATTTCTGCATCTCTTTTCGAAGCTATTGAAGAATCCAAGATCTCCATCGTTATTATCTCCCAAAAT
TATGCATCTTCCAGTTGGTGTTTGAATGAATTGGTGAAAATCATTATGTGTAAGGAATTGAGAGGACAAGTTGTTTTACC
AATTTTTTACAAAGTGAATCCATCTCAAGTACGGAAACAAAATGGAGCATTTGGAGAAGCATTTGCCGAACTTGAAGTTA
GATTCTTTGACAAGATGCAAGCATGGGGAGAGGCTTTGACTGCTGTTTCCCATATGTCTGGATGGGTGAGACAATTTGAG
AATCTGCTCTCTCATGTTATGATTGATGGAACTAGAATGGTTGGATTGCATGGAATTGGAGGTATGGGCAAGACAACTTT
GGCCAAAACATTATACAATCGGATTGCTGATGACTTTGAAGGCTGTTGTTTTTTAGCAAATATTAGAGAAGCTTCAAAGC
AACACGAGGGACTTGTTCGACTCCAAGAGAAACTACTTTATGAGATTTTAATGGATGACTTTATTAGAGTTAGTGATCTT
TACAAAGGAATCAACATCATAAGGAATCGACTATGCTCCAAAAAGATTCTCTTAATTCTTGATGATATAGATACCAGTGA
ACAACTACAGGTATTAGCTGGAGGATACGATTGGTTTGGATATGGAAGTAAGGTCATTGTGACAACAAGAAACGAACACT
TACTTGATATCCATGGATTTAATAAATTGCGAAGTGTTCCTGAATTGAATTATGGTGAAGCTCTTGAGCTTTTTAGCTGG

CATGCTTTTCAGTGTAGTAGTCCACCAACCGAGTATTTACAACTTTCAAAAGATGCTGTAAATTATTGTAAAAATCTTCC
CTTGGCGCTTGAAGTTTTAGGTTCATTCCTTTATTCAACTGATCAATCCAAATTTAAAGGTATATTGGAGGAATTTGCAA
TCTCCAACCTTGACAAAGACATCCAAAATCTTCTTCAAGTAAGTTACGATGAACTTGAAGGTGATGTACAAGAAATGTTC
TTGTTTATTTCTTGTTTCTTTGTGGGAGAAGATAAAACCATGGTTGAAACGATGTTGAAGAGTTGTGGTTGTTTATGTTG
GGAAAAGGGAATTCAAAAACTCATGAATCTATCACTTCTTACTATTAACCAATGGAACAAAGTTGAAATGCATGACTTAA
TACAACAATTGGGTCACACAATCGCACGTTCAAAGACTTCTATATCTCCTTCAGAAAAAAAATTATTGGTTGGAGATGAT
GCTATGCATGTGCTAGATGGCATTAAGGATGCAAGAGCAGTTAAAGCCATAAAGTTAGAATTTCCTAAACCGACAAAGTT
GGACATTATTGATTCAACGGCTTTTAGAAAAGTAAAGAACCTTGTAGTACTCAAAGTGAAGAATGTCATATCTCCAAAAA
TTAGTACTCTTGATTTTCTACCTAATAGCTTAAGGTGGATGAGTTGGTCTGAATTTCCTTTTTCATCATTTCCTTCAAGC
TACTCAATGGAGAACCTTATTCAACTCAAATTGCCACATAGCGCCATCCAACATTTTGGAAGAGCATTTATGCATTGTGA
AAGGTTGAAGCAACTTGATCTTAGCAACTCCTTCTTTTTGGAGGAAATTCCTGATTTATCTGCGGCAATAAACCTCGAAA
ATTTGTCTCTTTCTGGATGTATAAGTTTAGTAAAGGTTCATAAATCAGTTGGATCTCTTCCTAAACTTATTGATTTGAGT
CTTTCAAGCCATGTTTATGGCTTTAAGCAGTTTCCTTCACCACTCAGGTTGAAATCCCTTAAAAGATTTTCAACTGATCA
TTGTACAATACTTCAAGGCTATCCTCAATTCAGCCAAGAAATGAAGTCTAGTCTAGAAGATTTGTGGTTTCAAAGTAGTT
CTATAACAAAGCTATCTTCAACAATTAGATATCTTACCAGCCTCAAAGATTTGACCATCGTGGATTGCAAAAAGCTCACT
ACTCTTCCAAGTACAATTTATGACTTGAGCAAACTTACATCCATAGAAGTCTCACAATCCGATCTTTCAACATTTCCTTC
CTCATATTCCTGCCCTTCCTCACTTCCCCTCCTAACAAGATTACACCTTTATGAGAACAAGATAACAAATTTAGATTTTT
TGGAAACTATCGCTCATGCTGCTCCATCACTGAGAGAGTTGAACTTGTCTAACAACAACTTTTCTATACTACCTTCATGT
ATTGTTAATTTTAAATCCTTGAGATTTCTTGAAACATTTGATTGTAAGTTTCTGGAAGAAATTCCAAAGATTCCAGAAGG
CTTAATTTCTTTGGGTGCATATCATTGGCCAAATCTCCCGACAACTTAG

>Cucsa.091780
ATGGGTTCTTCCACTGCTGCAACAGAATCCATGGCTTTTGAATGGAGTTATGATGTTTTT
TTGAGTTTCAGAGGAGAGGATACTCGTACCAATTTCACCAGTCATCTTGATATGGCCTTG
CGTCAAAAGGGGGTCAACGTCTTCATAGACGACAAGCTCGAAAGGGGTGAGCAAATTTCT
GAATCCCTTTTCAAATCTATACAGGAAGCTTCCATTTCTATTGTTATATTCTCTCAAAAT
TATGCATCTTCTTCCTGGTGTCTGGATGAATTGGTGAACATAATTGAGTGTAAGAAATCC
AAGGGCCAGAATGTTTTCCCAGTTTTCTATAAGGTGGATCCGTCGGATATACGAAAACAA
ACTGGTAGCTTCGGAGAAGCAATGGCCAAACATCAGCCTAAGTTCCAAACAAAGACCCAA
ATTTGGAGGAAAGCTTTAACTACTGCTGCTAACTTGTCTGGTTGGGATCTAGGAGCTTAT
AGGAGGGAGGCTGATCTTATTCGGGATCTTGTTAAGGAAGTGTTATCTACAATAAATCGC
ACTCGCACACCCTTATATGTCGCCAAGTATCCAGTTGGAATTGATTCTCAACTAGAATAC
ATGAAGTTTCACTCACATCATCTCAACAAGGGAAACAAATTCCAATATTGGACACAAAAT
GAGTATGAGTCTGATATTGGTGTTTACATGGTGGGGATATATGGCATTGGAGGCCTTGGT
AAGACAACTTTGGCTAAAGCTCTATACAATAAAATAGCTAGCCAATTTGAAGGGTGCTGT
TTTCTATCAAATGTTCGACAAGCTTCAAACCAATTCAATGGCCTTGTTCAACTACAGCAA
AACCTACTCTATGAAATCTTAGAGGATGATTTGAAGTTTGTCAATCTTGATAAAGGAATT
ACCATCATAAGGAATAGACTGCGTTCAAAGAAAGTTTTGATAGTTCTTGATGATGTGGAT
AAGCTCGAACAACTAGAAGCATTGGTTGGTGGACGTGATTGGTTTGGTCAAGGTAGTAAA
ATCATAGTGACGACGAGGAATAGTCATTTACTTTCTAGCCATGGATTTGATGAAATGCAC
AATATTCAAGGATTGAATCAAGACAGAGCTATTGAGCTTTTTAGTTGGCATGCTTTTAAG
GAAAGTCATCCATCAAGTAATTATTTAGACCTTGCCGAACGTGCTACAAGTTATTGTAAA
GGTCATCCTTTGGCTCTTGTTGTTCTGGGTTCTTTCCTTTGTAATAGAGGTCAAACAGAA
TGGAGAAGGGAAAAAATACAATAATTGTGCTAAAAAAATGTTGAGTGCATGCCATTTGAAC
GTAGATTTTGGAATTATGATACTCATGGATCTTTCACTTGTTACGATTGAAAAGGATAGA
GTGCAAATGCACGGATTAATACAACAGATGGGTCATAGCATAGTTCATAATGAATCATTT
GAGTCAGGAAAGAGGAGTAGATTGTGGTCGGAGCGGGACATTTGGAACGTGTTTGTTAAT
AATTCGGGAACAGATGCAATTAAAGCCATAAAGTTAGACTTGCCTAATCCCATAAACGTA
AATGTAGATCCAAAAGCATTCTTTAGAAGCATGAAAAATTTGAGATTGCTTATCATTCGA
AATGCACAAGTTTGTACAAAGATTAAGTACCTACCTAATAGCTTAAAGTGGATTGAGTGG
CAAGGATTTGCTCATCGAACTTTCCCGTCGTGCTTCATTACCAAAAATCTTGTTGGACTT
GATTTGCGACATAGCTTCATCAAAAGATTTGGGAAGAGACTTGAGGATTGTGAAAGGTTG
AAGCATGTTGATCTTAGCTACTCTACTTTATTAGAGAAAATTCCTGATTTATCCGCTGCA
TCAAACCTTGAAGAATTGTATCTCATCAATTGCACAAATTTAGGAATGATAGATAAGTCT
GTTTTCTCTCTCAATAAGCTTACTGTCCTAAACTTTAAAGGTTGTTCTAACCTTAAAAAG
CTTCCAAAAGGCTACTTCATGTTCAGTTCTCTTAAAATATTGAATCTCTCTTACTGCCAA
GAACTTGAGAAAATTCCAGACTTATCTTCAGCATCAAACCTTCAGAGCTTGCTACTCAAC
GGATGCACAAATTTAAGAGTGATTCATGAATCTGTTGGATCTTTGAATGAGCTTGTATTG
TTGGACCTTGGACAATGCACTAACCTTTCAAAGCTTCCGAGCTATCTCAGGTTAAAGTCT
CTTGTCTATTTAGTACTTTTTGGGTGTGGTAAGCTTGAAAGCTTTCCAACAATTGCTGAA
AACATGAAATCTTTAAGGTGCTTGGATTTGCATTCCACCGCCATAAAGGAGTTACCTTCA
TCACTTGGATATCTTACTCAACTCGATAAATTACACCTTACCGGTTGCACAAACCTCATC
TCCCTTCCCAATACAATCTATTTGTTAAGGAATCTTAACGAACTTCATCTTGGTGGGTGT

TCTAGATTTGAAATGTTTCCCCATAAATGGGTCCCAACCATCCAACCAGTATGCTCTCCT
TCAAAAATGATGGAAGCAGCTTCGTGGAGCTTAGAGTTTCCCCATTTAGTAGTACCAAAT
GAAAGCATATGTTCCCATTTCACTTTGTTGGATCTTAAATCTTGCAACATATCAAATGCA
AAATTTTTGGAAATTTTATGTGATGTTGCCCCTTTCTTATCTGATCTACGTTTGTCCGAA
AACAAATTCTCTAGTTTACCCTCATGTCTCCACAAGTTCATGTCCTTATCGAATCTTGAA
TTAAGGAATTGTAAGTTTCTTCAAGAAATCCCAAACCTTCCCCAAAACATACGAAACTTG
GATGCCAGTGGTTGCAAATCGTTGGCTCGAAGTCCAGATAACATTGTGGATATAATATCA
ATAAAACAGGACCTTGAATTGGGTGAGATTTTAAGAGAGTTCTTATTAACGGACATTGAG
ATTCCAGAATGGTTCAGCTATAAGACTGCATCCAATTTGGTGACTGCTAGCCTTCGTCAC
TATCCAGACATGGAAAGAACTTTGGCTGTCGCTGTTAGTTTTAAAGTGAATGGAGATTCA
TCTGAAAGTGAGGCCCAAATTTCATGCAATATATTCATCTACAATAAACTCCGTTGTTTG
TTTTCAAGATCATTTCTTCCATCAAAATCAGAATATATGTGGTTAGTAACAATTTCTCTA
GCGTGTTCCCTGGAGGTGAATGATTGGAATAAAGTTTTCGTCTGGTTTGAGGTTCATGAA
GCACATGGTGTAACTGTAACAAGGTATGGGGTCCATGTCACTGAACAACTCCATGGGATA
CAAACGGATGTCAAGTGGCCGATGGTTAATTATGCTGATTTTTATCAACTGGAGAAATTG
CGAAGGGATCTGGATTTTGAGGATCTCAAAGCTAGTTTAAAGAAGTCTGCTGTTCAAATT
CCAAAGCAACATTGCATGCACTTTAGTATGATCCAGAGGCAATAA

>Cucsa.091820
ATGGGTTCTTCCATTGTTGGAGCTGAATCATCAACTTCTTCTTCTTCTAGTTTCAAGTGGAGTTTTGATGTGTTTTTGAG
TTTTAGGGGAGATGATACTCGTTCTAATTTCACCGGTCATCTTGACATGGCCTTGCGTCAAAAGGGTGTCAATGTCTTCA
TAGACGACATGCTCAAAAGGGGTGAGCAAATTTCTGAAACCCTTTCCAAAGCTATACAGGAAGCTTTGATTTCTATTGTT
ATTTTCTCTCAAAATTATGCATCTTCTTCATGGTGTCTGGATGAATTGGTGAAAATAGTTGAGTGTAAGAAATCCAAGGG
CCAGCTTGTTTTGCCAATTTTCTACAAGGTGGATCCTTCCGATGTACGAAAACAAACTGGTTGCTTTGGAGAAGCATTGG
CCAAACATCAGGCTAATTTCATGGAGAAGACTCAAATATGGAGGGATGCTTTAACTACTGTTGCCAACTTCTCTGGTTGG
GATCTAGGAACTAGGAAGGAGGCTGATTTTATTCAAGACCTTGTTAAAGAAGTATTGTCTAGATTAAATTGTGCCAACGG
GCAGTTATATGTAGCTAAGTATCCAGTTGGAATTGATTCTCAACTAGAAGATATGAAGTTACTCTCGCATCAGATACGAG
ATGCGTTTGATGGCGTTTACATGATGGGGATATACGGCATTGGAGGCATTGGTAAGACTACTTTGGCTAAAGCTTTGTAC
AATAAAATTGCTAACCAATTTGAAGGTTTCTGCTTTCTATCAAATGTTAGAGAAACTTCAAAACAGTTCAATGGACTCGT
TCAACTACAGGAAAAACTACTCTATGAAATCTTAAAGTTTGATTTGAAGATTGGCAATCTTGATGAAGGAATTAACATCA
TAAGAAGTAGATTGCGTTCAAAGAAAGTTCTTATAGTTCTTGATGATGTGGATAAGCTCAAGCAATTGGAAGCATTGGTT
GGTGAACGTGATTGGTTTGGCCATGGTAGTAAAATCATTGTGACAACAAGAAATAGTCATTTACTTTCTAGCCATGAATT
TGATGAAAAGTATGGTGTTCGGGAATTGAGTCATGGTCATTCCCTTGAACTTTTTAGTTGGCATGCTTTTAAGAAAAGTC
ATCCATCAAGTAATTACTTAGACCTTTCAAAACGTGCGACAAATTATTGTAAAGGTCATCCTTTGGCCCTTGTTGTTTTG
GGTTCTTTCCTTTGTACCCGAGACCAAATAAAATGGAGAACTATATTAGATGAATTTGAGAACTCTTTGAGTGAAGACAT
TGAACATATTATTCAAATCAGTTTTGATGGGCTTGAAGAAAAAATAAAGGAGATCTTCCTTGATATTTCTTGTTTGTTTG
TGGGAGAGAAAGATTGTAAAAGGTTGAAGCATGTTGATCTTAGTTACTCTTCTTTATTAGAGAAGATTCCCGACTTCCCT
GCAACATCAAATCTTGAAGAATTATATCTTAACAACTGCACAAATTTAAGAACAATTCCTAAGTCAGTTGTTTCTCTTGG
TAAGCTTCTTACTTTAGACCTTGATCATTGTTCAAACCTTATAAAGCTTCCAAGCTACCTCATGCTGAAGTCTCTTAAAG
TTTTGAAGCTTGCTTACTGCAAAAAACTTGAGAAACTTCCAGACTTCTCTACAGCTTCAAACCTTGAAAAGTTGTACCTC
AAAGAATGCACAAATTTAAGAATGATTCATGATTCTATTGGATCTCTGAGTAAGCTTGTTACCTTGGACCTTGGAAAATG
CTCTAACCTTGAAAAGCTTCCAAGCTACCTCACATTAAAGTCTCTTGAATATTTGAATCTTGCTCATTGCAAAAAGCTTG
AGGAAATTCCCGACTTCTCTTCTGCATTAAACCTTAAAAGCTTATATCTTGAACAATGCACAAATTTAAGAGTAATTCAT
GAGTCTATTGGATCTTTGAATAGTCTTGTTACCTTGGACCTTAGACAATGCACTAACCTTGAAAAGCTTCCAAGCTACCT
CAAGTTGAAGTCTCTTAGACATTTCGAACTCTCTGGCTGCCACAAGCTCGAAATGTTTCCAAAAATTGCTGAAAACATGA
AATCTTTAATTTCATTGCATTTGGATTCTACTGCCATAAGGGAGCTACCTTCATCAATTGGATACCTTACTGCGCTTTTG
GTATTAAACCTTCACGGTTGCACAAATCTCATCTCCCTTCCTAGTACAATTTATTTGTTAAAGAGCCTTAAGCATCTTTA
TCTTGGTGGGTGTTCTAGATTTCAATTGTTTTCCCATTTTTTGGAAATTTTATGTAATGTCGCCCCTTTCTTATCTAGTA
TACTTTTGTCGGAAAACAAATTCTCTAGTCTACCACCATGTCTTCATAAGTTTATGTCCTTGTGGAATCTCCAATTAAGG
AATTGCAAGTTCCTTCAAGAAATTCCTAACCTCCCTCATTGTATACAAAAAATGGATGCCACTGGTTGCACATTGTTGGG
TAGAAGTCCAGACAACATCATGGACATAATATCGAGCAAGCAGGTTCCTCACTTCCATTTCCATTTTCCTTTGGTTGGAG
ATTCATATCAAGGAATGGCCTTAGTTTCATGTAAAATATTCATTGGCTACAGACTCCAAAGTTGTTTTATGAGAAAATTT
CCATCATCAACATCAGAATATACATGGTTAGTAACAACTTCTTCTCCAACATTTAGCACTTCCTTGGAGATGAATGAGTG
GAATCATGTCACAGTCTGGTTTGAGGTTGTGAAATGTTCTGAGGCCACCGTAACTATAAAATGCTGTGGTGTCCATCTCA
CTGAAGAGGTCCATGGAATACAAAATGATGTCAAGGGGCCAGGGGTAGTTTATACAGTTTTCGATCAACTGGACAAATTA
CCGAGCCGGATACGTGGTATGGAAGGCATGGCAGAGACAACACTTGCCAACTCTATATGTAACAAATATGAAAGAAGTCG
GAATCTTTTCTCTGCAAAAAAAGCTTTGAATCATTCTACTGGCTTTCTTTGTGGAGATGGAAATGGGCTTTCTTGGGAAA
TGGTAGACAGACCGATATTAAGTGATAGATTGTCTTCCCAAAAATATCTTAGAATTTTCGACGATCGTGATCGATATGGA
GACCTAAATGATGTGGCTCATGGGACTGGTAATAGGTTTCGTTCAAGATTTTTAAGGATGGATGATATAAAAGAAGATGA
TATCAGAGAAGAGCCTTATTGGAAGTACATGGAAAG

>Cucsa.091840
ATGGGTTCTAACGCTGCTGGAGCGGAATCGTCGTCTTCTTCTCCAATCAATTGGATTTATGATGTGTTTTTGAGTTTTAG

AGGAGAGGATACTCGCTCCAATTTCACAAGTCATCTTCACATGTTCTTGCGTCACAAGGGTGTCAATGTTTTCATAGATG
ACAGGATCGAAAGGGGTGAGCAAATTTCTGAAGCCCTTTTAAAAACTATACAGTGTTCTTTGATTTCTATTGTTATATTC
TCGGAAAATTATGCATCTTCTACATGGTGTCTGGATGAATTGGTGGAAATAATTGAGTGTAAGAAATCCAAGGGTCAGAA
AGTATTGCCAATTTTCTACAAGGTGGATCCTTCGGATGTACGAAAACAAAATGGTTGCTATGGAGAAGGATTGGCCAAAC
ATGAGGCTAATTTCATGGAGAAGATTCCAATATGGAGGAATGCTCTAACGACTGCTGCCAACTTGGCTGGTTGGGATCTC
GGAACAATAAGAAATGAGGCTGACCTTATTCAAGTTATTGTTAAAGAAGTGTCGTCTACATTAAATGTCACCACGCCCTC
AGATAAGCCTCTACTAGTTGGAATTGATTCCAAAATTGAATCCCTTTATTGGCCTACAGAAGAAATGTACAAGTCTGAAT
GTGTTGACATGTTGGGGATATATGGCATTCGAGGCATTGGTAAAACAACTTTGGCTAAAGCTTTATACTACAAAATGGCT
AGCCAATTTGAATGTTGCTGCTTTCTATCAAATGTTAGAGAAGCTTCAAAGCAACTCAATGGCCTCGCTCAACTACAGAA
AAAACTACTTTTTCAGATCTTAAAGTATGATTTGGAGGATGTCGATGATCTTGACAGGAGAAATAATATCATAAAGCATA
GACTCCATTCGAAGAAAGTTCTTATACTTCTTGATGATGTGGATGAGATGAAGCAATTAAAAGCATTGGCTGGTGGGCAT
GATTGGTTTGGTCAGGGTAGTAAAATCATTGTGACGACTAGAGATAAACATTTACTTGATAGCCATGGATTTGGTCAAAC
ATATGAAGTTGAAGGATTGTGGGAACACAATGCATTTGAGCTTTTTTGTTGGCATGCCTTCAAAAAAAGTCATCCATCTA
GTAATTATTTAGACCTTTCAGAACGTGCTACAAGGCATTGTAAAGGTCATCCTTTGGCTCTTGTTGTTTTGGCTTCTTTC
CTTTGTGGCAGAGATCAAGCAGAATGGAGTGGTCTATTAGATGGATTTGAAAACTCTTTGAGAAAAGGTATTAAAGATGT
TCTTCAATTAAGTTTTGATGGGCTGGAAGACGAAGTAAAGAAATTTTTTCTTGATATTTCTTGTTTACTCGTGGGTGAGA
CAGTTACCTATGTTAAGAAAATGTTGAGTGAATTCCATTCGATTCTGGATTTCAAAATTAGCAATCTGAGGCATCTTTCA
CTTATTAGGATGGAAGAATATGATGATGATAGGGTGCAAATGCATGATTTAATAAAACAAATGGGTCATAAAATAGTTTA
TGATGAATGTGGTGATGAGCCTGGAAAAAGGAGTAGATCAGGGAAGCGATGCAGTGAAAGGCATAAAGTTGGTGTTTATC
TGATCCCACCAAGGGTCATAAACGTGAATCCAGAGGCATTTAGAAGCATGAAGAATTTGAGAATACTGATTGTTGATGGA
AATGTGAGGTTTTGCAAAAAAAAAAAGTATGTACCGAATGGGTTAAAGTGGATAAAATGGCATAGATTTCCTCATCAAAC
TTTACCCTCATGCTTTATTACAAAAGATCTGCTTCCAAGCTACCTCAAGTTAAAGTCTCTTACAGATTTAGATCTCTCTG
GTTGTCGTAAGCTCGAAACGTTTCCAGAAATTGATGAAAACATGAAATCCTTAGAAAGGTTGAGGTTGTCTTATACGGCC
ATAAGGAAGCTACCTTCAGTCTTTGCGGGCTTAGTCTTTCTGAGTGTTCTATTGAAATGCGTTCCAAAATTTTTCCATTC
ACGAGTTCCAAAAGAAATCTTATTTTTCGAACATTTGGAGTTGCTGGATCTTAAAGGTTGCAATATATCAAATGTTGATT
TTCTGGAAAATTTATGTAATGTAGCTCTATCCTTAACTAGTATAGTCTTGTCAGAAAACGAATTCTGTAGTCTACCTTCA
TGTCTCCATAAGTTTATGTCCTTGCGGAATCTCCAATTAAGGAATTGCATGTTTCTTCAAGAAATTCCAAACCTCCCTCA
GAGTATACAAATAGTAGATGCCACTGGTTGCATATCGTTGAGAAGAAGTCCAAACATTATGTGGACATAA

>Cucsa.091880
ATGGAAAGTATTCCTATTTCAATAATTGCAAAAATTTGTGAATACACTGTTAAACCTGTTGGACGTCAACTTTGTTATGT
ATGTTTCATTCATTCCAACTTTCAAAAACTCAAGAGTCAAGTAGAAAAGCTGACAGATACAAAAGGATCTGTGGAAGACA
AGGTTTTTATTGCAAGAAGAAATGCAGAAGACATAAAACCTGCAGTTGAGAAATGGTTGGAAAAGGTTGATCGCCTTGTT
AGAAAATCTGAGAAGATACTAGCCCATGAAGGTAGGCATGGTAGATTGTGTTCCACCAATTTGGTCCAAAGACACAAGGC
AAGTAGAAAAGCAAGCAAAATGGCAGATGAGGTTCTTGAGATGAAAAATCAGGGAGAAAGTTTTGATATGGTATCCTTTA
AAGGTCGTATCTCATTGGTTGAGAGTCCACTGCCAAAAGCACCTGACTTTCTTGACTTTGGCTCTAGAAAGTCAACAGTG
GAACAAATCATGGATGCACTCTCTGATGATAATGTCCATAAGATTGGAGTGTACGGGATGGGGGGTGTTGGCAAAACAAT
GCTAGTGAAAGAAATTGTAAGAAAAATTGAGGAGAGTAAGAAGTCTTTTGATAAGGTGGTAACATCCACGATTAGCCAAA
CACCAGATTTTAAAAGGATTCAAGGACAACTAGCTGACAAGATAGGTTTAAAATTCGAACAAGAAACAATAGAAGGAAGG
GCTACTTTTCTACGAAGGTGGTTGAAGGCGGAGAGAAGAATTCCAAGTGTTGAAGATCATAAAGGAATATGCAAGATCTT
GTTTACCTCTAGGAATAAACAATTGATCTCAAATGATATGGGCGCCAATAAAATTTTTGAGATAAAAGTTTTAGGAGAAG
ATGAGTCCTGGAATTTATTTAAGGCAATGGCGGGTGAAATTGTTGAAGCAACTGATTTGAAGCCTATAGCCATTCAAATT
ATGAGAGAATGTGCAGGTTTGCCTATTGCTATTACTACTGTTGCTAAGGCATTACTAAATAAACCTTCCGACATTTGGAA
TGATGCCTTAGATCAACTTAAAAGTGTTGATGTGGGTATGGCAAACATTGGAGAAATGGACAAGAAAGTGTATTTGTCAC
TAAAACTGAGTTACGATTACTTGGGATATGAAGAGGTGAAGTTACTATTCTTGTTATGTAGCATGTTTCCAGAAGATTTT
AACATTGATGTGGAAAAGTTGCACGTATATGCTATGAGCATGGGTTTCTTACGTGGTGTTGATACTGTGGTAAAAGGACG
ACGAAGGATTAAAAAATTGGTTGACGATCTTATATCTTCTTCCTTGCTTCAACAATATTCTGAGTATGGGAACAATTATG
TGAAAATACATGATATGGTTCGTGATGTAGCCATACTAATAGCATCTCAGAATGATCACATACGTACATTGAGCTATGTG
AAAAGATCGAATGAAGAATGGAAAGAAGAGAAATTGTCGGGTAACCATACTGTAGTGTTCTTAATTATTCAAGAATTGGA
TTCACCTGATTTCTCAAAGTTAATGCTACCTAAAGTTCAATTGTTCGTGTTATTTGGACCATCACCATCTATATATAATA
GACATGTTGTGTCAGTGGTAGAAACTTTCTATAAAGAAATGAAGGAGCTCGAAGGTTTGGTAATAGAAAGGGTGAAAATA
TCCTTATCGCCACAAGCTCTTTACTCATTTGCAAACCTTAGATTATTAAGATTACATGACTGTGAATTAGGGAGCATAGA
TATGATTGGTGAATTAAAAAAGCTTGAAATTCTTGATTTTAGTAAATCTAACATCGTTGAAATTCCTATGACCTTTAGCA
AATTGACGCAGTTAAAAGTGTTAAATTTATCTTTTTGTGATGAGCTTGAGGTAATTCCACCCAATATTCTTTCAAAATTG
ACAAAACTGGAAGAATTACATCTAGAAACTTTCGATAGTTGGGAAGGGGAAGAATGGTACGAAGGAAGGAAAAATGCTAG
TCTTTCTGAGCTAAGGTACTTGCCACACCTTTATGCTTTAAATTTAACCATTCAAGATGATGAGATTATGCCAAAACACT
TGTTTTTAGCTGGGGAGTTGAATCTTGAAAATTTTCACATTACTATTGGTTGTCAGAGACAAAAAAGACATATTGATAAT
AAGACCAATTTCTTTAGAATCAAGATGGAATCAGAAAGGTGCTTGGATGATTGGATAAAAACTTTGTTAAAGAGGTCAGA
AGAAGTCCATTTGAAAGGATCAATTTGTTCAAAGGTTCTCCACGATGCAAATGAATTCTTACATTTGAAAGAATCTCTTT
TTAGCAAGTTGAAAAGTGTAGTCGTAACGAAGTGCAATAAATTAGAAAAGCTCTTTTTCAACTGCATATTGGATGACATT
CTGAGTCTTGAGGAGATTGCTATTCATTATTGTGAGAAGATGGAAGTGATGATTGTGATGGAAAACGAGGAGGCAACCAA
CCACATTGAGTTTACTCATTTAAAGTATTTATTTCTAACGTATGTACCACAACTTCAAAAATTTTGCTCCAAAATTGAGA
AATTTGGACAATTAAGTCAGGATAACTCAATCAGCAATACCGTTGACATTGGTATATTTGAAGTGCAAGAGTCAAGTATT

ACAGATACAAGTCTTATTGTGCTCAAAAACTTGAGAGAGTTGAAATTATATAATCTTCCCAACCTTGAGTACGTATGGAG
CAAAAATCCTTGTGAGCTTCTGAGTTTTGTAAATATAAAAGGTTTGGCCATTGATGAATGTCCAAGACTTAGAAGAGAAT
ATTCAGTCAAAATTCTCAAGCAACTTGAAAGACTAACAATGGATATTAAACAATTGATGGAGGTTATTGAGAATCAAAAG
TCAACCGATCATAATATGGTGAAATCAAAGCAATTGGAGACTTCTTCTAAGGATAACTCAACTCATTTGCCAGTGGAAAT
TGTACAAATCTTATACCAACTTGAGCACTTTGAATTGGAAGGAGCGTATATTGAAGAAGTTTTCCCCAGCAATATATTGA
TTCCAATGAAAAAACAGTACTATGCAAGATCTAAGAATTCAGTGCGTAGTTGGTTTCTATCTAAACTACCCAAGCTTAGG
CATTTGTGGAGTGAATGTTCACAAAAGAATGCTTTTCCAATTCTACAAGATTTGAATGTAATAAGAATTTCAGAATGTGG
TGGGTTGAGTTCCTTAGTTTCGTCATCAGTATCCTTTACAAACTTGACAGTTCTTAAAGTGGATAAGTGTGATAGACTAA
CCTATTTGTTGAATCCTTTGGTGGCTACAACCCTTGTGCAACTTGAAGAATTGACTTTAAGAGAATGCAAAATGATGAGT
AGTGTAATTGAGGGAGGATCAGCTGAAGAAGATGGAAATGAGGAGACAACCAACCAGATTGAGTTTACTCATTTGAAGTC
TTTATTTCTAAAGGATTTACCACGACTACAAAAGTTTTACTCTAAAATTGAGACATTTGGTCAATTAAGCCGTGATAATT
CCGAAAACCCTGAAACAACCACAATTCACAATCGCATTGGTGATTCATTTTTCAGTGAACAGGAATCACTTCCTAATTTG
GAGACATTGAGAATTGATGGTGCAGAGAATTTGAGGATGATATGGAGTAATAATGTACTCATTCCTAATTCCTTTTCCAA
ACTCGAGGAAGTAGAGATTTATTCATGCAACAATCTTCAGGACGTATTATTTCATCCAAATATTATAAACATGCTTACAT
GCCTTAATACATTAAGGATTAAAAATTGTGAATTATTGGAGGGGATATTCGAAGTGCAAGAGCCGATTAGTGTTACAAAA
ACAAAAACAAATGCTATCGTGCTACCAAATAATTTGATAGAGTTGGAATTATATAATCTTCCAAACCTTGAGTACCTATG
GAGTAAGAATCCAAATTTTGAACGGCTCGTGACTTTTGAAAGTATAAGAAGTTTGTCCATTGAAAAATGTTCAAAACTCA
AAGGAGAATATTTTTTGTCAATCAAAACTTTCAAGCAACTTGTTGAGGTTCTACAGTTGAGAGATGGTTCTAAGTTGTTT
TCCAATCTTAAAGAATTGAAGCTATATGGTTTTGTTGAGTATAACTCAACTCATTTGCCAATGGAAATTGTTCAAGTCTT
AAACCAACTTGAGAAGTTTGAATTGAAAGGAATGTTCATTGAAGAAATTTTCCCCAGCAACATACTGATTCCAAGCTATA
TGGTTTTACGAAAATTGACTCTATCTAAACTATCCAAGCTTAGGCATTTGTGGGGTGAATGCTCACAAAAGAATAATGAT
TCACTTCTACGAGATCTGACCTTTTTATTCATTTCAAAATGTGGTGGATTGAGTAGTTTAGTGTCATCATCGGTGTCATC
TTTTACAAACTTGAGAATTCTTGAAGTGGAAAAGTGTGATGGACTAAGCCATTTGTTGAGTCCATCGGTAGCTACAACGC
TCGTGCATCTTGAAGAATTGAGAATAGAAGAATGCAAAAGGATGAGTAGTGTAATTGAGGGAGGATCATCTGAAGAAGAT
GGAAATGATGAAATTATTGTATTCAACAACCTACAACATTTAATCATTTCTTCTTGTTCCAACCTAACAAGTTTTCATTG
TGGAAGATGCATCATTCAATTTCCATGTTTGAAACAAGTATACATTAACAAGTGTACTGAATTGAAGGTCTTTTCGCTTG
GAATTGTAAGCACACCTCCTTTAAAATATGAAAATATTTATTTAAAGAATGATGATGATGATGATACGTGGCATCACCCA
AAAGAATCCATAGAAATGGTGGTGGAAACTGATATGAATGTCATCATCAGAGAATATTGGGACGACAACATCGATACTAG
AATTTCAAATTTGTTTGGAGAAGAG

>Cucsa.094560
ATGGCTGAGTTCCTATGGACTTTTGCTGTTCAAGAAGTGTTGAAGAAGGTATTGAAACTT
GCAGCTGACCAAATTGGTTTGGCATGGGGCTTGGACAAGGAGCTTTCAAACCTCTCCCAA
TGGCTACTCAAAGCAGAAGCTATTTTAGGTGAGATTAACAGGAAAAAACTACACCCTAGT
TCTGTGAGACTGTGGGTGGAAGATCTTCAACTTGTTGTTCATGAAGCAGACGATCTATTG
GATGAGCTTGTTTATGAAGATCTTCGTACGAAGGTGGAAAAAGGACCGATTAACAAGGTA
CGTTCTTCTATATCAAGTCTCTCGAATATTTTCATTATCTTTCGCTTCAAAATGGCCAAG
AAAATCAAGGCTATTATTCAAAAGTTGCGTAAATGTTACTCTGAGGCCACTCCTTTAGGA
CTTGTTGGTGAAGAATTCATACAAACAGAGAATGATCTTAGTCAGATTCGAGAGACGATC
TCAAAACTTGACGATTTTGAAGTTGTTGGAAGGGAGTTTGAAGTTTCAAGCATAGTGAAA
CAAGTGGTTGATGCTAGTATTGACAATGTTACATCTATCTTGCCCATTGTGGGTATGGGT
GGAATCGGAAAAACAACTTTGGCAAAGACAATCTTCAATCATGAGGAGATCAAAGGACAT
TTTGATGAAACAATTTGGATATGTGTGTCCGAACCATTTCTTATCAACAAAATTTTGGGA
GCAATTTTACAAATGATAAAGGGTGTTTCCAGTGGCTTGGATAATAGAGAGGCTTTACTT
CGAGAGCTTCAAAAGGTGATGCGAGGTAAAAGATATTTTCTTGTGCTTGATGATGTTTGG
AATGAAAATCTTGCTTTATGGACTGAATTGAAACATTGTTTACTGAGTTTCACTGAAAAA
TCTGGAAACGCTATTATTGTGACTACAAGAAGTTTCGAAGTAGGAAAGATTATGGAGAGT
ACTCTTTCTAGCCATCATTTGGGAAAATTATCTGATGAACAATGTTGGTCTTTGTTTAAA
AAAAGTGCAAATGCAGATGAACTGCCAAAGAATCTAGAGTTGAAGGATCTTCAAGAAGAA
TTGGTGACAAGATATCTCGACATTTCAAATTCAAAGATAGAGGAACTTCCAAATTCTATC
TCTTTGCTTTATAACTTACAAACACTGAAGCTTGGAAGCTCAATGAAAGACCTTCCACAG
AATTTGAGCAAGTTGGTTAGTTTAAGACATCTAAAGTTCTCAATGCCACAAACGCCTCCA
CATTTGGGTCGATTGACTCAACTTCAAACATTGTCTGGTTTTGCAGTTGGATTCGAGAAG
GGTTTCAAAATAGGAGAACTTGGATTTTTGAAAAACCTCAAAGGTAGATTAGAACTTTCA
AATCTTGATCGAATTAAACATAAAGAGGAAGCCATGAGTTCCAAATTGGTAGAAAAGAAC
TTGTGTGAGCTATTCTTGGAATGGGATATGCATATTTTAAGAGAAGGTAACAACTACAAT
GACTTTGAAGTGTTAGAAGGGCTTCAACCACACAAAAATCTTCAATTCCTGAGTATCATA
AACTTTGCTGGCCAACTTCTGCCTCCTGCCATTTTTGTTGAAAATTTAGCTGTGATACAT
CTAAGACATTGTGTAAGATGTGAAATACTTCCAATGCTTGGACAATTACCTAATTTAGAG
GAACTAAATATTTCCTACTTACTTTGTCTAAGAAGTATTGGGTATGAATTCTATGGAAAT
TATTATCATCCCTACAGCCATAAGGTTTTATTTCCCAAGTTGAAGAAATTTGTACTCTCT
CAAATGCCCAATCTAGAGCAATGGGAAGAAGTAGTATTCATATCAAAGAAAGATGCAATT
TTTCCTCTTCTTGAGGACTTAAATATTAGTTTTTGTCCTATATTAACAAGTATTCCAAAT

ATTTTTAGACGTCCTCTTAAAAAGCTACATGTTTATGGATGTCATGAAGTGACAGGATTG
CCAAAAGATCTACAACTTTGCACTTCCATTGAGGATCTAAAGATTGTTGGGTGCCGTAAA
ATGACACTAAATGTGCAAAATATGGATAGCTTGTCTCGTTTCTCTATGAATGGGTTGCAG
AAGTTTCCCCAAGGGCTGGCTAATCTAAAAAACTTGAAAGAAATGACAATCATTGAATGC
TCACAAGATTGTGACTTTAGTCCTCTCATGCAACTTTCTTCACTTGTAAAGCTTCATTTG
GTTATTTTCCCAGGGAGCGTGACTGAGCAACTTCCTCAACAACTTGAGCATCTCATTGCC
TTAAGATCTTTGTACATTAATGATTTTGATGGAATTGAGGTTTTACCAGAATGGTTGGGA
AACCTTACCTCTTTGGAAGTTTTGGGACTTTATTATTGTATAAATTTGAAACAGTTTCCT
TCAAAGAAAGCCATGCAATGTCTCACCCAATTAGTCCACGTGGATGTCCACAACTGCCCG
AGTTCGCAGATTTTGTCCCATGATCTAAAGGCCAAAGCTCATGCCAAAGCAAACTTAGTT
CAATGGTAA

>Cucsa.094580
ATGGCTATCGCTGAGTTCCTATGGACTTATGCTGTCCAACAAGTGTTGAAGAAGGTATTGGAACTTGCGGCTGACCAAAT
TGAAAAAAAACTACACCATAGTTCTGTGAGACTGTGGGTGGCAGATCTTCTACTTGTTGTTCATGAAGCCGACAATCTAT
TGGATGAGCTTGTTTATGAATATCTTCGTACAAAGGTGGAAAAAGGATCGATTAACAAGGTATGTTCTTCGGTGTCAAGT
CTTTCTAATATTTTCATTATCTTTCGCTTCAAAATGGCCAAGAAAATCAAGAGTATTATTGAAAAGTTGCGTAAATGTTA
CTACGAGGCGACTCCTTTAGGACTTGTTGGTGAAGAATTCATAGAAACAGAGAATGATCTTAGTCAGATTCGAGAGACGA
TCTCAAAACTTGATGATTTTGAAGTTGTTGGAAGGGAGTTTGAAGTTTCAAGCATAGTGAAACAAGTAGTTGATGCTAGT
AATCAATATGTTACATCTATCTTACCCATTATGGGTATGGGTGGAATCGGAAAAACAACTTTGGCAAAGACAATCTTCAA
TCATGAGGAGATCAAAAGACATTTTGATGAAACAATATGGATATGTGTGTCCGAACCATTTCTTATCAACAAGATTTTGG
GAGCAATTTTACAAATGATAAAGGGTGTTTCTAGTGGCTTGGATAATAAAGAGGTTCTACTTCAAGAGCTTCAAAAAGTG
ATGCGAGGTAAAAGATATTTTCTTGTGCTTGATGATGTTTGGAATGAAAATATTGCTTTATGGACTGAATTGAAAAAATG
TTTACTGTGTTTTACTGAAAAATCTGGAAACGGTATCATTGTAACTACGAGAAGTATTGAAGTTGGAAAGATTATGGAGA
GTACTCTTCCTAGCCATCATTTGGGAAAATTATTTGATGAACAATGTCGGTCTTTGTTTAAAGAAAGTGCAAATGCAGAT
GAATTGCCAATGGATCCAGAGTTGAAGGATCTTCAAGAAGAATTGGTGACAAGGTTTGGTGGTGTACCATTTGTTGCAAG
ACTTGGAAGCTCAATGAAACACCTTCCATATAATTTGAGCAAGTTGGTTAGTTTAAGACATTTAAAGTTCTCAATACCAC
AAACGCCTCCACATTTGAGCCGGTTGACTCAACTACAAACGTTGTCTGGTTTTGCAGTTGGATTTGAGAAGGGTTGCAAA
ATAGAAGAACTTGGATTTTTGAAAAACTTCAAAGGTAGATTAGAACTTTCAAATCTCAATGGAATTAAACACAAAGAGGA
AGCCATGAGTTCCAAATTGGTAGAAAAGAACTTATGTGAGCTATTCTTGGAATGGGATTTGCATATTTTAAGAGAAGGTA
GCAACTACAATGACTTGGAAGTGTTAAAAGGGCTTCAACCACACAAAAATCTTCAATTCTTGAGTATCATAAACTATGCT
GGCCAAATTTTGCCTCCTGCCATTTTTGTTGAAAATTTAGTTGTGATACATCTAAGACATTGTGTAAGATGCGAAACACT
TCCAATGCTTGGAGAATTACCTAATTTGGAGGAACTAAATATTTCCAACTTACATTGTCTAAGATGTATTGGGAATGAAT
TCTACGGAAGTTATGATCATCCCAACAACCATAAGGTTTTATTTCGCAAGTTGAAGAAATTTGTACTCTCTGAAATGCAC
AATCTAGAGCAATGGGAAGAATTAGTATTCACATCAAGGAAAGATGCAATTTTTCCTCTTCTTGAAGACTTGAATATTCG
TGATTGTCCTATATTAACAAATTGCCCAAAGATCGATCTACAACTCTGCACTTCCATTGAGGATCTAAAGATTGTTGGGT
GCCTTGAAATGATACTAAATGTGCAAAATATGCATACCTTGTCTCGTTTCTCTATGAATGGGTTGCAAAAGTTTCCCCAA
GGACTATCTCATCTCAAAAACTTGAAAGAAATGATAATCACTGAATGCTCACAAGATTGTGACTTTACTCCTCTTATGCA
ACTTTCTTCACTCGTAAATCTTGATTTGGTTCTTTTCGCGGGGAACGGGGCCGTGCAACTTCCTCAGCAACTCCAGCATC
TCACAGCCTTAAGATCTTTGATCATTAATGATTTTGATGGAATTGAAGTTTTACCAGAATGGTTGGGAAATCTTGCATCT
TTGGAAGTTTTGGGACTTTATTATTGTAGAAGTCTGAAACAGTTTCCTTCAAAGAAAGCCATTGCAATGTCTCACCCAAT
TAGTCCATGTGGATGTCTTTGGTTGTCCACAACTACCCAAGTTCGGAGATTTTGTGCCATGATGTTCTAA

>Cucsa.094650
ATGGCTGAATTCCTTTGGACATTTGCTGTGGAAGAGACGTTGAAGAGAACGGTGAACGTTGCAGCTCAGAAAATTTCTCT
CGTTTGGGGTTTGGAAGATGAACTTTCAAATTTAAGCAAATGGCTACTCGATGCTGGAGCCCTTTTGCGCGATATCGATA
GGGAAATACTTCGCAAGGAATCGGTGAAGAGATGGGCAGATGGGCTTGAAGATATCGTTAGTGAAGCTGAGGATCTTTTG
GACGAGCTTGCTTATGAAGATCTTCGAAGAAAAGTGGAAACAAGTTCAAGGGTGTGTAATAATTTCAAATTTTCTTCTGT
TCTTAACCCTCTTGTTCGTCATGATATGGCCTGTAAAATGAAGAAAATTACTAAAATGTTAAAACAACATTATCGCAACT
CTGCTCCTTTAGGGCTTGTTGGGAAGGAATCCATGGAGAAAGAAGATGGAGGTAATAATCTTAGGCAGATTAGGGAAACA
ACTTCGATTCTGAATTTTGATGTTGTGGGAAGGGAAACTGAAGTTTTAGACATATTGAGATTGGTGATTGATTCTAGTAG
TAATGAGTATGAGCTTCCTTTGTTGATTGTACCGATTGTAGGGATGGGTGGAGTTGGAAAAACAACTTTGGCGAAATTGG
TTTTTCGTCATGAGTTGATCAAGAAACATTTTCATGAAACAATATGGATATGTGTGTCGGAACACTTCAACATCGACGAG
ATTTTGGTAGCAATTTTGGAAAGTTTGACGGATAAAGTTCCAACCAAAAGGGAAGCTGTACTTCGCAGGCTTCAAAAAGA
GTTGCTAGACAAAAGATGTTTCCTTGTTTTGGATGATGTTTGGAATGAAAGTTCTAAGTTGTGGGAAGAGTTAGAAGACT
GTTTAAAAGAGATAGTTGGGAAATTTGGAATCACCATTATAGTAACTACAAGGTTGGATGAAGTTGCTAATATTATGGGA
ACAGTTTCGGGTTATCGTTTGGAAAAGTTACCTGAAGACCATTGTTGGTCCTTATTTAAGAGAAGTGCAAATGCAAATGG
AGTAAAAATGACTCCAAAGTTGGAGGCTATTCGAATAAAGTTGCTTCAAAAAATTGATGGCATACCGCTTGTTGCAAAAG
TTTTGGGAGGAGCCGTGGAATTTGAAGGAGATCTTGATAGGTGGGAGACCACACTTGAAAGCATAGTAAGAGAAATTCCA
ATGAAACAAAAAAGTTATGTGTTGTCCATATTACAATTAAGTGTGGACCGTCTACCCTTTGTGGAAAAACAATGTTTTGC
CTATTGTTCAATTTTTCCTAAAGATTGTGAAGTTGTTAAAGAAAATTTGATTAGAATGTGGATAGCACAAGGGTTTATTC
AACCAACAGAAGGAGAGAACACGATGGAGGATCTGGGAGAAGGGCACTTCAACTTCCTCTTATCTCGCTCCTTATTTCAA
GATGTCGTCAAGGATAAGTATGGGAGAATTACTCACTTTAAGATGCATGATCTAATACATGATGTTGCCCTTGCCATTTT

GTCAACTCGTCAAAAGTCGGTATTAGATCCTACTCATTGGAATGGAAAAACGTCAAGAAAGTTGCGCACCTTACTTTACA
ATAACCAAGAGATCCACCATAAAGTTGCAGACTGTGTTTTCTTGCGTGTTTTAGAAGTGAATTCCTTACATATGATGAAT
AACTTACCAGACTTCATTGCTAAGTTGAAACACTTGAGATACCTTGACATTTCATCATGTTCTATGTGGGTTATGCCCCA
CTCTGTTACTACGCTTTTCAATTTACAGACACTGAAGCTTGGAAGTATAGAAAATCTTCCAATGAATTTGAGAAATTTGG
TTAGACTACGTCACTTAGAATTCCACGTCTATTACAACACAAGGAAAATGCCTTCTCATATGGGTGAGTTGATTCATCTT
CAAATATTGTCTTGGTTTGTTGCAGGGTTTGAGGAAGGCTGTAAAATTGAAGAACTCGGAAATTTGAAAAATTTGAAAGG
TCAATTGCAACTTTCAAATCTTGAGCAAGTGAGGAGTAAAGAAGAAGCTCTAGCTGCAAAATTGGTCAATAAGAAAAACT
TACGTGAGCTAACTTTTGAATGGAGTATAGATATTTTACGAGAATGTAGCAGCTACAATGACTTTGAAGTGTTGGAAGGA
CTTCAACCACCCAAAAATCTCAGTTCTTTGAAAATTACCAACTTTGGAGGGAAATTTTTGCCTGCTGCTACTTTTGTTGA
AAATTTGGTGTTCCTATGTTTGTATGGTTGTACAAAATGTGAAAGGCTTCCAATGCTTGGACAATTAGCCAACTTGCAAG
AACTTAGTATTTGTTTCATGGATAGTGTGAGAAGTATAGGGAGTGAGTTTTATGGCATTGACTCCAACCGAAGGGGTTAT
TTTCCCAAGTTGAAGAAATTTGACTTCTGTTGGATGTGCAACCTAGAGCAATGGGAATTAGAAGTGGCAAATCATGAGTC
AAATCATTTTGGTTCTCTTCAAACTCTAAAGTTGGATAGATGTGGCAAATTGACAAAACTGCCAAATGGGTTAGAATGTT
GCAAATCTGTTCATGAGGTGATAATATCAAATTGTCCTAACCTTACCTTAAATGTAGAGGAAATGCATAACCTGTCTGTT
TTATTAATAGATGGGTTGAAGTTTTTGCCAAAAGGATTAGCTCTCCACCCTAACTTGAAGACCATAATGATTAAAGGATG
CATAGAGGATTATGATTATAGCCCTTTCCTAAACTTGCCTTCTCTTACAAAACTTTACTTGAACGATGGCCTTGGAAATG
CCACCCAGCTTCCTAAACAACTTCAGCATCTCACTGCCTTAAAGATTTTAGCCATTGAAAATTTTTATGGCATTGAAGTT
CTTCCTGAATGGTTGAGAAAGCTTACATGTTTGGAGACTTTGGATCTTGTTCACCTGGTGAAATCTTTACCATCTCTTCA
AGGAGAAGACAATCGTGTGGCGGAAATTGCGTATGAAACGGGTGAGATGGAAACATACGAATGTGACAACAAAAGATCAT
GGTTTCGACTACATTTAAATTTTTACGGTTGTACCTTAGTAGAGCTTGTTTAA

>Cucsa.094660
ATGGCGGATTTCCTATGGAGCTTTGCTGTAGATGAAGTGTTAAAGAAGACAGTGAAGCTTGTGGCAGAGCAAATTGGCAT
GTCATGGGGGTTTAAGAAGGATCTTTCAAAACTAAGGGACTCTTTACTAATGGTAGAAGCCATCCTACGTGATGTTAACA
GAATCAAGGCAGAACATCAAGCCTTGAGGCTATGGGTGGAGAAGCTTGAACATATCGTTTTTGAAGCCGACGTTTTACTC
GACGAGCTCTCTTACGAAGATCTTCGACGCAAGGTGGACGCCAGGCCGGTACGTAGTTTCGTTTCATCCTCAAAAAATCC
CCTTGTTTTTCGCCTCAAAATGGCCAATAAAATTAAAGCTATTGCTAAAAGGTTAGACGAGCATTATTGTGCAGCGAGTA
TCATGGGGCTTGTTGCTATAACATCCAAAGAAGTCGAGTCCGAACCTAGCCAAATTCTAGAGACAGACTCGTTTCTTGAT
GAGATTGGAGTTATAGGGAGGGAAGCTGAAGTATTAGAGATAGTGAATAAACTACTTGAACTTAGCAAACAAGAAGCAGC
TCTATCTGTTTTACCAATTGTTGGTATAGGTGGACTAGGAAAAACATCTTTGGCGAAGGCGATATTTCATCATGAAATGA
TAAGGGAGAATTTCGATAGAATGATATGGGTGTGTGTGTCTGAACCTTTTGTTATCAACAAGATTTTAAGAGCAATTTTG
GAAACTCTTAATGCTAATTTTGGTGGATTAGACAATAAGGAAGCTTTACTTCAAGAGCTTCAAAAATTGTTGAGGAACAA
AAAGTATTTTCTGGTGCTTGACGATGTCTGGAATGAAAATCCTGATCTGTGGAATGAGTTAAGGGCTTGTTTGCTAAAGG
CCAATAAAAAATTTGGAAGTGTTATTGTTGTGACTACTAGGAGTGATGAAGTTGCAAATATTGTGGAGACAAATCATCAA
AGACATCGTTTGAGAAAGTTATCAAATGATTATTGTTGGACTTTATTTGAAAAATGTGCATTTGGAAGTGATTTGCCAGT
GACTCCAAGAGTTGATCATGTAATCAGAGAAGAGCTTGTTAAAAGATTTGGTGGCATACCTTTGGTTGTGAAAGTGTTTG
GAGGAATGGTGAAATTAGACAAGAATAAATGTTGTCAAGGATTGCGATCAACTTTGGAAAATCTAATCATAAGTCCATTA
CAATATGAAAATAGTATTTTATCTACCATAAAATTAAGTGTGGACAGGCTGCCATCATCTTCATTGAAGCAATGTTTTGC
CTATTGTTCAAACTTTCCACGAGGCTTCTTATTTATAAGAGAACCACTTGTTCAAATGTGGATAGCACAAGGGTTTATTC
ATCTACCTAGTGGGAGCAATGTAACGATGGAGGATATTGGAGCAAACTACTTTAATACTTTGTTGTCTCGCTCTTTGTTT
CAAGATGTCGTCAAAGATGACAGAGAAAGAATTCTGTATTGCAAGATGCACGATGTTGTACATGATGTTGCATGTGCTAT
TTCAAATGCTCAAAAATTGAGACTGAGTGGCAAATCTAATGGAGACAAAGCTCTTTCGATCGGTCATGAAATTAGAACAC
TTCATTGCAGTGAAAATGTTGTTGAACGGTTTCACCTGCCAACCTTTGATAGTCATGTATTTCACAATGAGATCAGCAAC
TTCACCTACTTGTGCGTTTTAATTATTCATTCATGGTTTATACATCAACTGCCAGATTCAATTGCTAAGTTGAAGCATTT
AAGGTACCTCGACATTTCACACTCTCTAATAAGAACGCTTCCAGACTCTATTGTTTCACTCTATAATCTGCAGACATTGA
GGCTTGGAAGTAAAATTATGCATCTTCCTACAAAATTGAGAAAATTGGTCAATTTAAGGCATTTAGAATTCTCTCTCTCA
ACTCAAACTAAACAAATGCCTCAACATCTGAGTCGATTGCTTCAACTTCAAACGCTTTCGAGTTTTGTAGTCGGTTTCGA
CAAAGGATGTAAGATAGAGGAACTTGGACCACTGAATAACCTTAAAGGTGAACTAAGCCTTTTCCATCTTGAGCATGTCA
AAAGTAAAACCGAGGCTATGGCTGCAAATTTGGCAATGAAGGAAAACATTTCTGATCTATATTTTCAATGGAGTTTGTTA
AGTGAAAGAGAAGATTGTAGTAACAATGATTTGAATGTGTTGGAAGGGCTTCGACCACACAAAAACCTTCAAGCCTTGAA
AATTGAAAACTTTGGAGGTGTTCTGCCTAATGGCCTCTTTGTTGAAAATTTGGTGGAGGTAATTCTATATGATTGCAAAA
GATGTGAAACTTTGCCAATGTTGGGGCACTTATCTAAGCTTGAATTACTTCATATTCGTTGCTTAGATAGTGTAAAAAGT
ATTGGGGATGAATTTTATGGGAACAATAATAGTTACCACAATGAGTGGTCTTCATTGTTATTCCCTAAACTCAAGACCCT
TCATATTTCCCAAATGAAAAGTTTAGAGCTTTGGCAAGAAATAGGGAGTTCATCAAACTATGGTGCGACCTTTCCTCATC
TTGAAAGCTTGAGCATTGTTTGGTGTTCGAAATTGATGAATATTCCTAACCTTTTTCAAGTTCCTCCAAAGCTTCAATCT
CTCAAGATTTTTTATTGTGAAAAATTGACAAAGTTACCACATTGGTTAAATCTCTGCAGCTCCATTGAAAATATGGTCAT
ATGCAATTGTCCTAACGTTAACAATAATTCTCTTCCAAATTTGAAAAGTATGCCAAACTTGTCGTCCTTGAGCATCCAAG
CTTTCGAGAAGTTGCCGGAGGGGCTTGCCACCATTCATAACTTGAAAAGATTGGATGTTTATGGGGAATTGCAAGGTTTG
GATTGGAGTCCATTCATGTATCTCAATTCATCGATTGAAATTCTTCGTGACATTGATTCTTTGCCAGAATGGTTGGGAAA
CCTTACATCTTTAGAGACGTTAAATCTACGTTATTGCAAAAATTTGAAAAGTTTCCCTTCAATAGAAGCCAT

>Cucsa.094670
ATGGACTTATTGTATTCCAAAAATTATGAGGACAAAATACTGTCAAACCTCAGAGACTCTCTACTTATGGTGGAAGCCAT

TCTTCGTGATGTCGACAGAATTAAGGCAGAGCATCAAGCTGTGAAGCTATGGGTAGAGAAGCTTGAAGCTATTATTTTCG
AAGTCGATGTTCTACTGGATGAGCTCGCTTACGAAGATCTTCGCCGCAAGGTTGAACCCCAAAAAGAGATGATGGTAAGT
AATTTCATTTCTTTCTCCAAAACCCCTCTTGTTTTTCGTCTCAAAATGGCCAATAAAATCAAGAACATTGCTAAGATGTT
GGAAAGACATTATTCTGCTGCTAGTACTGTGGGGCTTGTTGCTATATTATCTAAACAGACTGAACCTGATTTTAGCCAAA
TTCAGGAGACAGATTCGTTTCTTGATGAGTATGGAGTTATTGGGAGAGAAAGTGAAGTTTTGGAGATTGTGAATGTATCT
GTCGATCTTAGCTATAGGGAGAATTTGTCTGTTTTGCCAATTGTTGGCATGGGTGGATTAGGAAAGACAGCTTTGGCTAA
GGTAATATTCAATCATGAATTGATAAAGGGGAATTTTGATAGAGCTGTATGGGTGTGTGTTTCGGAACCTTTTCTTATCA
AGAAGATTTTAAGAGCAATTTTGGAAACTCTTAATTCTCATTTTGGTGGCTTAGATAGTAAAGAAGCCTTACTTCAAGAG
CTACAAAAGTTGTTGAATGATAAAAAGTATTTTCTAGTTCTTGATGATGTTTGGAATGAGAATCCTATCCTCTGGAATGA
GTTGAAAGGTTGTTTGTTAAAGATTAGCCAAAGATCTGGAAATGTTGTTGTTGTGACTACTAGGAGTGACAGAGTTGCTG
AAATCATGGAGACACATTCTAGATATCATTTGACAAAACTATCCGATGACCATTGCTGGTCTTTATTCAAGAAATATGCA
TTTGGAAATGAATTGCTACGAATTCCTGAATTGGATATTGTTCAGAAAGAGCTCGTTAAAAGATTTGGAGGCATACCATT
GGCTGTAAAAGTGATGGGAGGAATCGTTAAATTTGACGAGAATCACGAGGGATTGCAGAAATCTTTGGAGAATCTAATGA
GACTTCAATTGCAAGATGAAAACCATGTTGTATCCACAATAAAGTTAACTGTAGATCGCCTACCATTGCCATCGTTAAAA
CAATGTTTTGCCTACTGTTCAAATTTTCCAAAAGACTTTAAGTTCAGAAAAGAAGCCCTTATTCAGATGTGGATAGCACA
AGGCTTTATTCAACCGTCTTTGGGAAGTGATGAAATGATGGAGGATATTGGTGAGAAGTACTTCAATGTTTTGTTGTCTC
GCTTCTTGTTTCAAGATATTGTCAAGGATAATAGAGGGAGAATTATATTCTGTAAGATGCATGATCTTATACATGATGTT
GCATGTGCTATTTCAAATTCTCCAGGATTGAAATGGGATCCTTCAGATTTGTTTGATGGAGAACCTTGGAGACGTCAAGC
TTGCTTTGCTAGCCTTGAACTAAAAACGCCAGATTGTAATGAAAATCCTTCTAGAAAGTTGCACATGTTGACATTTGATA
GTCATGTGTTTCACAATAAGGTCACAAACTTTCTCTACTTGCGGGTTTTAATTACACATTCGTGGTTTATATGTAAATTA
CCAAATTCAATTGCTAAGCTGAAGCATTTGAGGTATCTTGACATTTCATATTCTACCATAAGGGAGCTACCAGATTCCGC
TGTTTTGCTTTATAATTTGCAAACACTGAAGCTTTCAAGATTTTTAAACGGCCTTCCAAAAAAATTTGAGGAAGTTGGTTA
GTTTAAGACATTTAGAATTTTTCTCTGATCCTTGTAATCCTAAACAAATGCCTCAACATTTGGGTAAATTGATTCAACTT
CAAACGTTGTCTAGCTTTGTAGTTGGGTTTGATGATGGATGTAAGATAGAAGAACTCAGATCTTTGAGAAATCTTAAAGG
TAAGTTAAGCCTTTTATGTCTTGAGCGAGTGAAAAGTAAAAAGGAAGCCATGGCTGCAAATTTGGTGGAGAAGAGGAATA
TTTCATATCTGTCTTTTTATTGGGCCTTGAGATGTGAAAGATCAGAGGGAAGCAACTACAATGATCTGAACGTGTTAGAA
GGACTTCAACCACATAAAAATCTTCAAGCTTTGAGAATTCAAAACTTTTTAGGCAAACTTCTGCCCAATGTTATTTTTGT
CGAAAATTTGGTCGAGATATATCTACACGAATGCGAAATGTGTGAAACTTTACCAACACTTGGGCAGTTATCAAAGCTTG
AAGTACTCGAACTTCGTTGTCTATATAGTGTAAGAAGTATTGGAGAAGAATTTTATGGGAATTACCTTGAGAAGATGATT
TTATTCCCAACATTGAAAGCATTTCATATCTGTGAAATGATCAATCTAGAGAATTGGGAAGAAATAATGGTTGTATCAAA
TGGTACAATCTTTTCCAACCTTGAAAGCTTCAACATTGTTTGTTGTCCGAGATTGACGAGCATTCCAAACCTTTTTGCAT
CTCAGCATGAGAGTTCATTTCCAAGCTTACAACATTCGGCAAAGCTTCGATCTCTAAAGATTTTGGGATGTGAAAGTTTG
CAAAAACAACCAAATGGTTTAGAATTCTGCAGCTCCCTTGAAAACATGTGGATAAGCAACTGTTCTAACTTGAACTACCC
TCCAAGCTTGCAGAATATGCAGAATTTAACTTCTTTAAGCATAACCGAGTTTCGAAAGCTGCCAGACGGGTTAGCTCAGG
TTTGTAAGTTGAAAAGCTTGAGTGTTCATGGTTACTTGCAAGGTTACGATTGGAGTCCTCTTGTACATCTTGGTTCACTC
GAAAATCTTGTGTTGGTTGACTTGGATGGAAGTGGTGCAATACAACTTCCTCAACAACTTGAGCAACTCACTTCTTTGAG
ATCACTGCATATTTCGCATTTTAGTGGCATTGAAGCGCTACCAGAATGGTTCGGAAACTTTACATGTTTGGAAACGTTGA
AGCTTTACAATTGTGTAAACTTGAAAGACATGGCGTCGAAGGAAGCTATGTCAAAACTTACAAGATTAACGAGTCTACGA
GTTTATGGATGTCCACAACTTAATACTTTTATGTTTAATGGTATGTCACCAAATTCCTCGACGAGCTCTTCCCGAACCTT
ATCGACATTGGAATGA

>Cucsa.102240
ATGGCGCTGGAATTGGTGGGTGGGGCTGTTTTGGGGGCTGTCGTTGGGGAGCTATTCAAAGCGATCTTGAATCTGGGTGA
AAGGGCCATCAGTTTCAATCCTGTTCTTAAGGATATCCGTTCCAAGCTTAATGCTATAATGCCTTTGGTGAAGCAAATCG
ATGAGCTTAATGATTATCTCGATTACCCAAAAGAAGAAACAGAGAAATTGAGGGGTCTGATGGATGAAGGGAAGCAGTTG
CTTCTCCAGTGCGGCGATGTGAAATTGGGGGATCTTAATTATTTGAAGAGACCATCTTACACCCAAAAGCTTCGGGAATT
GGATACTGCACTTCGAAGCTTCATGGATGTTTTGATGTTGCAGATGGCTAGAGATCAGAAGAAGAACATGAAGATGATGA
ACCAAATGATGGAGATCATTTGTAGACTTGATAATAGAGGTGGGTCGAGTAAACCTATGGATTTGTTTGTTCCACCATGT
CTGGTTCCTCAACTGCGAGAAGAAACCGTTGGGTTGGAGAAGCCAGTTAAGGAGTTGAAGGTGAAACTTCTCAAAAATGG
GGTTCAAATGTTGGTGGTGACAGCTCCTGGTGGCTGCGGAAAAAACCACACTGGCCTTAAAATTTTGCCACGACAAAGAAG
TCAAAGATATATTCCAGGAGAAGATCTTTGTCCCAGTTTCAAGAAAACCAGATTTGAAGCTTATATTGAAAGATATAATT
GAAAGCCTTAGAGGAATTCAATTGCCTGATTTGCAAAGTGATGAACGTGCATTCTGCTATTTAGAATTGTGGTTGAAGCA
GACAAGTGTAAATCGTCCTGTTTTGATTGTGTTAGATGATGTGTGGAGTGGGCAAGAATCTGAAGTTCTTCTTGATAAGC
TGTTTCAATTGCCTTGCTGCAAGATCTTGGTCACTTCTAGGTTTTATTTCCCAAGATTTAGTGAGTCTTATTATTTGGAA
CCTTTGAACCATGAGAATGCAGTACAACTTTTTCGTCGTGCAGCATCACTGGACAAAGGAATTTCTAAGCTCCCCGATGA
TGAAACTGTAGAAAAGATAATTGGGGGATGCAAGAGACTACCTCTTGCACTGAAGGTAATCGGGAGGTCTCTTTCCCACA
AACCGACATCTGTTTGGAAAGTAACGGGGAGGAATTTGGCTAGAAGTGGCTCCATATTTGATTCTGACAATGAACTTCTT
GAATGCCTTCAGAGCAGTTTGGATGTCTTGGATGATAACATGGTAACTAAGAAGAGTTTCATGGATTTAGGCTCTTTTCA
TGAAGATCAAAGAATTTCTGCTTCTACCTTCATTGACATGTGCACAGTTTTGTACACACTAGACGAAAGTGAAGCAATGG
TTACCCTTGACGAACTATCCTCTCGAAGTCTAGTTAATTTTGTCACAGCGAGAAAATATGGATATGATGATGACTTTTAT
GAAGAGTACTCTTTTACTCAGCATGATATTCTCAGAGATTTGGCTATTCACTTGATGAATATGGAGCCCATAGAACAAAG
GAAAAGATTGATCTTAGACATTAATGGAAATGATCTTCCCAAATGGTGGGTTGATCAAGAAAAGCATACTTCCTATGCTC
GCCTTATATCCATAACCACAGATAAGAGATTCTCAGCAAGTTGGCCTGACATGGAAGCACCTGAAGTGGAGGTTCTGATT

CTTAATCTTCAGTCAAGAACTTACAACTTGCCTGGGTTCATCAAAAGAATGAATAAGCTGAAAGTTTTGATAATCACATA
TTTTGGTTCTTTTCTAACTGAGGTGACAAGTGAAGATAATCAACTACTCGACAGCCTAACAAGTCTTGAACGAATCAGGT
TTGAGCGGATTTCAGTTCCTATCTTTAGTAATCCAAACCCGAAACCACTGATAAATCTGCAGAAAATATCCTTCTTTATG
TGCAAATTTGGTCAAACATTCATGGATCCTTCAACCCCAATCTCAGATTTGTTGCCAAACCTGCTGGAGATTTCCATAGA
CTTCTGCAACAATTTGAGTGAAGTCCCCAATAGGTTGTGTGAAATTGTCAGCTTGCAGAAGCTGAGCATTACAAATTGCC
ATGGACTATCTTCCTTGCCAGAAGATGTAGGGAAGTTGATTAATCTAAAAAATCTAAGGCTAAGATCTTGCATTCATTTA
GAAGAGTTTCCAGAGTCGACAACGAAGCTTCGGGAATTAGTCCTGCTTGATATATCTAACTGTATTGGTCTTGCCAAGCT
TCCCGAGAAGATTGGTGAATTTCATAATTTAGAAAAGCTTGACATGAGACACTGCTGGAGTTTGAGCAAGCTGCCACTGT
CGATTGGAAAGCTGAAAAATGTGAAGTTTTTATGTGATAGAGAGGTTGGAGAGTGGTTGAGAAAGGTTGCACCTCGCCTT
GCCAAACAGGTGAAAGTGCAAGAGGAAGAAGCCAACCTGGAGTGGCTTGGTTTTTGA

>Cucsa.123410
ATGGCGGTTACAGATTTCTTTGTTGGAGAGATAGCCACTGAGCTTCTCAGAATGATGGTACAACTTTCGACCAAATCCTG
CCTTTGTAAAACGACGGCAGCTCAAATCGCCAATTCTATTCAACAAATTCTGCCGATTATTGAAGAGATCAAGTACTCGG
GAGTTGAATTACCCGCTCATCGCCAATTTCAGTTAGATCGCTTCAGCGAAACTCTTAGAAGAGGCATCGAGATTTCCGAG
AAGGCTCTTCAATGTGGCCGATTAAACATTTACAGAAACTTACGGCTCGCGAGGAAGATGGAGAAGCTTGAAAAGGATAT
ATGTCGATTCATTAATGGCACCATGCAGGCGCATATACTGGCCGACGTGCATCATATGAGATTCCAGACCACCGAGCGGT
TTGACCGGCTTGAAGGTGTTTTGTTGGAGCGGCGGCTTGAGTCGATGAAGATTAGAGCAGATGCTTCGGGAGAGGAAAGG
TGGTGGGTTGAGGAGGCGTTTAAGAAGGCCGAGGAGGAGGAAAGGTATGAGAGTAATTTCGTGAATATAGGAACTGGATT
GCGTGTGGGGAAGAGAAAATTGAAGGAGCTGGTGATTGGAAAGGAGGATTTAACGGCGGTTGGGATTAGTGGAATTGGGG
GTTCGGGGAAGACTACTTTAGCTAGAGAATTCTGCAAAGATCCGGAAGTTCGAAGACACTTTAAAGAGAGAATTTTGTTC
TTAACGGTGTCACAGTCCCCTGATGTGGAGCAGCTGAGGAGAACGATCTGGGAATTTGTGATGGGTAGTGATAGTGTCAA
TTCTAATAATTTGATTTTACATGGGAGGCCTTCAAATTCAGCGCTTTTGGTTCTGGATGATGTGTGGTCAATTTCAGTTC
TTGAAAATGTTATTCCAAACGTAACTGGTTGCAAAACTCTTGTTGTTTCACGATTCAAATTCCCTGAAGTTCTTAGAGAA
ACTTATGAAGTAGAGTTGTTGAAAGAAAGTGAAGCAATTGCTCTGTTTTGCCACTCAGCTTTCGGACAACAGTCGATTCC
TTTGTCTGCTAATCACAACTTGGTCAAACAGGTTGTGAATGAATGCAAATGTTTGCCTCTGGCTCTTAAAGTCATAGGAG
CATCACTCAGAGGACAGAGCGAGATGTTCTGGAATAATGCCAAGTCTAGGTTGTCACGTGGCGAGCCTATTTGCGAGTCC
CATGAGAACAAATTGCTTCAAAGAATGGCAATCAGTATTGAACGCCTCTCGAGTAAAGTGAGAGAATGTTTCCTCGACCT
GGGATGCTTTCCTGAAGACAAAAGAATTCCTCTTGACATTCTCATCAATGTTTGGAAGGAGTTACATGATCTTGATGACG
AAGAAGCTCTTGCTGTTCTTTTCGAGTTATCTCAGAAGAATCTTCTTACGTTGGTGAAAGATGCACGCGGTGGTGACATT
TATAGCAGTTATTATGAGATGTATGTCACTCAACACGATGTATTAAGGGACCTTGCCCTTCATTTCAGTTGCCAGGAGAA
TGTGAACGACCGCAAGCGATTACTGATGCCAAAAAGCGACACAGAGCTTCCAAAAGAATGGTTAAGGAAATCGGAACAGC
CATTTAATGCCCAACTTGTTTCAATTCACACAGGTGAAATGGAAGAAATGGATTGGGCGCCTATGATATTTCCTGAAGCT
AAAGTGCTCATTTTAAACTTCTCCTCGAGTGGATACTTCTTGCCTTCTTTTCTTTGCAACATGCCGAAGATAAGAGCATT
AATTGTGCTAAATAACAATGCAACACATGCAACTCTCACCAATTTCTCAGTTTTTTCTAGTTTGGTCAACTTGAGAGGCA
TCTGGCTGGAAAAAATTTCCATGACACAACTATTCGATGCTTGCACGCCATTGAAACATCTAAGGAAGCTATCTCTTGTT
TTCTGCAAGATCAACAACAGCCTCGACGAGTGGGCGGTAGATGTATCCCAGATCTTCCCGTTTCTTTTCGAACTCAAAAT
TGATCACTGCAACGACTTGCGTAAGCTACCTTCAAGCATTTGTGAGATGCAAAGTCTCAAGTGTCTTAGTGTCACCAACT
GTCATAATCTCAGTCAACTCCCTACCAACTTATGGAAGCTGAAAAATCTACAAATCTTGAGACTTTTTGCTTGCCCACTC
CTCAAAACTCTATCCCCAAGCATTTGTGTACTTTCTTGTCTAAAGTACATTGACATCTCCCAATGTGTTTACTTAACCAG
CCTTCCTGAAGAAATTGGCAAGCTGACAAGCCTAGAGAAAATTGACATGAGAGAATGCTCACTCATAAGGAGACTACCTA
GATCAGTTGTGTCTTTGCAATCTCTCTGTCACGTAATCTGCGAAGAAGACGTCTCGTGGCTATGGGAGGATTTGAAGAGT
CATATGCCTAATTTGTACATTCAAGTCGCCGAGAAATGCTTCAACTTAGATTGGCTCAAAGAGTGA

>Cucsa.128020
GGGGCGTCAAGTAAAAGTCACTTGCTGGAGAATTGGCTTCAAAAACTGGAAGAAGCTCTTTATGCTGTGGATGTGCTTGA
TGAACTCTCCAGGGAGGCTCTTCATCGAGAAGTGATGACTAGAGATAAAAATGCAAAACAAGTAAGGATCTTCTTCTCCA
AATCTAATCAAATCGCATTTAACTACAGGATGGCTCGTCAAATAAAGAAAGAGGTGATCGGGAGGAATGATGATAAGAAA
AAGGTAAAGCACCTTTTATTGGATGATGTATGGAATGAAAGTGAAGAGAAATGGCATGGGTTGAAACCTTTGTTAATGAG
TGGTGCGAAAGGGAGTAAGATTTTCATCACAATGCGTGATAGTAAAATAGCTGCAGAAATTGAAAGCATGACTTCTTTAT
TCACTTTAGAAGGCTTACCGAAGAGTAAATCTTGGTCATTATTTAGTAAAGTGGCATCCAAAGAAGGCAAGGTGCTTGAA
AATTCAAACTTGTTACAGTTAGGAAAAGAAATTTCAGTGAAATGTGGAGGTGTTCCCTTGTTTAGACATCTTCGATATCC
AAACCTCCCTGAAAATATTATGAAATCACTTCCAAATTCAATCACTGAATTGCAAAACTTGCAAATGCTAAATCTAATGT
ACTGTAGCGAACTAATGGAATTGTCAAGGGACACCAGAAATCTTATTAATCTTAGGCATCTTGATTTCAATAGTTCTACC
TTAACCCATATGCCCGAAGAGATGGGGAAGTTGAATTGTCCACAGACACTAAGTTATTTTGTCTTGGACTACGAAAGGTC
TAATCAGCTGAGTGAACCGACTGTGTTGATCCATTTAAAAGGAGATTTAAGAATAAAAAATTTAGAGCAACTGAGTTACA
ATCCATCTGAACTCAGTTTAGTAAACCTGAAAGACATAAAAGGCTTCAAGAATCTGGAACTAGAATGGAACCTAAGCCCA
GATGATCAAGAATATGAAGGTGAGGATGATGAAACTTCAGCAATGGAAGGCCTAGAACGACATTCAAATGTTGAATCCTT
GCACATTGACGGGTACAGCGGAGTAGGATTACCCAATTGGGTGTCCACCTTGCTCTTGAAGTTAACTAGAATTACAATTT
ATAAATGCCATAGATTGCAACATCTAACACAGATAGCCCATCTTCAGGCACTCACATTTCTATTTTTGGATGACATGAGC
TCTCTTGAGTTCATAGACAAGAATGAACCATCTTCCTCGTCCTCTTTCCCATCTCTCGAGCTTCTAATTATCGAAAACAT
GCCGAATTTGGAAGGATGGTGGGAATTAGGGAACACTCAAAAAAATTGGTTACCACCAACTTTCTCTACGCTGATTTCCC
TGCATATCTCTAGGTGTCCTAAGTTTAGATTCATGCCCAAGCCAGCTTCAACAGGAACAGTTGTGTTCTTACGTGATGTG

AGCGTTCAATTGGAGACTACATTAGATCCATTGTGGGGTTTGGAATGTCTAACACTGGAAAAAATTAAGGATCTCAAATA
TCTAGAAACCATGGAATCTCAGCTAAACATCAGTTCTTTGCCAATACAACTTCGAGATTTAGAAATAAATAAATGCTCGA
ATTTGATGAGTTTACCTGAATGGATTAGCAGCATTACTTCGCTAGAGGAGCTGGAGATTATGAAATGCCCGAAACTAAAA
GTAGCTTTTGCATCGGCGGTTGCCCCGAAGTAG

>Cucsa.128030
ATGGCCGAAGCAATTCTCTTCCAAGTTGCTGGGGAGATCTTGATGAAGCTAAGCTCTCAAGCTTTCCAGCGTCTTGGGAT
GCTATTTGGGCTGAAGGGTGATCTTAACAAACTCACAACAACTGTTTCCACCATTAAGGATGTGCTTCTTGATGCGGAGG
GACGTCAAACTAAAAGTCACTTGCTGCAAAATTGGCTCCATAAGCTGGAAGAAGCTCTTTATGATGCAGAGGATGTGCTT
GATGAACTCTCTACGGAGGCTCTCCGTCGAGAACTGATGACTAGAGATCATAAAAATGCAAAACAAGTAAGGATCTTCTT
CTCCAAATCTAATCAAATTGCATTTAATTATAGGATGGCTCGTCAAATAAAGAATATTTGGGAGAGGCTAGATGCTATTG
ATGCTGAAAAAACACAATTTCACTTGCGTGAAAACTGTGAATCACGGACTCAATACGGTTCATTTGATCGAATAATGATG
GGAAGGGAAACTTGGTCTTCTTCAAATGACGAGGAAGTGATTGGAAGGGATGATGATATAAAAGAAGTAAAAGAGCGTTT
ATTGGATATGAATATGAATGTCACGCATAATGTTTCGTTCATTGCTATAGCTGGAATGGGTGGGATAGGCAAGACGACCT
TGGCTAAATCTCTCTACAATGACGAAGAGGTATCAGGATTTTTCGACTTAAAAATATGGGTTTGGGTTTCTGATCAATTT
GAGGTACAAGTGGTAGCGGAAAAAATGATAGAATCAGCAACCAAAAACAATCCTAGTGTAAAAGGAATGGAAGCTTTACA
AGCAAAGCTTCAGAAAGTGATTGGAGAAAGGAAGTATCTGTTAGTTATGGATGATGTATGGAATGAAAGTGAAGAGAAAT
GGCATGGGTTGAAATCATTGTTGATGGGTGGTGCAAGAGGGAGTAAGGTTTTGATCACAAAGCGTGACAGAAAAGTAGCC
ACAGAAATCAAAAGCATGACATCTTTGTTCACTTTAGAAGGCTTATCAGAGAGTAATTCCTGGTTATTGTTTAGTAAAGT
GGCATTTAAAGAAGGCAAAGAGTCCACAGATCCAAGCACGATACATTTAGGAAAAGAAATTTTAGTGAGATGTGGAGGTG
TTCCTCTTGTTATAAGACATGTTGGACGCATGTTATACTCTAAAACTTCACAAGAAGAGTGGATGTCCTTCAAGGATAAT
GAACTTTTAGAAGTCATTCAACAAGACAATGATATGACATCAATATTAAAATTGAGTTATAACCATCTCCCACCAAATTT
GAAACGATGTTTTGCATATTCATCCCTGTTTCCCAAAGGATATAAAATAGAAATAAAAGACCTAATAAGGCAATGGGTGG
CTCAAGGTTTTATTGAAGTGTCAAATGGAAGAAAATCCTTGGAAGATACAGGGAAGGACTATTTTAACGAATTATGTTGG
AGGTTTTTTTATGCAAATTCTAGTGATGAGTGTAACATCAATGATATTGTTTGTATGCATGATGTGATGTGTGAGTTTGT
AAGGAAGGTGGCAGGAAATAAATTATATGTACGTGGAAATCCCAATAATGATTATGTTGTCAGCGAACAAACACTTCACA
TTTCATTTGACTACGGAATACAATCATGGCAAGATGTTCTATCTAAATTATGCAAGGCTAAGGGATTAAGAACAATCCTT
TTATTATTTCGTCCCTACGAGAAAATGAATAAAATTGATAAAGCTATTTTGGATGAATTATTTTCCAGTTTTCCACGTTT
GCGAGTATTAGATCTTCATTTCTCGCAGATTTCTGTAGTGCCGAAGTCTATAAAAAAACTTAGACACCTTCGATATTTGG
ATCTCTCTGAAAATGATATGGAATTAATTCCACATTCTATCATTGAATTGCAAAATTTGCAAACACTAAATCTAACAGAA
TGCTATGAGCTAAAAGAATTGCCAAGGGACATCGACAATCTTGTAAATCTCAGGCATCTTACCTTTGAACCTTGTATGGA
AGTAACTCCTACATCGGAGGGGATGGAGAAGTTGACTTGTCTACAAACAATCAGTTTATTTGTGTTTGACTGCAAAAAGA
CCAATAAGCTATGGGAATTGAATGATCTCAGTTATTTGACAGGAGAGTTAAAAATCATAGGTTTAGAGAAGTTGAGGTCT
TCTCCATCTGAAATCACCTTAATAAACCTGAAAGACAAAAAAGGTTGGCAAGGTTTAAATTTGGAATGGAAACTGGGCAA
GGATGAATACGAAGGTGAGGCTGATGAAACAATAATGGAAGGCTTGGAACCACATCCAAATGTTGAATCGTTGAGCATTA
ACGGGTACACTGGAGGAGCATTGCCCAATTGGGTGTTCAACTCGCTTATGAAGTTAACTGAAATTGAAATTGAAAATTGC
CCTAGAGTGCAACATCTACCTCAGTTCAACCAGCTTCAGGATCTCAGAGCTCTACATTTAGTGGGCTTAAGATCTCTCGA
GTTCATAGATAAGAGTGATCCATACTCATCATCAGTGTTTTTCCATCTCTCAAGTTTCTACGTTTAGAAGATATGCCTA
ATTTGGAAGGATGGTGGGAATTAGGGGAATCAAAAGTAGTAGCAAGGGAGACATCTGGGAAAGCTAAATGGTTGCCTCCA
ACTTTTCCTCAATTAAGTTCCATGCCCAAGCTAGCTTCTATTGGAGCAGATGTTATTTTACATGATATTGGGGTTCAGAT
GGTGAGTACCATAGGTCCAGTATCGAGTTTTATGTTTCTATCAATGCATGGAATGACGAATCTCAAATATTTATGGGAGG
AATTTCAGCAAGATCTAGTTTCTTCAAGTACCTCAACAATGTCCTCACCTATTTCCCTTCGTTATCTGACAATAAGTGGA
TGCCCCTATCTCATGAGTTTACCGGAATGGATTGGCGTTCTCACTTCCCTTGAAACATTGCATATTAAAGAATGTCCAAA
ATTAAAATCACTACCAGAAGGAATGCAGCAACTCAAATCTTTGAAAGAACTTCACATAGAAGACTGCCCTGAACTAGAGG
ACAGATGCAAGCAGGGAGGAGAGGATTGGCCAAACATTTCCCACGTTCCCAACTTTACTTACAAAAATGCCTCTGACATT
GACACACCACAATCTTCTTCAGGTTTTTCACACCATCCCTTTTCAATCGTTCGTATCTCTGTTATATAG

>Cucsa.128100
ATGGCGGATTCAGTTCTGTTCAATGTTGCTGCAAGTGTTATTACTAAACTGGGATCTTCCGCACTTCGAGAACTTGGGTC
TCTGTGGGGAGTCAACGATGAGCTCGACAAACTCCAAAACACTCTTTCGGCCATTAAAGCCGTCCTTCTCGATGCAGAGG
AGCAACAGTCCAAGAGCCACACAGTCAAGGATTGGATTGCAAAGATCAAAGATGTTTTCTATGACATTGATGACTTGATT
GACGAGTTCTCTTATGAAACCTTGAGAAGACAAGTTCTTACCAAGGATAGAACAATCACCAAACAAGTACGTATCTTCTT
CTCCAAATCTAATCAGATTGCTTTTGGTTTCAAAATGGGTCAAACAATTAAAAAAGTTAGGGAGAAGCTAGATGCTATTG
CGGCTATTAAAGCTCAACTTCACCTCTCTGTGTGTGCGAGGGAGGTACGAGATAATGAGCCAAGGAAGGTACGAGAGACG
TCCTCATTCATACCCGAGGGGGAAATCATTGGTAGGGATGAGGATAGGAAATCTGTTATGGATTTTCTATTGAATACCAG
CAACATCACAAAGGATAACGTTGAAGTTGTTTCCATTGTTGGAATGGGAGGATTAGGAAAGACAGCACTCGCTCAAACTG
TCTATAATGATGAAAAAATAAACAATCGTTTTAAGTGGAAAATATGGGTGTGTATTTCTCAAGAATTTGATATCAAAGTA
ATTGTTGAAAAGATTTTAGAGTCTATTACGAAAACAAAACAAGAATCCCTTCAGTTGGATATATTACAAAGTATGCTTCA
AGAGAAAATTTATGGAAAAAAATACTTGTTGGTCATGGATGATGTGTGGAATGTAGACCACGAGAAATGGATTGGTCTGA
AAAGATTTCTGATGGGTGGTGCCAGCGGAAGTAAGATTTTGGTGACAACCCGTAATCTACAAACTGCCCAGGCTTCTGAC
ACGGTTTGGTTCCATCACTTAAAAGAACTTGACAAGGATAACTCTTGGGCGTTGTTTAGGAAAATGGCATTCTTAAACAA
AGAAGAAGAGCTTGAGAATTCAAATTTGGTTAGAATCGGTAAAGAGATTGTAGCAAAGTTGAAAGGTTATCCCCTTTCAA
TAAGAGTTGTTGGACGTCTGTTATATTTCAAAAACACAGAAATGGATTGGTCATCATTTAAGGACAACGAACTTGACTCA

ATTTTGCAAGAAGATGATCAAATTCAACCAATACTGAAGATAAGTTTTAACCACCTTCCACCTAAATTGAAGCAATGTTT
TACGTACTGTGCTTTGTTTCCCAAGGATTATGAGTTTAAAAAGAATGGATTGGTAAAACAATGGATGGCACAAGGTTTCA
TTCAAGCACATAATAAAAAGGCAATTGAAGATGTGGGTGACGATTATTTTCAAGAGTTAGTGGGGAGGTCATTCTTTCAA
GACATAAGAAAAAACAAATGGGGAGACTTAAAGTACTGTAAGATGCATGATTTGTTACATGATCTTGCGTGTTCGATAGG
AGAAAATGAATGTGTGGTTGTAAGTGATGATGTCGGGTCCATTGACAAAAGGACTCGACATGCCTCATTTCTCTTGAGCA
AGAGGCTAACAAGGGAAGTTGTATCAAAATCATCCATTGAGGTAACGAGTTTGAGAACATTGGATATTGATAGTCGTGCT
TCTTTCCGTTCTTTCAAGAAAACTTGTCACATGAACCTTTTTCAATTACGAACATTGAATTTGGATAGATGCTGCTGTCA
TCCTCCTAAGTTTGTTGATAAGTTGAAACATTTGAGATATCTTAATCTTTCTGGTTTAAATGTAACTTTCCTTCCCAATT
CTATTACCACATTGTATAATTTGGAAACACTTATCCTTCGTTACTGCCTTTGGCTAAGAAAATTGCCAAAAGATATTAAC
AATTTGATCAATCTCAGGCATCTTGATATTTATGATTGTTCCAGTTTGACTCACATGCCAAAAGGATTAGGTGGGATGAC
TAGCCTTCAGACAATGAGTATGTTTGTATTAGGAAAGAATAAAGGTGGTGATTTAAGTGCATTGAATGGACTTAAAAGCT
TGAGAGGATTATTATGTATTAAAGGTTTACAATTTTGCACAACTGCTGATTTAAAAAATCTGGAATTACACTGGGATATA
AAAATGGATCATGAAGATGCCTTAGATGATGGTGATAATGATGATGAGGGAGTTTTGGAGGGCTTAAAACCACATTCAAA
TATTCGCAAAATGATTATAAAAGGATACAGAGGAATGAAGTTATGTGATTGGTTTTCTTCTAATTTCCTGGGTGGTTTGG
TTAGCATAGAGCTTTCACATTGTGAAAAATTGGAGCATCTCCCACAGTTTGATCAATTCCTATATCTCAAGCATCTTCTT
CTTGGATACTTACCCAATATTGAATACATTGATAGCGGCAATTCTGTTTCTTCATCAACAACATTTTTTCCATCTCTCGA
GAAGCTAAGGATTGAGAGCATGCCTAAGTTGAAAGGGTGGTGGAAGGGGGAAATTTCATTTCCAACAACAATATTACATC
AACTCTCAGAATTATGTATTTTTTATTGTCCTCTGTTGGCTTCTATTCCACAACATCCATCTTTGGAATCATTGAGAATA
TGTGGTGTTAGTGTGCAACTTTTTCAAATGGTAATACGAATGGCTACAGACCTTTCTGAACATTCTTCTTCTTCTTCAAC
ATTGTCTAAATTATCTTTCCTTGAGATTGGAACTATTGATCTTGAGTTCTTGCCAGTGGAGTTATTCTGCAATATGACAC
ATCTTGAGTCTCTTATCATAGAACGCTGCAAAAGTTTACAAATGTCTTCTCCGCATCCTGTTGATGAGGATAATGATGTG
GTATGGAAAAAAACTCAGCAATCTCCGGACACTTCGGCTTGAGAGCATCCTCAAATTGGAGTATTTTCCCAAGAGTTTGAA
ATATATTACAAGTCTTGAAACTTTGAAGCTATCAAATTGTGAAAATTTAGTGAGTACGGAAGGGATTGGCGAACTCATTT
CACTATCACATTTGGAAATTGATAGATGTCCTAATTTACCTATATTGTCGGAAGATGTCGGCGACCTCATTTCCCTATCA
CACTTGCTTATTTGGAATTGTCCCAAATTAACTTCCTTGTCAGAAGGAATCACTCGCCTCACTTCACTCTCAAGTTTGTG
TCTTGAAGATTGTCCCAACTTAGTCTCCTTGCCCCAAGAATTTCTCCACCACCACAGCTCCTTACCAGGAGGACGGTTCT
TGAGAATTTTGAACTGTCCCAAATTGCAGATTCAAGACAAGAAACAAAAGGAAGAAGAAGAAGAAGACCAGGAGGATTGG
AATGAACTCATCCATGTATTGACCGGATGTAGGTAA

>Cucsa.128110
ATGGCGGATTCTATTTTGTTCAACGTTGCTGCAAATGTTATAACCAAATTGGGCTCTTCCGCGCTTCGAGAGCTTGGATC
ATTGTGGGGAGTTAATGATGAGCTTGGAAAACTACAAAACATTCTTTCAGCCATCAAAGCTGTGCTTCTCGATGCAGAGG
AGCAACAATCAGTGAGCCACGCAGTCAAAGATTGGATTTCAAAGCTTAGAGATGTTTTCTACGACGTTGATGACTTGATT
GATGAGTTCTCTTATGAAACCTTGAGAAGACAAGTTCTTACCAAAGATAGAACAATTACCAAACAAGTATGTATCTTCTT
CTCCAAATCTAATCAGGTTTCATTTGGTCACAAAATGAGTCAAAAAATTAAACAAGTTAGGGAGAAACTAGATGCTATCG
CTAATGATAAAACTCAACTCCACCTTTCTGTCCGTATGAGGGAGACACGAGATGATGAGTTGAGAAAGATGCGAGAGACT
TGCTCTTTTATTCCTAAGGGAGAAGTGATTGGTAGGGATGATGACAAGAAAGCTATTATAGATTTTCTATTGGATACCAA
CACCATGGAGGATAATGTTGAAGTGGTTTCCATAGTTGGCATGGGAGGATTAGGAAAGACTGCAGTTGCTCAATCTGTTT
ATAATGATGAGAAGATAAATGAACATTTTAAGTTGAAATTATGGGTGTGCATCTCTCAAGAGTTTGATATCAAAGTAATT
GTTGAAAAGATTATAGAGTTTATTGCGAAAAAGAAACCCGATTCTCTTCAATTGGATATACTACAAAGTATGCTTCAAGA
AAAAATTGATGGAAAGAAATACTTGTTGGTCATGGATGATGTGTGGAATGAAAGCCATGAAACATGGGTTAGTCTAAAGA
GATTTTTAATGGGTGGTGCCAAGGGAAGTCGGATTTTGATCACAACACGTAATCTGCAAGTGGCACAGGCTTCTGATACA
GTTCAGTTTCATCACTTAAAAGAACTTGACAATGAGAGCTCTTGGGCGTTGTTTAGAAAAATGGCATTTTTGAATGAAGA
AGAAGAGATTGAGAATTCAAATAAGGTTAGAATCGGTAAAGAGATTATAGCAAAGTTGAAAGGTTCTCCTCTTACAATAA
GAATAGTTGGACGTTTGTTATATTTCAAAAACACAGAAATGGATTGGTTGTCATTCAAGGACAACGATCTTGGCACAATT
TTGCAACAAGAAAATCAAATTCAACCAATATTGAAGATAAGTTTCAACCACCTTCCATCTAATTTGAAGCACTGTTTTAC
ATATTGTGCATTGTTTCCGAAAGACTATGAGTTTCAAAAGGATGGATTGGTAAAGCAATGGATGGCACAAGGTTTCATTC
AATCACATAGTAATAAAGAAATTGAAGATGTTGGTGATGATTATTTTAAAGAGTTGTTGGGCAGGTCATTCTTTCACAAT
GTAAAAGTAAATAAATGGGGAGACGTCAAGGAGTGCAAGATGCATGATTTGATACATGATCTTGCATGTTGGATAGTAGA
AAATGAATGTGTGGATGCAAGTGATAAAACTAAGTCAATTGATAAAAGGACTCGACATGTGTCATTTCCCTCCAATTATT
CAAGGAAAAGTTGGGAACTTGAAGCAAAATCATTGACTGAGGTAAAGAATTTGAGAACTTTGCATGGTCCTCCATTTCTT
CTATCTGAAAATCATTTGCGATTACGGTCATTGAATTTGGGGTACAGCAAATTTCAGAAAATTCCCAAGTTCATTAGTCA
GTTAAGACATTTAAGATATCTTGACATTTCTGATCATGATATGAAATTTCTCCCAAAGTTTATTACGAAATTGTATAATC
TGGAAACACTTATCCTTCGTCACTGCAGTGATCTAAGAGAATTGCCAACCGATATTAACAATTTGATAAATCTTAAGCAT
CTTGATGTACATGGTTGCTACCGTTTGACTCACATGCCAAAAGGACTGGGTGGGTTGACTAGCCTTCAAACGATGAATTT
GTTTGTATTAGGGAAGGATAAGGGTTGTGATTTAAGTGAGTTGAATGAACTTGCTAGGTTAAGAGGATCCTTGCTTATTA
AAGGATTAGAACTTTGTACCACTACTGATTTGAAAAATGCTAAATATATGGAAGAGAAATTTGGAATTCAGAAGCTGAAA
CTACGTTGGAATAGGGATCTGTACGACGCTGAAACTGACTATGCATCAGAAAACGATGATGAGAGAGTTCTAGACTGCTT
AAAACCACATTCAAATGTCCACAAAATGCAGATAAGAGGATATAGAGGAGTAAAGTTGTGTAATTGGCTATCTTTTGATT
ATTTAGGCGGTTTGGTCAACATAGAGCTTCAAAGTTGTGAAAAGTTGCAGCATCTCCCTCAATTTGATCAATTTCCTTTT
CTCAAGCATCTTCTTCTTGAAAACTTACCCAGTATTGAGTACATTGATAATAACAATTCTCTTTCTTCATCAACTTTCTT
TCCATCCCTTGAGAAGCTAACCATCATGACAATGCCTAACTTGAAAGGATGGTGGAAGGGGGAAACCCCCCCGGAATCTG
CTCGTTACAGTGCCTTGTTTCCAACAATATTACATCACCTTTCTCGATTAGATATTTCTAATTGTCCTCAGTTGGCTTCT

ATTCCACAGCATCCACCTTTGCGATCATTAGCATTGAATGATGTTAGTGTGCAACTTTTTGATATGGTAATAAAAATGGC
TACAACCCCTGCTGCTGATTCTTCTTCAGCTTTGTCTAAATTATCTATTCTTCACATTCAGAATATTGATCTTGAGTTTC
TGCCAGAGGAGTTGTTTGGTAGTACGACAGATCTTGAGATTTTTACCGTAGAGTTGAAATATATGACAACTCTTGAACGT
TTGGATCTATATAATTGTCCTAATATAGTGAGTCTTGAAGGGATTAGCCACCTCACTTCTCTGTCAAGCTTGAGAATTTG
TAATTGTAGCAATTTAACTTCGTTGCCAGAAGGGATCAGCCATCTCACTTCACTATCATATTTGACGATTTATTGTGTCA
ATTTAACTTCGTTGCCAGAAGGAGTCAGCCATCTCACTTCATTATCAAGTTTCACTATTGAGGAATGTCCCTGTTTAACT
TCATTGCCAGAAGGGGTCAGTCACCTCACTTCACTGTCAACTTTGATAATTAGGCGTTGTGTCAATTTAACTTCGTTACC
CGAAGGGATCGGCCATCTCACTTCACTGTCAATTTTCACTATTGAGGAATGTCTCAATTTAACTTCGTTGCCAGAAGGAC
TTTTACACCTGTCCTCCTTGCGAGGATCATTGACAGTTTCAAAATGCCCCAAGTTATCGAAGACGTGGAAGAAGCTAAAC
AAGTAG

>Cucsa.128130
ATGGCAATTGGTGACCCTCAACTTCCTATCCTACATCGTGCACCTGTTGATAAAAGCCATGGCATAAAAAAGCTTAGCTC
TCATGCTTTAGAATGCCTTGGAATGGTATGTGGTCTTAATGATGATCTTAACAAACTAAGGAGCAATGTTTCGTCCATTC
AATCTGTACTTCGTGATGCAGAGCAACGTCAAATCAAAGGCAATGATCATTCTTTAACCGATTGGCTCGAAAAGTTGGGA
GACGTTTTTTACGATGTTGAGGACGTTCTTGATGAAATCTCTACTGAGGCTCTCCGTCGAGAAGTGATGACAAGAGGAAA
AAATGCAAAGCAGGTTAGAATCTTCTTCTCCAATTCTAACCAACTTGCATTTAACTATAGGATGGCATGTCAAGTCAAGA
AAATTAATGAGAGGCTTGATGTTATTTCCCAAGAAAAAGATAAGTTTCAGCTCAATGGAATTGCTTATCTTGGGATACAA
AATGTTTTATCTTATCCAATTGGGATGGAAAGGGATACTCACTCATCTTTAAGTGGGGATCAGAAAATAATTGGAAGGGA
TGATGAAATGAACAACCTTAAAAAAAATTTACTAGCAGAGGATGACAAGGTGAAAGCTAACGTTTCATTCATCGCTATTG
TTGGAATGGGTGGAATTGGCAAGACAACTTTGGCCAAATCTCTCTACAATGACAAACAAGTCTCTGATGGTTTTAGTTCA
AGAATTTGGATTTGGGTTTCTAATCAATTTGACACAAAAACAATATTGAAAAAGATAATTGAATCAGCAACCGAAAAGAA
ACCAAAGGTAGAAGAAATGGAACCTTTAAAGACAAAGCTTGAAGAAGTGATTGGAGGAAAGAAGTATTTGTTAGTTATGG
ATGATGTATGGAATGAAAATGAAAATGAATGGGAGAATTTGAAAAACCTGTTAATGCTTGGTGCAAGAGGGAGTAAGGTT
TTGATCACAAAGCGTGACAGTAAAGCAGTTCCAGGAGTCGAAACAATTCCTCTAAAAGACTTAACTGAAGATTTTTCTTG
GTTGTTGTTTAAAGAAGTGGCATTTAAAGAAAGTGACTTAGAGTCAATAAATCAAAACTTGATAAAAATGGGAAAAGAAA
TTTCAAAAAGATGTGGAGGTATTCCTCTTGTAATAAGACACATAGGACGTTTATTATATGGAAAAACTTCCGCAGAAGAT
TGGGAGTTCATCAAAGAAAATGAACTTTTAAATGTCACTCGTGAAAAAAATAATAATGATGGTCATGTGATATCAACATT
AAAATTGAGCTATAACCATTTGTCACCAAATTTGAAGCAATGTTTTTCCTATTCATCCTTGTTTCCCAAAGGATACAAAA
TTAGAATGAATGAATTGATTAGACAATGGATAGCTCAAGGTTTTATCGAATCATCAAATGGAGGAAAATCTGTAGAGAAT
ATTGGGAAGGAGTACTTGGATGAATTATGTTGGAGGTTTTTCTATGAAATTTCTATTGAGGATGTTCCTTTTGAAGAAGT
TGGCATGCATGATTTGATGTGTGATCTTGCAAGAGAGGTAGCTGGACAGAAATTGTACATACGTGGATATCCAGAGAGTG
GATATGTTGTGAGTGAACAAACTCGTCATATTTCATTTGAATATGAACCACGATCATGGATTGATGATGTGTCCAAATTG
CAACAAGCTAAAGGATTAAGAACGTTCCTTTTGTTTACGAAAAATCCTTTCTTTACGAGAAATCCAATTGAAAAAGTTCT
TTTGGACAGACTGTTTTCTCACTTTCCACGTTTGCGAGTATTACAAATCCCTAATGTGTCAAAGTCAATAAAAAAGCTTA
GACATCTTCGATATCTAGAACTCGGTGAAGATGCGAAATCAGTTCCAAACTCCATCACGAAATTGCAAAATTTGCAAACA
CTAGATCTAACCAAGTGTTATGACCTAAAGGAGTTGCCAAGGGATATTAACAATTTTGTAAACCTCAGACATCTTCTTTG
TGATTCAAGATTAATGAATATGCTGCAAGGGACGATGGAGAAGTTGACTAGTCTACAAACATTAAGTTCATTTTTGTTTG
ATTGTAAAAGGTTTGATAAGGTAAAGGAATTCAGTGAGCGGAGTTATTTTATAGAATTTGACTTAAAAATCAAAGGTTTG
GAGCAGTTGAGGTTTTCTCCATCAGACGTCAAATCAGTAAATCTTAAAAACAAAAAAGTCCCACTTTTGAGACTGAAATG
GAAATTTGAGAATGGTAATGAATATGAAGGTGATGCTGATGATATAGTATTGGAAGGCTTAGAACCACATCCATATGTTA
ATCTCTTGCAAATTGAAGGGTATTGTGGAGTAGGATTACCCAATTGGGTGTCCACCTCAATTTTGTTAAGGGGAATTCGA
ATTGGTAATTGTGATAGATTACATCTGAATCAACTCTCCCATCTTCATGCTCTTGAAATTCTAAATTTAGAGGGTTTAAA
ATCTGTCATGAGTATATCGGAATGGATTGGCACCCTTACTTCTCTTGTATCTTTGGAAATAGAAGAATGTCCAAAATTAA
AATCACTTCCAAAGGAAATGCAACAGCTCAAATCTTTGGTGCAACTTAACATAATCAAGTGCCCACAACTTGGGGAGAGA
TGCAAGGAGGGAGGAGAGGATTGGCCTAACATTTCCCATATTCCTGACGTTCTTATTGATTGA

>Cucsa.128140
ATGGCTGAAGCTATTCTCTACAACGTTACTGCAGACATCATATTCAAATTGGGCTCTTCCGCACTACAGGAGCTTGGGTT
GTTGTGGGGTGTCAATGATGAACTCGACAAACTCAAACACTCGCTTTCTGCCATTCAAGCTGTGCTTCTCGATGCGGAGG
AGCAGCAGTCCAAGAGCCTTGCTGTCAAGGCTTGGGTTTCAAGGCTTAAGGATGCTTTGTACGAGATTGATGACCTGGTG
GACGAGTCCTCCTACGAAACCTTAAGAAGGCAGGTTTTGGCCAAAGATCAGAGAAAAAGAAAACTAGTACGTATCCTCTT
TTCCAAATTTAAATCTAATTGGAAAATAGATCACAAAATCAAGGATATTAGACAGAGGCTACAATCTATTAATGATGACA
AAAATCAATTTAGCTTTTCTGAGCATGTGATCGAGAAAAGAGATGATGAAGAGTTGAGAAAGAGACGGGAGACTTACTCT
TACATACTTGAAGAGGAAGTGATCGGTAGGAATGATGACAAGGAAGTAGTCATAGATCTTCTATTAAATTCCAACATCAC
AGAGGATATTGCAATTGTTTCCATTGTTGGAATGGGAGGACTGGGAAAGACTGCCCTTGCTCAGTCTATTTATACCCATC
ACAATATGACTAATAGTGGGTTTGAATTGAAGTTATGGGTGTGTGTTTCTGAAGAATTTGATCTAAAAGTTATTATCCAA
AAGATGATAGAGTCTGCAACTGGGACGAAGCCTAAGCCGTACCTTCAAATAGATTCATTACAAAGTGAGCTTAGAAAGAA
AATCGATGGAAAGAAATACTTATTCGTAATGGATGATGTGTGGAATGAGAAAAAAGAGGAATGGTTACGCCTTAAAAGAT
TATTGATGGGCGGTGCAAAGGGTAGTAGGATTTTGATCACAACACGGAGTGAACAAGTTGCTAAAACTTTTGACTCTACT
TTCATCCATTTTTTACAAATTTTGGATGAGTACAATTCCTGGTTATTGTTTCAAAAAATTACTTGTTTGGAAGGACATCC
AAGTAATCCAGAGAAGCTTGATCAAAGTTCAAGTTTGATACAAATTGGCAGGGAAATCGTTTCAAAGCTAAAAGGTGTTC
CTCTCACGATAAGAACCATCGGAGGACTTTTAAAAGACAATAAATCAAAAAGAGTTTGGTTGTCTTTCAAAGATAATGAA

CTTCATCGAATTTTGGGGCAAGGACAAGATAATCTAAAAGAAGTGCGATTAATTCTTGAACTCAGCTATAAATACCTTCC
AGCTAATTTGAAGCAATGTTTCCTATACTGTGCTTTGTTCCCGAAAGATTATGAAATTAAAACACATGAACTTATACTAA
TGTGGAGTGCGCAAGGTTTCATTCAACCAAATGGCAGCAAGGACAACAGCCTCATTGATATTGGCAATGATTATTTCATG
GAGTTATTATCACGATCATTTTTTCAAGAGGTTACAAAAAATGAACGGGGAGACATAATAGCATGTAAGATGCATGATTT
GATGCATGATCTTGCTTGTTGGATAGCAGATAATGAATGCAATGTCATCAACATAGGAACTCGTCACTTTGCATGGAAAG
ATCAATATTCTCATAAAGATCAACTTCTAAGATCATTATCAAAGGTGACAAATTTGAGAACATTTTTCATGCTAGATTCT
GCAAATGATTTGAAATGGGAATTTACAAAAATACTTCATGATCATTTGCAATTACGAGCCTTGTATTTCAAAAATTTGAA
GAATGCAATGATCGTTTTGGAGTTTACTGGTAAGTTGAAACATTTGAGATATTTGAGTATTATGGACTCATTTATTTTAA
ATCTTCCAGATTCCATTACAGAATTGTATAATTTAGAAACACTGATCCTTCGGAATTCCAGTTTTAAAATGTTGCCCGAT
AATATCGGCAATTTGATCAACCTCAAGCATTTAGATCTTTCTAATAATCGAAATTTAAAATTCCTGCCAGATTCTATTAG
TGACTTGTGTAAATTGGAAGAACTCATCCTTCATGGTTGTTTGAGATTAGAAGAATTCCCAGAAGATACAAAAAAGTTGA
TCAACCTTAAGCATCTTAATGAGAAACACGCAGATGCTCTGTGGAGGGACAAAGAAAAGGGATTTAATGAAGGATTGTGT
AGTTTAATCAAGGCTGTGGAAAAAGACTTCCATTCTGTACACAAGTGTAAGCTCTTCTTTTGTTCAGTCGTTTTCCTTAT
G

>Cucsa.132370
ATGGGTGACCTACTGACTTTTGGTGTGCAAGAAACTTTGAAGCAGGCTGTAACTCTTGTAGCCAAAAAAATTATTGCGTC
AAGTGAATTTAAGGTGGTGCTAGAAGAGCTGAAAGATGATCTACTTCATGCTGAATGGATCCTCCATGCCATAAAAACAA
AGCATGATCATTCACTCAATGACAAAATAACTCATTGGGTGAATGATCTTCAACTTATTGTTTATGAAGCTGAGGATATG
TTAGACTTGTTTGCTTATGACGATGTTGAACGAAAAATAAGATCAAACAAGGTATTTCCTAATTCCTTATGCACCATAAA
ACCCATGCTTGATTGTTTTTCTTTGGTCGTCTTTGTACATCTTGACAATACAACCCGAAAAATAGAGAGTGAAGTTGAGC
AGGTTGAAGAGACAACTTCACTGCTTGAAAATTATGTGGTGGGAAGGGAGATGGAAGTTGAAAGCATAGTTCAAGATGTG
ACTGAGGCTAGTCAACAACAACTCAATTCTATTTTACCCGTTTATGGAACGGGTGGATCAGGAAAAACCACTTTGGCCCA
GTTGGTGTTTAATGACGAGAGGATTGGAAAACAATTTCATCATACTGTTTGGGTATGTGTGTCTCAACCTTTTGTCATCA
ACGAGATCTTGCAGTCAATCTTGAAAAAGGTAAGCAAAAGCAATGATAATCGTAGCAAGGATGATAAGGACACCTTAATT
CGCAATCTTAAAGAAGTGATGGGTGGAAAAAGATATTTTCTTGTGCTTGACAATGTTTGGAATGAAAACAAAATATTCTG
GGAGAAGTTGAAGGAATGCTTAATGAGTATTGTTGAAGAATTAGGAAGCAGTGTCCTTGTCACGACCAGGAGTCGTAAAA
TTGCAGAAATGATGAAAGAAACACTTGACACCTATCATTTAAACAAATTAACTGATGATCAATGTTGGTCAGTATTTAGC
TACTTTGCCAAGGCGAATGCAGTACCAATAACTTCCAATTTGGAGCTTGTGCGAGAAGAGTTAAGCGTGGATCGTCTACC
AAAAGCTTCAATAAAGCAATGCTTTGCTTACTGTTCAAATTTTCCTAAAGGTTATTGGTTTGACAAAAAACAAGTGATCA
AAATGTGGATGGCACACGGGTTTACTCGACCAGATGAAGGAAATAATGAAACAATGGAGGATACAGGAGAGAGGTACTTC
AATATCTTATTGTCTTATTGCTTATTTCAAGATGCTGATGATGACAAATGGCATATTGGTAGGAAGTTTCGTATGCATGA
TCTTATACACGATATTGCTTGTGATGTTTCAAGCGATAAAAGGTTGCAATTAGATCATAGCAGTTCATCAAAGTGGAAAG
GTTTGACAGAAGAGAAAAAAAAAATTGAGAGCAAGTTGCGTACGGTAATAGATTTCGGGAGGAATGGTAAGATTAAAGAT
TTTGTGTGTTTGCGTGTTTTGACAATTGCAGAAAATGTTCGTGAGTTACCAAACTCAATTTCTAAGTTGAAACATCTTAG
ATATCTAGACATTTCACGTTGTTATTCAATAAAGAAGCTTCCAGAATCTATTGTTGGGCATTTGGAAATTCTATTAATGG
GTATTGATTTACCCCCTAAATTTGAAATGCCTCCATATTTGAGCGAATTGGTTCAACTTCAAACGTTGTTTGCTTTTGCA
GTAGGATTTGAGACGGGTCGTAAGATTTCTGAACTTAGGGGTCTTAGAAACTTGAAAGGTTTGTTGAAGCTTCATCGTTT
AGAACATGTTGAAAGTAAAGAGGAAGCCAAGGCTGCAAAATTGGTGGAAAAAGAGAAGGTAGAAGGACTAAACTTGTCGT
GGCGTGGAAAGTGGAAGAATAGACTTGAACCACACAAAAATCTTAAAGATTTGAAAATCCAATCCTTTTTAGGTGGGTGT
TTTCCGAAGGAGACTTTTGTTGAGAATTTAGTAACAATAACTCTACATAAATGTGGAAATTGTGAAAAGCTTCCAATGCT
TGGGCAATTAAGCAAGCTAGAGGCACTTATAATTATATCAAACTTTCCAAAAGTAAAGAGTATAGGCAATGAATTCTATG
GAAATTATAATGACGGCCAAAGCAAGAGTAGTGTAGTATTCCCCAAGTTGAAGGAATTTTATGTTATTGCGATGTACAGC
CTAGTTGAATGGGAAGAAGTGGTAAATAATGTTAAAGCTTTTCCTCGTCTTGAATGTCTGCATATTGTTAAATGTACAAA
ATTAACATCAGCATTAAAAATTGTCTTTTCTTCTTTAATTAATTTTGATCATTCTATTAGTTACTTAAATTTGCTTCCAA
ATTCACTCAAAATCTTATTGTCTTGTTGCTTATTTCAAGATGTTGAAGATGAAAGTGAGATAGGTCAGAAGTTTCTTATG
CATGATCTTATACATGATATTGCTTGTCATGTTTCAAATGATGAAAAATTGCCATCGGATCATAGCCTTTTATCAATGAG
GAAACATTGGACGAACGATGATAAAATAGTTGCGAGCAAGCTACGTACGAATATAGTGAAGAATGAGAATGATTTTGAAG
TGTTGGAAGGACTTGAACAACACAATAATCTGAAATATTTGGAAATCGAATCCTTTTCAGGTGGGCAGTTTCCCAACCAG
ATTTTTGTTGAAAATTTAGTAAAAATAACTCTAATTGAATGTGGAAACTGTGAAAAGCTTCCAATGCTTGGGCAATTAAC
CAAGTACTTAGAAATACTTGTTATTTTTCGATTACGAAAAGTAGAGAGTATAGGCAATGAGTTCTATGGAAACCAAAGGC
GTAGTAGTAGTAGTGTATTCCCCAAGTTGAAGGAATTTTATGTTGATGAGATGGACAGCCTAGTTGAATGGGAAGAAGCT
GTGTCAAATTATAATGTTAAAGCTTTTCCACGACTTGAATGTTTGCATATTATTTCATGCAAGAAATTATTGAAAATTCC
AGATACAAAGGTTCAAATTTGTGGTCTTTTGTTACTACAACTACAAGAGGAGGCCCCTTGGCTCATTTGCTATTCCAACG
TCCGATCATTTTTTTTGAAAGAAGACACGGGTCCTTCATGA

>Cucsa.133510
ATGGCGGGAGCTTTAATTGGTGGCGCGGCATTGGGTGTTCCGTTTAACGAGCTAGCGACCCTCTTGAAGAATTTTGGCGA
GAGGGCGTGGAGTTTCAATTCTGTTCTTAACGAGACCGAATCCAAGGTAAATGATATAATTCCTCTGGTTAAAGAAATAG
ATGGTCTTAATGAATCCCTGGATTATCCAAGAGAAGAAACGGAGAAGTTGAAAAACTTATTAGAATATGCTGGAAAGCTA
CTTAGACGGTGTTTAAGAGTGGGGAAGGCTGATTTGATAAGGAAATCAAGTCATACAGAGAAGCTTCGTGAACTGAATGC
CAGAATCAAAAGTTTCAGTGACGTTGTGTTGTTCCAAACGTCTAGAGACGGGAAGAAGACATTGAGTTTAGTGACTGAGA
TCAAGGAAGTCGTTCGCAGGCTTGATAGCAAATCTGGATTAAGCAATCCGGTGGATTTAGTTGTGACGGTTCCTGTGATT

TCAGAAGAAAGTGTTGGGTTGGAAAAGCCTGTTGAGAAATTGAAGGCCAAACTATTTAGAGATGGGGTTCGATTGTTGGT
AGTGACAGCTCCCGGAGGTTGTGGAAAAAGCACTCTGGCCGAAATTTTTTGTCACGACAAGCAAGTTAAAAATAAATTTC
AGAGAAACATCTTGTTCCTCGTTGTCTCAAGCAAACCAGAAACGAAACGCATCTTAATATCTATAATTCAAAGACTCGGG
GGGCCTATAGAATCTGGTTCTGTAAGTGATGATGAGGCATTCCGGTTGTTAGAAGTTCGGGTGGGGGAATTGAGTCCAAA
TCCTGTATTGATTGTGTTGGACGATGTCTGGGACGGTTCTGAATCAAACAAGCTTCTTGAAAAGTTCTCCCGATTACCCA
ACTGCAAAGTTTTGGTCACTTCTAGATTTAAGTTTCCTGCATTTGGTGAGTCGTATGATTTGGAACCTCTGGACCATAAG
GATGCAATGGAGTTGTTTCGTCGCTGGGCATCGAGGGGTAACAGAGTGCTACAGTTCCCAGATGAAAGAATTGTAGAAAA
GATAGTGAGGGGTTGTAAGAGATTCCCACTTGCTCTGAAAGTGATTGCAGGATCACTTTCGGGTAGAGCCACTTCGGTTT
GGGAAGTTACGGGGAGGAAATTATCTAGAGGAGATTCTATTCTGGGTTCTGAGAAAGAGCTTCAGAAGTGCCTCAAAGAC
ACCTTAGATGCAATCCCAGATGACAAGATAGTTCTCAAGGAGTGTTTCATGGACTTAGGTTCATTTCCTGAAGATCAAAG
AATTCGTGCGGCTACCTTCATTGACATTTGTGCAGTGTTGTATGAACAAGATGAATGTGAAACAATGTCAAACCTTGATG
AGCTCTTCACCCGGACTTTAGTTAACACTGTCTCTTTGAGAAATAAAGCGCATGAAGATGATTACTACAGCGAGTCCTAT
ATTACACAGCATGACGTACTTAGAGAATTGGCTGTCCTTTTGACTAATGAGCAGCCAGTAGACCAAAGAACAAGATTGCT
TGTGGATATTAACAAAAATGAATTTCCCAAATGGTGGTCTGTAAGACAGATGCAACCTGTGAAAGCCCGCCTTTTGTCCA
TAACAACAGATGAGAAGTTCTCATCATGTTGGCCTGATATGGAAGCACCTGAAGTTGAGGTGTTAATTCTAAATCCTGGG
TCAGAAACTTACAAGTTACCTGATTTTGCAAAGAAAATGAACAGATTGAAAGCGCTGATAGTCAGGAATTACAGGTCCTT
TCCAACTGAATTGACAAGTGATTATCAATTAATCAATTGTTTGTCAAGGCTAGAAAGAATCAGTCTTGAGCGGATTTCAA
TATCTTCTTTCATTGACCAGAACCTGAAGCCCCTGTGGCATCTTAAGAAGCTATCGTTCTTTATGTGCAAAATTGACAAA
GCTTTCACAGACTGCTCAACTCAGATCTCATACATGTTGCCTAACTTACTTGAGATCTCCATAGATTTTTGCAACGATTT
GGTGGCTTTCCCTGTCGGACTATGTGAAGTTGTCACATTGGAGAAACTGAGCATTACAAACTGTCATGCATTATCTTCGT
TACCCGAGGAAATTGGGCAGTTGATTAATCTAAAAATTCTAAGGCTTAGATCTTGTATTCATTTGGAGAAGTTGCCAGAA
TCAATCTCAAGGCTCCGGGAATTAGTTTATCTTGACATATCTCATTGTGTTGGCCTTACCAAACTTCCAGATAAGATTGG
CAACTTGCAGAAGTTGGAAAAGCTTAATATGTGGAGTTGCCCGAACATGCGCAAGCTTCCAAAATCAGTAGGAAATCTAA
AAAATTTGAAGGAAGTAGTTTGTGAAAGCGAGATGAAAATATGGGTGAATTTTGTCGCACCTCGGCTTGGCAATGTGGTA
AAAGAACACAAGGAAGAAATCAACTTGGATTTTCTAAATTGA

>Cucsa.155730
ATGGCCATTTCTACAAACCACTCCACTCTCGTCCTCGGCATCTATGGCATGAGCGGCATTGGCAAAACCACTCTCTCTAA
AGCACTCTTCAACCACTTCTTCCACTTCTTCAATTCTAGATCTTTTCTCCCCAACATCAACTCCCTCTCTACCTCCTCTC
CCGACGGTCTCCTTCGACTCCAACAAACTCTCCTCTCCGATCTCCTCATCGCCACAAACCTCCGCTCTCGTTCCTCAACC
ACCACCGACTCCACCGTCGTTCGGATGCAGGAAAGACTCCAAAACAAAAAGGTCTTGGTAGTCCTCGACGACCTGGATCG
TATCGAACAAGCAAATGCACTAGCAATACGGGACCGAAGATGGTTTGGAGACGGAAGCCGAATCATAATCACAACACGAA
ACAAACAAATTTTGGACACTCTAAAAGTCGACGAAGTTTACAACATGGAATCCAATCTACTGAACGACGAGGAATCGTTG
GAGCTTTTTAGCTACCACGCATTCCGGGAGCAAAATCCACCAGAGGAGCTTTTGGAATGTTCGAAATCCATCGTTTCGTA
CTGCGGAAGCCTTCCTCTAGCTCTGGAAATCCTGGGTGGGTCATTCTTCGGAGGGAGACCGATGGAGGAATGGAGATCAG
CGATGGAGAGATTGAAGAGGATTCCGGCGTGGGATTTGCAAGAGAAGCTTCGAATAGGGTTTGAAGGATTGAGAGATGAG
ATGGAGAGGGAGATATTTCTTGATGTGTGTTGCTATTTTGTGGGAATGAAAGAGGAATTGGTAGTGAAGATTATGGATGG
ATGTGGAATGTATGGAGAAAGTGGATTGAGAGGGTTGAAATGGAGGTGTTTGGTTGGTGTTGAGTTTTGGAGTGGGAGGT
TGAAGATGCATGATTTGGTTAGGGACATGGGGAGGGAGATTGTGAGGCAAACATGTGTGAAGGAACCTGCTAGACGGTCC
AGGGTTTGGCTTTATCATGAGGCTCTCAAAATCTTACTCCATCAGAACGGAAGTGAAAACATTGAAGGACTTGCAATAGA
CATGGGTAAAGGAAATAACAAGGAGAAATTCAGATTGGAAGCATTTGGGAAAATGAGAAATCTAAGGTTACTCAAACTCA
ACTATGTGCATCTCATTGGAAGTAATTTTGAGCATATAATAAGCAAAGAATTAAGGTGGATTTGTTGGCATGGATTCCCT
TTGAAGTCTATTCCAAGCTCATTTTATCAAGGAAACCTTGTTGCCATTGACATGAGATATAGCAGCTTGATACACCCTTG
GACTTGGAGGGATTCACAGATTCTTGAGAATCTAAAAGTTCTAAACCTAAGCCACTCTGAAAAGCTAAAGAAGTCCCCAA
ACTTCACAAAGCTCCCAAACCTAGAGCAGCTAAAACTCAAGAATTGCACAGCCTTATCAAGCCTCCACCCCTCCATTGGT
CAACTTTGTAAGCTTCATCTCATCAACCTCCAAAACTGTACAAATCTCTCGTCTTTACCAACCTCCATCTACAACCTCCA
CTCCCTCCAAACTTTCATCATCTCTGGCTGCTCCAAGATCCACTGCCTCCACGACGACCTCGGTCACCTTGAGTCCCTCA
CCACCCTTCTCGCTGACCGAACCGCCATATCCCACATTCCTTTCTCCATTGTCAAGTTGAAGAAACTCACTGACTTGTCT
CTATGTGGTTGTAACTGCAGATCAGGATCGGGAAGCTCGGCATCGCTGCCATGGAGGCTGGTTTCATGGGCATTGCCAAG
ACCAAACCAAACATGCACAGCCCTAACTCTTCCATCTTCATTACAAGGTTTGAGCTCTCTAACAGAGTTGAGTCTACAAA
ATTGCAATCTCGAGTCACTTCCAATTGACATTGGGAGCTTGAGTGAACTAAAGAAGTTGAATCTTGGAGGCAACAAAAAT
TTGAGGGTTTTGGGGACTGAACTTTGTGGACTTTTGAAACTGAATGAGCTGAATGTGGAGAATTGTGGGAGGCTTGAGTT
CATCCAAGAATTTCCAAAGAATATGAGAAGTTTTTGTGCTACCAATTGTAAGTCATTGGTGAGAACTCCTGATGTTTCCA
TGTTTGAAAGAGCACCTAATATGATTCTCACCAATTGTTGTGCATTGCTTGAGGTTTGTGGATTGGACAAATTGGAGTGC
TCTACTAATATTCGTATGGCCGGTTGTTCGAATCTCTCTACTGACTTTAGGATGAGCCTTCTTGAGGTTTTTTCTTCCTC
TTATCTGTTGTTAAATTACAAATTTGGTTTTGCAGTTTTTCACTTATATATAGTATCAATTTAG

>Cucsa.163670
ATGATACCACACGAAATCTTATCACTTTTCATAACCTCTGTCTATGAGTATTTGACAAATATTGCAACCAAATTGGGTTC
TTTAGCACTCCAAGACCTTGGATTGCTGTGGACCGGTATCCATGAGGAGATTGACAAACTCAGAGACACTCTTTCCGCCA
TCCAAGCAGTACTTCACGACGCAGAACAGAAGCAGTACAAGAGTTCTGCTGTGAAGGAATGGGTTTCAAGGCTAAAAGAT
GCTTTCTATGATATGGATGATTTGATGGATGAGTTCTCCTATGAATCCTTTCAAAGACAGGTTATGACCAAACATAGAAC
CAACAACTGTACCAAACAAGTATGTATTTTCTTCTCAAAATCTAATCAAATTAGATTTCGTTTGAAAATGGTTCATAAAA

TAAAAAAGATCAGGGAGAAACTCGATACTATTGATAAGGATAAAACTCAATTCAATCTTTTTGATAATACAAGGGAGATA
CGAAATGATGAAATGACAAAACGATCAGAGACTTGCTCTTTTATACTTGAAGGAGAAGTAATTGGTCGAGATGATGACAA
GAAATGTATTGTACATTTTCTATTGGATACCAACATTATTGCAAAGGAAAATATTGTTGTGGTTGCCATTATTGGAATGG
GAGGATTAGGAAAGACTGCCCTTGCTCAATCTATCTACGGCGATATGAAGGAAAATAAACATTTTGAATTGACAATGTGG
GTGTGTATTTCTGAAGAATTTGATGTCAAAGTAATTGTTGAAAAGATCATAGAATCTCTCACAAAAAAGAGACCTAAGCC
CAACCTTACACTCGATACCTTACAAAGTATGCTACGAGAGAAAATTGATGGAAAAAAATACTTGCTTGTCATGGATGATG
TGTGGAACGATGAACGGACGAAATGGATTAATCTAAAAAAATTTCTTATGGGTGGAGCTAAGGGAAGTAGGATTTTGATC
ACAACTCGTACCCATCAAGTTGCACATATTTTTGACACAGATTTGTTCCATGATTTAAGTGAACTAGACAAGGACAACTC
TTGGGAGTTGTTTAGAAAAATGGCATTTTCCAACGAATCAGAGATGCTTGAGAATTCAAAGTTGGTCGGGATCGGTAAGG
AGATTGTGACAAAGTTGAAAGGTTCTCCTCTTGCAATAAGAGTAATTGGAAGCTATCTGTATTCTAAAAAGTCAGAAAAG
GATTGGTTGTCATTCAAGGAGAACGAACTTGACACAATCATGCAACAGGAAAATGAGATTCAATCCATACTAAAGATTAG
TTTTAACCACCTCTCATCCAGTTTGAAGCAATGTATCACATATTGTGCTTTGTTCCCTAAAGATTTTGAGATTGATAAAG
ATGATTTGATAAAACAATGGATGGGAGAAGGCTTCATTCAACCACATAATAAGAAGGCAATGGAAGATGTTGGTGATGAA
TATTTCAAAGAACTCTTGGGAAGATCATTTTTTCAAGACATAAGTAAAAACCAACTGGGAGAGATCATGAAGTTCAAGAT
GCACGACTTCATGCATGATCTTGCATGTTTTGTTGGAGAAAATGATTATGTGTTTGCTACTGATGACACTAAGTTCATTG
ACAAAAGGACTCGACATTTGTCAATTTCGCCCTTCATCTCAAAGACAAGATGGGAAGTCATTAAAGAATCATTAATAGCG
GCAAAGAATTTGAGAACATTGAACTATGCTTGTCACAATTATGATGGTGATGAAATCGAAATCGACTTCTCTAATCATTT
GCGGTTACGAACATTGAATTTAATATTTTCTACTCATGTTCCCAAGTGTATTGGTAAGATGAAACATTTGAGATATATTA
ATTTTACTCGGTGTTATTTTGATTTCCTTCCCAAGGTAGTTACAAAATTGTACCATTTGGAAACACTTATCTTTCGTGAA
TGTTTCAAGCTAAGAGAACTGCCAAGTGATATTACGAATTTGATCAATCTCAGGCATCTTGGTATTAACTCTTTAATTGA
AGGTTTAAGTTATATGCCAAAAGGAATGGGTTCAATGACTACCCTTCAAACAATGAATTTGTTTATATTGGGAGAGAATG
AAGGTGGTGAGTTAAGTGAACTTAATGGATTGATTAACTTGAGAGGATCATTAAGTATTCAACAATTGCAGTTCTGCAAA
CCCATTGGTATAGAAAATGCTAAACACCTTGAAGAAAAGTCTGGAATTCAAAAGTTAAAATTATATTGGTATCTCTTGGA
AAGGAAATATGAAATTGATGATGAAGATGAGAAAGTTTTAGAGTGCTTGAAACCACATCCAAATCTTCAGAAAATAGTCA
TAAATGGATACGGTGGAGTGAAGCTATGTAATTGGTTCTCATTTGATTATATTGTCAATTTGGTCATTATAGACCTTTTC
AACTGTAATAAATTGCAACAGCTCCCTCGATTTGATCAATTTCCTTTTCTCAAACATCTTAAGCTCCAATATTTACCAAA
TGTTGAGTTTATTGATAATAACGATTCTGTTTCTTCTTCGTTAACAACTTTCTTTCCCTCCCTTGAGAAACTGAGAATCT
TTAGGTTACCTAAGTTGAAAGAATGGTGGAAGAGGAAACTCATCGATCAAACTATTCCACAACATAGACGTTTGGAATCA
TTGAACATAAGTGGTGTTAGTTTGCAAGTTTTTGAGTTGGTAATGGAAATGGCTACTACAAACATTATTGTTGGATCACA
GGATTCTTCTTCTTCAACTACATCTATATCATTATCTTTTCTAAGTATTGAAGACATTGATTTTGAGTTTTTACAATTCC
ATGACTTATTCTCCAATATGACACATCTTAAGTCTCTTTGGATAATAAATTGCAAGAATATAAAAATGTCTTCTTCTCTT
GATGCTGTGACATGGAAAGGACTTGGAAGTCTTCGTGAACTTATGTTGTCCAGCATCCCTGATTTGGAATATTTGCCGAA
GAGTTTGCAATGTGTGACAACTCTTCAAAGTTTGCAAATATATAATTGTCCAAATTTGGTATCTATTGAAAGTATTAGGC
ATCTCACCACTTCACTATCAGTATTGGAAATTCATGGTTGTCCTAATATAACTTTCTACCCTCACGAAATGAGTCAACTC
GCTTCACTAGCTATCACATTTCAGAATCGTGGTTGGTCGGATAATTATGATCCAGGTGAAGGAAGGAAAGAAGATGACGA
TCAGAAACAATTTGGAAGAGATGAACAACATGAAGGAACGACTCATTGA

>Cucsa.178360
ATGGAGTTGTGTGCCGGTGCCATTGTTAATCCAATCGCAGAAAAAATCGCCAACTGCACGGTGGATCCGGTTTTCCGGCA
ACTAGATTATTTGCTCCACTTTAAAACCAATGTGAATGATCTCAAAGATCAAGGCAAGAAGCTGGTGGAAACCAGAGATT
TTGTTCAACATTCTGTCGACTCCGCCAAAACCAATGGGTACGAGATCGAAGTTATGGTCACTGAATGGTTGGGGATAGCT
GATCAATTTAGTGAAGATGTCGATAGGTTTTTCAACGAAGCCGACGGCCGAAGTCTTCGATGGTGGAATATGCTATCACG
CCATCGATTTAGTAGAAGAGCTACCAAATTGGCTGTGGCAGTTGATAAAGCCATTCAAGGTGGGAGTTTCGAGAGAGTTG
GGTTCCGTGTAACTCCACAAGAAATTATGACGCTAAGGAACAATAAGAAGTTCGAAGCCTTTGAATCTAGGGTTTTGATT
CTGAAGGAGATAATTGAAGCGGTTGGCGATGCTAATGCGAGGGTGATTGTGGTACATGGGATGGCGGGAGTTGGGAAAAC
CACCCTAGTTGAAGAAATTGCAAGATTGGCCAAGGAGGGGAAGCTTTTTGATGCTATAGCAATGGTGACTGTAAAGCACA
TTCCAAACATTAAGAAAATACAGGGGGAGATTGCTGATCAATTGGGGTTGAAATTTGAAGAGGAAAAGGAACGAATTAGG
GCCGATCGACTACGTCGAAGGTTAGAGATGGAGAAGAAGGTGTTAGTGGTTTTGGATGATGTTTGGAGTAGGCTTGATTT
GGAAGCTGTTGGAATTTCTAGCCATCACAAGGGATGTAAGATACTTGTAACTTCTAGAAAGGATGATTTGTTTTTCAATG
ATTTTGGTACTCAGAAAAATATATATATCAATATTCTGTCAAAAAAAGAAGCTAGGGATTTTTTCAACAAGGTGGCATGT
GATTCTGTTGAATCTTCTGATGATACTGATCCTGAAATGGAAGCTGTTGCTACTGAATTGGCAGATGAATGTGGAGGATT
GCCACTTTCTCTTGCAACTGTTGGACAAGCCTTGAAAGGTAAAGGGCTTCCAAGTTGGAATGATGCCTTGCAAGGAATGA
AGTTTCCTGGCGAACCCAGTAACTATGGGGTGAATAAAGTGGCATATTTGTCTCTGAAAGTGAGTTATAGATCTCTAAAC
AGAGAAGAAGCCAGATCACTATTCTTACTATGTAGCTTGTTTCCAGAAGATTATCAAATTAACATCAAATACTTGTTGAT
GTATGCCATGGGTTTGGGGTTATTAAACGCCATGAGTTCTCTAGCAATGGCAAAATGGAGAATACTTTCTTTGGTTGATG
AGCTCAAAACTTCTCACTTGTTGCTTGATGGGGTTGATAACGATTTTGTGAAAATGCACGATATAGTTCGAGATACAGCA
ATTTTGATTGCGTCGAAAATGAAGTCCAAGTATTTGGTTAGACATGGTGCTGGAGAGAGTTTGTGGCCCCCAATGGATGA
GTTCAAAGATTACACTGCAATCTCATTAGGTTGCAGTGATCACTCGGAACTCCCAGAATTTATATGTCCACAGCTTAGAT
TCTTATTACTGGTAGGAAAAAGAACATCTTTGCGATTACCTGAAAAGTTCTTTGCAGGTATGCAGGAACTACGAGTTTTA
GATCTCACTGGCTTATGTATTCAGCGGCTTCCACCATCAATCGACCAACTGGTAAATCTTCAAACATTGTGTTTAGATGA
CTGTGTTTTGCCAGACATGTCTGTAGTTGGTGAACTGAAAAAGCTTGAAATTCTTAGCTTGAGAGCATCTGATATTATTG
CACTTCCTAGAGTAATTGGGGAACTTACCAATTTGAAAATGTTGAATTTGTCTGATTGTTCTAAACTCAAGGTGATCCCT
GCTAACCTTTTATCTAGGTTGATAGGGTTGTCTGAGCTATACATGGACAATAGTTTTAAACATTGGAATGTAGGACAGAT

GGAAGGTTATGTTAATGCAAGGATTTCTGAACTAGACAACCTGCCACGGTTGACCACTCTACATGTCCATATTCCAAATC
CCACCATTCTACCACATGCCTTTGTCTTTAGAAAATTGAGTGGTTACAGAATACTAATTGGAGATAGATGGGATTGGTCT
GGCAATTATGAAACTTCAAGGACCTTGAAACTCAAGCTTGATAGTAGCATTCAGAGAGAGGATGCAATTCAAGCACTTCT
AGAGAATATTGAAGATCTGTATTTAGATGAATTAGAAAGTGTCAAGAATATTCTATTCAGTCTAGACTATAAAGGCTTTC
CGAAATTGAAAGGTTTGCGTGTCAAAAACAATGGTGAAATTGTGACTGTTGTCAACTCGGATAACATGCATCATCCACAC
AGTGCCTTTCCATTGTTGGAGTCCTTATTTCTGAAAAATCTAGCTGAACTTGGAAGCATTTGTCGTGGAAAGCTTCCACA
AATGTCCTTCCGTAACTTGAAAAGAGTAAAAGTTGAAAGTTGTGACAGATTAAAATTTGTTTTCCCATCTTCTATGGTCA
GAGGCCTTATACATCTTCAAAGCCTGGAGATTAGTGAATGTGGCATCATAGAAACTATAGTTTCGAAAAACAAAGAAACA
GAAATGCAAATCAATGGTGATAAGTGGGATGAGAACATGATTGAGTTTCCTGAATTGCGTTCTCTGATACTTCAACATCT
ACCAGCCCTTATGGGTTTCTATTGTCATGATTGCATAACTGTGCCTTCAACCAAAGTGGATTCACGTCAAACAGTTTTTA
CTATTGAACCTAGTTTTCATCCACTTCTCAGTCAACAGGTTTCCTTCCCCAAATTGGAGACATTAAAATTACACGCTTTG
AACTCAGGAAAGATATGGCAGGATCAACTTCCTTCTAGCTTTTATGGCTTTAAAAATCTAACTTCTTTGAGTGTGGAGGG
TTGTGCTTCAATAAAATATTTAATGACAATCACTGTGGCTAGAAGCCTTGTGAATCTTGAACGCCTTGAACTAAACGACT
GTAAGTTGATGAAAGCTATAATCATTTCAGAAGATCAAGATCTGGACAACAATTACCCTTCCAAATCTATCTTGCAGAAC
AAGGATGTTTTTGCGAACCTGGAGTCCCTCTTAATCTCTCGCATGGATGCTTTGGAGACATTATGGGTCAATGAAGCTGC
TTCAGGATCCTTTACAAAGCTGAAAAAAGTGGACATCAGAAACTGCAAAAAACTTGAGACAATCTTTCCAAATTACATGC
TTAACAGAGTGACAAATCTCGAGAGATTAAACGTTACAGATTGCAGTTCCCTAGTGGAGATCTTTCAAGTGAAAGTCCCA
GTTAACAATGGCAACCAAGTAAGAGACATTGGAGCTAACCATTTGAAAGAGTTGAAGCTGCTTCGTCTACCTAAACTAAA
GCACATATGGAGCTCAGATCCACACAATTTTTTACGCTATCCATCTCTCCAACTTGTTCATACAATTCATTGTCAAAGCC
TTTTGAATCTCTTCCCTGTATCCATAGCTAAGGATCTCATACAACTTGAAGTGCTTAAAATACAGTTCTGTGGAGTTGAG
GAAATTGTTGCGAAACGAGGAGACGATGGAGATGGAGATGATGCTGCGTCGTTTTTGTTGAGTGGTTTGACATCATTGAC
TCTTTGGAATTTGTTCGAGTTCAAGAGGTTTTATCCTGGGAAATATACTTTGGATTGTCCATCATTGACAGCGCTAGATG
TACGCCATTGCAAATCATTTAAGTTGATGGAAGGAACTTTGGAAAATTCGTCATCAATCTCATCCGCTGTTGAAAAGGTA
TTACATTCTCTCTAA

>Cucsa.178450
ATGAATCAAGCAAGTGGGTCATCTTCTTCCTCACGTTTTAGATGGCATTTCGATGTATTTTTAAGCTTTCGAGGGGAAGA
CACTCGATCCAACTTCACTAGTCATCTTAATATGGCTTTGCGTCAAAGAGGAATCAACGTTTTTATAGATAACAAACTTT
CAAGGGGTGAAGAAATTTCTGCATCTCTTTTGGAAGCTATTGAAGGATCCAAGATCTCCATTGTCATAATCTCTGAAAAT
TATGCTTCTTCCAGGTGGTGTTTGAATGAGCTGGTGAAAATCATTATGTGTAACAAATTGAGAGGACAAGTGGTTTTACC
AATTTTCTACAAAGTGGATCCATCTGAAGTAAGAAAACAAAGTGGAAAATTTGGAGAAGAATTTGCCAAACTTGAAGTTA
GATTCTCGTCGGAGAAGATGCAAGCATGGAGGGAGGCCATGATTTCTGTTTCTCATATGTCTGGATGGCCGGTTCCTAAG
AAAGATGACGAGGCCAATTTGATTCAAAGAATTGTTCAAGAAGTCTGGAAGAAATTAAATCGTGGAACAAGAGAGATGCG
TGTACCTAAATATCCAGTTGGAATAGATAGACAAGTTAATAATATACTCTCCCAAGTTATGTCTGATGAAATAATTACTA
TGGTTGGATTATATGGAATTGGAGGTATTGGCAAGACAACTTTGGCCAAAGCTTTATACAATAAAATTGCTGATGACTTT
GAAGGTTGTTGCTTTTTGATAAATGTTAGAGAAGCTTCAAATCAATATCGGGGTCTTGTTGAACTCCAAAAGGAGCTACT
TCGTGAGATTCTAATGGATGATTCAATCAAAGTTAGCAATCTCGATATAGGAATTAGCATCATAAGGGATCGACTATGCT
CAAGAAAGATTCTTTTGATTCTTGATGATGTTGATACGAGTGAACAACTAGAAGCATTAGCAGGAGGACATGATTGGTTT
GGACCGGGAAGTGTGGTCATTGCGACAACAAGAAACAAACACTTACTTGCTATTAATGAATTTGATATATTGCAAAGTGT
TCAGGGATTGAATGATGATGAAGCCTTCGAGCTTTTTAGCTGGCATGCTTTTAAGATGAGTTGTCCATCAAGTCATTATT
TATACCTAATTTCAAAACGTGCCGTAAGTTATTGTAAAGGTCTTCCCTTGGCTTTGGAAGTTGTAGGTTCATTCCTTTAT
TCTATTGAGCCATCCAAGCTTAAACTTATATTGGATGAATATGAAAACCAATACCTTGACAAGGGCATCCAAGATCCTCT
TCGAATAAGTTATGATGGACTTGAAGATGAAGTAAAAGAAATTTTTCTTTATATTTCTTGTTGCTTTGTAGGAGAAGACA
TCAACAAAGTTAAAATGAAGTTAGAAGCATGTGGTTGTTTATGTTTGGAAAAAGGAACAACAAAACTCATGAATCTATCA
CTTCTGACCATTGATAAATCCAATCGGGTTGAAATGCATAATTTAATACAACATATGGGTCGCACAATTCATCTTTCGAA
GACTTCTACATCTCATAAAAGAAAAAGATTGTTGATTAAAGATGATGCTATGGATGTCTTAAATGGGAATAAGGAAGCAA
AAGGAGTTAAAGCCATAAAATTAAGTTTTCCTAAAGCTACGGAGTTGGACATTGATTCAAGAGCTTTTGAAAAAGTGAAA
AATGTGGTAGTACTCGAAGTTGGCAATGTCACATCTTCAAAAGGTACTGATCTTGAGTATCTACCTAGTAGCTTAAGGTG
GATGAATTGGCCTCATTTTCCTTTTCCATCTTTGCCTACAACCTACACAATGGAGAACCTTATGGAATTGAAATTGCCAT
ATAGCTCCATCAAACATTTTGGAAGAGGATTCATGAGTGGTGAACGGTTGAAGGAAATTGATCTTAGTGGCTCTGAGTTT
TTAGTGGAAATTGCTGATTTATCTACTGCAACAAACCTTGAAAAGTTGAATCTTTTAGGGTGTGTAAATTTAGTAAAAGT
TCATGATTCTGTGGGATCTCTCACTAAGCTTGTTACATTTTCTCTTTCTAGCAATGTTAAGGGCTTTGAGCAGTTTCCAC
CCCACCTCAAGTTGAAATCCCTCAAACTTTTGTCAATGGAAAATTGTAGAATAGATGAATGGTGTCCTCAATTTAGCGAA
GAAATGAAGTCTAGCCTAGAAGAATTGTTGATTCAATATAGTACTGTAATTAATCAGCTATCTCCAACAATTGGATATCT
TACTAGCCTAAAACGTTTGTTTATCATAGAGTGCATGAAGCTCAAAACTCTTCCAAGTACAATTTATCGTTTAAGGAATC
TTACTTTTTTAAGTATAACAAATTTGGATTTCTTAGAAACAATGGTTCATGTTGCCCCTGCATTGAAACTGTTGGACTTA
TCTGGAAACAATTTTTGTAGACTACCCTCATGTATTATTAATTTTAAATCCTTGAAATCTCTTGTTACAATGGAATGCAA
GTTGCTTGAAGAAATTCCAAAGGTTCCAAAAGGAGTAGTTCGTATGAATGCTACAGGGTGTATATCATTAACCAGATTTC
CTGACAACATACCTGACTTCATATGCTGCGATGATAATGTGGTGCGTATCATTGTTCTTTCTCATGACCTCATGATCTCT
CGTGTATTTCGTTCATATAAGAATTAA

>Cucsa.178620
ATGGCTGAAGCTATTCTCTTCAACCTTACTGCAGACATCATATTCAAACTGGGTTCTTCAGCACTCCGACAGTTTGGATC

TCTACGGGGCGGTGTCAAGGATGATTTTGACAAACTCTGGCACTCTCTTTCTGCCATCCAAGCTGTTCTTCACGACGCGG
AGGAGAAGCAGTTCAAGGACCATGCGGTCGAAGTTTGGGTTTCAAGGCTTAAGGATGTTTTGTACGAGATTGATGACTTG
ATCGACGAGTTCTCTTACCAAATCTTGAGAAGGCAAGTTCTGCGAAGTAACAGAAAACAAGTACGTACCCTCTTCTCCAA
ATTTATAACTAATTGGAAAATAGGCCACAAAATCAAGGAAATCAGTCAGAGGCTACAAAATATTAATGAAGATAAAATTC
AATTTAGCTTTTGTAAGCATGTGATAGAGAGAAGAGATGATGATGATGAAGGGTTGAGAAAGAGACGGGAGACTCACTCT
TTTATACTTGAAGATGAAGTGATTGGTAGGAATGATGACAAGGAAGCAGTCATAGATCTTCTGCTAAATTCCAACACCAA
AGAGGATATTGCAATTGTTTCCATTGTTGGAATGCCAGGATTTGGAAAAACTGCCCTTGCCCAATCTATTTATAACCATA
AGAGGATAATGACTCAATTTCAGTTGAAAATATGGGTGTGTGTTTCTGACGAATTTGATCTGAAAATTACTATCCAAAAG
ATAATAGAGTCTGCAACCGGGAAGAAGCCTAAATCATTCCTTCAAATGGATCCATTACAATGTGAGCTTAGAAAGCAAAT
TGATGGAAAGAAATATTTGATCGTCATGGATGATGTGTGGAATGAGAAAAAAGAGAAATGGTTACATCTGAAAAGATTGT
TGATGGGCGGTGCAAAGGGTAGTAGGATTTTGATCACAACACGCAGTGAACAAGTTGCAAAAACTTTTGACTCTACTTTC
GTTCATCTATTACAAATTTTGGATGCATCCAATTCTTGGTTATTGTTTCAAAAGATGATTGGTTTAGAAGAACATTCAGA
TAATCAAGAGGTCGAGCTTGATCAAAAGAATTCAAATTTGATCCAAATCGGCATGGAGATTGTGTCAACGTTAAGAGGTG
TTCCGCTTTTAATAAGAACCATTGGAGGACTTTTAAAAGATAATAAATCAGAAAGATTTTGGTTGTCTTTTAAGGATAAG
GAACTTTATCAAGTCTTGGGACGAGGACAAGATGCTCTGAAAGAAATACAATTGTTTCTTGAGCTTAGTTATAAATATCT
CCCATCGTCTAACTTGAAACAATGTTTCCTATATTGTGCTTTGTTCCCCAAAGATTATCGAATTAAAAAGGATGAACTTA
TATTACTATGGAGAGCACAAGGTTTCATTCAACAAAATGGCAACAACGACGACAATAGTTCCCTCGTTGATATTGGTGAA
GATTATTTCATGGAGTTATTATCAAGGTCGTTTTTTCAAGAGGTTGAAAAAAATGATTTTGGAGATATAATAACATGTAA
GATGCATGATTTGATGCACGATCTTGCTTGTTCGATAACAAATAATGAATGTGTGCGTGGACTGAAGGGAAATGTCATCG
ACAAAAGAACTCATCACCTTTCTTTTGAAAAAGTTAGTCATGAAGATCAACTTATGGGATCATTATCTAAGGCAACTCAT
TTGAGGACACTTTTTAGTCAAGATGTTCATTCACGATGTAACTTGGAAGAAACCTTCCACAATATTTTCCAATTGCGAAC
ATTGCACTTAAACTCGTATGGTCCACCCAAATGTGCAAAGACTTTGGAGTTTATTAAATTCCTTCCCGATTCTATTACAA
AATTGTATAAGTTGGAAGCACTTATACTTGACGGTTGTTCCAATTTGAAAGAATTGCCAAAATATACTAAAAGGTTGATC
AACCTTAAGCGTCTTGTTTTGTACGGATGTTCGGCTCTCACTCATATGCCAAAAGGATTAAGTGAGATGACTAATCTTCA
AACATTGACTACATTTGTATTGGGAAAGAATATTGGTGGTGAGTTAAAGGAGTTGGAAGGACTTACTAAATTAAGGGGAG
GATTAAGCATTAAACATTTGGAATCTTGTACCAGCATTGTTGATCAACAAATGAAGAGTAAGAACAGTAAGTTCTTGCAA
CTAAAGTCTGGTCTTCAAAACTTGGAGTTACAATGGAAGAAACTGAAAATTGGTGATGATCAGTTGGAGGATGTGATGTA
CGAAAGTGTTTTAGATTGCTTACAACCACATTCAAATCTTAAAGAGATACGTATTGATGGATATGGTGGAGTAAATTTAT
GTAATTGGGTATCCTCTAATAAGTCCCTTGGTTGTCTTGTCACTACATATCTTTATCGTTGTAAAAGATTACGACATCTC
TTCAGATTAGATCAATTTCCTAATCTCAAGTATCTTACGCTTCAAAACTTACCCAACATCGAGTACATGATTGTAGACAA
TGATGATTCAGTTTCTTCATCAACAATTTTTCCATACCTAAAGAAATTTACTATTTCAAAAATGCCTAAGTTGGTGAGCT
GGTGCAAGGATTCAACCTCAACCAAATCTCCTACATATTGGCATGCACCTAAGTTGAAACTCTTGCAAATTAGTGATTCA
GAGGATGAGTTGAATGTTGTACCGTTGAAAATTTATGAAAACCTCACCTTTCTATTTCTTCACAATTTGAGTAGAGTGGA
GTACTTGCCCGAGTGTTGGCAACATTATATGACATCTCTACAACTTCTTTGTTTAAGCAAATGCAACAATTTAAAGAGCT
TGCCGGGTTGGATTCGCAACCTTACCTCTCTTACAAATTTGAATATTTCTTATTGCGAAAAGCTAGCTTTTCTACCAGAA
GGGATTCAACACGTCCATAATTTACAATCGATAGCAGTTGTTGATTGTCCTATATTGAAAGAATGGTGCAAGAAAAACAG
ACGAGAAGATTGGCCTAAGATCAAGTACTATATTTCCGAACATATTTGGGAGAATATTTGTAGCCTCACAGGTTCTTGGT
CATCTAGAAACAAGATTTTTTCTGATCATTTCCGCTCTGTAATTGCTCTAAACTGTAGTGCATGCTTTTACTTGATTTAT
ATTTGTCGCTGCTACTATGAGCTTATCTCTAGTCCTTTTTTTATCATCTTGTATCATGTGCTTTTCAATGAATGA

>Cucsa.189390
ATGGATATAATTTCTCCTGTCGTTGGACCAATTGTGGAGTACACTTTAAAGCCTATTGGTCGTCAATTGAGTTATCTATT
CTTCATTCGCCAACATATTCAAAACCTTGAGAGTCAAGTTGAATTGTTGAAGAACACTAAAGAATCGGTGGTTAACAAGG
TTAATGAAGCGATAAGAAATGCTGAAAAGATAGAATCCGGTGTTCAAAGTTGGTTGACTAAGGTGGATTCCATCATTGAA
AGATCTGAAACGTTACTAAAGAATCTTTCTGAGCAAGGTGGATTGTGCTTGAATTTGGTCCAGAGACACCAATTAAGTAG
GAAAGCTGTAAAGTTGGCTGAGGAGGTTGTTGTGATAAAAATTGAGGGGAATTTCGATAAAGTCTCCTCTCCTGTAGCTC
TTTCAGAGGTTGAGAGTTCAAAGGCAAAGAATTCTGATTTTGTCGACTTTGAATCAAGAAAGCCAACTATTGACAAAATC
ATTGCTGCACTTATGGATGATAATGTCCACACAATTGGAGTGTACGGGATGGGAGGTGTTGGCAAAACAATGCTAGTCCA
AGAGATTTCAAAATTAGCTATGGAGCAAAAGCTATTTGATGAAGTAATCACATCAACTGTTAGTCAAACGCCAGACTTAA
GAAGAATTCAAGGACAACTTGGTGATAAGCTTGGACTCCGATTTGAACAAGAAACAGAAGAAGGAAGGGCTCTTAAGTTA
CTAAATAGGTTGAAGATGGAACGTCAAAAGATCCTCATTGTACTTGATGATGTTTGGAAGCAAATTGACTTGGAAAAAAT
AGGAATTCCAAGCATTGAAGATCACAGTGGATGCAAGATCCTATTTACCTCTAGAGATAATGATGTTCTCTTTAATGATT
GGCGCACATATAAAAATTTTGAGATAAAATTTTTACAAGAGGACGAGACGTGGAATTTATTCAGGAAAATGGCTGGTGAG
ATTGTTGAAACATCTGATTTTAAGAGTATAGCTGTTGAAATAGTAAGGGAATGCGCACATTTGCCCATTGCTATTACTAC
AATCGCTAGGGCATTGAGAAATAAACCTGCATCCATTTGGAAAGATGCCTTAATCCAACTAAGAAATCCTGTCTTTGTGA
ATATTAGAGAAATAAATAAGAAAGTGTATTCTTCCCTAAAGTTAAGTTACGATTACTTAGATTCTGAAGAGGCCAAATCA
CTATTTTTGCTCTGTAGTATGTTCCCAGAAGATTATATCATTGATTGTCAGGTCTTGCATGTATACGCTATGGGCATGGG
TTTATTGCATGGTGTTGAGAGTGTAGCACAAGCACGAAATAGGATAACGAAATTAGTTGATGATCTCATATCTTCTTCTT
TGCTTTTAAAAGAATCAAATGTCGATTTGGTTATGTATGTTAAAATGCATGATATAGTTCGTGATGTGGCTATAATAATT
GCATCTAAAGATGATCGTATTTTTACACTAAGCTATTCCAAAGGATTATTGGATGAATCATGGGATGAAAAGAAACTAGT
AGGTAAGCATACTGCAGTGTGCTTAAATGTTAAAGGTTTGCATAACCTTCCCCAAAAGTTAATGCTACCCAAAGTTCAGT
TATTGGTGTTTTGTGGAACTTTATTAGGTGAACATGAGTTGCCAGGAACATTTTTTGAAGAAATGAAAGGGATGCGAGTT
TTGGAAATAAGAAGCATGAAAATGCCCTTATTGTCACCATCACTTTACTCTTTGACAAACCTTCAATCGTTGCATTTGTT

TGATTGTGAATTGGAAAACATAGATGTGATTTGTGAGTTGAACAAACTTGAAAATCTCAGCCTAAAAGGATCACATATCA
TCCAGATCCCTGCAACTATAAGTCAATTGACACAACTAAAAGTATTAGACTTATCAGAATGTTATGCACTAAAGGTAATT
CCGCCTAATATTCTTGTAAATTTGACAAAGTTGGAAGAATTATATTTGCTAAATTTTGATGGTTGGGAAAGCGAAGAATT
GAACCAAGGAAGAAGAAATGCTAGTATATCTGAGCTTAGTTACCTTTCTCAGCTTTGTGCTTTAGCATTACATATTCCAA
GTGAAAAAGTTATGCCAAAAGAGTTGTTTTCAAGGGTTTTCCCGTTTGAGTTGAATGAAAACGAATCTTCATATTTGAAG
TATCTCTACATCAACTATAATTCAAATTTTCAACATTTTATCCATGGACAGAACAAGACTAATTTGCAAAAAGTCTTGTC
CAATATGGAGCGTCTGGAATTGAGCTATTTGGAGAATTTGGAGAGTTTTTTTCATGGTGATATTAAAGATATTTCTTTCA
ACAACTTGAAGGTCATAAAGTTGTTAAGTTGTAATAAATTAGGAAGTCTTTTTTTGGATTCCAACATGAATGGCATGTTA
TTGCATCTTGAGAGGATTAACATTACTGATTGTGAGAAGGTGAAAACAGTTATTTTAATGGAAAGTGGAAACCCATCTGA
CCCTGTTGAATTTACAAATTTGAAGCGTTTAAGGCTAAATGGGTTACCACAACTTCAAAGTTTTTACTCCAAAATTGAAC
AATTGAGTCCTGATCAAGAAGCAGAAAAAGATGAGAGAAGCAGAAATTTCAATGATGGTTTACTTTTTAATGAACAG

>Cucsa.237070
ATGGAAACTGTAATCGCAATTTTAGGGACAGTTTGTGAGTACGCAGTTGCACCCATTGGACGTCAAGTAGGATATGTTTC
TTCCTACAAAAAGAACATCAATGATCTTAAAGACCAACTTCAAAATCTTGTGGATACTAAAACAAGGCTACAACACATGG
TCAATGAGGCAAGAAGTAGTGCGTACAATATCCAAAGTGATGTTTCATCATGGTTGAACCAAGTAGATAAAATCATTGAA
CAATCCAACGACATATTGTACAAGAATGAAAATGAATCAAATAGCAAGTATTGTTCCAATAAGCTTAACTTCATTCATCA
ATATCAAATGAGTAAGAAAGCTAAGAAGATGGTGAAAGTAATTTCACAAATTATAGAGAAAAGAAAGTTGATGTTTCACC
AAGTTGGTTATCCTACACCTCTTTCAAGGATTCATGGAAGTTCTACTAGTAGTTCTCATGGCTATGATCAGATTTTGGAA
TCAAGAACATCAATCGCCAAGCAAATTAGAGATGCACTCGTTGATTGTAACGTGAATAAGGTTGGAGTATATGGTATGGG
AGGTGTTGAAAAAACTACGCTGCTGAAACAAGTCACACCATTAGTGATGGAAGAAAAATTGTTTGATCACGTGATTATAG
TGAATGTAGGTCAAACATTGGGGGTAGAAGGCATACAAGCTCAAATTGGAGATAAGTTAAGGTTGGAATTAAATAAGAAG
GTAGAGAGTAAGGAGGGAAGAGCATCTTTACTACAAAATAAGTTGGAGATGGAAAGTAACGTCCTCTTGGTGTTAGATGA
TTTATGGAAGGGACTCGATCTAGAAGAGGTTGGAATTCCTTGTAGATCAGAGTCATGTGAAAAGGGATGTAAGATACTCA
TAACAAGTCGAGATAGAGATGTCTTAACTAATGAAATGGACACACAAGTTTATTTCGAGGTGAAGCCTTTAAGTGAAAAG
GAATCATGGGAGTTTTTCAAGAACATGATTGGTGAGTTTGATAATAAATGCATAGAACTAATAGGGAAAGAGATGGTGAA
GAAGTGTGGGGGATTGCCAATAGCACTTGCTACAATTGTAAAAACTTTGAAGGGGAAGGAAGTGCCTATTTGGAAGGATG
CTTTGAAGCAATTGAAAAATCCTATTGCAGTGGATGTTAAAGGGGTGACTGATGTATTCCCAGATGATTATGAGATTTCT
GTGGAAGACTTGCAAATATATGCCATGAGTTTGAGATTGTTAAACCAAGTGAATACCTGGGACGAAGCTAGAAACAGGGT
AATTAAATTGGTTGATGATCTTAAAGCTTCTTCTTTACTTCTAGAATCCAATTCAAGGGATAATCATGTTAAAATGCATG
ATATCGTTCGTGATGTGGCAATATACATTGCATCAAAGGAAGCTAATATGTCTACATTGAGCTATGGTTTCGGGCTGAGT
GAATGGCAAGAAAAGGATAGACATGGATTCTATAGAGCAATCTTTGGAAACTGTCACAACTTCTACAACTTTCCTCAAAA
CTTAGAGTTTCCAAAACTTGAATTGTTGATATTAGATGGACATGATTGGAGGGGAGAAAAACTTCAAATTTGTTACTCTT
TTTTTGAAGGAATGAAAGAACTTAAAGTTTTGAACTTGTCAAGGATGTGTTTTCAACTACTAAGGAGGCCATCAATCCAC
TCTCTAGAAAATCTTCAAACATTATGCATGTCACATTGTACATTCAATGACATTGATGCAATAAGTCACCTAAAGAAGCT
ACAAATTTTGAGGATTGATAAATGTCCAATCACATTGTTACCTAAAAGCATGAGTCAATTGACACAACTTAAGGTACTAC
AAGTGTCAAATTGCCCTTTGAAAGTGATTCCTCCAAACACCCTTTCAAGCCTTTTGAAGTTACAAGCACTAGATATATGG
ACAAGCTTCAATGGATGGGGAGAAGAAGTATCACACAATAACAAATTGATCAATAATGCAAGGCTATCAGAGTTGAAGTG
TCTACCACATCTAACAAATTTAAAGATACATATCTTGGACATCAAAATTCTTTCAGATCTAATATTTCTGAAAAATTTGA
AGCTAGAAAGATTTGTTATCCATGTTGGTGAATTGAAAATGTCCCAAAGGTTGCAAGGATGTGAACAATATGCAACAACT
TTGATGCTTAAAATCATAACATCATCATCCCAAATTGTTTCAATCGATCATCATGAGTTCTTGTCCCTTGAAAAAATGGA
GAGCTTGGAGAATATAGTTCATGCAGATGTGTTTACAAGTCCTTTCAGAAAGTTGAGATCCATAAAAGTAATAAGTTGTA
AGAGATTAAGGTATCTCTTCTCTTTCTCTATCTTTAAAGGCCTTGTGGATCTACAAAGGGTTTTTATATTTGATTGCAAC
ATGATGGATGAGATACTTTGCATGGATAGTGAAGACTCAACAATTGCAGTTGAAGGCAATTCTATTGAGTGTCCTCAATT
GAAGGATTTGACAATCATTGGAGCACATAATTTGAAGATGTTATGGCATAAAAATGGGTTGGCTCCAAATTTCTTCAGCA
AACTCCAAAGGATAAGTATCAATAGTTGCAACACTTTAAGATACCGTGGG

>Cucsa.237390
ATGGTCCCTTCTTCTCCTTCTTCTTCTGCTGCTTCTTCTTCTTCTCCTTCTTCTTCTCCTTCAATTGGTAAATGGAAATT
TGATGTGTTCTTGAGCTTTCGAGGCGAAGATACACGTGGTGGCTTCACGGATCATCTCTACAAAGCCTTAACACGAAAGG
GAATTTCAACATTTAGAGATGAAAATGAGATCGAAGAAGGTGAACACATTCCTTCAAATCTGCTGGCTTCCATTGATGCC
TCGAGATTTGCCATTGTTGTGGTTTCGGAAGATTATGCATCTTCAAGATGGTGCCTCGAGGAATTGGCTAGGATGTTTGA
ATGTAAAAAAGAAGTTTTACCAATTTTTTATAAAGTGGATCCCTCTCATGTGAAAAACCAAAGTGGAACGTTTGAAGAAG
CTTTTGTTAAACATGAAAAGAGATTTGGAAGAGGTGATGGGAAGGTTCAAAGTTGGAGGACGTTTCTCACCGAGCTTGCT
AACACCAAAGCTTGGCTTTCTCAATCTTGGTCACATGAATCAAATATCATTGAAGAAATCACCACAAAAAATATGGAAAAG
ATTGAAACCCAATTTGACAGTCATTAAGGAAGACCAACTAGTTGGAATTAATTCTAAAATAAACAAACTTTCTTCACTTT
TGATTCCAAACTCAGATGATGATGATGCTGATGATGATGTGATCTTTGTGGGAATACATGGAATGGGTGGCATTGGTAAG
ACCACAATAGCTAGGGTCTGTTATGAGCGAATTCGTGACGAATTTGAAGCTCATTGCTTCCTCTCCAACGTTCGAGAGAA
TTATATCAGAACCCTTGGGAACCTTTCATGTTTACAAACCAAACTCCTTTCAAGCATGTTTTCGCTTAAAAACAATCACA
TAATGGATGTTGAAGAAGGTACCGCTATGATCAATAAAGCCATTTTTCGAAAAAAGACACTTCTCGTCCTTGACGACGTG
GATTCTTCGGATCAAATCAAAGGATTGATTCCAGACAACAACTCTTTTGGCAATGGAAGTAGAGTCATCATCACAACACG
GAATGCGGATTTTCTTTCGAATGAATTTGGGGTGAAAAGAATTTTTGAAATGGATGAACTTAAATATGAGGAAGCTCTTC
AACTTCTTAGTTTGAGTGCTTTTATGAAAACATGTCCAAAAGAAGGTTACTTGGAACACTCCAAGAAGATTGTAAAGGTT

GTGGGAGGCCACCCTCTTGCACTCAAATTGTTAGGGTCGTCTCTAAGAAACAAAAATTTGAGTGTGTGGAATGAGGTGAT
AGAAGAGGTTGGAGGAGGTGGGAATATTCATGAAAAAATTTTCAAGTGTCTTAAAGTGAGTTATGATGGGTTGGATGAAA
GGGAGAGAGAGATATTTCTTGACGTTGCTTGCTTCTTCAATGGGAAGAGAAGAGAAGTTGTAGAAGAGATATTAAATGGA
TGTGGTTTCTATGCCAAAACAAGGATTGAACTTCTTATTCAAAAGTCTCTCTTAACTCTTTCTTATGACAATAAGTTACA
TATGCATAATTTATTGCAAGAAATGGGTCGAAAGATTGTTCGGGATAAGCATGTTCGAGATCGATTAATGTGCCACAAAG
ATATAAAAAGTGTGGTGACAGAGGCATTGATCCAAAGCATATTTTTCAAATCAAGTTCAAAGAATATGGTGGAATTTCCA
ATTTTGTTTTCAAGAATGCACCAACTTAGGCTGCTTAATTTTCGCAATGTGAGACTGAAAAACAAGTTGGAATATAGCAT
TCCAAGTGAGTTAAGGTATTTGAAGTGGAAAGGATATCCGTTGGAGTTTCTGCCAATCGATAGCTCTGAAGAATGTAAGC
TTATTGAGCTTCACATGTGCCATAGCAATCTCAAACAATTTTGGCAACAAGAAAAGAATTTGGTGGAGCTGAAGTATATC
AAACTCAATAGTTCTCAAAAGTTGTCCAAAACTCCAAACTTTGCAAACATTCCAAATCTCAAAAGATTAGAGCTTGAAGA
TTGCACAAGTTTAGTCAACATTCATCCATCAATTTTCACTGCAGAAAAACTCATATTCTTGAGTTTGAAAGATTGCATCA
ATCTCACCAATCTTCCTTCTCACATTAACATCAAGGTTCTTGAAGTCTTGATTCTCTCTGGTTGTTCAAAAGTAAAAAAA
GTCCCTGAATTTTCAGGTAACACTAATAGATTACTCCAACTCCATTTGGATGGTACCTCCATATCAAACCTACCTTCATC
AATTGCAAGCTTGAGTCATCTAACAATATTGAGTTTAGCCAACTGCAAAATGTTAATCGACATTTCGAACGCGATTGAGA
TGACATCTCTCCAAAGCTTAGATGTTTCTGGATGTTCGAAGCTTGGAAGTAGAAAAGGAAAGGGGGACAATGTCGAATTG
GGGGAGGTCAACGTGAGAGAAACCACACGAAGAAGAAGAAACGACGACTGTAACAATATTTTCAAAGAAATCTTCCTTTG
GTTATGCAACACTCCAGCTACTGGCATTTTTGGGATCCCATCATTAGCTGGTTTGTACTCTCTTACAAAACTAAACTTGA
AGGATTGCAACCTTGAAGTAATCCCACAAGGGATTGAGTGTATGGTGTCATTGGTAGAGCTCGACTTGAGTGGCAATAAT
TTCTCTCATCTTCCAACAAGCATATCAAGACTTCATAACTTGAAAAGATTGAGGATAAACCAATGCAAAAAGCTTGTACA
TTTCCCAAAGTTACCTCCAAGGATCTTGTTTTTGACGTCAAAGGATTGCATTTCATTGAAAGATTTTATAGATATTTCAA
AAGTTGATAATTTATACATAATGAAAGAAGTGAACCTTTTGAACTGCTACCAGATGGCTAACAACAAAGACTTCCATAGA
TTGATCATTTCTTCGATGCAGAAGATGTTCTTTCGAAAAGGAACATTCAACATCATGATTCCGGGGAGTGAGATTCCCGA
TTGGTTTACAACAAGGAAAATGGGATCTTCGGGATGA

>Cucsa.237450
TATGACGTTTTCCTCAGCCACAGAGCTAAAGACACTGGACGTAGTTTCACGTCCTATCTTCATGAAGCTCTAACAAGTCA
AGGAATTGTAGTTTTCATAGACGAGGAAGATGAAGATAACGGAGGGAAACCGTCAATGGAGAAGACGAAAGCGGTGGATG
AATCGAGGTCTTCGATCGTGGTTTTTTCGGAGAATTATGGGAATTTGGTTTGCATGAAGGAAATAAGGAAGATTAGAATG
TGTCAGAAGTTAGGGGATCAATTGGTCCTTCCAGTATTTTACAAAATAGATCCAGGCGATGTGAGGAAGCAAGAGGGGAG
CTTTGAGAAGTACTTTAATGAACATGAAGTCAATCCTAATATTAGTATTGAAGAGGTTAAAAAATGGAGAAAATCTATGA
ATAAAGTTGGCAATCTCTCCGGATGGCATGTCCAAGATTCCCAGTTAACCTTCAGTGATGACTCTCGTCAATTATGGGTT
TCCTACGAGCCTCGTGAAGTTTACCCTTATAGGTTAAATAAATGGAGAAACCTTCGTGTTTCTTTTCTACCAAGTTGCTC
ACAAACAAAGGTAATATTATGTGGTGCACGTCTTCTTTACAAGGAAGATTTGGATGAGTTTGTAGACACTATTATTGACA
GTGTATTGGGTTGCTCCATAAACCTTCATGAGTTTTATGATGGAGTGTTTTTAAACGGTATGTTGAGTTTGATAAGATCA
CAAAAGTATGATCCAAATATCGAGGAGGAAGAGGAGGAAGAGGAGGACGAGGACGAGGCTTTGATGGAAACTAAAGGAGG
AAATTATGCTTCCACATCTAGCAGAGTTTACAGGTTCTCTCTCTTTTTATCTCTCTCATGCATTTTAATTTTCGAGCAAA
CTACAAAAATTACTCCTAAAATGTATGATAGCCGTAGTTGCAATAATTATAAAAACTCAACCTGA

>Cucsa.237500
ATGAGTACTTTTGATACTTTCATAAGTTTTAGAGGCGAAGATACTCGTAACACGTTCACCGGACATTTGTACAAGGCGCT
GGTTGATTTTGGAATATCGACTTTTATGGACGACAAGAAACTCTTGATTGGAGATAGTCTTAGTGAAGATCTTATTGGAG
CTATAGAAAAATCAGGGTCTTTCATTGTTGTTTTATCAGAGAACTATGCTTCTTCAAAGTGGTGCTTGAGAGAATTGGTG
AAGATAATTGGTTGTATGGTGGAACAAAAGCGTCGAGTTCTTCCTGTATTTTACCACGTGAGTCCTCATGATGTTCGACA
TCAATCAGGGTGTTTCAAGAAAAGCTTTTGTGAATATGAAGAAATTCTTCAAGAGCTCAACGATAGGGAAGGAGATAAAT
ATACGAAGGAGGTTCAAGAATGGAGGAGTGCGTTGACAAAAGTTGGCGAGCTCACTGGAGTAGTTGTAACAAAAGATAGT
CTTGAAGCCGCTAGCATCGACAAAATCACAGAACAACTAAGTTCTACGTTGCATCAACAAAAGTTAGTAAATTTGGACGA
GCTCACTGAGTTAGTTGATATTGAACGTCAGTTATGCAAGATGGATAAGCTAAATGATTTGGAGCCAAATGTGGTACGTT
TTATAGGGATAATAGGGATGGGCGGAATTGGTAAAACAACCATTGCTGAAGTTTTTTATGAAAAAGTTGCATATAAATTT
GGAAAAAATTGTTTTTTGATTGTTCTTGATGGGGTGAAAGAAAAAAGTCAGTTAGAACAGTTAGTTGGAAATCCTAATTG
GTTTGGTCAAGGGTCCAAAATCATCATTACAACCAGAAATAGGGATGTTCTTCGTCAGCCAAATTATAAAGATAAAATGG
TTGAATACAAAGTGGAGTTTCTTGATAATAAAAGTGCCATGACACTCTTTTGCAAACAAGCATTTGGATCATGTGATCAG
TTTCCCAGTAAGAATTTTGAGGACTTTTCTAAGGAGATTGTAGAAAGGGTTAAAGGACATCCACAGGTTTTGAGACAAAT
TGGGTCGTCTTTATATGATAAAGGTATAGAGATATGGAAAGAACAATTGAAGAGTCTTGAGGAAGATTACAACAATCGTA
TATTTAAGACATTAAAGATAAGTTTTGATGATTTAGGAAAGACAAGCCAAGAAGTTTTTCTTGATTTTGCATGCTTCTTC
AATGAGAAGAAGAAAGAGAGTGTGATTGAAATACTTAAGAGTCTTGATTATAGACCTCATAGCGAAATACAATTGTTGGA
AGATAGATGTCTCATTGAAGTAAGACGTGACAACACAATATTTATGCCTAAGTGCATTCAAGCTATGGGTCAACAGATTG
AACGTGAAGCTGATAAACGGAGTAGGATTTGGCTTCCGAAAGATGCCCATGATGTATTTGATGAACCACATAGAGTAAAG
GACATAAAAGGTGTAGTCTTGAAATTGGAAGAGAAGCAAGAAGAAGTAAAGTTAGAGGGTAAGGTTTTTGAGGATATGAG
AAGTTTAAAAATATTGGAAATTGGGAATGTAGAGGTGAGTGGAGACTTCACACATCTCTCAAAACAATTGAGATTGCTCA
ATTGGCAAAGCTATCCCTCACAATGTTTGCCATTACGTTTTGAATCAAGATATTTATTTCAACTTCTTTTGCCTCTAAGT
CAAACAAGGCAACTTTGGAATGGTCAAAAGGGATTTGAGAAATTGAAGGTTATTAATGTTAGCCGTTCGAAGAATTTACG
AGAGACTCCTAACTTTACTAAGGTTCCAAATCTTGAAAGTTTGGACCTAAGTTATTGTCCAAGGTTGTGGAAGATTGATT
CTTCTATTAGTCGTCTCAATCGTTTGACGTTGTTGGATGTATCGTATTGTATCAATCTTGAAAGCTTGCCATTTTCTAGA

AGCTGCAAAAGCCTCGCAAGAATAAATTATGCTGGCTCAGGTCTTGAAGAAAGAGGTATATATACGTACCTTTCATTATG
GTAA

>Cucsa.237520
ATGTGTCGACCTCCATACTGGTGTTGGGATGATTCATGGATGTTCTTTGATGTTGGCTATACAAAGTTCAAAATCCAATC
CATCGCAAATTCAATAGGTGATCACTTGCTTCGTCTTAAGCTCCAAGCCAAAGAAGAAAATTTATTTGAAATGCCACTTC
GATTAAGAACAATGAAAATGCTCCTTGGCTTAGGCTCAAATGACGTACGTTTTATAGGGATAGTGGGGATGAGTGGTATT
GGTAAAACAACCCTTGCGGAAATGACATATTTACGTATTTTTAAGCCTTTTGTATCTGCCTTACGAAAACCTTACTTTCT
TCACTTTGTTGGACGCAGTATAGTCTCCTTGCAGCAACAACTACTTGATCAACTTGCTTTCCTAAAACCTATCGATATCC
AAGTTTTGGATGAGAATCATGGAGTAGAATTGATTATGCAGCATTTGAGTTCACTTAAAAATGTGCTTATTGTTTTTGAT
GGAATAACCGAAAGAAGTCAATTAGAAATGTTAGCTGGCAGCCCCGATTGGTTTGGCGCAGGGAGTCGAATCATTATTAC
AACCACAAATAAAAATATTTTTCATCACCCTAATTTCAAAGACAAAGTGCAAGAATATAATGTAGAATTACTTTCTCATG
AGGCTGCCTTCTCCCTCTTTTGCAAGCTTGCATTTGGAGATCACCCTCATACCCAGAATATGGATGATCTTTGTAATGAG
ATGATCGAAAAGGTTGGAAGACTCCCATTAGCTTTGGAAAAAATAGCTTTTTCATTGTATGGTCAGAACATAGATGTATG
GGAACATACATTGAAGAATTTTCATCAAGTAGTTTATGATAATATTTTCTCTGATGTATTAAAGTCAAGTTATGAAGGAT
TAGAAGCAGAGAGCCAACAAATTTTCCTAGATTTGGCATGTTTCCTCAATGGAGAGAAGGTGGATAGAGTGATTCAAATA
CTTCAAGGCTTTGGTTATACCTCACCTCAAACTAATTTGCAATTGTTGGTTGATAGATGTCTTATTGATATTTTAGACGG
ACATATACAAATGCACATCTTGATTCTTTGTATGGGCCAAGAAATTGTGCACCGCGAGCTGGGAAATTGTCAACAAACAA
GGATTTGGCTTCGAGATGATGCTCGTCGTCTATTTCATGAAAACAATGAATTAAAATATATTCGTGGAATAGTGATGGAC
TTAGAGGAGGAAGAAGAATTGGTATTGAAGGCTAAGGCATTTGCAGATATGTCTGAGCTAAGAATTTTACGAATCAACAA
TGTGCAACTTTCGGAAGATATTGAATGTCTGTCAAATAAATTGACGTTGCTCAACTGGCCTGGCTATCCTTCAAAGTATT
TGCCATCAACTTTTCAACCACCATCTCTGCTTGAGTTACACTTGCCTGGTAGTAATGTTGAACGACTCTGGAATGGAACA
CAGAATTTTAAGAACTTAAAGGAGATTGATGCAAGTGATTCGAAGTTTTTGGTTGAAACTCCTAATTTTTCAGAGGCTCC
AAAGCTTCGACGATTGATTTTACGAAATTGTGGAAGACTAAACAAGGTTCATTCTTCAATAAATAGTCTCCATCGTCTAA
TTTTATTGGACATGGAAGGTTGTGTCAGTTTCAGAAGCTTCTCATTTCCTGTCACTTGCAAAAGTCTCAAAACTTTAGTT
CTTTCGAACTGTGGTCTAGAGTTTTTTCCAGAGTTTGGATGTGTGATGGGATATTTGACTGAACTACACATTGATGGGAC
TTCCATAAATAAACTTTCTCCCTCAATTACAAATCTACTTGGCTTGGTTTTATTGAACCTGAGGAATTGTATTAGACTTT
CTAGTCTTCCAACTGAAATTTGTAGGTTGAGTTCACTTAAAACTCTCATTCTGAATGGTTGCAAAAACTTGGACAAAATT
CCACCATGTTTGAGGTATGTAAAGCATCTTGAGGAGCTTGACATTGGCGGAACATCCATAAGCACAATTCCTTTCTTGGA
AAATCTAAGAATTTTGAACTGCGAAAGGCTGAAAAGCAATATTTGGCATTCTTTGGCTGGTTTGGCAGCACAGTATTTAA
GGTCACTCAACGATTTAAATTTAAGTGATTGTAATCTTGTGGATGAAGACATTCCAAATGATCTTGAACTCTTTTCCTCA
TTGGAAATTCTAGATCTGAGCAGCAATCATTTTGAAAGACTGTCAGAAAGTATTAAACAACTTATTAACCTTAAAGTATT
GTACTTGAATGATTGCAACAAGCTAAAGCAAGTACCCAAGCTTCCAAAAAGTATAAAGTATGTGGGAGGAGAGAAGTCCT
TGGGCAT

>Cucsa.237540
ATGCAGAGTTCATCATCGTCTTCTTTGGATCGTCCTAAGATGAACTATGATGTGTTCATAAGCTTTAGAGGTAGAGATGT
TCGTCACACTTTTGCAGGATATTTGTACGATGCTTTGAATCGTTTGGGGATAAAAGCTTTCCTGGACAACAAGAGGTTTC
TAATTGGAGATGATCTTCATGACTTATTCAAAATAATCGATGAATCAAGATCAGCAATTGTTGTTCTTTCAGAAGACTAT
GCTTCTGCTAAATGGTGTTTGAGAGAGTTGACTAAGATAATGGATTCCATGGGAACCTCAATGGAGCGTGTCCTTCCTGT
GTTTTATCATATTGATCCATCAATTGTTAAAGATCAATCTGGAACTTTTAAGACAAGTTTTGATGAACATGAAGCCAATG
TTTTAAAGGAAATTGATAATCAAGAGAAGGAGAAGCGCTTGAAGGAACTCCAGAATTGGAAAAGTGCACTGAAGAAAATT
GGCAATCACACTGGAGTTGTCATCACTAAGAACAGTTCTGAGGTAGATATAGTAAATAAAATTGCAAGTCAAATATTCGA
TGCATGGCGTCCTAAGTTGGAAGCATTGAATAAGAATTTAGTTGGAATGACATCCCGATTGCTCCATATGAACATGCATC
TTGGTTTAGGATTAGACGATGTACGCTTCGTTGCGATAGTAGGAATGGGTGGTATTGGTAAAACAACTATTGCTCAAGTC
GTTTTTGATTGCATTCTTTCAAAGTTTGAAGATTGCTGCTTTCTAACATTACCTGGAGGTGATTCAAAGCAAAGTTTAGT
GTCATTACAACGGGAAATGCTTTCTCAAATTTTTCATAAAGAAGATTTTAGAATATGGCATGAGAATCATGGAGTAGAGA
TGATTAAAAATCGACTGAGTGGTAGAAAGGTTCTTATTGTTCTTGATGGCATCGAAGAGAGAAGGCAGTTAGAAATGTTG
GCTGGAAGCATCGAGTGGTTTGGTCCTGGAAGCAGAATCATCATTACAACTAGAAATAAAGGATTATTGTGCCATCCTAA
TTATGATGAAATGAAAGTATACAATGTTGAAGAACTAGATCATGATAGTGCCCTTCAACTCTTTTTGAAGCATGCATTTG
GTAGTAATCATCAAAACAATGACAGTTTCATGGATCTTAGTAATGAGATAGTTGAGAAGGCTAAAAGACTTCCATTAGCT
TTAAGAGTGATTGGATCTTCTTTGTATGGTAAAGATATTACAGTATGGAGAGAAACGTTGAAGAGGCTGATCAAAGTGGA
TGAAAGAAATTTTTTTGATGTATTGAAAATAAGTTATGATGGATTAGGAGTTGAAAGCCAACAAGTTTTTCTTGACATTA
CATGTTTCTTCAATGGAAAAAATGAAGATAGAGTAATTGAAATATTAGAGAGTTTTGGTTATAGTCCTAATAGTGAAGTA
CAATTACTGATGCAAAGATGTTTAATTGAAGTTTCACACAAGAAAATATTGGTGCATGATTTAATTCTTGAAATGGGTCG
AGAAATTGTGCGTAAGGAGTCCCTCACTCAAGCAGAAAAACAGAGTAGGATTTGGCTTCATGAAGATCTTTACTGCAGGT
TTGCTGAAAAACATGACTTGATGCATATTCAAGGGATAGTTTTAAGTTTGGCAAAAGAAATGGAAGAATCAATAGAATTG
GATGCTGAATCCTTTTCAGAGATGACCAAACTAAGAATACTGGAAATCAGTAATGTGGAGCTCGATGAAGACATTGAATA
TCTCTCTCCACTCTTACGGATAATTAATTGGCTTGGCTATCCTTCGAAGAGTTTGCCCCCAACGTTTCAATCCCGCTATT
TGTTTGAACTACTCTTGCCTCATAGTCACCTTTTACGAATTTGGGATGGAAAAAAGAGATTTCCAAAGCTGAAATTAATT
GATGTTAGTAACTCAGAACACTTGAGGGTGACACCTGATTTTTCTGGGGTTCCAAATCTTGAGAGATTGGTTCTATGTAA
CTGTGTTAGACTGTGTGAGATTCATCCCTCCATCAATTCCCTCAACAAACTCATTTTACTGGATTTAGAGGGTTGTGGTG
ATCTTAAACATTTTCCAGCAAATATAAGATGTAAAAATCTCCAAACACTCAAACTTTCTGGTACAGGTCTTGAAATTTTT

CCAGAGATAGGCCATATGGAACATTTGACTCATCTTCATCTTGATGGATCCAATATAACCCATTTTCATCCTTCAATTGG
GTATCTAACTGGCTTAGTTTTCTTGGACCTATCCTCCTGTTTAGGTCTTTCTAGTCTTCCTTGTGAAATTGGTAACTTGA
AGTCTTTGAAAACCCTCCTTTTGAAATATTGTAAAAAACTTGATAAAATCCCTCCAAGCTTAGCAAATGCAGAATCCTTG
GAGACACTTTCTATTAGTGAAACCTCAATAACCCATGTTCCACCAAGCATTATTCATTGTTTAAAGAACCTAAAAACGTT
AGATTGTGAAGGACTATCACATGGAATTTGGAAGTCATTGCTCCCCCAATTCAACATTAATCAAACAATAACCACTGGTT
TGGGGTGCCTCAAAGCTCTAAATTTAATGGGTTGCAAACTTATGGATGAGGACATTCCTGAAGATCTCCATTGCTTTTCT
TCATTAGAAACACTAGATCTCAGCTATAATAACTTCACAACACTCCCTGATAGTCTTAGCCACCTCAAGAAGTTAAAGAC
ATTGAACCTGAATTGTTGCACTGAGCTTAAAGACTTACCAAAGCTTCCAGAAAGTTTGCAATATGAACGATTTAGAAGTA
AGTTTGATCTGCTTCTACATGGAGATAAGATCCCAAAATTTTTCAGCAATCAAAGCAAAGGAAACATGACAGAAATAAAG
TTACCACAATATTTGGAAAAATTTCGAGAGAGTATAGGTGTTGCTGTGTGTGCTCTTGTGGTTGTGGACAAGAAAAGAAG
AAAACTAAATGAGATTATTCCAGAACGAGAGAGGTATACAAAAGTTGTGGATCTTATATGTAAATTCAAAGTTGACTCGT
ATCAAATTATGCCAGAGCACTGTCACTTCACATCCCAACAAAAACTGTTGAGTGAATATGCTTCACAATTCCTTTGGCTC
TCTTACATTCCCCTTCATGGATTTAATATCAATTGGCATTATTGCACCCAGTTTGAAATTGCACTTGAAACTAGCTGTGA
TGAGCTATTTGGAGTGAAGAATTGTGGTCTTCATCTCATACATAAGCATGAAAGGATGATGATTGATAAGATGGTAATGG
AGTCAACTGTTCCATCATCCACTAGCCACAAAGGAAAGGAACCTCAAATTCATTGA

>Cucsa.237560
ATGGCTTCCTCAACCCCCAAGGAATTATCTTCTTTTTCTTCTTCTCCTAGATTCATATTTGACGTCTTTCTCAGCTTCAG
AGGCGTCGACACTCGCAAGAATGTCACAAATCGTCTTTACGAAGCTCTGAGGCGACAAGGCATCATTGTTTTCAGAGATG
ACGATGAGCTCGAGAGAGGGAAGACTATTGCTAACACTCTAACCAACTCGATTAACCAATCCAGGTGTACCATTGTTATT
CTCTCTAAAAGATATGCAGATTCAAAATGGTGCTTGAGGGAGTTGGTTGAGATTGTCAAATGCAAGAATACCTTCAAGCA
ATTAGTTCTTGTGGTTTTCTACAAAATTAAGCCCTCCGATGTCAACAGCCCTACTGGGATTTTTGAGAAATTTTTTGTTG
ATTTCGAAAATGATGTTAAGGAGAATTTTGAAGAGGTTCAGGACTGGAGGAAGGCCATGGAAGTGGTTGGAGGTCTCCCT
CCATGGCCTGTAAATGAACAGACCGAAACAGAGAAAGTCCAAAAGATTGTTAAGCATGCTTGCGATCTTCTGCGTCCTGA
TTTGCTTAGCCATGATGAGAATTTGGTTGGCATGAACTTGAGATTAAAAAAAATGAATATGCTTATGGGCATAGGACTGG
ATGATAAGCGCTTTATTGGGATATGGGGGATGGGTGGAATAGGCAAGACAACTATTGCTAAAGCTGTTTTCAAAAGTGTC
GCTCGTGAATTCCATGGAAGTTGCATTCTGGAAAATGTTAAGAAAACTTTAAAGAATGTTGGAGGCTTGGTGTCCTTGCA
GGAGAAACTTCTTTCCGATACTCTAATGAGAGGAAAAGTTCAAATTAAAGATGGCGATGGAGTTGAAATGATAAAGAAAA
ACTTAGGAAATCAAAAAGTTTTTGTTGTTCTTGATGGTGTTGATCATTTTAGCCAGGTGAAAGATCTGGCAGGAGGAGAA
GAATGGTTTGGTTGTGGAAGTAGAATCATCATTACAACAAGAGATGAAGGTTTGCTTCTTTCTCTTGGAGTTGATATAAG
ATACAATGTTGAGAGTTTCGATGATGAAGAGGCTCTTCAGCTCTTTTGCCATGAAGCATTTGGAGTAAAGTTCCCTAAGA
AAGGTTATTTGGATCTTTGTATGCCATTTATAGAATATGCTGAGGGCCTTCCATTAGCAATCAAGGCTCTTGGGCATTCT
TTGCACAATAGATTGTTTAAGTCATGGGAAGGTGCTATTAGAAAGTTAAATAATTCTTTAAACAGGCAAGTATATGAAAA
CTTGAAAATTAGTTACGATGCACTTGGAAAGGAAGAGAGGAGAATTTTTTTGTATATTGCCTGTTTTCTTAAAGGACAGA
ACAAAGACCAAGTCATTGACACATTCGTGAGTTTTGAAATTGATGCTGCTGATGGGCTTCTTACCAGAAAAAATGCTGCC
GATGTACTTTGTATAAAAGAAACTGCTGCTGATGCTCTAAAAAAATTGCAGGAGAAATCCCTTATAACTATGTTATATGA
CAAAATAGAGATGCATAATTTACACCAAAAACTAGGTCAAGAAATTTTTCATGAGGAGTCATCGAGGAAAGGTAGTAGGC
TATGGCATCGAGAGGATATGAACCACGCTTTAAGGCATAAACAGGGAGTTGAAGCTATTGAAACCATTGTCTTGGACTCA
AAAGAGCATGGAGAGTCACACTTAAATGCCAAGTTCTTTTCAGCAATGACCGGTCTAAAAGTGTTGCGTGTTCATAATGT
ATTCCTTTCTGGAGTTCTTGAATATCTCTCAAACAAGTTGAGACTTCTCAGTTGGCATGGATATCCCTTCAGAAATTTAC
CATCGGATTTCAAGCCGAGTGAACTATTGGAACTCAATTTACAGAATAGCTGCATTGAAAATATTTGGAGAGAAACAGAG
AAGTTGGATAAATTGAAGGTAATTAACCTTAGTAATTCCAAGTTCTTATTGAAGACCCCTGACCTGTCAACGGTGCCAAA
TCTTGAGAGGTTGGTCTTGAATGGTTGTACAAGACTACAAGAGCTTCACCAATCTGTCGGCACTCTAAAGCATCTAATCT
TTTTGGATCTTAAGGACTGCAAATCTCTCAAAAGCATTTGTTCTAATATTTCTCTTGAATCACTCAAGATTCTCATTCTT
TCTGGTTGTTCAAGACTTGAAAATTTTCCAGAGATTGTGGGAAACATGAAACTTGTGAAAGAGCTTCATTTAGATGGCAC
TGCTATTCGAAAATTGCATGTCTCGATTGGAAAACTTACAAGCCTTGTTTTGTTGGATCTTAGATACTGCAAAAATCTTC
GTACACTTCCAAATGCAATCGGTTGCTTAACATCCATTGAACATCTCGCATTGGGTGGCTGCTCAAAGCTTGATAAAATT
CCTGACAGCTTGGGGAACATTTCTTGTTTAAAGAAACTTGATGTGAGTGGTACTTCTATTAGTCATATCCCATTTACTCT
AAGACTTTTGAAGAACCTTGAAGTATTGAATTGCGAAGGCCTATCCCGAAAATTATGTTATTCGTTGTTCCTATTATGGA
GTACGCCGAGGAATAACAATTCACATTCATTTGGTTTGTGGTTGATAACTTGCTTAACGAATTTTAGTTCGGTAAAGGTT
TTGAATTTTAGTGATTGCAAGCTGGTAGATGGAGACATACCCGACGACCTCAGCTGTTTGTCTTCATTGCACTTTCTGGA
TCTAAGCAGGAACCTCTTCACCAATCTGCCTCATAGTTTGAGTCAACTTATCAATCTCAGATGCCTTGTTTTGGACAACT
GCAGTAGACTCAGGTCATTACCGAAGTTCCCAGTCAGTTTACTTTATGTACTCGCAAGGGATTGTGTGTCACTGAAAGAA
CACTATAACTATAACAAAGAAGATCGCGGGCCTATGAGCCAAGCAGAAGTAAGGGTCCTTAGTTACCCCTCATCAGCTAA
AGACCAAAACTCTAAAATCTCTCAGTTAATGATATCAAGTATGTGCACAGCTTGCGAGAATGGGGGTTGA

>Cucsa.237410
ATGGAGAGAAGAGCTTCAATTAAATCCTTATCTCCTCCTCCCTATTCTATCTCTCTTCCTCTTCCTCCCTTACGAAACTA
TGACGTTTTCCTCAGCCACAGAGTTAAGGATACCGGGAGTAGTTTCGCAGCTGATCTTCATGAAGCTTTGACAAACCAAG
GAATTGTAGTTTTCAGAGACGGCATAGACGACGAAGACGCAGAGCAACCATATGTAGAGGAGAAGATGAAGGCCGTGGAA
GAATCGAGGTCTTCGATCGTGGTTTTTTCAGAGAACTACGGGAGTTTTGTTTGCATGAAGGAAGTAGGGAAGATTGTAAC
GTGTAAGGAGTTGATGGATCAACTGGTTCTTCCTATATTTTACAAAATAGATCCAGGCAATGTGAGGAAGCAAGAGGGGA
ACTTTAAGAAGTACTTTAATGACCATGAAGCCAATCCTAAGATTGATATTGAAGAAGTTGAGAACTGGAGATATTCTATG

AATCAAGTTGGCCATCTCTCTGGATGGCATGTCCAAGATTCCCAGTCTGAAGAAGGGAGCATAATCAATGAAGTTGTGAA
GCATATATTCAACAAATTGCGTCCTGATTTGTTTCGATATGATGATAAATTAGTTGGAATTTCCCCAAGATTACACCAAA
TAAATATGCTTTTGGGAATAGGTTTAGATGATGTACGCTTTGTTGGAATATGGGGAATGGGTGGAATTGGCAAAACTACA
ATTGCTAGAATCATTTACAAAAGTGTTTCTCATTTATTTGATGGATGTTATTTCTTGGACAATGTCAAAGAAGCTTTGAA
GAAAGAAGACATAGCTTCATTACAACAAAAGCTTCTAACAGGAACTCTAATGAAAAGAAACATTGACATCCCTAATGCTG
ATGGAGCTACATTAATTAAGAGAAGAATAAGTAATATTAAAGCTCTTATAATTCTTGACGATGTCAACCATCTAAGCCAA
CTTCAAAAATTAGCCGGCGGTTTAGATTGGTTTGGCTCAGGAAGTCGAGTCATCGTTACAACGAGAGACGAACATCTCCT
AATTTCACATGGAATCGAAAGACGATACAATGTTGAAGTGCTGAAAATTGAAGAAGGTCTTCAGCTTTTTTCACAAAAGG
CATTTGGAGAAGAGCATACAAAGGAAGAGTATTTTGATGTTTGTAGCCAAGTTGTAGACTATGCTGGAGGACTTCCATTG
GCAATTGAGGTTCTTGGATCTTCTTTACGTAATAAACCAATGGAGGATTGGATAAATGCAGTGGAAAAGTTGTGGGAAGT
TCGTGATAAGGAAATTATAGAAAAGTTGAAAATTAGTTATTATATGTTGGAGAAATCTGAACAGAAAATTTTTCTAGATA
TTGCATGTTTTTTTAAGAGAAAGAGTAAGAAACAAGCAATAGAAATTCTTGAAAGTTTTGGATTTCCTGCTGTTCTTGGA
CTAGAAATATTGGAGGAGAAATGTCTTATTACTACACCACATGATAAGCTACATATGCATGATTTAATACAAGAAATGGG
CCAAGAAATTGTTCGCCAAAACTTTCTGAATGAGCCCGAAAAGCGAACTAGGTTGTGGCTTCGTGAGGATGTCAATCTCG
CACTAAGTCGAGATCAGGGAACAGAAGCAATTGAAGGGATAATGATGGATTTGGATGAGGAAGGAGAATCACATTTGAAT
GCCAAAGCCTTTTCAGAAATGACAAATCTAAGAGTATTGAAATTGAACAATGTTCATCTTAGTAAAGAAATTGAATATCT
GTCTGATCAACTAAGGTTTCTCAATTGGCATGGTTACCCTTTAAAGACCTTACCATCAAATTTCAATCCCACAAATCTAT
TGGAGCTTGAGTTGCCAAATAGCTCTATTCACCATCTTTGGACTGCTTCAAAG

>Cucsa.237440
ATGACATCCTTATCTTTTCCTCCTCCTCCTCCTCCTCCTTATTCTATCTCTCTTCCTCTTCCTCCCTTACGAAGATATGA
CGTTTTCCTCAGCCACAGAGCTAAGGACACTGGATGTAGTTTCACTTCCAACCTCCACGAAGCTCTAACAAGTCAAGGAA
TTGTAGTTTTCATAGACAAGGAAGACGGAGGGAAACCGTTAACGGAGAAGATGAAAGCGGTGGATGAATCGAGGTCTTCG
ATCGTGGTTTTTACCAAGAATTATGGGAGTTTGGTTTGCATGAAGGAAATAAGGAAGATTAGAATGTGTCAGAAGTTAAG
GGATCAATTGGTCCTTCCAGTATTTTACAAAATAGATCCAGGCGATGTGAGGAAGCAAGAGGGGAGCTTTGAGAAGTACT
TTAATGAACATGAAGTCAATCCTAATATTAGTATTGAAGAAGTTAAAAAATGGAGAAAATCTATGAACAAAGTTGGCAAT
CTCTCCGGATGGTCTGAAGAAGGGACCATCAATGAAGTTGTGAATCATATTTTCAACAAATTACGTCCAGATTTATTTCG
ATATGATGATAAATTAGTTGGAATTAGCCGAAGATTACATGAAATAAATAAGCTAATGGGAATAGGCTTAGATGACGTAC
GGTTGATTGGAATATGGGGAATGGGTGGAATTGGCAAAACAACCATCGCTAGAATCATTTACAAAAGTGTTTCCCATTTG
TTTGATGGATGTTATTTTTTGGACAATGTCAAAGAAACTTTAAAGAAAGAAGGCATAGCTTCTTTACAACAAAAGCTTCT
AACAGGAGCTCTAATGAAAAGAAACATTGACATCCCTAATGCTGAAGGAGCTACATTAATCAAGAGAAGAATGAGTAATA
TTAAAGCTCTTATAATTCTCGATGATGTCGACCATCTAAGCCAACTTCAGCAGTTAGCTGGCGGTTCGGATTGGTTCGGT
TCAGGAAGTCGAGTCATCGTTACGACGAGAGAAGAACATCTCCTAATTTCACATGGAATCAAAAGACGATACAATGTTGA
AGTGCTGAAAATTGAAGAAGGTATTCAGCTTTTCTCACAAAAGGCATTTGGAGAGGACCATCCAAAGAAAGGGTATTTTG
ATCTTTGTAGCCAAGTTGTAGATTATGCTGGAGGGCTTCCATTAGCAATTGAGGTTCTTGGATCTTCTCTACGTAATAAA
CCAATGGAGGATTGGATAGATGCTGTGAAAAAGTTGTGGGAAGTTCGTGATAAGGAAATTATTGAAAAGTTGAAAATTAG
TTATTATATGTTAGAGAAAGATGATAGGGAAATTTTTCTAGATATTGCATGTTTTTTCAAGAGGAAGAGTAAGAGACAAG
CAATAGAAATTCTTGAAAGTTTTGGATTTCCTGCTGTTTTTGGACTAGACATATTGAAGGAAAAGTCTCTTATTACTACA
CCACACGAGAAGATACAAATGCATGATTTGATACAAGAAATGGGTCAAAAAATCGTTAACGAAAAGTTTCCAGATGAACC
CGAAAAACGAAGCAGGTTGTGGCTTCGTGAGGATATAACTCGTGCTCTAAGTCATGATCAGGGAACAGAAGCAATTAAAG
GGATAATGATGGATTTGGATGAGGAGGGAGAATCACATTTAAATGCCAAAGCCTTTTTTTCAATGACAAATCTAAGAATA
TTGAAATTGAACAATGTTCATCTTAGTGAAGAAATTGAATATCTGTCTGATCAACTAAGGTTTCTCAATTGGCATGGTTA
CCCTTTAAAGACCTTACCATCAAATTTTAATCCCACAAATCTATTGGAGCTTGAGTTGCCAAATAGCTCTATTCACCATC
TTTGGACTGCTTCAAAGGTACATCAAAACAACAGTAGTAATTAA

>Cucsa.237470
ATGCTACTAATTAACTATGCATCCAACGCTTGCACATACTTGTTATCAATTGGAAGTCTTATTACAACTAAAAAGAGAGA
TGTACTTCATCAACTAAATTATAGAGATAAAGTGCTAGAATACAAGGTGGAGTTACTTTCTCGTGAAAGTGCTTACTCAC
TGTTTAGCAAGAATGCATTTGGAGGTGGCCCTTCCGATAAAGATGAACTTTGTAATGAAATTGTGGAAAAGGTTGGAAGA
CTTCCATTAGCTTTGAAAACCATTGGCTCCTATTTGCATAATAAGGAGTTGGATGTGTGGAATGAAACATTGAAGAGACT
AGATGGAGTGGAGCAAGACTTCTGTGATACAGTATTGCAGAAAA

>Cucsa.237480
ATGGGGAAGCAGACTAATCATAAGCTAGTACTTGCTCACAAAACTAGTTTAGTTGGAATGGAGAATCAAGTGGAGAAAGC
TTGTAATCTCCTAGATTTAGAACGATCCAAGAACATACTTTTTGTGGGGATTTTTGGGTCAAGTGGCATTGGTAAAACAA
CCATTGCTGAAGTTGTTTACAACACAATTGTAGATGAATTCCAAAGTGGTTATTTTCTCTACCTTTCTTCAAAGCAAAAC
AGTTCAGTCCCACTTCAGCATCAAATGCTTTCTCATCTTCAATCAAAAGAAACTAAAATCTGGGATGAAGATCATGGAGC
ACAACTGATTAAGCATCACATGATTCATGAAAGAAACCAAATTCAAAGGTTAGTTGGAAGTCCGAACTGGTTTGCACCTG
GAAGCAGAGTTATTATTACAGCTAGAAATAGAGATGTTCTGCATGAACTCAATTATAGAGATCAAGTGCAAGAATACAAG
GTGGAGTTACTTTCTCGTGAGAGAGCTTACTCACTGTTTTGCGAGAATGCATTTGGAGATGGCGGCCCATCTGATAAAAA
GGATCTTTGTAGTGAAATTGTGGAAAAGGTTGAAAGACTTCCATTAGCTTTGAGAACCATTGGTTCCTATTTGCATAATA
AGGACTTGGATGTATGGAATGAAACATGGAAGAGACTAGATGAA

>Cucsa.237530
ATGGGGAAGCAGACTGATAATAAGCTAGTACTTTCTCACAAAACTAGTTTAGTTGGAATGGAGAATCAAGTGAAGAAAGT
TTGTAATCTCCTAGATTTAGAACGATCCAAGGACATACTTTTTGTGGGGATATTTGGATCAAGTGGCATTGGTAAAACAA
CCATTGCTGAAGTTGTTTACAACACAATTATAGATGAATTCCAAAGTGGTTGTTTTCTTTACCTTTCTTCAAAGCAAAAC
AGTTTGGTCCCACTTCAGCATCAAATTCTTTCTCATCTTCTATCAAAAGAAACTAAAATTTGGGACGAAGATCATGGAGC
ACAACTGATTAAGCATCACATGAGTAATAGAAAAGTTGTTATTGTTCTTGACGGAGTTGATGAAAGAAACCAAATTGAAA
AGTTAGTTGGAAGTCCGAATTGGTTTGCACCCGGAAGCAGAGTTATTATTACGGCTACAAATAGAGATGTTCTGCATCAA
CTCAATTATAGAGATCAAGTGCAAGAATACAAGGTGGAGTTACTTTCCCGCGAGAGTGCTTACTCACTGTTTTGCAAGAA
TGCATTTGGAGATGGCCCATCTGATAAAAATGATCTCTGCAGTGAAATTGTGGAAAAGGTTGGAAGACTTCCATTAGCTT
TGAGAACCATTGGTTCCTATTTGCATAATAAGGACTTGGATGTATGGAATGAAACACTGAAGAGACTAGATGAAGAGGAA
CAAAATTACTTTGATACAATATTGAAGAGAAAT

>Cucsa.239860
ATGGCCGAATTTATAATAAATGTTGCGTCAGTAATTGTAACACCAATAGGAAAGTATGTG
ATTAAACCAATTGGAAATCAACTTGGTTACATTGTTTTCTACAACAGAAACAAGAATGAG
ATTAAAGAGCAACTTGAAAGTCTTGAGACTACTAAAAAGGATTTGGATCTAAGGGTTGAA
GATGCAAAAAGCAAGGCATATACCATCTTTACGAAAGTTTCAGAGTGGTTGGTCGCTGCG
GATGACGAAATAAAGAAATCTGATGAGCTATTCAATTCCAACCCACCTTGCCTTAACTTT
CTCCAACGACACCAACTAAGTAGAAAGGCAAGGAAGAGGGCGACGGATATCCGCCGACTC
AAAGACGGAGGAAACAACTTTCTGGAAGTTGGTTGTCCTGCCCCTTTACCGGATACTATG
AATACTATTGTTCCTGAAGCTTATCAAACTTTAGGATCAAAAACCTCAATGGCCAAGCAA
ATTAAGGACGCCCTTGCAAAACCTGAGGTAAGAAAGGTTGGAATCTATGGTATGGGAGGT
GTTGGAAAAACATATTTGCTCAAGGAAGTTAAGAAATTGGTGTTGGAAGAAAAATTGTTT
GATCTAGTGATTGATGTGACTGTAGGTCAATCTAATGATGTAATGAATATGCAACAACAA
ATTGGAGACTTCCTCAATAAAGAATTGCCAAAGAGTAAGGAGGGAAGAACATCCTTTCTA
CGAAATGCATTGGTGGAAATGAAAGGTAATATCCTGATCACATTCGATGATTTATGGAAT
GAATTTGATATCATAAACGATGTTGGAATTCCGTTAAGTAAAGAAGGATGTAAGACACTT
GTCACAAGTCGTTTTCAAAATGTTCTAGCCAATAAAATGAATATAAAAGAGTGTTTTAAG
GTGACTTGTCTAGACGATGAAGAGTCTTGGAAGTTTTTTAAGAAAATTATTGGTGATGAG
TTTGATGCAAAAATGGAAAACATTGCAAAGGAAGTGGCCAAACAATGTGGAGGATTACCA
CTTGCACTTGATATCATTGCAAAAACATTAAAGAGATCAAGACATATAAATTATTATTGG
GAGGGAGTGTTAAGTAAGCTGAAAAATTCAATTCCGGTGAATATTGACGTGGGTGAAAAA
GTTTATGCTTCACTTAAACTAAGCTATGAACATTTGGATGGAGAAGAAGTCAAATCACTA
TTTCTTCTTTGTAGCGTATTTCCAGATGATCATGGGATTTCAGTAAACGATCTGCAAATG
TATGTGATGGGTATGGGACTATTGAAAATGGTAAATACTTGGAAGGAAGCAAGAGCTGAA
GCACATTACTTGGTCGAGGATCTTACATCATCTTCTTTACTTCAACGACTTAAGAATAGA
GATGTTAAAATGCATGATATAGTTCGTGATGTTGCAATATACATTGGACCAGACTTTAAC
ATGTCTACACTTTACTATGGATATAGTACAAGTAGCAAAGGGCTAGATGAGGATAAATGT
AGATCTTATCGTGCAATCTTTGTAGACTGTAAGAAGTTTTGCAACCTTCTTCCAAACTTG
AAGCTTCCAAAACTAGAATTGTTAATATTAAGTTTTCCTTTTTGGGGGAAAGATAGAAAT
ATTGACATTATGGATGCATATTTTGAAGGAATGGAAAATCTTAAGGTTTTGGACATTGAA
GGAACAAGTTTCCTTCAACCATTTTGGACACCGTTAAAGAACCTTCGAACGTTATGTATG
TCATATTGTTGGTGTGAGGATATTGATACAATTGGGCACTTAAAGCAATTGGAAATTTTG
AGGATTAGTAATTGTAGAGGCATCACAGAATTACCAACGTCTATGAGTGAATTGAAACAA
CTTAAGGTATTAGTTGTGTCGCATTGCTTCAAGTTGGTGGTGATTCACACAAACATTATT
TCAAGCATGACCAAATTAGAAGAGTTGGATATACAAGACTGCTTTAAGGAATGGGGAGAA
GAAGTAAGGTACAAGAACACATGGATTCCAAATGCACAACTTTCAGAATTGAATTGTCTG
TCACATCTTTCTATTTTAAGAGTACGTGTTTTGAAGCTTACCATTCTCTCCGAGGCTTTG
AGTTCACAAATGTTGAAAAACCTAAGAGAATTCTTTATTTATGTTGGTACCCATGAGCCT
AAGTTTCATCCTTTTAAATCATGGTCGAGTTTTGATAAATATGAAAAAAATATGTCCTTT
AATATGAAATCGCAGATTGTTTCAGTCAACGGGACGAAACTTAGCATATTATTAGAAGGA
ACTAAAAGGTTGATGATTCTAAATGACTCCAAAGGTTTTGCAAATGATATTTTCAAAGCA
ATTGGAAATGGTTATCCCCTGTTGAAGTGTCTTGAAATTCACGATAATTCAGAGACACCA
CATTTGAGAGGAAATGATTTCACATCTTTGAAGAGGTTGGTTCTTGATAGAATGGTGATG
TTGGAGAGTATTATTCCGAGGCATTCTCCAATAAATCCTTTCAACAAACTTAAATTCATA
AAAATAGGAAGGTGCGAGCAGCTAAGGAATTTTTTTCCACTCTCTGTTTTTAAAGGGCTT
TCAAATCTTCGACAGATTGAGATCTATGAATGTAATATGATGGAGGAGATTGTATCAATA
GAAATTGAAGATCATATCACTATTTACACTTCTCCTTTGACATCTTTACGCATCGAGCGT
GTGAATAAACTTACAAGTTTTTGCAGTACCAAATCATCCATCCAACAAACAATTGTTCCC
TTATTTGATGAACGACGGGTTTCATTTCCTGAATTGAAGTATTTATCAATTGGTAGAGCA
AACAATTTGGAGATGCTATGGCATAAGAATGGAAGTTCCTTTTCCAAACTTCAAACAATA
GAGATTAGTGATTGCAAGGAGTTGAGATGCGTGTTTCCTTCAAATATAGCGACGTCACTT
GTCTTTTTAGATACATTGAAAATCTATGGTTGTGAGTTATTGGAAATGATATTTGAAATT

GAAAAGCAGAAGACTTCGGGAGATACAAAAGTAGTGCCATTGAGATACTTATCTTTAGGA
TTTCTAAAAAATTTAAAGTACGTGTGGGACAAAGATGTTGACGATGTTGTGGCATTTCCA
AACCTAAAGAAAGTTAAGGTTGGTAGATGCCCTAAGTTGAAAATTATTTTTCCAGCTTCC
TTCACCAAATATATGAAAGAAATTGAAGAGTTAGAAATGGTTGAGCCGTTTAATTATGAA
ATATTTCCAGTGGATGAAGCATCAAAGTTAAAAGAGGTTGCATTGTTCCAAAGCTTGGAA
ACATTGAGAATGAGTTGTAAGCAGGCTGTAAAAGAGAGGTTTTGGGTTATGTCAAAGTTC
TTCAAACTCAAAAGTCTTGAATTGTTTGGTTGTGAAGATGGTAAAATGATTAGCTTGCCG
ATGGAAATGAATGAAGTATTATACAGCATTGAAGAATTGACAATTAGAGGATGCCTCCAG
CTGGTAGATGTAATTGGAAATGACTATTATATCCAAAGATGTGCAAATTTGAAGAAGTTA
AAATTGTATAATCTTCCGAAGCTTATGTACGTGTTGAAGAACATGAATCAAATGACTGCA
ACCACATTCTCCAAGTTGGTTTATCTTCAAGTAGGTGGTTGCAATGGAATGATAAATTTA
TTTAGTCCTTCAGTGGCAAAGAATCTAGCGAATCTCAATTCCATTGAAATATATGATTGT
GGAGAAATGAGAACCGTAGTTGCAGCAAAAGCAGAGGAAGAAGAGGAAAATGTTGAAATT
GTGTTCAGCAAGCTAACTGGTATGGAATTCCATAATTTAGCAGGATTGGAATGTTTTTAC
CCTGGAAAATGCACACTTGAATTCCCCTTATTAGATACGTTGAGGATAAGCAAATGCGAT
GACATGAAAATCTTTTCATACGGAATAACAAACACTCCCACTTTGAAAAACATCGAGATT
GGAGAACATAACTCATTGCCAGTATTACCAACACAAGGGATAAATGACATTATCCATGCT
TTTTTCACAATTGAGTTTACTAGTATATGTGGTGTACATACTATACTTAGTGAAAAAGAA

>Cucsa.248810
ATGGCTGATTTCCTATGGACTTTTGCTGTCGAAGAAATGTTGAAGAATGTGTTGAAGGTTGCAGGGGAGCAAACTGGCCT
TGCATGGGGCTTCCAGGAGCATCTCTCCAACCTCCAAAAATGGCTACTCAACGCTCAAGCTTTCTTACGCGATATCAACA
CCAGAAAACTACATCTTCATTCTGTGAGCATCTGGGTGGACCATCTTCAGTTTCTTGTTTATCAAGCCGAGGATCTATTA
GACGAAATTGTTTATGAACATCTTCGACAAAAGGTCCAAACAACAGAAATGAAGGTGTGTGATTTCTTCTCTCTTTCTAC
CGATAATGTTTTGATCTTTCGTCTTGACATGGCAAAAAAAATGATGACCCTTGTACAACTGTTAGAAAAGCATTACAATG
AGGCTGCTCCTTTAGGACTAGTTGGGATTGAAACTGTAAGACCCGAGATCGATGTTATTAGTCAATATCGAGAGACAATT
TCAGAACTTGAAGATCATAAGATTGCGGGGAGGGATGTTGAAGTTGAAAGTATAGTGAAACAAGTGATTGATGCTAGCAA
TAATCAACGTACATCTATCCTGCCCATTGTTGGTATGGGTGGATTAGGAAAAACAACTTTGGCAAAGTTAGTTTTTAACC
ATGAGTTGGTTAGACAACGTTTTGATAAAACTGTATGGGTTTGTGTGTCTGAACCATTTATTGTCAACAAGATTTTGCTT
GATATTTTAAAAAATGTAAAAGGTGCCTATATTTCTGATGGAAGGGATAGCAAGGAGGTTTTACTTCGTGAACTCCAAAA
AGAGATGCTTGGGCAAAGCTATTTTCTTGTGCTTGACGATGTTTGGAACGAAACTTTTTTTCTATGGGATGACTTGAAAT
ATTGTTTGCTCAAGATCACTGGAAACTCTAACAATAGTATCCTTGTGACTACAAGGAGTGCTGAAGTTGCAAAAATCATG
GGAACATGTCCTAGTCATCTTTTAAGTAAATTATCTGATGATCAATGTTGGTCTTTGTTTAAAGAAAGTGCAAATGCATA
TGGACTATCAATGACTTCAAACTTGGGGATCATTCAAAAAGAGTTGGTCAAAAAAATTGGTGGCGTACCATTGGCTGCAC
GAGTTTTGGGTAGGGCAGTAAAATTTGAAGGAGATGTTGAGAGATGGGAGGAAATGTTGAAAAATGTGCTAACAACTCCA
CTGCAAGAGGAAAATTTTGTTTTATCTATATTAAAATTAAGTGTGGATCGTTTACCATCATCTTCAGTAAAGCAGTGTTT
TGCATATTGTTCAATTTTTCCCAAAGACTTTGTGTTTGAAAAACAAGAATTGATTCAAATGTGGATGGCCCAAGGTTTTC
TTCAACCACAACAAGGAAGATACAATAACACAGCAATGGAAAATGTAGGAGATATATACTTCAACATCTTGTTGTCACGT
TGCTTATTTGAATTCGAAGATGCCAATAAAACAAGGATAAGAGATATGATAGGTGATTATGAAACAAGAGAAGAATATAA
GATGCATGATCTTGTACATGATATTGCAATGGAAACTTCAAGGTCGTATAAAGATTTGCATCTAAATCCTAGCAATATAT
CGAAGAAGGAACTTCAAAAGGAGATGATAAATGTTGCAGGCAAGTTACGCACAATTGATTTCATTCAAAAGATTCCTCAC
AATATAGATCAAACACTTTTTGATGTTGAGATAAGAAACTTTGTTTGTTTGCGTGTTTTGAAGATATCGGGTGATAAATT
ACCAAAGTCAATTGGTCAATTGAAACACTTGAGATATCTAGAAATTTTAAGTTATTCAATAGAATTAAAATTACCAGAGT
CTATTGTTTCACTTCATAATTTGCAAACGCTAAAGTTCGTATACTCAGTGATTGAAGAATTTCCAATGAACTTTACAAAT
TTGGTAAGTTTAAGGCACTTGGAATTAGGGGAAAATGCTGACAAAACACCTCCACATTTAAGTCAATTGACTCAACTTCA
AACATTGTCTCATTTTGTAATCGGATTTGAAGAAGGTTTTAAGATTACTGAATTGGGTCCATTGAAAAACTTGAAAAGAT
GTCTGTGTGTTTTGTGTTTGGAGAAAGTTGAAAGTAAAGAGGAAGCAAAGGGAGCAGATTTGGCAGGAAAGGAGAATTTA
ATGGCGCTACACTTAGGGTGGTCCATGAATAGAAAAGATAATGATTTGGAAGTGTTGGAAGGACTTCAACCAAACATAAA
TCTCCAATCATTGAGAATCACCAACTTTGCTGGAAGACATTTGCCTAACAATATTTTTGTTGAGAATTTAAGAGAGATAC
ATTTGTCTCATTGTAATAGTTGTGAAAAGCTTCCAATGCTTGGACAACTAAACAACCTAAAGGAACTTCAGATTTGCAGC
TTTGAAGGCCTCCAAGTTATAGACAACGAGTTCTACGGCAATGATCCAAACCAAAGAAGGTTCTTCCCAAAGCTTGAGAA
ATTTGAAATCAGTTATATGATCAACTTAGAGCAATGGAAAGAAGTAATAACAAATGATGAATCATCAAATGTCACAATTT
TCCCCAATCTCAAGTGCTTGAAAATATGGGGATGTCCCAAATTATTAAACATTCCAAAAGCTTTTGATGAGAATAATATG
CAACACCTTGAATCATTGATCCTTTCATGTTGTAACAAATTGACAAAACTCCCAGATGGATTACAATTTTGTAGCTCTAT
TGAAGGGTTGACAATAGACAAATGTTCAAATTTGAGCATAAATATGAGAAATAAGCCGAAATTATGGTATTTAATCATTG
GTTGTGTAACGCAAATTCCTGAACAACTTCAACACCTCACTGCCTTACAATTTCTGTCTATTCAACATTTTAGATGCATT
GAAGCTTTGCCAGAATGGTTAGGAAACTATGTATGTTTGCAAACACTCAATCTTTGGAATTGCAAAAAATTGAAAAAACT
GCCTTCTACAGAAGCAATGCTACGTCTCACCAAATTAAATAAATTGCATGATCTTAATATTTGTTTATCACCTTCATTCT
CATCTCTCTTGTGCTTTTTAATTAATTAA

>Cucsa.249360
AATCTAAAGCGATTACTACGAGTAGCGAAACATCTAGTCGAAATTGATTCTCAACTAAGAGAAATTGAGGAGTCGGTCTC
TCATATTGGGTCCGAGGGAATTGGTAAGACCACTTTGGCTAAGGATTTGTACAACAAAATTGCTACCCAATTTGAAAGAT

GCTGCTTTCTACAAGATGTTAGACGAGAAGCTTCAAAGCAATATGGGCTCGTTCAACTACATGAAACCTTACTCTGTGAG
ATTTTAAAGGAGGATTTGAAGGTTGTCAATTGTGATAAAGGAATTAACATCATAAGAAGTAGACTGTGTTTAAAGAAAGT
TCTTATAGTTTTTGATGATGTGGATCATCACAGGCAATTAGAAGCACTAGTTGGTGAGCTCGATTGGTTTGGTCGAGGTA
GTAAAATCATTATGTTGACAAGGAATGGACATTTACTTTCTAGCCATGGATTCGATGAAAAGCATAAAATTCATGAATTG
GATCAAGACCATGCTCTTGTTCTTTTTAGCCTTTCAGAAAGTGCTACAAATTATTGTAAAGGTCTCTCTGTGGCACTCGT
TGTTTTGGGTTCTTTCCTTCGTGGTAGAGATCAAACAAGATGGAGTTGTATATTAGATGAATTTGAAAACTCTCTACCAA
AAGATATTAAAGATGTTCTTCAATTAAGTTTTGATGGACTAGAAGACAAAGCAAATGATATTTTTCTTGATATTTCTTTG
CATGTCATTCGACCGTTAGATTTTGGAATTATGATTCTCATGGATCTTTCACTTATTATGATTGAAAGTGATAGAGTGCA
AATGCATGGATTAATACAACAAATGGGTTGTAGCATAGTTCGTAATGAATCATCTCAACCTGAAAAGAGGAGTAGGTTGT
GGTTGGTTCAGGATATTGGGGAGGTATTCGTTAATAAGTCTGTGAGAAACTCTTTACCTAAATTGAAGCATGTTGATCTT
AGCTACTCTACTTTATTAGAGAAAATTCTGGATTTCTCTGCTGCATCAAACCTTGAAGAATTGTATCTCACCAATTGCAC
AAATTTAGGAATGCTAGATAAGTCTATTTTATCTCTCAATAAGCTTACTGTCCTAAACTTTGAAGGTTGTTCTAACCTTA
AAATGCTTTCAAGAGGCTACTTCATGTTAAGTTCTCTTAAAGAATTGAGGCCCTCTTACTATAAGAAGCTTGAGAAAATT
CCAGACTTATCTGCAGCATCAAACCTTAAGAGATTGTATCTCCAAGAATGCACAAATTTAAGAGTGATTCATAAATCTGT
TGGATCTTTGGATAAGCTTGGATTGTTGGACCTTAGTCAATGTACTAACCTAGTAAAGCTTCCAAGCTATCTCAGGTTAA
AGTCTCTATACACTTTATATCTTTCTGGGTGTTGTAAGCTTGAAAGCTTCCCAACAATTGCTGAAAACATGAAATATTTA
GAGGAATTGTATTTGAATTTTACTGCCATTTAG

>Cucsa.251930
ATGGCTGATTTTATATGGACATTTGCACTGCAAGAGATTCTCAAGAAGACATTGCACCTTGCAACCCAACAAATCCGTCT
GGCCTCCGGTTTCAACCACGACCTCTCTAAACTCCTCCACTCATTGCTCTTCTTCGAAGCCATTCTTCGCGATGTCGATC
GAACAAAATCCGACCTACAGTCGGTCAAGATTTGGGTCACTAAGCTTCAGGATTTAGTGCTCGATGCTGAAGTTGTGCTG
GACGAGCTCTCCTACGAGGACCTTAGGCGAGAAGTGGACGTCAATGGAAATTCGAAGAAAAGAGTACGCGATTTCTTTTC
GTTCTCGAATCCCTTGATGTTTAGGTTGAAAATGGCGCGTAAAATTAGAACCATCACCCAAGTTTTGAATGAGATTAAAG
GGGAGGCTAGTGCTGTTGGGGCTATTCCTACAGGGGGCAGTGATGAAATAGTGGCTGATAATGGCCATATTCCGGAGACT
GACTCATTTCTTGATGAATTCGAAGTTGTAGGAAGAAGGGCTGATATATCTAGAATAGTGAACGTTGTTGTTGATAATGC
CACTCATGAAAGGATCACTGTGATTCCTATTGTGGGAATGGGTGGTCTTGGAAAGACAACTTTGGCAAAAGCAGTTTTCA
ACCATGAGCTTGTGATAGCACATTTTGATGAAACTATTTGGGTGTGTGTGACTGCAACTTTTGATGAAAAGAAGATTTTA
AGAGCAATTTTGGAATCTCTAACGAATTTTCCAAGTGGTTTGGATAGTAAGGATGCTATACTTAGAAGGCTACAAAAGGA
GCTGGAAGGGAAAAGGTATTTTCTTGTGCTGGATGACGTGTGGAATGAAAATGTTAAACTGTGGAACAATTTCAAGAGTC
TTCTGCTAAAGATTACAAATAGTATTGGGAACAGAGTTCTTGTGACAACTAGAAGTGAGGAAGCTGGAAAAATCATGGAA
ACATTTCCCAGTCATCATGTAGAAAAGTTATCGGATGATGAATGCTGGTCAATATTCAAGGAAAGAGCATCGGCAAATGG
ATTACCACTGACTCCAGAATTGGAAGTTATTAAGAATGTGCTTGCAGAGCAGTTTGGAGGCATTCCATTGGTTGCAAAAG
TTTTGGGAGGGGCTGTACAATTTAAGAAAAGAACAGAGACTTGGTTGATGTCAACATTGGAAACCCTTATAATGAATCCA
CTTCAAAATGAAAATGACGTTTCATCTATTTTGAGATTAAGCGTGGATCATCTGCCAAACTCATCATTGAAACAATGCTT
TGCCTACTTTTCTAATTTTCCCAAGGGTTTTAACTTTGAAAAGGAACAACTAATCCAATTTTGGATGGCAGAAGGGTTCA
TTCAACCTTCTGATAAAGTAAACCCCGAAACCATGGAAGATATAGGAGATAAATACTTCAATATCTTGCTGGCTCGTTCC
TTATTTCAAGATATTGTTAAAGATGAGAATGGTAAAATTACACACTGTAAGATGCATCATCTTCTACATGATCTTGCTTA
TTCTGTCTCAAAATGTGAAGCACTGGGTTCGAATCTTAATGGTCTGGTTGATGATGTTCCTCAAATTCGACGATTATCCC
TGATTGGCTGCGAGCAAAATGTAACGTTGCCTCCTAGAAGGAGCATGGTGAAGTTGCGTTCTCTATTTTTGGATAGAGAT
GTGTTTGGCCACAAGATTTTAGATTTCAAGCGTTTGCGTGTTCTGAACATGTCCCTATGTGAAATCCAAAACTTACCAAC
TTCAATCGGAAGGTTAAAGCATCTAAGGTATCTTGATGTCTCAAATAATATGATAAAGAAACTTCCAAAATCTATTGTTA
AGCTTTATAAATTGCAGACCCTGAGGCTGGGTTGTTTCCGTGGAGAAGCCCCCAAAAAATTCATAAAATTGATCAGCTTG
AGACATTTCTATATGAATGTTAAAAGACCAACAACTAGGCACATGCCTTCGTATTTAGGCAGGTTGGTTGATCTTCAATC
CTTGCCTTTTTTTGTTGTTGGGACAAAGAAGGGTTTCCATATAGAAGAGCTTGGATACTTGAGGAATCTCAGAGGTAAAT
TAAAGCTTTACAATCTTGAATTAGTAAGAAATAAGGAGGAAGCCATGAGGGCAGATTTGGTGAAAAAGGATAAGGTGTAC
AAATTGAAACTGGTATGGAGTGAAAAAAGAGAAAATAATAATAACCATGACATTTCTGTTTTAGAAGGACTTCAACCACA
CATCAATCTTCAGTACTTGACAGTTGAAGCCTTTATGGGAGAACTTTTTCCAAATCTTACTTTTGTTGAAAATTTGGTAC
AAATTTCTCTAAAAAATTGTAGCAGATGTCGAAGAATTCCAACATTTGGACATCTACCCAATCTTAAGGTTCTTGAGATT
TCTGGATTACACAACCTAAAATGTATAGGAACAGAGTTCTATGGGAATGAATATGGAGAAGGAAGTTTGTTTCCAAAATT
GAAAAGATTTCATCTTTCAGACATGAATAATCTTGGACGTTGGGAAGAAGCAGCAGTGCCAACAGAAGTTGCAGTTTTTC
CTTGTCTTGAAGAGTTGAAAATTCTCGACTGTCCTAGACTAGAAATTGCACCTGATTACTTCTCAACTCTTAGGACATTA
GAAATTGATGATGTCAACAACCCAATTTCACAGATCACTCTTCAGACATTCAAACTACTTGGTATTATACACTCTGGCAA
CCTGAGTGGTTTGCCTGAGGAGTTACGTGGTAATCTGTCATCTCTTGAGGAGTTTAAGGTTTGGTATTATCTTCACTTGA
AATCCTTTCCAACTATTCAGTGGCTCACTGATATTTTGAAAGGCAAGACCGGATATGACACAAAGTGGACAAATATTCAA
TCTCATGGGCTAGAATCGTACACTTCTGTGAATGAATTGTCCATTGTTGGGCACTCTGATCTCACATCAACCCCAGATAT
AAAAGCTTTATATAATCTTTCGTCTTTAACAATTAGTGGCTTGAAGAAATTGCCAAAAGGATTTCACTGCCTCACTTGCT
TGAAAAGTTTGTCAATTGGTGGATTCATGGAGGGGTTTGATTTAGGCCTCTTTTGCATCTCAAGTCTCTTGAAAATCTT
GCAATGATAGACTTTATCCTTGCAGAAAGCACACTTCCTGATGAGCTTCAACACCTAACTGGCTTAAAGCACTTGAAAAT
TGTTGGATTTCAGGGCATTGAATCTCTGCCAGAGTGGTTAGGAAATCTTAACTCATTGGAAAGTTTGCACATTGAGAGTT
GCAGAAAATTGAGAGAGCTTCCAGAAGCCATGGGTTGCCTTGCCAAATTGGAGGAAGTGCGGAGTTTCAATTGCCCAGAG
TTGAGGGTTTACCAAGACGAATCAGAATGGGCGAAGATTTCTTACATTCCAAGATTCATATCATTCAATTATTGGGTTGA
TGAGGACCAACAAAGGATTCAGTTCAAAGTTTGCCACAAACCTAGCTAA

>Cucsa.252030
ATGGCGGGTTCAACCGGCGCTAATTCTTCTTCCAATTCTTCTCCTGCTCCTCCTCCCCCCAAAATCCTCCTAGCAAAGCC
TGGTCTCGTCCCCGGCGGACCTATTAACTCTAAGATTGGACGTGGCGCCGGCGCCGACGACGAACCAACATCCATTCGCT
CCCGTCTCCCTTCTCTTGGATCCCTTAATCTTCTCTCCGATTCATGGGATCTCCACATTGACCGTTTCCTCCCTTTTTTG
ACTGAGAATACGGAGTTCAAGGTGGTGGGGATAATTGGTCCACCGGGAGTTGGTAAGTCGACGATTATGAATGAGATTTA
TGGCTACGATGGAAGCTCTCCAGGTATGCTACCACCATTTCCAATACTATCTGAAGATGTTAGAGCAATGGCTAGGCATT
GTACATTGGGTATTGAGCCTCGAATTTCTTCTGAGAGGATTATACTTCTGGATACTCAGCCTGTGTTCAGTCCTTCGGTT
TTAGCTGAAATTATGAGACCCGATGGTTCGTCCACTGTTTCAGTTATTAATGGAGAATCTCCATCTGCTGAATTGGCTCA
TGAACTCATGAGTATCCAGCTGGGCATTCTTTTAGCGTCCATTTGCAACATTGTTCTTGTGATATCAGAGGGAGTTCATG
ATCTAAATATGTGGCATTTGATGTTAACTGTTGACTTGCTTAAACATGGCTTGCCTGACCCATCTTCTCCTATTTCTTCT
CACGCACAAAATTCTAATGTAGCATCTGAGAAGGAATACAAAGAAAAAACTTCTACAAGTGAAGAATATATGGCAACTCC
CATCTTTGTTCATGCCAAAGTGCAGGATCGAGATCTTGTTCCACAAAATATCTTGCAACTGAAGAGAGCATTTGCCTACT
ATTTTAAAACCTCTTCATTTATGGGAGACAAATTTGAAAAAGTTCATAGTGAGCAACTACTCTCCTCTGTGGTTCCTGAT
ACCAGGAATTTAGATGTAGATGGTGAAGATAGAAGGTTGCTTCTCATCCCAAACCGGAACAAGGATGACTCTACCAGAGG
TCAATACGAAAGTTTTAACTTAGCTCTATGGAAGCTGAGGGATCAGGTTCTATCCATGAATGGTGCATCGTTCCCAAGAA
CTGTTTCGGAACGTGATTGGTTAAAAAACTCTGTGAAGATATGGGAATCAGTGAAGAGCTCTCCTATTGTAATGGAGTAT
GCCAGAACGCTTCAATCTTCAGGTATGTTCAGGAGATAG

>Cucsa.275630
CAATCATCATCGTCTTGTTCTTCAAATTTGAAATGGAGTTATGATGTGTTTTTGAGTTTCAGAGGTGAGGATACTCGAAA
CAACTTCACTAGTCATCTTGACAGGGCCTTGCGTGAAAAGGGTGTCAATTTCTTCATAGATGACAAGCTAGAGAGGGGTG
GTCAAATTTCTGAATCCCTTCTCAAATCTATTGATGGTTCTAAAATTTCCATCATTATTTTCTCCAAAAATTATGCATCT
TCCACCTGGTGTTTGGATGAACTGGTGAAAATAGTTCAGTGCATGAAATCCATGGGACATATAGTTTTTCCTGTCTTCTA
CAAGGTGGATCCATCTGAGGTTCGAAAACAAACTGGTGGGTTTGGTGAAGCATTGGCCAAACATGAAGCTAATGAGTTAA
TGACCAACAAGGTTCAACCATGGAAGGAGGCTTTGACCACTGCTGCTTCTTTGTCTGGTTGGGATTTAGCAACTAGGAAG
AATGAGGCTGATCTTATTCATGACCTTGTTAAGGAGGTGTTGTCTATATTAAATCAAACACAACTACTACATGTAGCCAA
GCATCCAGTTGGAATTGATTCTCAACTTAGAGCTGTTGAGGAATTGGCCTCCCATGATGTGCCCGATGGTGTTAACATGG
TGGGGATACATGGGATGGGAGGCATTGGTAAGACCACTCTGGCCAAAGCTTTATACAACAAAATCGCTTATCAATTTGAA
GCTTGTTGCTTTCTTTCGAATGTTAGAGAAACCTTAGAGCAATTCAAAGACCTGGTTCAACTACAAGAAAAACTACTCAG
TGAGATCTTAAAAGATAATGCTTGGAAGGTGGGCAACGTTCATAAAGGAAAGAATATCATTAGGGATCGGTTATGCTCAA
AGAAAGTTCTTATCATTCTTGATGATGTGGATAAGGATGAACAATTAGACGCACTAGTTGGTGAACGTGATTGGTTCGGT
CGAGGAAGTAAAATCATAGCAACAACAAGAGATCGACATTTACTAGAAAACCATTCATTTGATATAGTATATCCTATTCA
GTTGTTGGATCCTAAGAAATCCCTTGAGCTTTTTAGCCTGCATGCTTTTAAGCAAAATCATCCCTCAAGTAATTATGTAG
ACCTTTCAAAATTTGCTGTAAGTTATTGCAAAGGTCTTCCATTGGCTCTTGTTATTTTGGGTTCTCTTCTCCATAAGAGA
GAGCGAAAAAATATGGAAAAGTAAATTACATGAACTTGAAAATTCCCTCGAACCAAGTGTTGAAGCTGTTTTTCAAATAGG
TTTTAAGGAGCTTCACGAAAGAGTGAAGGAGATTTTTCTTGATATTTCTTGCTTTTTCGTGGGAGAGGATATTAACTACA
GTAAGGATGTGTTAAAGGCATGTGATCTCAATCCAGACTATGGAATTATAATTCTTATGGATCTTTCCCTTGTTACTGTT
GAAGATGGAAAGATACAAATGCATGATTTAATACAACAAATGGGTCAAACAATTGTTCGCCATGAATCTTTTGAGCCTGC
AAAAAGGAGTAGGTTGTGGGAGGCAGAAGGAGCTATCAAGATATTGAAAGAGAAATCTGTGAGTGACTTTAGACAATGTT
TTTATTTACTTATTGCCAAAGATATATACTCAGAAGCATTTAGAAACATGAAAAATCTTAGATTGCTTATCCTTCAAAGA
GTAGCATACTTCCCTAGAAATATATTTGAGTATTTACCTAATTCGTTGAAGTGGATTGAGTGGTCTACATTTTATGTTAA
CCAGTCTTCGTCCATAAGTTTTTCTGAAACTCCCAACTTCTTTGCGACATTAAACCTTGAGAAATTATATCTTAGGGGAT
GCACGAGTTTGAAAGGAATGCGACCCTCTTTTAGAAAGTTTCCAAGCCACCTGAAGTTCAAATCTCTTAAAGTTCTGAAT
CTACGGGATTGTCTAAATCTTGAAGAAATTACTGACTTTTCAATGGCATCAAACCTTGAGATATTAGATCTCAATACTTG
CTTCTCTTTAAGAATAATTCACGAGTCTATTGGGTCTCTTGATAAACTTATCACCTTACAACTCGATTTATGCCATAACC
TAGAAAAGCTTCCTAGCAGCCTGAAGTTGAAGTCTCTTGATTCTTTGAGTTTCACTAATTGTTACAAGCTTGAACAACTT
CCAGAATTTGATGAAAACATGAAATCTTTAAGGGTGATGAATTTGAACGGTACAGCCATAAGGGTGTTACCTTCATCAAT
TGGATATCTTATTGGGCTCGAGAATTTAAACCTTAATGATTGTGCAAACCTGACTGCCCTTCCAAATGAAATTCATTGGC
TAAAAAGTCTCGAGGAACTTCATCTTCGCGGGTGTTCTAAACTCGACATGTTTCCCCCGAGATCAAGCTTAAATTTTTCC
CAAGAAAGCTCATATTTCAAGCTGACGGTATTGGATCTCAAAAATTGTAATATATCAAATTCTGATTTCCTCGAAACATT
ATCTAATGTCTGCACTTCCTTGGAGAAGCTAAATTTGTCAGGAAACAAATTCTCTTGTCTACCCTCTCTCCAAAATTTTA
AGTCATTAAGGTTTCTTGAATTAAGGAATTGCAAGTTTCTTCAAAATATAATAAAGCTTCCCCATCATTTAGCTCGGGTG
AATGCCAGTGGTAGCGAATTGTTGGCTATACGTCCTGATTGCATTGCTGATATGATATACTTGCACGCCAATGATCGACA
CCACATCAAAGTCTTGTTTCCCAACACAACATCAAAATTTGTATCGAAAAGATTTAAAAGTGCAAATGTTATTATGAGAA
CCCAAGGAGTCTATATGGTTGATAGACGGTTTTCATGCTATACATAA

>Cucsa.277260
ATGGCTGAATTTTTATGGACTTTTGCTGTTCAGGAAGTTTTGAAGAAGATTGTGAACTTTGGAGCAGAGCAAATTAGTTT
GGCATGGGGTTTGGAGAAGGAGTTGTCCCACTTGAAAAAGTGGTTACTCAAAGCGCAAACAATCTTAGCAGACATTAACA
CAAAGAAATCACACCATCATTCTGTTGGGTTATGGGTGGAAGAACTTCATGATATTATCTATGAAGCTGATGATTTGTTA
GATGAGATTGTTTATGAACAAATTCGACAAACTGTGGAGCAAACTGGTAAACTTAGAAAGGTACGTGATTCTATCTCACC
ATCCAAAAATTCCTTTTTGTTTGGTCTCAAGATGGCCAAGAAAATGAAGAAGATTACCAAAACTTTATACGAACATTACT

GTGAGGCAAGTCCTTTAGGACTAGTTGGTGATGAATCCCCCACAGAATCAGAGGCTGCACTTAATCAGATTCGGGAGACA
ACCTCAATTCTTGACTTTGAAGTTGAAGGAAGGGAAGCTGAAGTCTTGGAGATACTAAAATTGGTGATTGACTCTACCGA
TGAAGATCATATCTCTGTGATATCCATTGTTGGAATGGGTGGTCTTGGAAAAACAACTTTGGCCAAGATGGTTTTCAATC
ATGATGCCATTAAAGGACATTTTGATAAAACTGTATGGGTTTGTGTGTCTAAACCATTTATTGTGATGAAAATTTTGGAA
GCAATCTTTCAAGGTTTAACGAATACTAGTAGTGGTTTGAACTCCAGGGAGGCCTTGCTTAATCGACTCCGAGAGGAGAT
GCAAGGAAAAAAGTATTTTCTTGTGCTTGACGATGTTTGGGATAAAGAGAATTGCTTGTGGGACGAGCTTATTGGCAATT
TGAAATATATTGCTGGAAAATCTGGAAATAGTATTATGGTGACCACAAGGAGTGTAGAAGTAGCGACCATGGTGAAGACA
GTTCCCATTTATCATCTAAAAAAATTATCGGATGATCATTGTTGGGCGTTGTTAAAAAAAAGTGCAAATGCAAATCAGCT
GCAGATGAATTCAAAGTTGGAGAATACGAAAAATATTTTGGTTAGAAAAATTGGTGGTGTACCACTCATTGCAAAAGTTT
TAGGTGGGGCAGTAAAGTTTGAAGAAGGTGGGTCTGAGAGTTGGATGGCAAAAATTGAAAGCTTTGCGAGAAATATTTCA
ATAGAGGACAAAGATTTTGTTTTGTCCATATTAAAATTAAGTGTAGAGTCTCTCCCTCATTCTGCATTGAAGCAATGTTT
TGCTTACTGCTCAAATTTTCCTCAAGATTATGAATTTGATAAAGATGAAGCAATCCAAATGTGGATAGCCGAAGGATTTA
TTCAACCCGAACAAGAAAGAGAAAACTTGACAATGGAGAACATAGGAGAAGAGTATCTTAACTTTTTATTGTCTCGCTCC
TTATTTGAAGATGCCATTAAATATGATGGAAGAATTGTCACCTTTAAGATTCATGATCTAATGCATGATATTGCTTGTGC
AATTTCAAATCATCATAAGATGGACTCAAATCCTATTAGTTGGAATGGAAAAAGTACAAGAAAGTTGCGCACATTAATTT
GCGAGAATGAAGAAGCTTTTCATAAAATTCAGACTGACATTATTTGTTTGCGTGTGTTAGTCTTAAAATGGTTTGACACT
AATACCTTGTCGACTATTATGGCGAAATTGATACATTTGAGATATCTTGATATTTCAAACTGTAATATAAACAAGCTTCT
TCGAGATTCTATTTGTGCACTTTATAATTTACAAACGCTAAAACTTGGATATATTGAATGTGATCTGCCGAAGAATTTGA
GGAACTTGGTTAATTTGAGACATTTAGAATTTAAGAAATTTTTTGATATGGGACAAATGCCTTCACATATGGGCAACATG
ATTCATCTTCAAACACTATCTGAGTTTGTAGTTGGACTTGAGAAGGGTTGTAAAATTGATGAGCTTGGACCGTTAAAAGA
CCTCAAAGGTACACTAACTCTTAAAAATCTACAAAATGTGCAAAATAAAGACGAGGCTATGGCTGCAAAATTGGTGGAAA
AGAAGTATTTACGTCATCTAATCTTTCAATGGTTTCTAAATCTTTATGATAGAGGAGAATATGATGAAGATGATAACAAA
CAAGTGTTGGAAGGACTTCAGCCACACAAAAACGTACAGTCATTGGACATTAGAGGCTTCCAAGGAAGAGTTTTGAATAA
TAATATTTTTGTTGAAAATTTAGTTGAGATACGTTTGGTTGATTGTGGAAGATGTGAAGTGCTTCCTATGCTTGGACAGT
TGCCCAACTTGAAGAAACTTGAGATTATTTCAATGAACAGTGTGAGAAGTATAGGCAGTGAGTTCTATGGAGTTGACTGT
AACGACAGAAATTCTTCTGCTTTTCCTCAGCTGAACAAATTTCATATTTGTGGGTTGAAGAAGCTACAACAATGGGATGA
AGCAACGGTTTTTGCATCAAATCGCTTTGGATGTCTAAAAGAACTTATTCTTTCTGGATGTCATCAATTGGCAAAATTGC
CAAGTGGGTTAGAAGGGTGCTACTCCATTGAATATTTGGCCATCGATGGGTGTCCTAATTTAATGCTAAATGTGCAAAAT
TTGTACAACTTGTATCATTTAGACATTCGTGGGTTGAAAAGATTGCCAGATGAATTTGGTAAGCTCACTAACTTGAAAAA
ATTGAGAATTGGTGGATGTATGCAAAACTATGAATTTAGTCCCTTCATACATTTATCTTCTCAGCTTGTTGAACTTGAGT
TGACTGATGATGGGTCAAGTGGTAGTGAAACAACCCAACTTCCCCAACAACTTCAGCATCTGACCAACTTGAAGGTTTTG
AAGATTGCAGATTTTGATGACATTGAAGTTCTACCAGAATGGTTGGGAAACCTTACATGTTTGGCAACATTGGTTTTCCT
CGAATGCAAAAATTTGAAAGAGTTACCTTCGAGAGAGGCCATACAACGATTAACCAAATTAGATGATTTGGTGATCGATG
GATGTCCCAAACTACTACTAGGGGAAGGCGATCAGGAGAGGGCTAAACTTTCTCATCTCCCATCAAAATGTGGTTTGGGA
TTGAAGAGTGTTATGATAAACTTGTTGCCAAAAAAATCATTTTAA

>Cucsa.292710
ATGGGTTCTTCTTCAGTTAATGGAGCAGAATCATCATCGTCTTGTTCTTCAAATTCGAAG
TGGAGTTATGATGTGTTTTTGAGTTTTAGAGGAGAGGATACTCGAGACAAGTTCATCAGT
CATCTTGATTTGGCCTTACGTCGAGAGGGTGTCAATTTCTTCATAGATGACAAGCTAGAT
AGGGGTAAACAAATTTCTAAATCTCTTCTCAAATCTATAGAGGGGTCTAGGATTTCCATC
ATTATTTTCTCCCAAAATTATGCATCTTCCACTTGGTGTTTGGATGAAGTGGTGAAAATA
ATTGAGTGTATGAGATCCAAGAAACAAACAGTTCTGCCAGTCTTCTACAATGTTAGTCCG
TCTGAGGTTGTAAAACAAACTGGTATTTTTGGTGAAGCGTTTGCCAAATATGAAACAAAT
CCGTTAATGACCAACAAGATTCAACCATGGAAGGAGGCTTTGACCACTGCTGCTACTTTG
TCTGGTTGGGATCTTGGAAATTATTGGAAGAATAATGAAGCTCATCTTATTCAAGACCTT
GTTAAGAAGGTGTCTATATTAAAACAAACACAATTACTAAATGTAGCCAAGCATCCTGTT
GCAATCGATTCTCAACTTAAAGCTATTGAGGAATTAGCCTCCCATGGTGTGTCAGATAAT
GGTGTTAACATGGTGGGGATACATGGGATGGGAGGCATTGGTAAGACAACTTTGGCCAAA
GCTTTATACAACAAAATCACTTATCAATTTGAAGCTTGTTGCTTTCTTTCAAATGTTAGA
GAAACTTCAGAGCAATTCAACGGCCTAGTTCAACTGCAAGAAAAAATTACTCAATGAGATC
TTCAAGGATAATAACTTGAAGGTTGACAATGTGGACAAAGGAATGAATATCATAAAGGAT
CGATTGTGCTCAAGGAAAGTTCTTATGGTTTTGGATGACGTGGATAAGGACGATCAACTA
GATGCATTGGTAGGTGGACGTGATTGGTTCGGTCGAGGTAGCAAAATCATTGTGACAACA
AGAGATAGACATTTACTCGAAACATATTCATTTGATAAAATACATCCTATTCAATTGTTG
GATTGTGATAAATCTCTTGAGCTTTTTTGTTGGCATGCTTTTAAGCAAAGCCATCCATCA
AGGAATTATTCGGAACTTCCAGAACTAGTACGTTATTGCAACGGTCTTCCTCTAGCTCTT
GTTATTTTGGGCTCTCTCCCTTTGTAAGAGAGATCAAATAATATGGAAAAGCAAATTAGAT
GAACTTAAAAACTTCCCCGAACCAGGTATTGAAGCTGTTTTCCAGATAAGTTTTAAGAGG
CTTCCAGAAAACCCCCCAGTAAAGGAAATTTTCCTTGATATTTGTTGTTTTTTTGTGGGA
GAGGATGTTAGCTATAGTAAGAATGTGTTAAAGGCATGTGATCCTTATCTAGAATCAAGA
ATTATAATTCTCATGGATCTTTCTCTTGTTACGGTTGAAGACGGCAAGATACAAATGCAT
GATTTAATTCGACAAATGGGTCAGATGATTGTACGTCGTAAATCTTTTAAGCCAGAAAAA

AGGAGTAGGCTGTGGGTGGCAAAAGAAGCTGTCAAGATGTTGATAGAAAAATCAGGAACT
CATAAAGTTAAAGCCATAAAGCTAGACTTGCGCAACAACGGTTCACTGATTGTTGAAGCA
GAAGCATTTAGAAACATGGAAAATCTTAGATTGCTTATTCTTCAAAATGCAGCAAAATTG
CCTACAAATATATTCAAGTATTTACCTAATATTAAGTGGATTGAGTACTCATCATCTAGT
GTTCGATGGTATTTCCCTATAAGCTTTGTTGTGAATGGCGGGCTAGTTGGACTAGTCATA
AATGGTGTATCCAACAAACATCCAGGGATTATATTTGAGGATTGCAAAATGTTGAAGCAT
GTTGATCTGAGTTATTGGCGGTTATTAGAGGAAACCCCTGACTTCTCTGCAGCATTAAAC
CTTGAAAAATTATATCTTTTAAGTTGCAAACGTTTAAAAATGATTCATGGATCTGTTGCT
TCTCTTAGTAAGCTTGTTACCTTGGACCTCGAAGGCTGTGAAAATCTAGAAAAGCTTCCA
AGTAGCTTCCTCATGTTAAAGTCTCTTGAAGTTTTGAATCTAAGTGGATGCATAAAGCTA
AAAGAAATTCCTGACTTATCGGCATCGTCAAACCTTAAAGAACTACATCTTAGAGAATGC
TATCACTTGAGAATAATTCACGACTCTGCAGTTGGACGCTTTCTTGATAAACTTGTTATC
CTGGACCTTGAAGGATGTAAAATTCTTGAAAGGCTTCCAAGATACATCAGCAACTCAAAG
TCTATTGAAGTTATGAATCTTGATTCATGCCGAAAGATCGAACAACTTTTTGACAACTAT
TTTGAAAAGTTTCCAAGCCACCTCAAGTTCGAATCTCTTAAAGTTTTGAATCTTAGTTAT
TGTCAAAATCTTAAGGAAATTACTGACTTTTCAATTGCATCAAACCTTGAGATATTTGAT
CTTAGGGGCTGCTTCTCTTTAAGAACGATTCACAAGTCTGTTGGATCTCTCGATCAACTT
ATTGCCTTAAAACTTGATTTTTGCCATCAACTTGAAGAGCTTCCTAGTTGCCTCAGATTG
AAGTCTCTTGATTCTTTGAGTCTCACTAACTGTTATAAGATTGAACAACTTCCAGAATTT
GATGAAAATATGAAGTCTTTGAGGGAGATGAATTTGAAAGGTACAGCCATAAGGAAGTTA
CCCACATCAATTAGATATCTTATTGGGCTTGAGAATTTGATCCTTAGTTATTGCACAAAC
CTGATTTCTCTTCCAAGTGAAATTCATTTGTTAAAGAGTCTTAAGGAACTTGATCTTCGA
GAGTGTTCTAGACTCGACATGCTTCCCTCGGGATCAAGCTTAAACTTTCCCCAACGAAGC
TTATGTTCAAACTTGACTATATTGGATCTACAAAATTGCAACATATCAAATTCAGATTTT
TTGGAAAATTTATCTAATTTCTGCACTACCTTGAAGGAGCTAAATTTGTCCGGAAACAAA
TTCTGTTGTCTACCTTCTCTCAAAAATTTTACATCATTGAGGCTTCTTGAACTAAGAAAT
TGTAAGTTTCTTCGAAACATTGTGAAGATTCCACATTGTTTAAAACGAATGGATGCTAGT
GGTTGCGAATTGTTGGTAATAAGCCCCGACTACATTGCCGATATGATGTTCAGAAATCAG
GACCTTAAGTTAAGGAACTTCAAAAGAGAGCTAATCGTAACGTACAGCGAGATCCCGAAA
TTCTGCAACAATCAAACCACAGAAACTTATGATGCCTACTATGGAAAGTTGGTGTGGGTC
AAAATCAGAGCATATGTTGTTACTAAGAACTCCTCCATCACAGTTGATATGCTTGAATGA

>Cucsa.303290
ATGGAAATGAATATTTGGGACCTAGAGAAAAGTTTGCACGGTTCGAAGCCTTCCTTGCTGCGATTTGCTAACTGGCTTCG
TGCGGAGATGGAGGTTCATGGGATGAGTTGCTTTGTGTCTGATAGAGCAAAATGTAGAAATTCTCGCAAGCATAGAGTTA
TTGAGAGGGCAATGGATGCGTCCTCGTTTGGAGTCGTGATTCTGACAAAGAAATCATTCCAAAATCCTTACACCATTGAG
GAGCTTCGATTTTTCTCTGGCAAGAAGAACTTGGTCCCAATATTTTTTGATTTGAGCCCAGGAGATTGTCTTGCCAGAGA
CATAGTGGAGAAGAGGGGAGATTTGTGGGAAAAACATGGAGGTGATTTGTGGATTCTGTATGGAGGATTGGAGAAGGAAT
GGAAAGAAGCTATTGAGGGGCTTTGTCGGGTCGACGAGTGGAAATTTGAAGCTCAGAATGGTAACTGGAGGGATTGCATT
CTGAAAGCCGTCATGCTACTTGCAATGAGGTTGGGAAGGCGAAGTGTTGTCGAGCATTTGACTAAGTGGAGGGAGAAGGT
AGAGAAAGAAGAGTTTCCTTTCCCCCGAAATGAGAATTTCATAGGCAGGAAGAAAGAATTGTCAGAGCTAGAATTCATAC
TTTTTGGCAATATAGCTGGTGATTCTGAAAGAGATTACTTTGAACTGAAAGCTCGGCCAAGACGAAAAAATTTGACCCTA
GGCTGGAGTAAAAGTAGTTCATTAGAAGAGAAACAGAGGGAGCTACCATTAGAAGTGCGCAACAAGAAGGGTAAAGAACC
TATTGTGTGGAAGGAGTCAGAAAAGGAGATCGAAATGCAGAGTATTGAATTTCCTCAACGGCATCGGCGACTGAAAACAA
AAAGTGGTGAAAGGTATGCCAAGAGAAAAAGAACAGCCAAAATTTTGTATGGGAAAGGCATTGCTTGCATTTCAGGGGAC
TCCGGAATTGGTAAGACTGAGCTTCTCTTAGAATTTGCATATAGAAATCACCAAAAGTACAAGATGGTATTATGGATTGG
AGGCGAAAGCCGATATATCAGGCAGAATTACCTTAACTTAGGTTCCTTCCTAGAAGTTGATGTAGGATTTGGAAATTTCT
CAGGTAAAAGCAAGATAAAAAATTTTGAGGAGCAGGAAGAGGCAGCCATTTCAAGAATTCGCACAGAGCTGATGAGAAAC
GTTCCCTTTTTGTTGATAATCGATAATTTAGAGTGTGAAAAGGATTGGTGGGATCACAAACTTGTGATGGATCTTCTTCC
TCGTTTTGGTGGAGAGACACATATCATAATATCCACTCGACTTCCTCGTGTAATGAATTTGGAGCCTCTGAAACTATCTT
ACCTATCAGGGGCTGAAGCAATGTGTTTAATGCAAGGAAGCCTTCGAGACTACTCAATGGCGGAGATTGACGTTTTGAGA
GTCATTGAAGAGAAAGTTGGAAGGCTAACATTGGGCCTTGCAATAATTGGCGCAATCTTATCTGAGCTTCCCATCACACC
CACTAGATTGTTGGATACAACCAATAGAATGCCCTTCAAAGATCAATCATGGAGCGGTAGGGAAGCTCATGTATTTCGGC
GAAATACCTTCCTTATGCAGCTTTTTGAAGTATGTTTCTCTATTTTCGACCATGCTGACGGGCCACGGAGCTTAGCAACT
AGAATGGCTCTAGCAAGTGGGTGGTTTGGTCCAGCTGCTATTCCCATTTCGCAGTTAACCCTTGCAGCACACAAGATACC
AGAAAAGCGACAGCGGACGAGGTTGTGGAGAAAGTTGTTACGTTCCATGGCTTGTGGTTTAACTTCATCTTACATTAAAA
AGTCAGAAGCCGAAGCAACTTCCATGTTGTTAAGGTTTAATATGGCCAGAAGCAGCACCAAACAGGGATGCCTACATTTC
AACGATCTAGTCAAGCTATATGCACGCAAGAGAGGGGTGAACGGATTTGCCCAAGCAATGGTTCAAGCTGTCATGAACCG
TCCATTCATCATTCATCACTCAGAACATATATGGGCAGCATGTTTCTTACTGTTTGGGTTTGGTCGGGACCCTGTAGTTG
TCGAGCTCAAAGTGTCGGAGCTATTATACTTAATAAAAGAAGTCGTTTTACCGCTTGCCATTAGGACATTTCTCACGTTC
TCTCAGTGCACCACTGCATTAGAACTTCTCCGGTTATGCACAAATGCACTAGAAGCTGCAGACCAAGCCTTCGTTACCCC
GGTCGAGAAGTGGTTCGATAAGTCGCTTTGCTGGAGGCCAATTCAGACAAATGCACAGCTGAATCCTTATCTCTGGCAGG
AGTTAGCTCTATGTAGAGCAACACTGTTAGAAACCCGAGCAAGACTAATGCTAAGAGGTGGACAATTCGACATTGGGGAC

GATCTAATTCGAAAGGCGATCTTTATCAGAACTTCCATATCTGGTGAGGATCATCCAGACACAATATCTGCTCGTGAAAC
TCTCAGTAAACTCAATAGGCTTATTGCTAATTTCCATGTTCATTCTTCTCCATAG

>Cucsa.318890
TTAGTTTTCAACCATGAGTTGGTTAGACAACATTTTGATAAAACTGTTTGGGTTTGTGTCTCTGAACCATTTATTGTCAA
CAAGATTTTGCTAGATATTTTACAAAATCTAAAAGGCACCATTTCTAATGGAGGGGATAGTAAGGAAGTTTTACTTCGTG
AACTCCAAAAGAAGATGCATGGCCAAAGATATTTTCTTGTGCTTGACGATGTTTGGAACGAAAATTCTTTTCTATGGGAT
GAGTTGAAATACTGTTTGCTCAAGATCACTGGAAACTCTAAAAATAGTATTGTTGTGACTACAAGGAGTGCTGAAGTTGC
AAAAATCATGGGAACATGTTCTGGTCATCTTTTAAGTAAATTATCTGATGATCATTGTTGGTCCTTGTTTAAAGAAAGTG
CAAATGCATATGGATTATCAATGACTTCAAACTTGGAGATCATTCAAAAAGAGTTAGTCAAAAAAATTGGTGGTATACCA
TTGGCTGCACGAGTTTTGGGAAGGGCAGTAAAATTTGAAGGAGATGTTGAGAGATGGGAGGAAATGTTGAAAAATGTGTT
AAGCACTCCACTCAAAGAGGAAAATTTTATTTTGTCTATATTAAAATTAAGTGTGGATCGTCTACCGTCATCTGCATTAA
AGCAATGTTTTTCATATTGTTCAATTTTTCCCAAGGATTTTGTGTTTGAAAAACAAGAACTAATTCACATGTGGATGGCA
CAAGGTTTTCTTCAACCACAAGAAGGAAGGAACATGACAATGGAAACTGTAGGAGACATATACTTCAAGATCTTGTTGTC
ACACTGCTTATTTGAAGATGCCCATGAAACAAAGACAGAGGAATATGAGATACCTGATCTGCTTGAATTTGAAACAAGGC
CAGAAGAATATAAGATGCATGATCTTGTACATGATATTGCGATAGAAATTTCAAGAGATCAAAATTTGCAACTAAATCCT
AGCAATATATCAAAGAAGGAACTTCAAAAGGAGATTAAAAAGGTTGCATGCAAGTTACGCATGGTTGATTTCATTCGACG
GATTCCTTGCAATATAGGCCAACTAACATTTTTTGATGTTGAGATAAGGAACTTTGTTTGTTTGCGAGTTTTAAAGCTAT
CAACGCTGCCTAGTGATAAGTTACCGAAGTCAATTGGTCAATTGAAACACTTGAGATATCTAGAAATTGCATGTTATTTA
GGTAGATTAAAATTTCCAGAGTCTATTGTTTCTCTTCATAATTTGCAAACACTAAAGTTTCTATACTCATACGTTGAAAA
ATTTCCGATGAACTTTACAAATTTGACATTGTCTCATTTTGTGATCGGGTTTGAAGAAGGTTGTAAAATTACTGAATTGG
GTCCATTGAAAAACTTGCAAGGTTGTTTGAGTCTTTTGTGTTTGGAGAAAGTTGAAAGCAAAGAGGAAGCCAATGGAACA
AACTTGGCAGAAAAGGAGAAGTTAAAAGATCTACACTTAAGTTGGTCCAATGAAAGAAAAGATAACAACAATTACAATGA
TTTGGAAGTGTTGGAAGGACTTCAACCAAACCAAAATCTACAATCATTAGGAATCTACAACTTTGCAGAAAGACGTTTGC
CTAACAAGATTTTTGTTGAAAATTTAAGCGTGATAGGTTTGTATGGTTGTAATAATTGTGAAAAGCTTCCAATGCTTGGA
CAATTAAACAACCTAAAGAAACTTGAGATTTACAGCTTCCATGGCGTCCAAATTATAGACAACGAGTTCTATGGTAATGA
TCTAAACCAAAGAAGGTTCTTCCCAAAGCTTGAGATATTTGTAATGTGTGATATGATCAACTTAGAGCAATGGAAAGAAG
TAATGACAAATGATGCATCATCAAATGTTACAATCTTTTCCAATCTTAAATGCTTGGAAATACGTGGATGTCCCAAATTA
ACAAAACTTCCAAATGGACTACACTTTTGTAGCTCCATTCGACGTCACCTTCTTTCCCTTAAAAAAATTACTTTAGTCGA
GGATGAGTTGAGCAACAATAGTGTAACACAAATTTCTGAACAACTTCAACACCTCACTGCCTTGGAATTTCTGTCCATTG
AAAATTTTGGAGGCATTGAAGCTTTGCCAGAATGGTTAGGAAACTTTGTATGTTTGCAAACACTCAGTCTTTATAACTGC
AAAAATTTGAAAAAACTGCCTTCTACAAAAGCAATGCTACGTCTCACTAAATTAAATCAATTGTATGCTTGCAAATGTCC
GATGCTACTACTCGAAGAAGGTGATCCAGAGCGAGCAAAACTTTCCCACTTTCCAAACATGTTGGTTCAGCGCAACGGTT
ATCTGAAGTGTATTTAG

>Cucsa.326910
AAAATACCCATTTTACACCAACTTGATTACCTGTTCCACTACAAGCAAAATATTAAGGAAGTTGAAAAGAAAGTTGAAGC
TCTTGGGACTGCAAAAGGGAATGCAGTTTTTGATGGAGTTTCTAAATGGTTGACAATTGTAAAGGATGTATTGGAGATAG
CACAACAAAATGAAAACCCCTCATGCTTTAATTTTGTTGAACGATATCAATTATCCAGAAAAGCAAAAAAGAGGGTGGAA
AATATTATTGAACTCATAAATGAAGGAAATGGATTTAACAAAGATAATGTTGGTTATCCCGTACCTTCTCCCGATACTAA
TTCTCCCACTCTCCCTACCGATTATCAAATTATCGCGTCAAGAACTTCAATAGTAGAAGAAATAAAAGAGGCACTTGCAA
ACCCTAATGTCGACACAGTTGGAGTATGTGGTATGGGTGGTGTTGGAAAAACTGCTTTATTGAATGAAGTTAAGAAGTTA
GTGTTGGAAAAGAATTTGTTTGATCGAGTGATTCAAGTGGAAGTTGGTGAATCCAAAAGTGTATTCAATATTCAAGAACA
AATTAAAGATGAGTTAAATATGGAATTGAATATAGAATGTGAGGAGGTAAGAGCATGTCGCCTACGAACTCATATTGCCG
AGAGGAAAGAGAATATGTTGTTTATGTTGGATGATATATGGAAGGAACACGATGTTGAAAAAGAGTTTGGAATTCCTTTT
AAACATGTATTAAACAATGAAATGAACACAGAAAAGACATTTGAGGTGAATTCTCTTACCAACGAAGAGTCTAGGAACTT
TTTCGTGACAATAGTTGGTGAGTCTTCGTGTGTTGAAGATGGACATAATATACAACAAATAGCAGAGGATGTGGTAAAAG
AATGTGGAGGGTTACCACTTGCACTTAAAATTCTAGGAAAAGCATTGAAGGGAAAAAGAGTACAGATATGGAAGGATGCG
TTAAAGTCATTGAAAAATCCTGTTACAGTGACTATTTCAGGAGTGAGTGAGCAATTGTATTCTTGTCTCCAATTCAGTTA
CGATTCAACAGAAGATGAAGCAGAGCAAGTATTACTTCTATGTAGTGTATTTCCAGATGATTACAAGATTGAAGTGAAGG
ATTTGCAGATGTATGCAATGGGTATGGGATTGGTAAAGCACATAAATACTTGGGAAGATGCAGGGAATAGAGTAATCAAG
TTGGTTGATGATCTTAAATCTTGCTATTTGCTTCAAGATGAGCAGTCCAAGAAAGGATCAGATGATTGTGTTCAAATGCA
TGATGTGGTCCACGATTTTGCGAAATACGTTGCATCAAAGAAAGATAAGATGACGTCCTTGACGTATAGGAGTGGGCAAA
GGCTGGAATACTGGCAAGAAGAAGATGATGATATGCATGAATCTTATAAAGCAATTTATGCAGATTGTGCGAAATATTGT
GTATACCTTCCCCCAAAAGTTGGAGTTTCCGAACCTTCAATTGGTAATAAAATTCGAATTCCTACTGCATTTTTTGAAAG
AATGAAAGCGCTTCGAGTTTTGTCTGTGGAAACTATGAGTATAAGTTTTGAACCATCAAGTTGGGCATCAATTAACAACC
TTGAAGCATTATATGAATTGATAAAACTAAAGGTGCTACACGTGTTGAAATGCGACGACTTTAATCCGTCGGAGTTTCCT
CCAAACATCATTGAAAGTATGACACAACTGGAAGAGTTGAAATTTGATGGCTTCAAAATGAATGAACTTTCCGAATTGAA
CCGTCTGACACGCCTTTTCAGTTTAGAGTTAAGGATTCAGAATGTTGAGATCTTGTTAAACGAACTGAGTGTAGAAAAAG
CAGAAAAGTTGGAAGAATTTAGTTTTTGTGTTGATTCGGTGGGTTTTACAAACCTTCTTTGCAATAATAACCATACTGTT
CCATATGGAAATTATAATTGGTATCCCCGTTTGAAGGAACTTCAAATCTACATTCACAATAATCAAAATCAATACTTAGA
CATGCCACGTGGGATTGAAAATAACCCATGCATCTTGATCTTCAGTTGCAACAAATTAACATACGTATTTCCATCACATA
TGCTTACGTTACTTGTTTTCTTAAATACATTAGAAGTACATCATTGTAAGTTAGTAGAAAGGATATTTGAAATTGAAGAA

TGGAGTGGTAGTGGTGGTGCAGGTGATGTAAACCAAGTACTAGTCCCCTTCACGATCTTACACCTATCTTTTCTACCAAA
CTTAAAGCATGTTTGGAATACCGATCCAAATCCCACAACTTCAGACCTTCTTCCCCAATGTAAAGAAAGTGGAGATTACA
AAGTGTCCGACGCTTAA

>Cucsa.328080
ATGGCAGAATCCATTCTGTGCAGCCTTGCAGGAAGCATTATTACCAAATTGGGTTCTTTCGCACTTCAAGACCTTGGATT
GTTGTGGGGTTTCCATGATGAACTTGACAAACTGAAAGGCACTGTTTCCGCCCCTCGAAGCCGTACTTCTCGACGCAGAAG
AGAAGCAGTCCAAAAGTCGTGCAGTGAAGGACTGGATTTTAAAGCTTAAAGATACTTTCTACGACATCGACGATTTGTTG
GACGTGTTCTCCTATGAATCTTTGAAAAGACAAGTTATGACCAAACATAGAACTAACAACACCAAAAAAGTACGCATTTT
CTTCTCAAAATCTAATCAAATTGCATTTCGTTTGAAAATGAGTCAAAAAATCAAAAGGGTCCGAGAGAAATTAGATGCAA
TTGCTATGGATAAAACTCAATTCAATCTTTATGAGAATACTAGGGAAATACAAGATGATGAATCGACGAAACGACTGGAG
ACTACCTCTTTCATACGTGAAGGAGAAATAATTGGTCGGGATGATGACAAAAAAAGTATTATACATTATCTATTGGATAC
CAACATCCACGAAGATAGTGTTGCAGTGATTGCGATTATTGGAATGGGAGGATTAGGAAAGACTGCTCTTGTTCAATCTA
TTTATGGTGACGAGAAGGTAAAGAAACATTTTGAGTTGACAATGTGGGTGTGTATTTCTGAAGAATTTGATGTCAAAGTA
ATTATTGAAAAAATTATAGAGTCTCTCACAAAAAAGAAACGTGAGCCTGACCTTCAGCTTGATACGTTGCAAAGTATGGT
CCGAGAGAAAATTGATGGAAAAAGATACTTGCTTGTCATGGATGATGTGTGGAATGTTAATCGTGCAAAATGGATAAGTC
TAAAAAGGTATCTCATGGGTGGAGCTAAGGGAAGTAGGATTTTGATCACAACCCGTACTCATCAAGTTGCACAGACTTTT
GAAACAATTTTATCCCATCATTTAAAAGAACTAGATGAAGAAAAATCTTGGAAATTGTTTAGAAAAATGGCATTTTCCAA
CGAATCAGAGGTGCTTGAGAATTCAAAGTTGGTTGTAATTGGTAAGGAGATTGTTACAAAGTTGAAAGGTTCTCCTCTTG
CAATAAGAGTAATTGGGAGTTATCTCTATTCAAAAAAGTCAGAAAAGGATTGGTTGTCATTCAAGGACCATGAACTCGAC
ACAATCATGCAACAAGAAAATGAGATACAATCGATACTAAAGATCAGTTTTAACCACCTCTCATCTAGTTTGAAGCATTG
TTTCACATATTGTGCATTATTCTCCAAAGATTATCACTATGAAATTCGAAAAAATGATTTGATAAAACAATGGATGGCAC
AAGGCTTCATTCAACCACATAATAAGAAGGCAATGGAAGATGTTGGTGATGATTATTTCGAAGAACTACTGGGGAGATCA
TTTTTTCAAGACATAAGAAAAAACAAATGGGGAGAGATCAAGAAGTTCAAGATGCACGACATCATACACGATCTTGCATG
TTCTGTTGTAGAAAATGACTGTGTACTTGCTAATGATGACACTAAGTCCATTGACAAAAGGACTCGACATGTGTCAATTT
CGGCCTTCAACTCAATGACAAGATGGAAACTCATTACAAAATCATTAATAGAGGCAAAGAATTTGAGAACATTGAATTAT
GCTCGTCGTCATCACATCGATCTCTCTAATCATTTGCGGTTACGAACATTGAATTTGGAGTTTCATTTTGTTCCCAAGTG
TATTGGTAAGATGAAACATTTGAGATATATTAATATTACTTACTGTTATATTGATTTCCTTCCCAAGGCAGTTACAAAAC
TGTACCATTTGGAAACACTCATCATTCGTGGATGTCTCGAGCTAAGAGAATTGTCAAGTGATATTAAGAATCTTATCAAT
CTTAGACATCTAGATATTAAGGATTTTAAACATGTTTGGAGTTATATGCCAAAAGGAATGGGTTCAATGACTACCCTTCA
AACAATGAATTTGTTTATATTGGGAGAGAATAAAGGTGGTGAGTTAAGTGAACTCAATGGATTGGTCAACTTGAGAGGAT
CATTAAGTATTCAACAATTGCAGTTCTGCAAACCCATTGGCTTAGAAAATGTTAAATACCTTGAAGAAAAATCCAGAATT
CAAAAGTTGGAATTACATTGGAAGACCTATCAAAGGGAATCTAAAATTGATGATGAAGATGAGAGAGTTTTGGAGAGCTT
GAAACCACATTCAAATCTTCAAAAAATACGCATAGAAGGATATAGAGGATTGAAGTTATGTAACTGGTTCTCATTTGATT
CTATTGTGAATTTGGTCTTTATAAAGCTTTTCAACTGTGAAAAATTGCAACAGCTTCCTCGATTTGATCGATTTCCTTTT
CTCAAACATCTTCATCTGGAAGATTTACCGAGTATCGAGTATATTGCTATTAACAATTATGTTTCTTCATCAATGACTAC
TTTCTTTCCATCCCTTGAGAATCTAAGCATCATTAAGTTGCCTAACTTGAAAGAATGGTGGAAGGGGGAAAGCATTGATC
AAAATACTTCATTTCCAACAATTTTACGTCATCTTTCTCAACTAAAGATTCATTATTGTAGACAATTGGCTTCTATTCCA
CAACATGGACCTTTGCAATCATTGGACATACGTGATATCAGTTTGCAACTTTTTGAGTTGGTAATCAAAATGACCGCTAC
GAACATTATTTTTCTACCAAATGACTTATTCTCCAATGTGACACATCTCCAGTCTCTTGTCATAGGACGTTGCTTCAATT
TAAAAATGTCTTTTGATGATGATAATGTAAGATGGAAAGAACTCGGAAGTCTTCGCACACTTCGACTTTGTTTCATCCCC
AAATTAGAGTATTTGCCTAAGGGTTTCCAATATTTGAAAGCACTCGAACATTTGGAACTACTTTGGTGTGAAAATTTGGC
ATGTATTTTAGGGATTGAGCATCTCACTTCACTATCACGATTGGAAATTTCAAATTGTCCTAATTTAACTTCTTTGCCGG
AAGGGATGACTCAACTTATTTCATTAACATGTTTGATAATCGATGATTGTCCCAATTTAAGTACCTTGCCAGAAGGGCTT
CATCACCTCCTTAATACCCCGAGGTACGCACCACTAATTTTTTCCCACTAA

>Cucsa.337180
ATGGCTTGTTGCATCTATGAGCAAGCAGAAAATATCTTGATAGAGCTAAAAAAATTCCCAATGTACCTGAGACGAATGCA
GTATACCATGTTGAGCCTTAAAACAATTCTTAAGGATGCTGAAAAGGAAGAATATCGTCATTGTCTAAATGATTGGCTAC
AGAAGCTTCAAAGTGTATTTTTACAAATTGAGGAATTGCTATATGAATCCAATAGGGAAGTCAAAAAACAAGAGGCTACT
GGAAAATGGGTATTTCTTCCTTCCTTTAACTTCAGTCAAATTGATCAAACTAAAAAAATGATGAAACTATGCGACGATTT
GGATGAAATTGCATCCCATATGTATGGCTTCAATCTAACAAACATGGAGACAACACACTCCTTTCTTAGTGCTACTGAAG
TTTCGACAAGACTCATGAAACCAAGTTGGCAATTGCTTTACTCGTTGACTAATGCTCCCAAGGTTTTCCAAGACAAACGA
TATCATAACTTTCTGGATCATTTCAAAAAATCTACTCACGGGCTCTTCCACATAGTTGGAGAACCAGGTATAGGTAAGAC
CACACTTGCCAAATTCTTTTACAACAATCTGGTGAACACGTTTCCATCAAGATTGTGGATTTGTGTGAAAGAGGAATTTG
ATCCACAGAGATTGATAAAAGAGATGCTCAGTTTTTCACATTGCCAAGTAACATGTGATAACTTGACTGAGAAACAATTG
TGCTTTGCAGTTCAACAATTTCTGAGGGATAAAAAATTTCTGATTGTTTTTCAAGATATTTCAATCAAAAACCTTGGTAA
TTGCTCCATATTTAAAAGTTTATTGGGGATGGGAAACCGTGGCAGCAAAATCATAGTGACCACTCAGAATGAGAAAATAG
CTGATGCTGTCGGACTAAAAAAACTCTACAAGAACGAGAGCCAGGTAGTTCCATCCCCAGAAGCCACAAAACCTTCAGAT
GTTAACAAGGATAATATGAAACATCAAACAATTTTCAAAGTTGAGAGGTTGTCAAAGGAAAATTCATTGTCTTTGTTCAA
AGTTCATGCTTTCACAGAAACACAGGAAGCACAAATCCCAAATCTCACAAAAAATACAAGAAGTAATTGAGCAGAAATGTC
ATGGGGTTCCTTTGGCAATAAAGTGCCTGGGGGGTCTGCTATCAAAAACTAGTATAGCTGAGTGGAACGGTGTCATCGAT
AAGTTATGGGAACATGAGGAAGAGGAGGATGGGAATAAGAGTATTTTACCTACACTTAGATTATGCTATGATCAAATGCC

TTCACACCTACAACGTTGTTTTCTTTATTGTTCCCAATTAAAAAAAGATCGCATATTGTCTTCCAATGATGTGATTCAAT
TATGGATTGCAAGCGACCTCCTACCCAAAGAGAATTACTTATCTTTGGAAAAAATAGGTGAGAATTATTTCAAGGAACTA
TGCTCAAGATGTTTCCTACAAGAACTAGAGGAATATGGTTTTGGCTATTGGTTTAAATTGCACCCTCTTATTGAAAAACT
TGCACGTCTACTCACACAAAAACAGGTATTCGAAGTCACAAAAACCCAATCTATAGCCTTCACAATAAGAGATAAGGTGC
CCCCTAGTGCATTCCTAGCAAATGCATGCATCGACAAGTTCAAATACTTAAGACTATTGCATTTAGGCAATGCAAATCTA
CAGGGAATTCCAAGTGCTGTAGAAAATCTGGTACAGCTCAGATACCTAGACTTGCAAGGGAATAAGAAAATCAAGCGGCT
ACCAAATTCAATCTTCAAGCTAAAAAATTTACAAACCTTGATTCTTGCATCCTGTTCCGCACTTAAAGAACTGCCCAATG
ATATTAGGCAATTGACCAACCTGAGATACCTCTGGGTAACAGCAAACAACCTTCGTCTGCACAAAAATGGAGTTGGAACC
ATGACTTCTCTTCGATTTCTCGCAATTGGAGGTTGCAAAAAACTTACACTGACGTTGAAAGGAGTGGAGTTCAGGCTTCA
GAGGTTCACAATCAGAGAGCTTCCAATAGTGAAAAAATTGCCGGAATGGACTCAAAGATTCACCGAAACCCTAAGAGTTT
TGGAAATCATCGATTGTCCCATCGAATGGAATGATGATGTGTTAAAATCATACAAATCACTTGAACGGTTTTCAATTCAT
GGAGCTGTGAGGACCAAAAACCAGATCGGGGGGTACAACATCGATTATCGTAATTTCGTTAGGAGTAGGAAAGTCAAGAA
GGAAGTGAAGACATGTGTCTACTACTAA

>Cucsa.337190
ATGGCTTATTGCATCTATTACCGAGCTGAAAACATCTTGAGTGAACTGAAAAACCTCCCAAACTACCCAAGAAGAATTGA
GTATACCATGTTGAGTCTTAAATCAATTCTTATGGATGCGGAAGAGAAGCAAGAACAGAGTCGTGGTCTACAGAATTGGC
TAGAGGAGCTTCAAAATGTATTTTCCCAAATTGAAGGCTTCATAGATGAACACAAAGAGGAAGCCTACGAGGGTATTGGT
AAACAGGTACTTGCTCCTTTCTCGTGCTCCAGTAATCAAATAGCACGTACTTGGAAAATGGAGAAACTATTCGACCATTT
GAATGAAGTTGCGGCAAAAATGTATGAATTTAATCTTACAGAAAGGCACACTGGTGCCATAAAAACGGAGACAACAAACT
CTTTCCTTACTGCTACTGAAGTTTCAACAAGACTCATGAAACCAAGCTGGAAAGTACTTTACCCCTTAACTAATGCTCCG
AAGTTTTATCAGGATGAGCGGTACCGTAAGATTCTGAATGATTTCAAAAACCCTACTCTAGGGTTCTTCCACATAGTTGG
AGAAGCAGGTATAGGTAAGAGCACACTTGCCAAATTCATTTACAATGATCCAGAAGTAGAAGGAATGTTTCCATCAAGAT
TGTGGGTTTGTGTGAAAGAGGAATTTGATACACAGAGATTGATGAAAGAGATACTCAACTTTTCATATTCTCCAGCAACT
TGTGACAATTTGACTACAAAATTGTGCCCCACAGATCAATATCTGAGAGAGAGAACTTTTCTGCTTGTTTTTCAAGACCT
TTCAATCAAGAACCTAGATAATTGTTCCCTGTTTACAAGTTTATTGATGATGGGAAAGCCTGGTAGCAAAATCATAGTGA
CCACTCAGAATGAGGAAATTGCAAATGCTATAGAACTAACAATGATTTACAAGGTTGGGCAACAATCAGAGCAAAATCGG
AGCCAGACAGCCCTAGACACGGTTACTAAAGAGACTGCAAATGTTAACAACGCCGACCAGTTCGTTCAAGCTAACCCTTT
GGGTAAGATAGATCAATCTATCCCTTCTCAAACAATATTCAAAGTTAAGAGGCTGTCAGAAAAAGATTCCCTTTCTTTAT
TCAAAGATTATGCTTCTACATATGAAGGTAATGAAAAAGATATAATGAAAACTCTGAAGAAATGTAATGGAATACCATTG
GCAATAAAGTGTCTGGGGAGCATGTTATCTCTAGGTCCTCCAGCAACTAAATGGATGGAGGATAATGAGCGACAAAAGGG
AGATAATGAGTCTTCTAGTACATTTAGTATACTTAAACTATGCTACAATGAGATGCCCTCACACCTGAAGCGTTGTTTTC
TTTATTGTTCTCAATTACCAAACGATAGCATACTGTCCTCAAATGATGTCATTCAGTTATGGATGGCAAATGGACTTCTC
CGTTCACGCCAAGAGAATTACTTATCCTTGGAAGACATAGGTGAGATTTATTTCAAAGAACTATGCTCAAGATGTTTCCT
TCAAGATGTTGAGGAATATGGTCTTGGCTATTGGTTTAAAATGCACCCTCTCATTCGGGAACTTGCACGCCTCGTGCAAA
AACGAACTAAGGACTTGATAAGCATTAAACCAGTCACCAATGTCACATCTATAGCCTTCCCAGTAAGAGATGAGGTGCCA
TCTAGTTCATTTCTAGCTGAAAAATGCATCTCAAAGTTCCAACACTTAAGATTATTGTATTTAGGCCACACAGATCTACA
GGAAATTCCAAATACTATAGAAACACTGAATCACCTAACATACCTCGACTTGCAGGGGAATAAGAACATCAAGCGGTTAC
CAAATGCAATCTGTAATCTACAACATTTGCAGACCTTGATTCTTGCATCTTGTTCTGCACTTGAAGAATTGCCAAAAGAT
ATATGCAAGTTGAGCAACCTCAGATACCTGTGGGTAACATCAAACAAGCTTCGTTTGCACAAAAATGGAGTAGGAACCAT
GACTTCTCTAAGATTCCTCGCAATTGGAGGATGTGACAAACTTCAAGATCTATTCGAACGGCCATCATGCCTTGTACGCC
TTGAAACCCTAATGATTTACGATTGTAACTCTTTGCAATTGTTGCCAAACGAGATGGGGTCTCTAATATCGTTACAGAAT
TTGGTGATATGGAGTTGCAAACAACTTACACTGAAGGGCTTAGAGAAAGTCGATTTCAGCCTCCAAAGATTCACAATCAG
AGAGCTTCCAGAAGTGAATAAATTGCCTGAATGGCTTCAAAGGTCGACAGAAACCCTAAGAGTCCTGGAAATCATCGATT
GTCCCATCAAAGTGGAGGAAGAGGGAATCAAAATACATCACTGGGAATTTGGTACGGCGTAG

>Cucsa.337200
ATGGCTCATTGCATCTACACCCAAGCTGAAAATATCTTGACTAAGCTAAAAGATTCCCCACCCTACCAAAAAAGAATAGA
GTATGCGATGTCGAGCCTTAAAGCGGTTCTTTTGGATGCTGAAGAGAAGCAAGAACAAAATCAACGTCTACAGAACTGGC
TAAAGGAACTTCAAAATGTCTTTTACCAAGTTGAGGACTCCATAGATGAATTCAAATGGGAAATCTTCAAACAAAAGGAC
ATTGGAAAACAGGTACTTGCCCCTTTCTCGTGCTCTAATCAAATTTCTGCAAATAAATTGAAACAGAAATGTAAAAGGAA
GCAAGTATGCGACGAACTCAATAACATTGCAACCAGAATGTATGAATTTCATCTCAAAGTAAAGCACATTGATTCCATAA
GTATGGAGACAACACACACTTTCCCTAGTGCTTCAGAAATTTCAATAAGACACCTGAAACCAAGTTGGCAATTGCTTTAC
CCTTTAATAGATGCTTCCAGAACATATGACGAAATATATGATGGAATTTTGAATGTTTTCAATGAATGTACTCATGTATT
CCACATAGTTGGGGAAGCCGGTATAGGTAAGAGCACAGTGGCCAGATTCTTGTACAATCATAACAATGTAGTTGGTAAGT
ATACTTCAAGATACTGGGTTTGTGTGGAGGAAGGCTTTAATAAACATAGATTGGTGAAGGAGGTTTACAGTCATGCAGAC
AATAAAGAAATTTGCGAGGACTTGACGACAGAACAATTGCTTTCTAAGGTTAAACGACTTCTGAAAGTGGAAACTTTTTT
GCTCGTCTTTCAAGACCTTTCAATCACCAACTTGAAAGATCGTTCCCCTTTAGTGTTAAATGAATTATTGGAGATGGGAC
AACATGGCAGCAAGATCATAGTGACCACACAAACTGAGGAAATTGCAAATTATTTACAAGACCGTGGCTACAAGACTGAG
AGACGATCAAAGGAAAATCTGGCGAATGGTGACAGAGTATCTGCACACAATCAGTCGTTAACAAAAAATACCCAGAACCA
AGCAGTTCCAGGTTCACAAATTGAGGAAACTGCAGACGCTATAGCAAACAAAATCTATGAACACAGCCTATCATTAACAG
AAGATGCTCAGAATCTAGAAGTTCCAATTCCACAAGTTACAGAAGATACTAATATTGGCTTGGGGATCAATCCAGATATC
CCACCGATAAAGCAAGATGATACGGAATATCAAACAATTTTCAAACTTGAGAAACTGTCAAAGCAAAGTTCATCTCGTTT

ATTCAAAGAATATGCTTTCAGAAACAGACAAGAAGTAGAAAATCCAGAACTCACTAAAATAGTTGATCAACTTTTGGAGA
AATGCATGGGAGTTCCTTTGGCAATAAAGTGTCTAGGAAGCTTGCTATCTTCAGAAACTAGCATAGCCAAGTGGAAAAAC
ATCGAGGAAAAGTTGTCGCTCCAAGAGAAAAAGGAAAACGGTATTTTACATGTACTCAGAGTTTGCTATGATCAAATGCC
CTCACAACTGAAGCCTTGTTTTCTGCATTGTTCTCAATTACCTAACGATCGCATAATTTCTTCAAATGATATGATTCAGT
TATGGATGGCAAATGGGCTCCTCCATTCACCTGAAGAGAAGAACTCAACTATGGAAAATATAGGTGAGAAGTACTTCATG
GAGCTATGGTCAAGATATTTCATTCAAGAAATTGAAGAACATGGGCTTGGCTACTGGATTAAATTGCACCCTCTCATCCA
AAAACTTGCACACAAAATCACACAAGAACAATCTGAGGGCTCGGGGGGCAACAATCATCCCAAAGAAGTCACTGAAATAA
GATCCATAGCCTTTCAAGAAAGAAATATGGTGCTACCTAATGCATCCCTAACTGAAAAGTGCATCTGGAAGTACAAAGGG
TTAAGATTGTTGTATTTAAGCAATGCAGACCTACAGGAAATTCCAAATTCCATAGGAACACTCAAGTACCTGAGATACCT
CGACTTGCATGGCAATACGAAAATCAAGCATCTACCAAATTCAATATGTAATCTACAAAGTTTGCAAACCTTAATTCTTG
GATCTTGTTCCGCACTTGAAGACCTGCCCAAGGATATAAGGAATTTGATCAGCCTGAGATACTTGTGGGTAACAACAAAC
AAGCTTCGTCTGGACAAAAACGGAGTTGGAACCATGAATTCTCTGCGGTTTCTTGCCATTGGAGGGTGCAATAACCTAGA
AAATCTATTTGAACGCCCAGATTGCCTTGCAGGCCTCGAAACCCTAATGATATATAATTGCACTACCTTGAAATTGTTGC
CAGACGAGATGAGATATCTAAAATCACTACAAAATTTGATGATTTGGAGTTGCAAGCAACTTACACTAAACTTAAAAGAA
GTGGAATTCAAGCTTCAAAGATTCACGATCAAAGAGCTTCCAAGAGTGGAAAGATTACCCCAATGGCTTGAAAACTCGGC
AGAAACTTTGAGAACCTTGCAGATCATCAATTGTCCCATAAGAATAATGGAACGACAGGGAATTGAAAAGTACGAAGCAG
TTGAAAATACCATAATCTATGGTGCTGTAAGGTTTGAAATGGCACCACCAGGTTACGACTTCGAACACCGGAATTTGGCA
GTGCGTAATGGGAATGAGGAGATGCATATATATCCTTAG

>Cucsa.338100
ATGGCTGAAATTGGAACTTTTGTTGTTCAGGAAGTTTTGAAGAGGATTGTAAAATATGGA
GCAGAGCAAATTGTTGTGGCATGGGAGTTGGAGAATGAGGTGTCCCTGTTGAAAGATAAG
TTACACGATGCTGATACAATCTTAGAAGACATCAACAGAAAGAAATCACACCCTGGTAAT
TCTGTGAAAAGATGGGTGGAAAAACTCGAAGATATTGTCCATGAAGCCGATGATCTACTG
GATGAGCTTGTTTATGAACATCTTCGACGAACAGTGGAGCATACTGAGAAATTTAGCAAG
GTAAGTGATTCAATCTCATCATCCATAAATTCTTTTTTGTTTCGTCGCAAGATGGCCAAG
AAAATCAAGAACATTACCGATACTTTAAATCAACATTATTGTGCGGCAAGTGCTTTTGGG
CTAGTTGGTGTGGAAACTGTCACAGAAATAGAGCTTGCGCTCAATCAGATTCGAGAGACA
ACCTCAATTCTTGACTTCCAAGTCGAAGGAAGGGAGGCTGAAGTTTTGGAGCTACTTAAA
TTGGCGATTGACTCTACCAATGAACATCATATGTCTGTGATATCCATCGTTGGAATGGGT
GGTCTTGGCAAAACAACTTTGGCCAAGATGATCTTCAATCATCGTGAAATTGAAGGACAT
TTTGATAAAACTATATGGGTTTGTGTGTCAAAACCATTTATTGTCACAAAAAATTTTGGAA
AAAATCTTTCAGGGTTTAACAAAAACTTGTAGTGGGTTGGAATCCAATAAGGAGGCCTTG
CTTGGGAGGCTGCGAAAGGAGATGCAAGACAAGAATTATTTTCTTGTGCTTGATGATGTT
TGGGATAATGAGAAACACTTGTGGGACGAGCTTAGAGGCTGTTTGAAACATATTGCTGGA
AAACCTGGAAATACTATTGTGATGACCACAAGGAATGAAGAAGTAGCGACGATGGTGGAG
CCAATTTCTATTTATCGTCTAAAAAAGTTATCCAATGATCAATGTTGGGCGTTGTTTAAA
GAAAGTGCAAATGCAAATCAGTTGCCAATGAATTCGAAGTTGGAGATTATGAAAAAGGAG
CTGGTTAAAAAAATGGGTGGTGTACCACTCGTGGCAAAAGTTTTAGGAGGTGCAGTCAAG
TTTGAAGAAACTGAACTTGAAGAGGAAGATCATGAGATCAGTTGGATGACAAAAGTTGAA
AGCATTGTAAGGAACATTTCATTAGAGGACAAAGATTTTGTTTTGTCCATATTAAAATTA
AGTGTGGATTCTTTACCAAATCCCGTGTTAAAGCAATGTGTTGCCTATTGCTCAAATTTT
TCCCAAGATTATGACTTTCAGAAAGATGACCTAATTAAAATGTGGATAGCACAAGGATTT
ATCCAACCCGGACAAGGAAGAGATAAGAACTTGCTAATGGAGGATATTGGAGAACAATAC
TTCAACTTCTTATTGTCTCGTTCCATATTTCAAGATGTCACTAGGGATGCGAATAAGAGA
ATTGTTGGGTTTAAGATGCATGATCTAATGCATGATATTGCTTGTGCAATTTCGAGTCAT
CAAAATGTAGAATCAAATCCAAATAATTTGAGTGGAAAAAAGTGTAAGAAAGTTACGCACG
TTGATTTGCAATGATGAAGTGATTAATTATTTGAATCAGAAAGACATTGTTTGTTTACGT
GTTTTAAAGGTTATTTTTCAATCGCATACGGATTTGTGGATTCCAATAGACAAGTTGATT
CATTTGAGATATCTTGATATTTCAGAATGTTCTATAAACAAGCTTCTTCTTGAATCCCTT
TCTCTTCTTTATAATCTACAAACGCTAAAGCTTGGACAAAGTGGTCTACCGAAGAATTTG
AGAAAATTGGTTAACTTAAGACATTTAGAATTTAAAATGTTTGGTGATACAGCAATGCCT
TCAGATATGGGCAACTTGATTCATCTTCAATCATTGTCTGGATTTTTAGTTGGGTTCGAG
AAGGGTTGTAAAATAGAAGAGCTTGGACCGTTGAAAAACCTGAAAGGTAAACTAACTCTT
ACAAATCTCTGGAGAGTGCAAAATAAAGATGAAGCTATGGCTGCAAAATTGGTGGAAAAG
AAGAACTTACGTCATCTAAACCTATGGTTTTTCGAAACCGATAAGAGAGGAGAAGATGAT
GAAGATGGTATAGTACAAGTGTTGGAAGGACTTCAACCACACAAAAACCTACAATCATTG
GAAATCCTTGGTTTTCGAGGAAAAGTTTTGCCTACTGGTATTTTTGTTGAAAATTTAGTA
AAGATACGTTTGGGTCATTTTGAAAGATGTGAAGTGCTTCCCATGCTTGGACAGTTGCCC
AATTTAAAGGAACTTGAGATTATGTACATGGAAAGTGTGAGAAGTATAGGGAATGAGTTC
TATGGAGTTGACTCCAGCCACCAAAATTCTGTTGCTTTTCCACAGTTAAAGAAAGTCAGC
ATTTATGAGATGATGAACCTAGAGCAATGGGATGAAGCAACGGTGGTTCTTGCATCAAAT
CTCTTTGGATGTCTAAAAGAAGTTAGGATTAGGAGATGTAATCCATTGGCAAAGTTGCCA

AGTGGGTTGGAAGGTTGCCATTCCCTTGAATATTTGAGCATCCGTGGTTGTTTTAATTTG
ATGCTAAATGTGCAAAATTTGCACAAATTATACCATTTAGAGATTGATGGGTTGAAAAGA
TTGCCAAAGGGAATGGACGGACTCACTCGCTTGAAAGAGTTGAAAATTGGAGGATGCATG
CAAAATTATGAGTTTAGTTCCGTCATACACTTGGCTTCTCAGCTTGTTGAACTTGAGTTG
TCTGGCCGTTATGGGTCAGTTGACACCCAACTTCCCCAACAACTTCAACACCTCACTAAC
TTGCAAGTATTAAAGATTACACAGTTTGATTGCATTGAAGCTCTGCCAGAATGGATTGGA
AACCTCATCTCTTTGAAAACATTGAAATGCTCCTATTGCTTTAAGTTGAAAGAATTACCT
TCGAGAGAGGCCATATTACGCCTAACCAAATTAGAAAATTTGGACATTTTTGAATGTCCA
AAGCTACTAGTTGGGGAAGGTGACCAGGAGAGGGCTAAGCTTTCCCATCTTCCATCAAAA
TGTGTTCATAAATCTGAGTAA

>Cucsa.338190
ATGGTTGGACTTCTCGACAGTGTGGCCGGAAATCTGCTCGGAAGGATAATCGAAGCCGCCGACCGACTAGAGTTTCGTGC
TATCCAAAGCGAATTGAAAAACCTCGAAACAGATGTGTTGAATCTTAAGGCCAGACTCCGAGACGCCGAGGAGAAGCAGG
CTAGTAATTGTGAACTCAATGAACTGCTTAAAAACCTCAAAAATGTGTTTTCAAGGGCAGACATTGCAATTGAGGAATTG
GAATGCGATTATTTGAAGTGGAGAGTGCAGAATCGAAAGAACGACGTTGACGATAAGGGATGCCAGTTCTCTTCTTGTTT
CTCCTCCAATTTCCTCATTTCTCCATTTAATACCGGCAGTAAATTCCAGGAAGATCTTAAAATAATTACCTCCGAATTAC
GTTCGATTGAGAAAGCCATGTCTAAATTCTCTCTGGTTGAAGATGAAGATGAATATATAAAAAAATTGAAGGGTGAAATG
ACTTTGCGGACCTCCATTACTGGTTCGCATGCTTTCGCTAGGCTTCTGCGCTTGAGGAGAGAGGCGATTCTCTCTAATGT
AGATTCCATTTTTGGTAGAGATAAAATACAAGAGAGTATCATTAAGGAACTTGTGAATGATGAACAAAAATCTCCCCGTA
TTCTTTCAATCCAAGGAGATGGAGGGATGGGAAAGACGGCTCTGGCCAAGTTAGTCTACAATGCAGACGAAGTGTTTGAT
CATTTTGACAAGAGAATGTGGGTATGCGTTTCTGAAGATTTTGATATCCGGAGAATCTTAAGGGAGGTTCTGATGTCTGC
AACTGGAGAAAATGTTACCACTGTTGCCTTAACCGAAAGTCGTTTACGAATCCGGCTCCAGCGGTACTTTTTTGGCAAAA
AAATCTTGCTTGTTTTGGATGATTTTGGGAATTTGGATCCCGAAAGAGTATCAGAACTGAAAAAAATCGTGAAGATGGGT
GTTGGTGGCAGCAAGATAATGATAACCACTCGCAGCGATGAAACTCTAAATGTTGCTACGACACACAAGATTGACAAACT
CGACGAGACGATATCTATGCAAATATTCGAAGATACATATGGAAGCGAAGGGCTTAGCGAAGGGCTTAGAGACGATTTGT
ATCTCAAAAACCTTGTGGCAGAATGTGGAGGAGCTCCTTTGGCAATCAAATGTTTGGCTGGACTGCTCTCTTCAAAACCG
AGCGATGGTGCTAAGAGTCCAAATGTCAAGGACTTGAGTGAGAAATGGAAACAGGAAGAGGCAAACAACGGTGGTGGCGT
TTTATGTGCACTAAGACTGAGTTATGATCTAATGCCATCTTATTTGAAACCTTGTTTTCTTTGCTTTTCAGTGTTGCCGA
AAGATAATGTGTTCTTCTCATTTGAGCTAATCCAGTTATGGATGGCACAAGGAATCCTTCCTTCGGGTACCAAAGATAAT
CCTGAAGAAGTTGGGGAGAAATATTTCAAGGAATTTCGGGATCGCCGTTTACTCGTTGATGTTGAGGAGCACACTCTTGG
ATATTGGTTCAAAATCCATAGCCTTGTACATGATCTTGCAGTCCAAAAGGCTACGGAACAAAAGAACCTCGGAAATTTTC
ATATGCTTTCATTTGTCGATTGCGACAGCATCCCTTCGTCGACAAACTATGATAACACTCGTTTTATTTCCATTCCCGTG
GTAGGAGGTGCGGGACCAAATATCAATAGTGACCTTTTCAAATGCATCACCCAGTTCAGGCAGCTAAGGTTTTTGTACTT
GTGCAACTCTTCTCTGGAAGAAATTCCAACCTCCATCGACACGCTGAAACATTTGAGGTGTTTAGATTTGCGAGGGAGTC
AACGTCTGAAGAGGTTGCCAGAATCAATTTGCAAACTACAGAGCCTACAGACTTTGGTTCTTGCATTCTGCTCAGAGCTT
GAAGAGCTTCCCAGAAACATAAAGAACTTGATCAGCCTCAGATTCTTATGGATACAAACAAAGCAAGCCCGCTTGGAAAA
AGATGAAATAGGAAGCTTAACATCCCTTCGTTTTCTCGCCATTGGAAGGAGTGAAAACTTGACTCACTTGTTTGAAGATA
TCAACAAACTCAATTCCCTCAAAACACTGATCATTTATGAGTGCAAATCGCTGCTAACACTGCCAAAAGGCTTGGAAAAC
ATGAAATCTATATGTAATATGGGAATATGGGAATGTGATCGGCTGAGATTTACATTCTCACTGGCTTCACTTCACCTCAA
GAAACTGATACTCAGAGAACTTACAGCAGTGTCCACTTTGCCTAATTGGCTGTCCAATTTGGATGGTACTTTAGAAGTGC
TAGAAATTGGAGAGTTCCCCACGCTAAGAAAATTGCCAATCTGGCTTTTAAACTTTTGGGAACTCCGAATTCTTGGGATC
TCCAACTGTCCTAAGTTGAAGCATGATTCCTTCCCTCCTGAGCTAAATTATTTTTGTGATAAGATTGAGGAGTTGAGGAT
CACATTTTGTGGGTCTTTGAGCAAGTCTTTGTTGAAAAAAAGCATGAAGGAAATTGAACCTGAAAGCCGGGTAATCTTTT
ACATCCATACCATTTATGTGGACTCCAAAAGAATGACGCCACCAGTAGAATCAACAGACGAACCTAAGGAAGCAGAGACA
AAACAGGATGATGCTTATAACAATGCAAGTCCTCCTGGGACTGAACAACCTTCAAAGACTAAACATGATGATGCGAATAA
CAATATGAGTCATCCTGGGATTGGACTACTTTCAGAGTCAAAACAGGAGCATACAAATAACAATATAAACGAGATTGAGA
CTGTTAAGGTTTGTTTGGGTGATAATGACCATGCTGAAGCTCACCAAGCTATG

>Cucsa.338650
ATGGCCGACGAGCTCCGACCTCAACACGGGAATTGGACTTACGATGTTTTCTTGAGTTTTAGAGGTGAAGATACTCGCAA
GAACTTCACTGATCATCTCTACTACGCATTCAAAGATGCAGGCATCAATGTGTTTCGAGACGATCCAGAGCTCGAACGGG
GTGAAGACATAAGTTCGGAGCTCGAGCGAGCGATCGAAGGGTCGAAGGTGGCAGTTGTCGTATTCTCGGAAAGGTATGCG
GAGTCGGGATGGTGTTTGGAGGAGTTGGTAAAGATCATGGAGTGCAGGAGGACTTTGAGACAACTGGTTTTCCCAATATT
TTATAATGTGGATCCTTCATGTGTGAGGAAGCAAAAGGGTGAATTTGAAGAGGCTTTTGTTAAACATGAAGTGCGTTATT
TTAGGGATATTGATAGAGTTCTTAAGTGGAGAATGGCTCTCACTGAAGCTGCTAATTTATCTGGTTGGGATTTGAGAAAC
ATTGCAAATGGACATGAAGCGAAGTTCATAAGGTTGATTGTTGAAAAGGTATCAAAGGAGGTGAACAGTAAATACTTATT
CATAGCTCTTTATCCAGTGGGAATTGAATCAAGACTCAAACTTCTTTTATCACATCTTCATATTGGTTCAAATGATGTTA
GGTTTGTAGGAATTTTGGGGATGGGAGGACTGGGTAAAACCACCGTTGCAAAAGCACTTTACAACCAGCTTTATCACAAC
TTTGAAGCCAAATGTTTCCTTTCCAATATCAAAGCTGAAACCTCCAATCTAATTCACTTACAAAAACAACTCCTCTCTTC
CATCACAAATTCTACCAACATCAATCTTGGAAACATCGACCAAGGAATCGCAGTGTTGCAAGAAAGACTTCGTTGCAAAA
GGCTTCTTTTGATATTAGACGATGTAGACGACTTAAGCCAGTTAACTGCATTAGCAACAAGTCGTGATTTGTTTGCTTCA
GGTAGTAGAATTATCATAACAACTCGAGATCGACATCTGCTAAATCAGCTTGAAGTAGACGAAATTTGTTCCATCGATGA

AATGGATGACGATGAAGCACTTGAACTCTTTAGTTGGCATGCTTTTCGCAATAGTTATCCATCAGAAACCTTTCATCAAC
TTTCGAAACAAGTGGTCACTTATTGTGGAGGATTGCCATTAGCTCTCGAAGTGTTGGGTTCTTTCCTTTTTGGTAGAAGT
AGAGAAGAATGGGAAGATACACTGAAGAAATTGAAGAAAATCCCAAACGATCAAATTCAAAAAAAGCTTAAAATAAGCTT
TGATGGGCTAAACGATCATACTTACAAAGATATATTTCTCGACGTGTCATGTTTCTTTATTGGAATGGAAAGAAACTACG
TTGAACAAATATTAGATGGGTGTGGATTTTTTCCAAGAATCGGAATTAGTGTTCTTCTTCAAAGATGTCTATTAACAATT
GGAGACAAAAACAGATTAATGATGCATGATTTGTTAAGAGATATGGGGAGAGAAATTGTTCGTGAAAATTTTCCAAAATA
CCCTGAGAGACATTCAAGACTTTTTCTTCATGAGGAAGTGCTTTCTGTTCTTACAAGACAAAAGGGAACTGATGCAACTG
AAGGCCTAAGTTTGAAGTTGCCAAGATTTAGCAAGCAGAAGTTGAGCACAAAAGCATTTAATGAAATGCAAAAATTGAGG
TTACTTCAACTTAATTTTGTTGATGTAAATGGAGATTTCAAGCATATTTCTGAAGAGATAAGATGGGTTTGTTGGCACGG
ATTTCCTTTGAAGTTTTTGCCTAAAGAATTTCATATGGACAAATTGGTTGCTATGGACTTGAGATATAGCCAAATCAGAT
TCTTTTGGAAGGAGTCTAAGTTTCTCAAGAATTTGAAGTTTCTTAATCTAGGCCATTCTCATTACTTAACCCACACTCCA
AATTTCTCCAAACTCCCCAATCTAGAGATACTCAGCCTCAAAGACTGCAAGAATTTGATTGAATTGCACCCTACAATTGG
AGAATTAAAAGCCCTCATTTCCCTAAACTTAAAAGATTGCAAATCCCTCAATTCACTTCCAAATAGTTTCTCCAACTTAA
AATCCTTACAAACTCTCATTATTTCAGGTTGTTCAAAGCTCAATAGTTTGCCAGAAGATTTAGGCGAAATTACATCATTA
ATAACTCTAATAGCTGATAACACACCAATCCAAAAAATCCCTAACACAATTATAAACTTAAAAAACCTCAAATATTTATC
TTTATGTGGGTGCAAAGGGTCACCATCAAAATCATCATTCTCTTCAATGATTTGGTCTTGGATTTCACCAAAGAAATTAT
CTCAAAACTACACATCAATTCTTCTCCCTTCTTCATTACAAGGCTTAAACTCCTTAAGAAAATTATGCCTTAAAAATTGT
AACTTGTCAAATAACACAATTCCAAAAGATATCGGGAGTTTGAGTTCTTTGAGAGAATTGGATTTGAGTGAGAATTTATT
CCACAGTTTGCCATCAACTATCAGTGGCCTTTTGAAACTTGAGACACTTTTGTTGGATAATTGCCCTGAACTTCAACTTA
TACCAAATTTGCCACCACATTTGAGTTCATTGTATGCATCAAACTGTACTTCATTGGAAAGGACTTCAGATTTGTCTAAT
GTGAAGAAAATGGGATCTTTGTCTATGAGTAATTGTCCTAAACTTATGGAGATTCCTGGCTTGGACAAATTATTGGATTC
TATTAGAGTTATTCACATGGAAGGATGTAGCAACATGTCCAATTCCTTCAAGGATACCATTCTACAGGGATGGACAGTTA
GTGGATTTGGAGGAGTATGTCTTCCAGGCAAAGAAGTTCCAGATTGGTTTGCATACAAAGATGAAGGTCACTCAATATTT
TTAGAATTGCCTCAGTATAATAATTCCAATTTAGAAGGCTTCATTGTTTGCATAGTTTACTGTTCTTGTTTTAACAACAC
AGTCTCAACTGACCTTCCAAGTTTATCAGTCATTAATTACACAAAATCTTCCATTACAACCAACAAACCTCTTACCAATG
ATGTAATAATGTCAACTCAAGATCACTTGTGGCAAGGCCATTTATCTAACAAAGCCTTCAAGATGGAACCTGGCGATGAA
GTCGAGATCATCGTTGATTTCGGTGCTGAAATCACCGTGAAGAAAATTGGCATCTCGCTTGTGTTTGACAAGTATGTCGA
TCAAACAATGTTAGAGTTTGCATCCACCTGTAATGATGATGATGTCGTCGTGGATAACCAAGATGAAACTGTAAGTGAAA
AGGATGGAGAAGTTGGGAGCAAGAGAGGTTTTGACGAGAATGATGATGAAGGATTGAAAAATTCATACCAAATTCCCAAA
AGGTTGAAGTGTGAGATTGATTCTAACATGAAAATTGATGAGGAGTAG

>Cucsa.338660
ATGGCCAACGAGTTCCAAGCTCAACATGGAGACTGGACGTACGATGTTTTCTTAAGTTTTAGAGGCGAAGATACTCGAAA
AAACTTCACAGATCATCTCTACTACGCATTGAAAGATGCAGGCATCAATGTCTTTCGAGACGACCCAGAGCTCCAACGAG
GCGAAGACATAAGTTCGGGGCTGGAGCGAGCAATCGAAGGATCGAAGGTGGCAGTTATCGTATTCTCGGAAAGGTATGCG
GAGTCGGGATGGTGTTTGGAGGAGTTGGTAAAGATCATGGAGTGTAGAAGGACTTTGAGACAAATGGTTTTGCCAGTATT
TTATAATGTGGATCCTTCATGTGTGAGGAAGCAAAAGGGTGAATTTGAAGAGGCTTTTGTTAAGCATGAAAAGGGTAAGG
ATATTGATAAAGTTCGTAGGTGGAGAATGGCTCTCACTGAAGCTGCTAATGTAGCTGGTTTGGGTTTGACACAAAATGCA
AATGGGCATGAGGCAGAATTCATAAGGTCAATTGTTAAAATGATATCAAAGGAGGTGAAGAGCAATTACTTATTCATAGC
TCTCTATCCAGTGGGAATTGAATCAAGAATCAAACTTGTTTTACCACATCTTCATATTGGTTCAAATGATGATGTTAAGT
TTGTAGGAATTTTGGGGATTGGAGGTTTGGGAAAAACCACCATTGCAAAAGCACTTTACAATCAACTTCATCACAACTTT
GAAGCTGCATGCTTCCTTGCTAATATCAAACAAACCCCCAACCAACCCAATGGTCTAGTTCACTTACAAAAACAACTCCT
CTCTTCGATTACAAATTCCAGTAACATCAATTTCGAAAACATGGATCGAGGAATCGTTGTGTTGCAAGAAAGCCTTCGTC
GCAAAAAGCTTCTTTTGATATTAGACGATGTAGACAAAATAAGCCAATTAACTGCATTAGCAACAAGACGTGAATGTTTC
GGTTCAGGTAGTAGAATTGTCATAACAACTCGACATCGACGTTTACTAAACCAGATTGAAGTAGATGGAATTTGTTCCAT
TGATGTAATGGATGACGCTGAAGCGCTCCAACTCTTTAGTTGGCATGCCTTTCACAATAGTTATCCTTCAGAAACTTTTC
ATCAACTTTCAAAACGTGTTGTTAATTATTGTGGAGGATTGCCATTAGCCCTTCAAGTGTTGGGCTGTTTCCTTTTTGGC
AGAAGTAGAGAAGAATGGCAAGATACATTGAAGAATTTGAAGAAAATTCTAGATGATCAAATTCAAATAAAGCCTAAAAT
AACCTTTGATACCCACAATGATCACACTTGTAAAGATATATATCTTGTGAACCAAATGTTAGATGGGTGGGGATCTTTTC
CAAGAATTGGTGACATAAACAGATTAGTGACAAGTGATTTGTTAAGAGATCATACCCAACTTTTTCTTCCGAAGGAAGTG
CGCCTTTCTGTACTTGGACCAAAGGTAAGAAAATAA

>Cucsa.368510
ATGCTGGCTTTTACGGTTGAAAGTGTCGGTAGCTTGATTGTTGGTTCCTCTTTTGTGCTCAAACTTGACACTGCCCTTGA
GCTTCTTAGTTGGTATGCTTTGAAGGATAAGTCCCCATCAAGCAGTTACTTAGAGCTTGCAGAAGTTGCGGTAGGTTATT
GTCAAGGACTTCCTTTGGCTCTTGTTCTTTTGGACGAACTTGTCATCTCCCTCGAGAAAGGTGTTCAAGATATCTTCCAA
ATAAGTTATGAGGGGCTTGAATACAGATTGAAGGAACTCTTCGTTGATATTTGTTGCTTGTTCGTTGGGGATGATGTTTA
CTGCATGAAGAGTATGTTGAAGGCACGTGATCTCAATCCAGATTATGGAATTACAGTTCTCAATGATCTTTCTCTTATTT
CTATTGAAAATGGTAAGGTGCAAATGCATGATTTAATACCACAAATGGGTCATACCATTGTTCGTGGTGAATCATTTGAC
CTTGAAAATAGGAGTAGGTTGTGGGTAGCGAAGGAAGGAACAGATGCAGTTAAAGTCATAAAGCTGGACTTATCCAACGA
CTTATCACCGACATCCATTGATGCACAAGCATTTAAAAACATGAAAAATCTTAAATTGCTTATCCTTCGAAATATAACAT
TTTCTTCACATATGTTTGAGTATGGTCTTAATAAGTTCAAGTGGGTTTTATCTAGCTTTGGAATTAGATTTTCATCTTTG
CCTGCAAGCTTTTTGGTGAAAGAGGGGCTTGTTGGACTAGACATGCAACATTGTTGCATCAAATATTTGGGGAATAGATT

TGAGAATTGTGAACGGTTGAAGCATGTTGATCTCAGTTACTGTGAGCTTTTGGAAGAAATTCCTGACTTATCTGTTGCAG
TAAACCTTGAGTATTTGTACCTGAGAGGGTGCATAAGCTTAAAAACGATTCACGAGTCTGTTTCTTCTCTTAACAAGCTG
ATAACCTTAGACCTTGAGGGTTGTGTTAATCTTGAAAAGCTTCCTAGCTACCTCATGTTAAAGTCTCTTGATTCTTTCTG
TCTTTCGGACTGTCGTAAGCTTCAACGAGTTCCAGAATTTGATGAAAACATGAAATCTTTAACAAGGATGATTTTAGACT
ATACTGCCATAGAGGAGTTACCTTCATCGATTGAACATCTTGCTAAGCTCGAGTTTTTAAGCCTTAAAGGTTGTGCAAAC
CTCGTAGCTCTTCCAAGTGAAATTTATTTGTTACGAAGTCTTGTGGAACTTCGTCTTCCTGGATGTTCTAAACTCCACAT
GTTTCCTTGTCCAACTACCGAACAGATATTCCTTCTTTCGAAGCTGACTATATTGGACCTTAAAAATTGCAACCTATCAA
ATATAGATTTCTTGGAACCTTTATCTACTGATAGATTAAATGACTCAGGGTCAAGAGAGTTCATTCTAATGAATGGTATG
ATCCCAGAATGGTTCAACCATCAAACTACTACAACAAGTTCAATAAGCGTTAGCCTTCAACACTGTCCACAAAAGACACA
AGTGTTGGCTGCCTGTGTTATTTTCAAAGTAGATGGAGATTCATGTGAAGCGAAGGCTTCTATAAAATATGATATTTTCA
TTGATGGTGAATCACTAAAATATTTTGAGATCACCGTCCGGCCCTCATCGAAATCAGAATATATGTGGTTAATAACGACC
CCTCTAACATTTAGCTCTGTGAGCATGAGTTGTCAAGTCTCCTGTACGATTAACAAGACTTATGATAATGCTAGAGCAAC
CATAAGAAGTTTGGGCGTCCATATTGACGTTAGAGGGCAGCAAAGGCAAACTTG

>Cucsa.017450
MLGQRYFLVLDDVWNENSFLWNELKCCLLKITENSKNSIVVTTRSAEVAKIMETCHSYFLSKLSNDHCWSLFKESANAYG
LSNDFKLGDRSKRQCFSYCSIFPKDFVFEKQGLVKIWMAQGFLQPQEGRNVTMENVGDMYFKILLSQCLFQISSNSMRLK
FPESIVSLHNLQTLKFLHSKIEEFPMNFTNLASLRHLELWVSSDKTPLHLSRLTQLQILSHFVVGFEKGCKITELGRLKN
LQGSLSLLCSEKVESKEEANGANLAEKENLKELHLNWDMERKDNNSYNDLEVLEGLQPNQNLQSLIIHSFAERRLPNKIF
VENLRVIHLYSSFNCVKLPMLGQLNNLKELEIYSFLGVRIIDNEFYGNDPNQRRFFPKLEKFVMYEMINLEQWKEISRCP
KLLNIPKVFYENDMRNKSKLCYLNIGPLRKLPKDLCHLMNLREMEIVGNMHNYDFGILQHLPSLKQITLVEDELSNNSVT
QIPQQLQHLTALEFLSIKNFGGIEALPEWLGNFVCLQTLGLSNCKKLKKLPSTGAILRLTKLKKLYAYKCPQLLLDEEKK
R

>Cucsa.017460
MAEFLWTFAVQEILKKVLTLVAEQIILAREVKDVLQQLQKELVESQKIVSAITTQRQNHYSPDSLVTQWVNDLQLIVHEA
DDLLDFILNKNQPIERLWSVISLSCLLYSSNPETKKMKEIIALLNKHCTKLPHLLQLEPTPSNIAETEVAQIQETVSKPE
DYVVGRNREVETIVDRVIDASKQELNSILPVFGMGGLGKTTLAKSVFNHDRIKNHFGITIWIYVSQPFVINNILQAILQK
VEVHSSDCSNNREALLEKLTENMGEKTYFLVLDDVWNENKMLWEKLKECLMSITHMSGNSILVTTRSSGIAKMMEENIGS
HELRKLSDDQCWSIFRNFANAKDVPMTSNLEFVQKEFDKRIGGLPLIAKVLGAAVPFSGDHDQWVANIKSVLTTPIKEEE
FVKFTLKLSVDRLPNASVKQCFAYCSNFSKGCEFDKKQVIRMWMAQGFTQPDERNNETMEDTGERYFNILLSFCLFQDVV
KNERGIIEKVRMHDLIHDIACQVSNDKKLRIDHIISSNWKDWTKDDKILVSKLRTINFYDRHHVVVQDKIGDFTGLRVLT
IENYIVEELPNSIFKLKHLRYLDISYCYSIKKLPESIVLLYNLQTLRFHLLSKGFLPKNVGQMISLRHLEFSSIDKQMSP
YLSQLIQLETLPKFAVGFEKGCKITELGVLRNLKGLLKLQRLEHVESKEEAETAKLVEKENLEEVHFVWTKERKRKVENK
NDLEVLEGLQPPKNVEYLRIKYFLGGCLPNQTFVENLVKIELRDCGNCEKLPRLGQLGNLEILDISWFERVKSIGNEFYG
NSSNNQRSLFPRLKELYVDEMRRIGEWEEVGSNVKAFPRLERLYIGCCRDLVKIPDVFGYCDEYGEKHLEVVEIIEHLX

>Cucsa.017490
MDILVSVIAATIKPIGHQLGYLVCYNRNKKELREQLENLETTKKDVNQRVEEAKGKSYTI
SEEVSKWLADVDNAITHDELSNSNPSCFNLAQRYQLSRKREKQVNYILQLMNKRNSFVEV
GYRAPLPDTENTVVPGDYQVLESKTLLAKDIKNALSKPEVNKIGVYGMAGVGKTYFLNEV
KKLVLKGEDRLFDRVIDVRVGRFNDVTDIQEQIGDQLNVELPKSKEGRASFLRNNLAKME
GNILILLDDLWKEYDLLKEIGIPLSKDGCKVLITSRSQDILTNNMNTQECFQVSSLSEEE
SWKFFMAIIGDKFDTIYKKNIAKNVAKECGGLPLALDTIAKALKGKDMHHWEDALTKLRN
SIGMDIKGVSDKVYASLRLSYDHLDGEETKLIFLLCSVFPDDYKISIKNLQMYAMCMRLL
NKVKTWEDSKNRVMKLVNDLISSSLLLEAESDSKDKYVKMHDVVRDVAIHIASKEGNMST
LNIGYNKVNEWEDECRSGSHRAIFANCDNLNNLPLKMNFPQLELLILRVSYWLVEDNLQI
PYAFFDGMVKLKVLDLTGMCCLRPLWTTPSLNNLQALCMLRCEFNDIDTIGELKKLEVLR
IVKCNMLDHLPPTMSQLTHLKVLEVLNCPKLEVVPANIFSSMTKLEELKLQDSFCRWGEE
VWYKDRLVKNVTVSELNCLPCLSNLSLESWNVKILSEISSQTCKKLKEFWICSNESDDFI
QPKVSNEYARTLMLNIESQVGSIDEGLEILLQRSERLIVSDSKGNFINAMFKPNGNGYPC
LKYLWMIDENGNSEMAHLIGSDFTSLKYLIIFGMKRLENIVPRHISLSPFKKVKTIAIQF
CGQIRNLFSFSIFKDLLDLQEIEVINCGKMEGIIFMEIGDQLNICSCPLTSLQLENVDKL
TSFCTKDLIQESSQSIIPFFDGQVSFPELNDLSIVGGNNLETLWHKNNNPTTVLWSLNEL
HLLNLPNLKHVWRKDIIKILTFPSLKRVKIHGCTKLTHVWKDNNKVTRSFDSLERIEVEK
CKNLKYLLPSSIAFLNLKELHIKKCNGMINLFSSTVTKKLVNLSSIKVSYCKGMRCMVEV
DQAENDEIITFKKLSTLELDYLPRLDSFYSGKCMLEFPCLESLVIKRCPEMKTFSYGVII
APRLQTLWMNDKEFGVSSPACGINETIQNFPRRVVCMFNSN

>Cucsa.017500

LPTSMSELKKLKVLVVLDCSNLVVIHKNIISSMTKLEELDIQGRFTKWREKIVSVNPTKLSILLEGAEELVIVNDSKGFA
NNIFKAIGNGYPLLKYLKILENSETPHLRGNDFTSLEWLVLKGMVMLESIVPRHSPTNPFNKLKVVEITRCKQLRNFFSL
SIFKGLSNLREIKISECDMMEEIVSIEIEDQITICTSPLTSLHICHMNKLASFCSTKSSIQQTIVPFFDERRVSFPQLED
LSIFRANNLEMLWHKNGTSFSKLQTVEISYCKKLRCVFPSNIVTSLVSLDTLEINCCGLLEMIFEIEKPKTSCDTKVVVP
LRHLYLQVLPSLKYVWDKDGDDVVAFPNLKKVEVSRCPKLRSIFPPSFTKYMKEIEELIVEDEPIFPVEDEASKLKEVAL
FQSLKTLKMSCKEVVDERFWVMSKFSKLKRLELVGCEDDDDDDDDDDKMISLPMEMSEVSYSIEELTIRGCLQLVQVFGN
DSYIQRCANLKNMNQMTATTFSKLVDLQVHDCNGMINLFSPSVAKNLANLKSVEICNCREMTSIVAAKAEEEEENVEIVF
NNLTNMEFDNLERLEWFYSRKCRFEFPILDTLRIDKCYDMKIFSYGINKHSHFGKDLDWRVELLYSNTTNTRDK

>Cucsa.041670
MDSDGVESESTPAISTCLTIKIAPTSSKPPGTSSDLALPELKSSIESSPYNSPSLLSPPSSAFVSALQSPYISPRAVVLK
PEEKPIPAESTAALTCHSPLVSQSEDIPSSSYTPPSDQYEYSDDPSDSKVQFVACVPVPDSAPPRISFSFPVPRTSFAKC
GGPLSPVSTSKLRSCDVYIGFHGQANGLIRFCKWLKSELELQGIACFIADRSKYSDNQSHEIADRVISSVTFGVVVLTSS
SFHNHFTLEEVRFFAQKKNLIPFFFDMESSEISSFLNYNSMDKEYKETVQGLLRFHEYKLEANEGNWRSCIAKAAGILRG
KLGRMSTESDVERYEELPFPRNRCFLGREKEIMEMEATLFGNRSYHKQDGTVSTLIVEGNSSQQSEGLADEESEPVSVRG
SRFINLEIGRSDNPTLETWIEPVKGRNSFKRSKHKEMVKSGNHKSMSSSIVCINGNPGIGKTELALEFAYRYSQRYKMVL
WVGGEARYFRQNILNLSLNLGLDISADAEKDRGRFRSFEEQEQEAFKRVKRELFGDMPYLLIIDNLEAEEDWWEGKDLND
LLPRNTGGSHVIITTRLSKVMSFRMINIHPLALADAMVLMRGRRKKEYPADELEYLKKFDERLGRLTYGLWVIGSLLCEL
AITPSSLFEAIEQVPIDECSPCPYISINEEHYCKSNPFLMKIIYFSFSILEQTNGPLASGIFLVGAWLAPAPISVSVLAT
AAKDMAVSRKGFKIWSKYLSFMFGCCSTCLASQAWKSEEESALLLIKFGLARKANKQTGSWIQFHPITQVFAKRKEGLSA
AKSIVQGIRKCSSNTMANLDHLWASAFLVFGFKSEPPFVQLKAVDMVLYIKKAALPLAIRAFTTFSRCNSALELLKVCTN
ALEEVEKSFVSQIQDWCEGSLCWKKKFQGYQRVDEYVWQDVTLLKATLLETRAKLLLRGGHFDSAEELCRTCISIRTVML
GHNHAQTLAAQETLAKIVRLRSKI

>Cucsa.088220
MDILVSVTAKIAEYTVVPVGRQLGYVIHIHANFQKLKTQVEKLKDTRESVQQNIYTARRNAEDIKPAVEKWLKNVDDFVR
ESDKILANEGGHGRLCSTNLVQRHKLSRKASKMAYEVNEMKNEGEGFNTVSYKNAIPSVDCSLQKVSDFLDLDSRKLTAE
QIMDALSDDNVHRIGVYGMGGVGKTMLVKEILRKIVESKSFDEVVTSTISQTPDFKSIQGQLADKLGLKFERETIEGRAP
SLRKRLKMERRILVVLDDIWEYIDLETIGIPSVEDHTGCKILFTSRNKHLISNQMCANQIFEIKVLGENESWNLFKAMAG
KIVEASDLKPIAIQVVRECAGLPIAITTVAKALRNKPSDIWNDALDQLKSVDVFMTNIGEMDKKVYLSLKLSYDCLGYEE
VKLLFLLCSMFPEDFSIDMEELHVYAMGMGFLHGVDTVVKGRRRIKKLVDDLISSSLLQQYSEYGYNYVKMHDMVRDVAI
FIASKNDHIRTLSYVKRLDEEWKEERLLGNHTVVSIHGLHYPLPKLMLPKVQLLRLDGQWLNNTYVSVVQTFFEEMKELK
GLVLEKMNISLLQRPFDLYFLANIRVLRLRGCELGSIDMIGELKRLEILDLSGSNIIQIPTTMGQLTQLKVLNLSNCFNK
LEIIPPNILSKLTKLEELRMGTFGSWEGEEWYEGRKNASLSELRFLPHLFDLDLTIQDEKIMPKHLFSAEELNLEKFHIT
IGCKRERVKNYDGIIKMNYSRILEVKMESEMCLDDWIKFLLKRSEEVHLEGSICSKVLNSELLDANGFLHLKNLWIFYNS
DIQHFIHEKNKPLRKCLSKLEFLYLKNLENLESVIHGYNHGESPLNNLKNVIVWNCNKLKTLFLNCMLDDVLNLEEIEIN
YCKKMEVMITVKENEETTNHVEFTHLKSLCLWTLPQLHKFCSKVSNTINTCESFFSEEVSLPNLEKLKIWCTKDLKKIWS
NNVLIPNSFSKLKEIDIYSCNNLQKALFSPNMMSILTCLKVLRIEDCKLLEGIFEVQEPISVVEASPIALQTLSELKLYK
LPNLEYVWSKDSCELQSLVNIKRLTMDECPRLRREYSVKILKQLEALSIDIKQLMEVIGKKKSTDYNRLESKQLETSSSK
VEVLQLGDGSELFPKLKTLKLYGFVEDNSTHLPMEIVQNLYQFEKFELEGAFIEEILPSNILIPMKKQYNARRSKTSQRS
WVLSKLPKLRHLGSECSQKNNDSILQDLTSLSISECGGLSSLVSSSVSFTNLTFLKLNKCDGLTHLLDPSMATTLVQLKQ
LRIGECKRMSRIIEGGSSGEEDGNDASFNFHV

>Cucsa.089350
MNRASGSSSSSPFRCSFDVFLSFRGEDTRSNFTSHLNMALRQRGINVFIDNKISRGEEIS
ASLLEAIEKSKILIVIISENYASSSWCLNELEKIIMCNELRSGQLVLPIFYRVDPSEVRK
QSGRFGEEFGRLEVRFSSDKMQAWREAMIYVSQMSGWPVLQEEYFSSSHISFYSFALHII
SLLYFHYISSSDEANLIQEIVQEVFKKLNRGIIMQLRIPKYPVGIDIQVNNILFQIMSDK
KIVMLGLYGIGGIGKTTLAKALYNRIAHDFEGCCFLEKIREASNQYDGLVQLQKKILCDI
LMDNSINVSNLDIGVNIIRNRLCSKKILLILDDVDTREQLEALAGGHDWFGHGSKIIATT
RNMQLLASHGFNKLEKVNGLNAIEGLELFSWHAFNNCHPSSDYLDLSKRAVHYCKDLPLA
LEVLGSFLNSIHDQSKFERILDEYKNFYLDKDIQDILRISYDELEQDVKDIFLYISCCFV
GEDINEVKMKLEACGCLCLEKGTTKLMNLSLLTIESNRIKMHDLIQQMGRSIHLSKTFTS
HKRKRLLIKDDAMDVLNGNKEARAVKVIKLDFPRPTQLDIDSRAFEKVKNLVVLDVRNVT
SSKGTDLEYLPSSIRWMNWPQFPFSYLHTSFTIENLVKFNLPYSSIKKFGKALMCGEWLK
EINLSYSKFLVEIPDLTTAINLEKLNLEGCEKLVKVHESVGSLSKLVEFYLSSSVEGFEK
FPSCLKLNSLEALVVRYCRIEECCPQFSEEMNSLEILEIDDSIINQLSPTIEYLTGLKEL
WITECTKLETLPKILKVPKGVVRMDTRGCVSLAKFPNNIPDFISCDDNVEYDTKDGVIKQ
LILMNCDIPDWCKYKSMNNSVTFDFLADYLSWKRKAFIALCVKFHVTNDHELVKLNCGVL
FINDIEVWSRMSISNFNFWLSRGECLWMAVLHPCMHRLINPYGDDIMDISPNFSIGILDN
KITLLFEVNPECKDT

>Cucsa.091460

MGSSVVGDESFSSSPNFNYDYDVFFSFRGEDTRSNFISHLHMALRLKEVNVFIDDKLKRGEQIYESLLKFIERSRLSLVI
FSKDYASSTWCLDELVKIIECKKSKGQAVWPVFYKVDPSEVRKQTGGFGEALAKHEANKLLANKIQPWREALTFAAGLSG
WDLANSKDEAELIQKIVKRVLSAVNPMQLLHVAKHQVGVDSRLRKIEELVSHIGSEGVNLVGLYGIGGIGKTTLAKALYN
KIATQFEGCCFLQDVRREASKHGLVQLQETLLNEILKEDLKVIVSRDRGINIIRSRLCSKKVLIVLDDVNDLEQLEALVG
GRDWFGQGSKIIVTTRNEHLLSSHGFDEKHKIQELNQDHALELFSWHAFKKSHPSSNYLDFSKRATSYCKGLSLALVVLG
SFLCGRAKEEWNGILDEFENSLRKDIKDVLQLSFDGLEDKIKDIFLDISCLFVGEEYKCAKKMLSACHLNIDFGIMILMD
LSLITVEMDRVQMHELIQQMGRSIVHNESSEPGKRSRLWLEHDIWEVFVNNSGTDAVKAIKLDLPKSTRLNVDPRAFGSM
KNLRLLIIRNARFCTKIRYLPNSLKWIEWHGFAHRTLPSCFITKNLVGLDLQHSLIKRFGKRLKVSSRNSEPSSKHTNFE
CEWL

>Cucsa.091470
MDSSTVATESPTFKWTYDVFLSFRGEDTRTNFTSHLDMALRQKGVNVFIDNKLERGEQISESLFKSIQEASISIVIFSQN
YASSSWCLDELVNIIECKKSKGQNVFPVFYKVDPSDIRKQTGSFGEALAKHQPKFQTKTQIWREALTTAANLSGWNLGTR
KEADLIGDLVKKVLSVLNRTCTPLYVAKYPVGIDSKLEYMKLRSHNLFEKSNKFHYRKQHEYESDTGVYMVGLYGIGGIG
KTTLAKALYNKIASQFEACCFLSNVREASKQFNGLAQLQETLLYEILTVDLKVINLDRGINIIRNRLCLKKVLIVLDDVD
KLEQLEALVGGRDWFGQGSRIIVTTRNKHLLSSHGFDEMENILGLDEDEAIELFSWHAFKKNHPSSNYLDLSKRATSYCK
GHSLALVVLGSFLCTRDQVEWCSILDEFENSLNKDIKDILQLSFDGLEDKVKDIFLDISCLLVGEKVKYVKNMLSACHVN
LDFGIIVLTDLSFITIENDIMQMHDLIKQMGHKIVCGESLELGKRSRLWLVQDVWEVLVNNSGTDAVKGIKLDFPNSTRL
DVDPQAFRKMKNLRLLIVQNARFSTKIEYLPDSLKWIKWHGFRQPTFPSFFTMKNLVGLDLQHSFIKTFGKRLEDCERLK
YVDLSYSTFLEKIPNFSAASNLEELYLTNCTNLGMIDKSVFSLDKLTVLNLDGCSNLKKLPRGYFMLSSLKKLNLSYCKK
LEKIPDLSSASNLTSLHIYECTNLRVIHESVGSLDKLEGLYLKQCTNLVKLPSYLSLKSLLCLSLSGCCKLESFPTIAKN
MKSLRTLDLDFTAIKELPSSIRYLTELWTLKLNGCTNLISLPNTIYLLRSLENLLLSGCSIFGILPSCLHKFMSLWNLEL
RNCKFLQEIPSLPESIQKMDACGCESLSRIPDNIVDIISKKQDLTMGEISREFLLTGIEIPEWFSYKTTSNLVSASFRHY
PDMERTLAACVSFKVNGNSSERGARISCNIFVCNRLYFSLSRPFLPSKSEYMWLVTTSLALGSMEVNDWNKVLVWFEVHE
AHSEVNATITRYGVHVTEELHAIQTDVKWPMVNYADFYQLEKLQSLDIEELLLKRLFEEMSCWSNSQAMLYAANYDPEAI
IDSNIQPMIFPLHVTYNGETFICGMEGMGDTTLANSLCNKFNRSNDNGWPREALDDSTSFLHFRGGKFYGGSWSLSHHRK
RGDGERGTNITTRTISSKRYLILFHKAGSYNHLFNFAGSHRLIAGSGSYDSLNGRGDVRLLIERVDTSLL

>Cucsa.091530
QNHLLSRSYDVFLSFRGEDTRDNFTSHLDMALRQKGVNVFIDDQLERGEQISETLFKSIHKTSISIVIFSENYASSTWCL
DELVEIIECKKSKGQEVLPIFYKVDPSDVRKQTGWFGGALAKHEANFMEKIPIWRDALTTAANLAGWDLGTIRKEADLIQ
VIVERVLSILNQTHTPLKVAEYPVGIDYKIESLYWTQEMYKSECVDMVGIYGIRGIGKTTLAKALYNKIASQFEGCCFLS
NDNNSSAFREFVLMNSGIPEWLSYQTTSDSIRVSFQHNRNTERTLATSVTFRVDGDSYQGMALVSCNIFIGCRLKSCYMR
KFPTSTSEYTWLVETFSATSSSSLEMNDWNDVVICFEAVNCPAVVTIRSYGVYFTEKVSGIQNDIKGPGATYTDFDQPDE
LRSSR

>Cucsa.091680
MNQAGGSSSSSCFRWSFDVFLSFRGEDTRSNFTSHLNMTLRQRGINVFIDKKLSRGEEIC
ASLLEAIEGSKISIVVISESYASSSWCLNELVKIIMCNKLRGQVVLPIFYKVDPSEVGKQ
SGRFGEEFAKLEVRFFNKMQAWKEALITVSHMSGWPVLQRDDEANLIQNIVQEVWKKLDR
ATMQLDVAKYPVGIDIQVRNLLPHVMSNGITMFGLYGVGGMGKTTIAKALYNKIADEFEG
CCFLSNIREASNQYGGLVQFQKELLCEILMDDSIKVSNLPRGITIIRNRLYSKKILLILD
DVDTREQLQALAGGHDWFGHGSKVIATTRNKQLLVTHGFDKMQNVGGLDYDEALELFSWH
CFRNSHPLNVYLELSKRAVDYCKGLPLALEVLGSFLHSIGDPSNFKRILDEYEKHYLDKD
IQDSLRISYDGLEDEGITKLMNLSLLTIGRFNRVEMHNIIQQMGRTIHLSETSKSHKRKR
LLIKDDAMDVLNGNKEARAVKVIKLNFPKPTKLDIDSRAFDKVKNLVVLEVGNATSSESS
TLEYLPSSLRWMNWPQFPFSSLPTTYTMENLIELKLPYSSIKHFGQGYMSCERLKEINLS
DSNLLVEIPDLSTAINLKYLNLVGCENLVKVHESIGSLSKLVALHFSSSVKGFEQFPSCL
KLKSLKFLSMKNCRIDEWCPQFSEEMKSIEYLSIGYSTVTYQLSPTIGYLTSLKHLSLYY
CKELTTLPKISKVPEGVICMSAAGSISLARFPNNLADFMSCDDSVEYCKGGELKQLVLMN
CHIPDWYRYKSMSDSLTFFLPADYLSWKWKPLFAPCVKFEVTNDDWFQKLECKVFINDIQ
VWSSEEVYANQKERSGMFGKVSPGEYMWLIVLDPHTRFQSYSDDIMDRRSLKIIDLNQLS
SEINSSQSILGKITVSFEVTPWYKDVVIKMCGVHVIMGE

>Cucsa.091690
MNRATGSSSSHLRLPFDVFLSFRGEDTRSNFTSHLHMALCQKGINVFIDDDKLPRGEEIC
TSLLKAIEESKISIVIISENYASSHWCLDELMKIIMCNKSNNRQVVFPVFYKVDPSHVRR
QRGVFGEEFAKLQVRFSNKMQAWSEALTFISTMSGWDLKNYENEASLIQIIVQEVRKKLK
NSATTELDVAKYPVGIDIQVSNLLPHVMSNEITMVGLYGIGGMGKTTLAKALYNKISDDF
EGCCFLANVREASNQYWGLVELQKTLIREILMDDSIKVSNVGIGISIIRDRLCSKKIILI
LDDIDTHEQLQALAGGHDWFGHGSKVIATTRNKQLLASHGFNILKRVNGLNAIEGLELFS
WHAFKNSHPSSDYLDVSKRAVHYCKGLPLALEVLGSFLNSIDDQSKFERILDEYENSYLD
KGIQDILRISYDELEQDVKEIFLYISCCFVHEDKNEVQMMLKECDSRFRLEMGIKKLTDL

SLLTIDKFNRVEMHDLIQQMGHTIHLLETSNSHKRKRLLFEKDVMDVLNGDMEARAVKVI
KLNFHQPTELDIDSRGFEKVKNLVVLKVHNVTSSKSLEYLPSSLRWMIWPKFPFSSLPST
YSLEKLTELSMPSSFIKHFGNGYLNCKWLKRINLNYSKFLEEISDLSSAINLEELNLSEC
KKLVRVHESVGSLGKLAKLELSSHPNGFTQFPSNLKLKSLQKLVMYECRIVESYPHFSEE
MKSSLKELRIQSCSVTKLSPTIGNLTGLQHLWIDVCKELTTLPKILKVPEGVIYMNAQGC
RSLARFPDNIAEFISCDSEYVDGKYKQLILMNNCDIPEWFHFKSTNNSITFPTTFNYPGW
KLKVLAACVKVQVHDPVNGYHRGGDLECEVFFKDILVWSSGDWTNYLGYDSRWLPLGASP
SEYTWFIVLNPHRDFSLDDWDDMMERSPETDLSQLCFGINSMEMDRNRSNDKWNSIGGSI
WKNFTVLFEPRPLSPDTTISIKGCGVHVIME

>Cucsa.091710
MDRASGSSSSSCFRWTFDVFLSFRGEDTRSNFTSHLNMALRQRGINVFIDNRISRGQEISASLFEAIEESKISIVIISQN
YASSSWCLNELVKIIMCKELRGQVVLPIFYKVNPSQVRKQNGAFGEAFAELEVRFFDKMQAWGEALTAVSHMSGWVRQFE
NLLSHVMIDGTRMVGLHGIGGMGKTTLAKTLYNRIADDFEGCCFLANIREASKQHEGLVRLQEKLLYEILMDDFIRVSDL
YKGINIIRNRLCSKKILLILDDIDTSEQLQVLAGGYDWFGYGSKVIVTTRNEHLLDIHGFNKLRSVPELNYGEALELFSW
HAFQCSSPPTEYLQLSKDAVNYCKNLPLALEVLGSFLYSTDQSKFKGILEEFAISNLDKDIQNLLQVSYDELEGDVQEMF
LFISCFFVGEDKTMVETMLKSCGCLCWEKGIQKLMNLSLLTINQWNKVEMHDLIQQLGHTIARSKTSISPSEKKLLVGDD
AMHVLDGIKDARAVKAIKLEFPKPTKLDIIDSTAFRKVKNLVVLKVKNVISPKISTLDFLPNSLRWMSWSEFPFSSFPSS
YSMENLIQLKLPHSAIQHFGRAFMHCERLKQLDLSNSFFLEEIPDLSAAINLENLSLSGCISLVKVHKSVGSLPKLIDLS
LSSHVYGFKQFPSPLRLKSLKRFSTDHCTILQGYPQFSQEMKSSLEDLWFQSSSITKLSSTIRYLTSLKDLTIVDCKKLT
TLPSTIYDLSKLTSIEVSQSDLSTFPSSYSCPSSLPLLTRLHLYENKITNLDFLETIAHAAPSLRELNLSNNNFSILPSC
IVNFKSLRFLETFDCKFLEEIPKIPEGLISLGAYHWPNLPTT

>Cucsa.091780
MGSSTAATESMAFEWSYDVFLSFRGEDTRTNFTSHLDMALRQKGVNVFIDDKLERGEQIS
ESLFKSIQEASISIVIFSQNYASSSWCLDELVNIIECKKSKGQNVFPVFYKVDPSDIRKQ
TGSFGEAMAKHQPKFQTKTQIWRKALTTAANLSGWDLGAYRREADLIRDLVKEVLSTINR
TRTPLYVAKYPVGIDSQLEYMKFHSHHLNKGNKFQYWTQNEYESDIGVYMVGIYGIGGLG
KTTLAKALYNKIASQFEGCCFLSNVRQASNQFNGLVQLQQNLLYEILEDDLKFVNLDKGI
TIIRNRLRSKKVLIVLDDVDKLEQLEALVGGRDWFGQGSKIIVTTRNSHLLSSHGFDEMH
NIQGLNQDRAIELFSWHAFKESHPSSNYLDLAERATSYCKGHPLALVVLGSFLCNRGQTE
WRREKYNNCAKKMLSACHLNVDFGIMILMDLSLVTIEKDRVQMHGLIQQMGHSIVHNESF
ESGKRSRLWSERDIWNVFVNNSGTDAIKAIKLDLPNPINVNVDPKAFFRSMKNLRLLIIR
NAQVCTKIKYLPNSLKWIEWQGFAHRTFPSCFITKNLVGLDLRHSFIKRFGKRLEDCERL
KHVDLSYSTLLEKIPDLSAASNLEELYLINCTNLGMIDKSVFSLNKLTVLNFKGCSNLKK
LPKGYFMFSSLKILNLSYCQELEKIPDLSSASNLQSLLLNGCTNLRVIHESVGSLNELVL
LDLGQCTNLSKLPSYLRLKSLVYLVLFGCGKLESFPTIAENMKSLRCLDLHSTAIKELPS
SLGYLTQLDKLHLTGCTNLISLPNTIYLLRNLNELHLGGCSRFEMFPHKWVPTIQPVCSP
SKMMEAASWSLEFPHLVVPNESICSHFTLLDLKSCNISNAKFLEILCDVAPFLSDLRLSE
NKFSSLPSCLHKFMSLSNLELRNCKFLQEIPNLPQNIRNLDASGCKSLARSPDNIVDIIS
IKQDLELGEILREFLLTDIEIPEWFSYKTASNLVTASLRHYPDMERTLAVAVSFKVNGDS
SESEAQISCNIFIYNKLRCLFSRSFLPSKSEYMWLVTISLACSLEVNDWNKVFVWFEVHE
AHGVTVTRYGVHVTEQLHGIQTDVKWPMVNYADFYQLEKLRRDLDFEDLKASLKKSAVQI
PKQHCMHFSMIQRQ

>Cucsa.091820
MGSSIVGAESSTSSSSSFKWSFDVFLSFRGDDTRSNFTGHLDMALRQKGVNVFIDDMLKRGEQISETLSKAIQEALISIV
IFSQNYASSSWCLDELVKIVECKKSKGQLVLPIFYKVDPSDVRKQTGCFGEALAKHQANFMEKTQIWRDALTTVANFSGW
DLGTRKEADFIQDLVKEVLSRLNCANGQLYVAKYPVGIDSQLEDMKLLSHQIRDAFDGVYMMGIYGIGGIGKTTLAKALY
NKIANQFEGFCFLSNVRETSKQFNGLVQLQEKLLYEILKFDLKIGNLDEGINIIRSRLRSKKVLIVLDDVDKLKQLEALV
GERDWFGHGSKIIVTTRNSHLLSSHEFDEKYGVRELSHGHSLELFSWHAFKKSHPSSNYLDLSKRATNYCKGHPLALVVL
GSFLCTRDQIKWRTILDEFENSLSEDIEHIIQISFDGLEEKIKEIFLDISCLFVGEKDCKRLKHVDLSYSSLLEKIPDFP
ATSNLEELYLNNCTNLRTIPKSVVSLGKLLTLDLDHCSNLIKLPSYLMLKSLKVLKLAYCKKLEKLPDFSTASNLEKLYL
KECTNLRMIHDSIGSLSKLVTLDLGKCSNLEKLPSYLTLKSLEYLNLAHCKKLEEIPDFSSALNLKSLYLEQCTNLRVIH
ESIGSLNSLVTLDLRQCTNLEKLPSYLKLKSLRHFELSGCHKLEMFPKIAENMKSLISLHLDSTAIRELPSSIGYLTALL
VLNLHGCTNLISLPSTIYLLKSLKHLYLGGCSRFQLFSHFLEILCNVAPFLSSILLSENKFSSLPPCLHKFMSLWNLQLR
NCKFLQEIPNLPHCIQKMDATGCTLLGRSPDNIMDIISSKQVPHFHFHFPLVGDSYQGMALVSCKIFIGYRLQSCFMRKF
PSSTSEYTWLVTTSSPTFSTSLEMNEWNHVTVWFEVVKCSEATVTIKCCGVHLTEEVHGIQNDVKGPGVVYTVFDQLDKL
PSRIRGMEGMAETTLANSICNKYERSRNLFSAKKALNHSTGFLCGDGNGLSWEMVDRPILSDRLSSQKYLRIFDDRDRYG
DLNDVAHGTGNRFRSRFLRMDDIKEDDIREEPYWKYMEX

>Cucsa.091840
MGSNAAGAESSSSSPINWIYDVFLSFRGEDTRSNFTSHLHMFLRHKGVNVFIDDRIERGEQISEALLKTIQCSLISIVIF

SENYASSTWCLDELVEIIECKKSKGQKVLPIFYKVDPSDVRKQNGCYGEGLAKHEANFMEKIPIWRNALTTAANLAGWDL
GTIRNEADLIQVIVKEVSSTLNVTTPSDKPLLVGIDSKIESLYWPTEEMYKSECVDMLGIYGIRGIGKTTLAKALYYKMA
SQFECCCFLSNVREASKQLNGLAQLQKKLLFQILKYDLEDVDDLDRRNNIIKHRLHSKKVLILLDDVDEMKQLKALAGGH
DWFGQGSKIIVTTRDKHLLDSHGFGQTYEVEGLWEHNAFELFCWHAFKKSHPSSNYLDLSERATRHCKGHPLALVVLASF
LCGRDQAEWSGLLDGFENSLRKGIKDVLQLSFDGLEDEVKKFFLDISCLLVGETVTYVKKMLSEFHSILDFKISNLRHLS
LIRMEEYDDDRVQMHDLIKQMGHKIVYDECGDEPGKRSRSGKRCSERHKVGVYLIPPRVINVNPEAFRSMKNLRILIVDG
NVRFCKKKKYVPNGLKWIKWHRFPHQTLPSCFITKDLLPSYLKLKSLTDLDLSGCRKLETFPEIDENMKSLERLRLSYTA
IRKLPSVFAGLVFLSVLLKCVPKFFHSRVPKEILFFEHLELLDLKGCNISNVDFLENLCNVALSLTSIVLSENEFCSLPS
CLHKFMSLRNLQLRNCMFLQEIPNLPQSIQIVDATGCISLRRSPNIMWT

>Cucsa.091880
MESIPISIIAKICEYTVKPVGRQLCYVCFIHSNFQKLKSQVEKLTDTKGSVEDKVFIARRNAEDIKPAVEKWLEKVDRLV
RKSEKILAHEGRHGRLCSTNLVQRHKASRKASKMADEVLEMKNQGESFDMVSFKGRISLVESPLPKAPDFLDFGSRKSTV
EQIMDALSDDNVHKIGVYGMGGVGKTMLVKEIVRKIEESKKSFDKVVTSTISQTPDFKRIQGQLADKIGLKFEQETIEGR
ATFLRRWLKAERRIPSVEDHKGICKILFTSRNKQLISNDMGANKIFEIKVLGEDESWNLFKAMAGEIVEATDLKPIAIQI
MRECAGLPIAITTVAKALLNKPSDIWNDALDQLKSVDVGMANIGEMDKKVYLSLKLSYDYLGYEEVKLLFLLCSMFPEDF
NIDVEKLHVYAMSMGFLRGVDTVVKGRRRIKKLVDDLISSSLLQQYSEYGNNYVKIHDMVRDVAILIASQNDHIRTLSYV
KRSNEEWKEEKLSGNHTVVFLIIQELDSPDFSKLMLPKVQLFVLFGPSPSIYNRHVVSVVETFYKEMKELEGLVIERVKI
SLSPQALYSFANLRLLRLHDCELGSIDMIGELKKLEILDFSKSNIVEIPMTFSKLTQLKVLNLSFCDELEVIPPNILSKL
TKLEELHLETFDSWEGEEWYEGRKNASLSELRYLPHLYALNLTIQDDEIMPKHLFLAGELNLENFHITIGCQRQKRHIDN
KTNFFRIKMESERCLDDWIKTLLKRSEEVHLKGSICSKVLHDANEFLHLKESLFSKLKSVVVTKCNKLEKLFFNCILDDI
LSLEEIAIHYCEKMEVMIVMENEEATNHIEFTHLKYLFLTYVPQLQKFCSKIEKFGQLSQDNSISNTVDIGIFEVQESSI
TDTSLIVLKNLRELKLYNLPNLEYVWSKNPCELLSFVNIKGLAIDECPRLRREYSVKILKQLERLTMDIKQLMEVIENQK
STDHNMVKSKQLETSSKDNSTHLPVEIVQILYQLEHFELEGAYIEEVFPSNILIPMKKQYYARSKNSVRSWFLSKLPKLR
HLWSECSQKNAFPILQDLNVIRISECGGLSSLVSSSVSFTNLTVLKVDKCDRLTYLLNPLVATTLVQLEELTLRECKMMS
SVIEGGSAEEDGNEETTNQIEFTHLKSLFLKDLPRLQKFYSKIETFGQLSRDNSENPETTTIHNRIGDSFFSEQESLPNL
ETLRIDGAENLRMIWSNNVLIPNSFSKLEEVEIYSCNNLQDVLFHPNIINMLTCLNTLRIKNCELLEGIFEVQEPISVTK
TKTNAIVLPNNLIELELYNLPNLEYLWSKNPNFERLVTFESIRSLSIEKCSKLKGEYFLSIKTFKQLVEVLQLRDGSKLF
SNLKELKLYGFVEYNSTHLPMEIVQVLNQLEKFELKGMFIEEIFPSNILIPSYMVLRKLTLSKLSKLRHLWGECSQKNND
SLLRDLTFLFISKCGGLSSLVSSSVSSFTNLRILEVEKCDGLSHLLSPSVATTLVHLEELRIEECKRMSSVIEGGSSEED
GNDEIIVFNNLQHLIISSCSNLTSFHCGRCIIQFPCLKQVYINKCTELKVFSLGIVSTPPLKYENIYLKNDDDDDTWHHP
KESIEMVVETDMNVIIREYWDDNIDTRISNLFGEE

>Cucsa.094560
MAEFLWTFAVQEVLKKVLKLAADQIGLAWGLDKELSNLSQWLLKAEAILGEINRKKLHPS
SVRLWVEDLQLVVHEADDLLDELVYEDLRTKVEKGPINKVRSSISSLSNIFIIFRFKMAK
KIKAIIQKLRKCYSEATPLGLVGEEFIQTENDLSQIRETISKLDDFEVVGREFEVSSIVK
QVVDASIDNVTSILPIVGMGGIGKTTLAKTIFNHEEIKGHFDETIWICVSEPFLINKILG
AILQMIKGVSSGLDNREALLRELQKVMRGKRYFLVLDDVWNENLALWTELKHCLLSFTEK
SGNAIIVTTRSFEVGKIMESTLSSHHLGKLSDEQCWSLFKKSANADELPKNLELKDLQEE
LVTRYLDISNSKIEELPNSISLLYNLQTLKLGSSMKDLPQNLSKLVSLRHLKFSMPQTPP
HLGRLTQLQTLSGFAVGFEKGFKIGELGFLKNLKGRLELSNLDRIKHKEEAMSSKLVEKN
LCELFLEWDMHILREGNNYNDFEVLEGLQPHKNLQFLSIINFAGQLLPPAIFVENLAVIH
LRHCVRCEILPMLGQLPNLEELNISYLLCLRSIGYEFYGNYYHPYSHKVLFPKLKKFVLS
QMPNLEQWEEVVFISKKDAIFPLLEDLNISFCPILTSIPNIFRRPLKKLHVYGCHEVTGL
PKDLQLCTSIEDLKIVGCRKMTLNVQNMDSLSRFSMNGLQKFPQGLANLKNLKEMTIIEC
SQDCDFSPLMQLSSLVKLHLVIFPGSVTEQLPQQLEHLIALRSLYINDFDGIEVLPEWLG
NLTSLEVLGLYYCINLKQFPSKKAMQCLTQLVHVDVHNCPSSQILSHDLKAKAHAKANLV
QW

>Cucsa.094580
MAIAEFLWTYAVQQVLKKVLELAADQIEKKLHHSSVRLWVADLLLVVHEADNLLDELVYEYLRTKVEKGSINKVCSSVSS
LSNIFIIFRFKMAKKIKSIIEKLRKCYYEATPLGLVGEEFIETENDLSQIRETISKLDDFEVVGREFEVSSIVKQVVDAS
NQYVTSILPIMGMGGIGKTTLAKTIFNHEEIKRHFDETIWICVSEPFLINKILGAILQMIKGVSSGLDNKEVLLQELQKV
MRGKRYFLVLDDVWNENIALWTELKKCLLCFTEKSGNGIIVTTRSIEVGKIMESTLPSHHLGKLFDEQCRSLFKESANAD
ELPMDPELKDLQEELVTRFGGVPFVARLGSSMKHLPYNLSKLVSLRHLKFSIPQTPPHLSRLTQLQTLSGFAVGFEKGCK
IEELGFLKNFKGRLELSNLNGIKHKEEAMSSKLVEKNLCELFLEWDLHILREGSNYNDLEVLKGLQPHKNLQFLSIINYA
GQILPPAIFVENLVVIHLRHCVRCETLPMLGELPNLEELNISNLHCLRCIGNEFYGSYDHPNNHKVLFRKLKKFVLSEMH
NLEQWEELVFTSRKDAIFPLLEDLNIRDCPILTNCPKIDLQLCTSIEDLKIVGCLEMILNVQNMHTLSRFSMNGLQKFPQ
GLSHLKNLKEMIITECSQDCDFTPLMQLSSLVNLDLVLFAGNGAVQLPQQLQHLTALRSLIINDFDGIEVLPEWLGNLAS
LEVLGLYYCRSLKQFPSKKAIAMSHPISPCGCLWLSTTTQVRRFCAMMF

>Cucsa.094650

MAEFLWTFAVEETLKRTVNVAAQKISLVWGLEDELSNLSKWLLDAGALLRDIDREILRKESVKRWADGLEDIVSEAEDLL
DELAYEDLRRKVETSSRVCNNFKFSSVLNPLVRHDMACKMKKITKMLKQHYRNSAPLGLVGKESMEKEDGGNNLRQIRET
TSILNFDVVGRETEVLDILRLVIDSSSNEYELPLLIVPIVGMGGVGKTTLAKLVFRHELIKKHFHETIWICVSEHFNIDE
ILVAILESLTDKVPTKREAVLRRLQKELLDKRCFLVLDDVWNESSKLWEELEDCLKEIVGKFGITIIVTTRLDEVANIMG
TVSGYRLEKLPEDHCWSLFKRSANANGVKMTPKLEAIRIKLLQKIDGIPLVAKVLGGAVEFEGDLDRWETTLESIVREIP
MKQKSYVLSILQLSVDRLPFVEKQCFAYCSIFPKDCEVVKENLIRMWIAQGFIQPTEGENTMEDLGEGHFNFLLSRSLFQ
DVVKDKYGRITHFKMHDLIHDVALAILSTRQKSVLDPTHWNGKTSRKLRTLLYNNQEIHHKVADCVFLRVLEVNSLHMMN
NLPDFIAKLKHLRYLDISSCSMWVMPHSVTTLFNLQTLKLGSIENLPMNLRNLVRLRHLEFHVYYNTRKMPSHMGELIHL
QILSWFVAGFEEGCKIEELGNLKNLKGQLQLSNLEQVRSKEEALAAKLVNKKNLRELTFEWSIDILRECSSYNDFEVLEG
LQPPKNLSSLKITNFGGKFLPAATFVENLVFLCLYGCTKCERLPMLGQLANLQELSICFMDSVRSIGSEFYGIDSNRRGY
FPKLKKFDFCWMCNLEQWELEVANHESNHFGSLQTLKLDRCGKLTKLPNGLECCKSVHEVIISNCPNLTLNVEEMHNLSV
LLIDGLKFLPKGLALHPNLKTIMIKGCIEDYDYSPFLNLPSLTKLYLNDGLGNATQLPKQLQHLTALKILAIENFYGIEV
LPEWLRKLTCLETLDLVHLVKSLPSLQGEDNRVAEIAYETGEMETYECDNKRSWFRLHLNFYGCTLVELV

>Cucsa.094660
MADFLWSFAVDEVLKKTVKLVAEQIGMSWGFKKDLSKLRDSLLMVEAILRDVNRIKAEHQALRLWVEKLEHIVFEADVLL
DELSYEDLRRKVDARPVRSFVSSSKNPLVFRLKMANKIKAIAKRLDEHYCAASIMGLVAITSKEVESEPSQILETDSFLD
EIGVIGREAEVLEIVNKLLELSKQEAALSVLPIVGIGGLGKTSLAKAIFHHEMIRENFDRMIWVCVSEPFVINKILRAIL
ETLNANFGGLDNKEALLQELQKLLRNKKYFLVLDDVWNENPDLWNELRACLLKANKKFGSVIVVTTRSDEVANIVETNHQ
RHRLRKLSNDYCWTLFEKCAFGSDLPVTPRVDHVIREELVKRFGGIPLVVKVFGGMVKLDKNKCCQGLRSTLENLIISPL
QYENSILSTIKLSVDRLPSSSLKQCFAYCSNFPRGFLFIREPLVQMWIAQGFIHLPSGSNVTMEDIGANYFNTLLSRSLF
QDVVKDDRERILYCKMHDVVHDVACAISNAQKLRLSGKSNGDKALSIGHEIRTLHCSENVVERFHLPTFDSHVFHNEISN
FTYLCVLIIHSWFIHQLPDSIAKLKHLRYLDISHSLIRTLPDSIVSLYNLQTLRLGSKIMHLPTKLRKLVNLRHLEFSLS
TQTKQMPQHLSRLLQLQTLSSFVVGFDKGCKIEELGPLNNLKGELSLFHLEHVKSKTEAMAANLAMKENISDLYFQWSLL
SEREDCSNNDLNVLEGLRPHKNLQALKIENFGGVLPNGLFVENLVEVILYDCKRCETLPMLGHLSKLELLHIRCLDSVKS
IGDEFYGNNNSYHNEWSSLLFPKLKTLHISQMKSLELWQEIGSSSNYGATFPHLESLSIVWCSKLMNIPNLFQVPPKLQS
LKIFYCEKLTKLPHWLNLCSSIENMVICNCPNVNNNSLPNLKSMPNLSSLSIQAFEKLPEGLATIHNLKRLDVYGELQGL
DWSPFMYLNSSIEILRDIDSLPEWLGNLTSLETLNLRYCKNLKSFPSIEAX

>Cucsa.094670
MDLLYSKNYEDKILSNLRDSLLMVEAILRDVDRIKAEHQAVKLWVEKLEAIIFEVDVLLDELAYEDLRRKVEPQKEMMVS
NFISFSKTPLVFRLKMANKIKNIAKMLERHYSAASTVGLVAILSKQTEPDFSQIQETDSFLDEYGVIGRESEVLEIVNVS
VDLSYRENLSVLPIVGMGGLGKTALAKVIFNHELIKGNFDRAVWVCVSEPFLIKKILRAILETLNSHFGGLDSKEALLQE
LQKLLNDKKYFLVLDDVWNENPILWNELKGCLLKISQRSGNVVVVTTRSDRVAEIMETHSRYHLTKLSDDHCWSLFKKYA
FGNELLRIPELDIVQKELVKRFGGIPLAVKVMGGIVKFDENHEGLQKSLENLMRLQLQDENHVVSTIKLTVDRLPLPSLK
QCFAYCSNFPKDFKFRKEALIQMWIAQGFIQPSLGSDEMMEDIGEKYFNVLLSRFLFQDIVKDNRGRIIFCKMHDLIHDV
ACAISNSPGLKWDPSDLFDGEPWRRQACFASLELKTPDCNENPSRKLHMLTFDSHVFHNKVTNFLYLRVLITHSWFICKL
PNSIAKLKHLRYLDISYSTIRELPDSAVLLYNLQTLKLSRFLNGLPKNLRKLVSLRHLEFFSDPCNPKQMPQHLGKLIQL
QTLSSFVVGFDDGCKIEELRSLRNLKGKLSLLCLERVKSKKEAMAANLVEKRNISYLSFYWALRCERSEGSNYNDLNVLE
GLQPHKNLQALRIQNFLGKLLPNVIFVENLVEIYLHECEMCETLPTLGQLSKLEVLELRCLYSVRSIGEEFYGNYLEKMI
LFPTLKAFHICEMINLENWEEIMVVSNGTIFSNLESFNIVCCPRLTSIPNLFASQHESSFPSLQHSAKLRSLKILGCESL
QKQPNGLEFCSSLENMWISNCSNLNYPPSLQNMQNLTSLSITEFRKLPDGLAQVCKLKSLSVHGYLQGYDWSPLVHLGSL
ENLVLVDLDGSGAIQLPQQLEQLTSLRSLHISHFSGIEALPEWFGNFTCLETLKLYNCVNLKDMASKEAMSKLTRLTSLR
VYGCPQLNTFMFNGMSPNSSTSSSRTLSTLE

>Cucsa.102240
MALELVGGAVLGAVVGELFKAILNLGERAISFNPVLKDIRSKLNAIMPLVKQIDELNDYLDYPKEETEKLRGLMDEGKQL
LLQCGDVKLGDLNYLKRPSYTQKLRELDTALRSFMDVLMLQMARDQKKNMKMMNQMMEIICRLDNRGGSSKPMDLFVPPC
LVPQLREETVGLEKPVKELKVKLLKNGVQMLVVTAPGGCGKTTLALKFCHDKEVKDIFQEKIFVPVSRKPDLKLILKDII
ESLRGIQLPDLQSDERAFCYLELWLKQTSVNRPVLIVLDDVWSGQESEVLLDKLFQLPCCKILVTSRFYFPRFSESYYLE
PLNHENAVQLFRRAASLDKGISKLPDDETVEKIIGGCKRLPLALKVIGRSLSHKPTSVWKVTGRNLARSGSIFDSDNELL
ECLQSSLDVLDDNMVTKKSFMDLGSFHEDQRISASTFIDMCTVLYTLDESEAMVTLDELSSRSLVNFVTARKYGYDDDFY
EEYSFTQHDILRDLAIHLMNMEPIEQRKRLILDINGNDLPKWWVDQEKHTSYARLISITTDKRFSASWPDMEAPEVEVLI
LNLQSRTYNLPGFIKRMNKLKVLIITYFGSFLTEVTSEDNQLLDSLTSLERIRFERISVPIFSNPNPKPLINLQKISFFM
CKFGQTFMDPSTPISDLLPNLLEISIDFCNNLSEVPNRLCEIVSLQKLSITNCHGLSSLPEDVGKLINLKNLRLRSCIHL
EEFPESTTKLRELVLLDISNCIGLAKLPEKIGEFHNLEKLDMRHCWSLSKLPLSIGKLKNVKFLCDREVGEWLRKVAPRL
AKQVKVQEEEANLEWLGF

>Cucsa.123410
MAVTDFFVGEIATELLRMMVQLSTKSCLCKTTAAQIANSIQQILPIIEEIKYSGVELPAHRQFQLDRFSETLRRGIEISE
KALQCGRLNIYRNLRLARKMEKLEKDICRFINGTMQAHILADVHHMRFQTTERFDRLEGVLLERRLESMKIRADASGEER
WWVEEAFKKAEEEERYESNFVNIGTGLRVGKRKLKELVIGKEDLTAVGISGIGGSGKTTLAREFCKDPEVRRHFKERILF
LTVSQSPDVEQLRRTIWEFVMGSDSVNSNNLILHGRPSNSALLVLDDVWSISVLENVIPNVTGCKTLVVSRFKFPEVLRE

TYEVELLKESEAIALFCHSAFGQQSIPLSANHNLVKQVVNECKCLPLALKVIGASLRGQSEMFWNNAKSRLSRGEPICES
HENKLLQRMAISIERLSSKVRECFLDLGCFPEDKRIPLDILINVWKELHDLDDEEALAVLFELSQKNLLTLVKDARGGDI
YSSYYEMYVTQHDVLRDLALHFSCQENVNDRKRLLMPKSDTELPKEWLRKSEQPFNAQLVSIHTGEMEEMDWAPMIFPEA
KVLILNFSSSGYFLPSFLCNMPKIRALIVLNNNATHATLTNFSVFSSLVNLRGIWLEKISMTQLFDACTPLKHLRKLSLV
FCKINNSLDEWAVDVSQIFPFLFELKIDHCNDLRKLPSSICEMQSLKCLSVTNCHNLSQLPTNLWKLKNLQILRLFACPL
LKTLSPSICVLSCLKYIDISQCVYLTSLPEEIGKLTSLEKIDMRECSLIRRLPRSVVSLQSLCHVICEEDVSWLWEDLKS
HMPNLYIQVAEKCFNLDWLKE

>Cucsa.128020
GASSKSHLLENWLQKLEEALYAVDVLDELSREALHREVMTRDKNAKQVRIFFSKSNQIAFNYRMARQIKKEVIGRNDDKK
KVKHLLLDDVWNESEEKWHGLKPLLMSGAKGSKIFITMRDSKIAAEIESMTSLFTLEGLPKSKSWSLFSKVASKEGKVLE
NSNLLQLGKEISVKCGGVPLFRHLRYPNLPENIMKSLPNSITELQNLQMLNLMYCSELMELSRDTRNLINLRHLDFNSST
LTHMPEEMGKLNCPQTLSYFVLDYERSNQLSEPTVLIHLKGDLRIKNLEQLSYNPSELSLVNLKDIKGFKNLELEWNLSP
DDQEYEGEDDETSAMEGLERHSNVESLHIDGYSGVGLPNWVSTLLLKLTRITIYKCHRLQHLTQIAHLQALTFLFLDDMS
SLEFIDKNEPSSSSSFPSLELLIIENMPNLEGWWELGNTQKNWLPPTFSTLISLHISRCPKFRFMPKPASTGTVVFLRDV
SVQLETTLDPLWGLECLTLEKIKDLKYLETMESQLNISSLPIQLRDLEINKCSNLMSLPEWISSITSLEELEIMKCPKLK
VAFASAVAPK

>Cucsa.128030
MAEAILFQVAGEILMKLSSQAFQRLGMLFGLKGDLNKLTTTVSTIKDVLLDAEGRQTKSHLLQNWLHKLEEALYDAEDVL
DELSTEALRRELMTRDHKNAKQVRIFFSKSNQIAFNYRMARQIKNIWERLDAIDAEKTQFHLRENCESRTQYGSFDRIMM
GRETWSSSNDEEVIGRDDDIKEVKERLLDMNMNVTHNVSFIAIAGMGGIGKTTLAKSLYNDEEVSGFFDLKIWVWVSDQF
EVQVVAEKMIESATKNNPSVKGMEALQAKLQKVIGERKYLLVMDDVWNESEEKWHGLKSLLMGGARGSKVLITKRDRKVA
TEIKSMTSLFTLEGLSESNSWLLFSKVAFKEGKESTDPSTIHLGKEILVRCGGVPLVIRHVGRMLYSKTSQEEWMSFKDN
ELLEVIQQDNDMTSILKLSYNHLPPNLKRCFAYSSLFPKGYKIEIKDLIRQWVAQGFIEVSNGRKSLEDTGKDYFNELCW
RFFYANSSDECNINDIVCMHDVMCEFVRKVAGNKLYVRGNPNNDYVVSEQTLHISFDYGIQSWQDVLSKLCKAKGLRTIL
LLFRPYEKMNKIDKAILDELFSSFPRLRVLDLHFSQISVVPKSIKKLRHLRYLDLSENDMELIPHSIIELQNLQTLNLTE
CYELKELPRDIDNLVNLRHLTFEPCMEVTPTSEGMEKLTCLQTISLFVFDCKKTNKLWELNDLSYLTGELKIIGLEKLRS
SPSEITLINLKDKKGWQGLNLEWKLGKDEYEGEADETIMEGLEPHPNVESLSINGYTGGALPNWVFNSLMKLTEIEIENC
PRVQHLPQFNQLQDLRALHLVGLRSLEFIDKSDPYSSSVFFPSLKFLRLEDMPNLEGWWELGESKVVARETSGKAKWLPP
TFPQLSSMPKLASIGADVILHDIGVQMVSTIGPVSSFMFLSMHGMTNLKYLWEEFQQDLVSSSTSTMSSPISLRYLTISG
CPYLMSLPEWIGVLTSLETLHIKECPKLKSLPEGMQQLKSLKELHIEDCPELEDRCKQGGEDWPNISHVPNFTYKNASDI
DTPQSSSGFSHHPFSIVRISVI

>Cucsa.128100
MADSVLFNVAASVITKLGSSALRELGSLWGVNDELDKLQNTLSAIKAVLLDAEEQQSKSHTVKDWIAKIKDVFYDIDDLI
DEFSYETLRRQVLTKDRTITKQVRIFFSKSNQIAFGFKMGQTIKKVREKLDAIAAIKAQLHLSVCAREVRDNEPRKVRET
SSFIPEGEIIGRDEDRKSVMDFLLNTSNITKDNVEVVSIVGMGGLGKTALAQTVYNDEKINNRFKWKIWVCISQEFDIKV
IVEKILESITKTKQESLQLDILQSMLQEKIYGKKYLLVMDDVWNVDHEKWIGLKRFLMGGASGSKILVTTRNLQTAQASD
TVWFHHLKELDKDNSWALFRKMAFLNKEEELENSNLVRIGKEIVAKLKGYPLSIRVVGRLLYFKNTEMDWSSFKDNELDS
ILQEDDQIQPILKISFNHLPPKLKQCFTYCALFPKDYEFKKNGLVKQWMAQGFIQAHNKKAIEDVGDDYFQELVGRSFFQ
DIRKNKWGDLKYCKMHDLLHDLACSIGENECVVVSDDVGSIDKRTRHASFLLSKRLTREVVSKSSIEVTSLRTLDIDSRA
SFRSFKKTCHMNLFQLRTLNLDRCCCHPPKFVDKLKHLRYLNLSGLNVTFLPNSITTLYNLETLILRYCLWLRKLPKDIN
NLINLRHLDIYDCSSLTHMPKGLGGMTSLQTMSMFVLGKNKGGDLSALNGLKSLRGLLCIKGLQFCTTADLKNLELHWDI
KMDHEDALDDGDNDDEGVLEGLKPHSNIRKMIIKGYRGMKLCDWFSSNFLGGLVSIELSHCEKLEHLPQFDQFLYLKHLL
LGYLPNIEYIDSGNSVSSSTTFFPSLEKLRIESMPKLKGWWKGEISFPTTILHQLSELCIFYCPLLASIPQHPSLESLRI
CGVSVQLFQMVIRMATDLSEHSSSSSTLSKLSFLEIGTIDLEFLPVELFCNMTHLESLIIERCKSLQMSSPHPVDEDNDV
VWKKLSNLRTLRLESILKLEYFPKSLKYITSLETLKLSNCENLVSTEGIGELISLSHLEIDRCPNLPILSEDVGDLISLS
HLLIWNCPKLTSLSEGITRLTSLSSLCLEDCPNLVSLPQEFLHHHSSLPGGRFLRILNCPKLQIQDKKQKEEEEEDQEDW
NELIHVLTGCR

>Cucsa.128110
MADSILFNVAANVITKLGSSALRELGSLWGVNDELGKLQNILSAIKAVLLDAEEQQSVSHAVKDWISKLRDVFYDVDDLI
DEFSYETLRRQVLTKDRTITKQVCIFFSKSNQVSFGHKMSQKIKQVREKLDAIANDKTQLHLSVRMRETRDDELRKMRET
CSFIPKGEVIGRDDDKKAIIDFLLDTNTMEDNVEVVSIVGMGGLGKTAVAQSVYNDEKINEHFKLKLWVCISQEFDIKVI
VEKIIEFIAKKKPDSLQLDILQSMLQEKIDGKKYLLVMDDVWNESHETWVSLKRFLMGGAKGSRILITTRNLQVAQASDT
VQFHHLKELDNESSWALFRKMAFLNEEEEIENSNKVRIGKEIIAKLKGSPLTIRIVGRLLYFKNTEMDWLSFKDNDLGTI
LQQENQIQPILKISFNHLPSNLKHCFTYCALFPKDYEFQKDGLVKQWMAQGFIQSHSNKEIEDVGDDYFKELLGRSFFHN
VKVNKWGDVKECKMHDLIHDLACWIVENECVDASDKTKSIDKRTRHVSFPSNYSRKSWELEAKSLTEVKNLRTLHGPPFL
LSENHLRLRSLNLGYSKFQKIPKFISQLRHLRYLDISDHDMKFLPKFITKLYNLETLILRHCSDLRELPTDINNLINLKH
LDVHGCYRLTHMPKGLGGLTSLQTMNLFVLGKDKGCDLSELNELARLRGSLLIKGLELCTTTDLKNAKYMEEKFGIQKLK
LRWNRDLYDAETDYASENDDERVLDCLKPHSNVHKMQIRGYRGVKLCNWLSFDYLGGLVNIELQSCEKLQHLPQFDQFPF
LKHLLLENLPSIEYIDNNNSLSSSTFFPSLEKLTIMTMPNLKGWWKGETPPESARYSALFPTILHHLSRLDISNCPQLAS

IPQHPPLRSLALNDVSVQLFDMVIKMATTPAADSSSALSKLSILHIQNIDLEFLPEELFGSTTDLEIFTVELKYMTTLER
LDLYNCPNIVSLEGISHLTSLSSLRICNCSNLTSLPEGISHLTSLSYLTIYCVNLTSLPEGVSHLTSLSSFTIEECPCLT
SLPEGVSHLTSLSTLIIRRCVNLTSLPEGIGHLTSLSIFTIEECLNLTSLPEGLLHLSSLRGSLTVSKCPKLSKTWKKLN
K

>Cucsa.128130
MAIGDPQLPILHRAPVDKSHGIKKLSSHALECLGMVCGLNDDLNKLRSNVSSIQSVLRDAEQRQIKGNDHSLTDWLEKLG
DVFYDVEDVLDEISTEALRREVMTRGKNAKQVRIFFSNSNQLAFNYRMACQVKKINERLDVISQEKDKFQLNGIAYLGIQ
NVLSYPIGMERDTHSSLSGDQKIIGRDDEMNNLKKNLLAEDDKVKANVSFIAIVGMGGIGKTTLAKSLYNDKQVSDGFSS
RIWIWVSNQFDTKTILKKIIESATEKKPKVEEMEPLKTKLEEVIGGKKYLLVMDDVWNENENEWENLKNLLMLGARGSKV
LITKRDSKAVPGVETIPLKDLTEDFSWLLFKEVAFKESDLESINQNLIKMGKEISKRCGGIPLVIRHIGRLLYGKTSAED
WEFIKENELLNVTREKNNNDGHVISTLKLSYNHLSPNLKQCFSYSSLFPKGYKIRMNELIRQWIAQGFIESSNGGKSVEN
IGKEYLDELCWRFFYEISIEDVPFEEVGMHDLMCDLAREVAGQKLYIRGYPESGYVVSEQTRHISFEYEPRSWIDDVSKL
QQAKGLRTFLLFTKNPFFTRNPIEKVLLDRLFSHFPRLRVLQIPNVSKSIKKLRHLRYLELGEDAKSVPNSITKLQNLQT
LDLTKCYDLKELPRDINNFVNLRHLLCDSRLMNMLQGTMEKLTSLQTLSSFLFDCKRFDKVKEFSERSYFIEFDLKIKGL
EQLRFSPSDVKSVNLKNKKVPLLRLKWKFENGNEYEGDADDIVLEGLEPHPYVNLLQIEGYCGVGLPNWVSTSILLRGIR
IGNCDRLHLNQLSHLHALEILNLEGLKSVMSISEWIGTLTSLVSLEIEECPKLKSLPKEMQQLKSLVQLNIIKCPQLGER
CKEGGEDWPNISHIPDVLID

>Cucsa.128140
MAEAILYNVTADIIFKLGSSALQELGLLWGVNDELDKLKHSLSAIQAVLLDAEEQQSKSLAVKAWVSRLKDALYEIDDLV
DESSYETLRRQVLAKDQRKRKLVRILFSKFKSNWKIDHKIKDIRQRLQSINDDKNQFSFSEHVIEKRDDEELRKKRRETYS
YILEEEVIGRNDDKEVVIDLLLNSNITEDIAIVSIVGMGGLGKTALAQSIYTHHNMTNSGFELKLWVCVSEEFDLKVIIQ
KMIESATGTKPKPYLQIDSLQSELRKKIDGKKYLFVMDDVWNEKKEEWLRLKRLLMGGAKGSRILITTRSEQVAKTFDST
FIHFLQILDEYNSWLLFQKITCLEGHPSNPEKLDQSSSLIQIGREIVSKLKGVPLTIRTIGGLLKDNKSKRVWLSFKDNE
LHRILGQGQDNLKEVRLILELSYKYLPANLKQCFLYCALFPKDYEIKTHELILMWSAQGFIQPNGSKDNSLIDIGNDYFM
ELLSRSFFQEVTKNERGDIIACKMHDLMHDLACWIADNECNVINIGTRHFAWKDQYSHKDQLLRSLSKVTNLRTFFMLDS
ANDLKWEFTKILHDHLQLRALYFKNLKNAMIVLEFTGKLKHLRYLSIMDSFILNLPDSITELYNLETLILRNSSFKMLPD
NIGNLINLKHLDLSNNRNLKFLPDSISDLCKLEELILHGCLRLEEFPEDTKKLINLKHLNEKHADALWRDKEKGFNEGLC
SLIKAVEKDFHSVHKCKLFFCSVVFLM

>Cucsa.132370
MGDLLTFGVQETLKQAVTLVAKKIIASSEFKVVLEELKDDLLHAEWILHAIKTKHDHSLNDKITHWVNDLQLIVYEAEDM
LDLFAYDDVERKIRSNKVFPNSLCTIKPMLDCFSLVVFVHLDNTTRKIESEVEQVEETTSLLENYVVGREMEVESIVQDV
TEASQQQLNSILPVYGTGGSGKTTLAQLVFNDERIGKQFHHTVWVCVSQPFVINEILQSILKKVSKSNDNRSKDDKDTLI
RNLKEVMGGKRYFLVLDNVWNENKIFWEKLKECLMSIVEELGSSVLVTTRSRKIAEMMKETLDTYHLNKLTDDQCWSVFS
YFAKANAVPITSNLELVREELSVDRLPKASIKQCFAYCSNFPKGYWFDKKQVIKMWMAHGFTRPDEGNNETMEDTGERYF
NILLSYCLFQDADDDKWHIGRKFRMHDLIHDIACDVSSDKRLQLDHSSSSKWKGLTEEKKKIESKLRTVIDFGRNGKIKD
FVCLRVLTIAENVRELPNSISKLKHLRYLDISRCYSIKKLPESIVGHLEILLMGIDLPPKFEMPPYLSELVQLQTLFAFA
VGFETGRKISELRGLRNLKGLLKLHRLEHVESKEEAKAAKLVEKEKVEGLNLSWRGKWKNRLEPHKNLKDLKIQSFLGGC
FPKETFVENLVTITLHKCGNCEKLPMLGQLSKLEALIIISNFPKVKSIGNEFYGNYNDGQSKSSVVFPKLKEFYVIAMYS
LVEWEEVVNNVKAFPRLECLHIVKCTKLTSALKIVFSSLINFDHSISYLNLLPNSLKILLSCCLFQDVEDESEIGQKFLM
HDLIHDIACHVSNDEKLPSDHSLLSMRKHWTNDDKIVASKLRTNIVKNENDFEVLEGLEQHNNLKYLEIESFSGGQFPNQ
IFVENLVKITLIECGNCEKLPMLGQLTKYLEILVIFRLRKVESIGNEFYGNQRRSSSSVFPKLKEFYVDEMDSLVEWEEA
VSNYNVKAFPRLECLHIISCKKLLKIPDTKVQICGLLLLQLQEEAPWLICYSNVRSFFLKEDTGPS

>Cucsa.133510
MAGALIGGAALGVPFNELATLLKNFGERAWSFNSVLNETESKVNDIIPLVKEIDGLNESLDYPREETEKLKNLLEYAGKL
LRRCLRVGKADLIRKSSHTEKLRELNARIKSFSDVVLFQTSRDGKKTLSLVTEIKEVVRRLDSKSGLSNPVDLVVTVPVI
SEESVGLEKPVEKLKAKLFRDGVRLLVVTAPGGCGKSTLAEIFCHDKQVKNKFQRNILFLVVSSKPETKRILISIIQRLG
GPIESGSVSDDEAFRLLEVRVGELSPNPVLIVLDDVWDGSESNKLLEKFSRLPNCKVLVTSRFKFPAFGESYDLEPLDHK
DAMELFRRWASRGNRVLQFPDERIVEKIVRGCKRFPLALKVIAGSLSGRATSVWEVTGRKLSRGDSILGSEKELQKCLKD
TLDAIPDDKIVLKECFMDLGSFPEDQRIRAATFIDICAVLYEQDECETMSNLDELFTRTLVNTVSLRNKAHEDDYYSESY
ITQHDVLRELAVLLTNEQPVDQRTRLLVDINKNEFPKWWSVRQMQPVKARLLSITTDEKFSSCWPDMEAPEVEVLILNPG
SETYKLPDFAKKMNRLKALIVRNYRSFPTELTSDYQLINCLSRLERISLERISISSFIDQNLKPLWHLKKLSFFMCKIDK
AFTDCSTQISYMLPNLLEISIDFCNDLVAFPVGLCEVVTLEKLSITNCHALSSLPEEIGQLINLKILRLRSCIHLEKLPE
SISRLRELVYLDISHCVGLTKLPDKIGNLQKLEKLNMWSCPNMRKLPKSVGNLKNLKEVVCESEMKIWVNFVAPRLGNVV
KEHKEEINLDFLN

>Cucsa.155730
MAISTNHSTLVLGIYGMSGIGKTTLSKALFNHFFHFFNSRSFLPNINSLSTSSPDGLLRLQQTLLSDLLIATNLRSRSST
TTDSTVVRMQERLQNKKVLVVLDDLDRIEQANALAIRDRRWFGDGSRIIITTRNKQILDTLKVDEVYNMESNLLNDEESL
ELFSYHAFREQNPPEELLECSKSIVSYCGSLPLALEILGGSFFGGRPMEEWRSAMERLKRIPAWDLQEKLRIGFEGLRDE

MEREIFLDVCCYFVGMKEELVVKIMDGCGMYGESGLRGLKWRCLVGVEFWSGRLKMHDLVRDMGREIVRQTCVKEPARRS
RVWLYHEALKILLHQNGSENIEGLAIDMGKGNNKEKFRLEAFGKMRNLRLLKLNYVHLIGSNFEHIISKELRWICWHGFP
LKSIPSSFYQGNLVAIDMRYSSLIHPWTWRDSQILENLKVLNLSHSEKLKKSPNFTKLPNLEQLKLKNCTALSSLHPSIG
QLCKLHLINLQNCTNLSSLPTSIYNLHSLQTFIISGCSKIHCLHDDLGHLESLTTLLADRTAISHIPFSIVKLKKLTDLS
LCGCNCRSGSGSSASLPWRLVSWALPRPNQTCTALTLPSSLQGLSSLTELSLQNCNLESLPIDIGSLSELKKLNLGGNKN
LRVLGTELCGLLKLNELNVENCGRLEFIQEFPKNMRSFCATNCKSLVRTPDVSMFERAPNMILTNCCALLEVCGLDKLEC
STNIRMAGCSNLSTDFRMSLLEVFSSSYLLLNYKFGFAVFHLYIVSI

>Cucsa.163670
MIPHEILSLFITSVYEYLTNIATKLGSLALQDLGLLWTGIHEEIDKLRDTLSAIQAVLHDAEQKQYKSSAVKEWVSRLKD
AFYDMDDLMDEFSYESFQRQVMTKHRTNNCTKQVCIFFSKSNQIRFRLKMVHKIKKIREKLDTIDKDKTQFNLFDNTREI
RNDEMTKRSETCSFILEGEVIGRDDDKKCIVHFLLDTNIIAKENIVVVAIIGMGGLGKTALAQSIYGDMKENKHFELTMW
VCISEEFDVKVIVEKIIESLTKKRPKPNLTLDTLQSMLREKIDGKKYLLVMDDVWNDERTKWINLKKFLMGGAKGSRILI
TTRTHQVAHIFDTDLFHDLSELDKDNSWELFRKMAFSNESEMLENSKLVGIGKEIVTKLKGSPLAIRVIGSYLYSKKSEK
DWLSFKENELDTIMQQENEIQSILKISFNHLSSSLKQCITYCALFPKDFEIDKDDLIKQWMGEGFIQPHNKKAMEDVGDE
YFKELLGRSFFQDISKNQLGEIMKFKMHDFMHDLACFVGENDYVFATDDTKFIDKRTRHLSISPFISKTRWEVIKESLIA
AKNLRTLNYACHNYDGDEIEIDFSNHLRLRTLNLIFSTHVPKCIGKMKHLRYINFTRCYFDFLPKVVTKLYHLETLIFRE
CFKLRELPSDITNLINLRHLGINSLIEGLSYMPKGMGSMTTLQTMNLFILGENEGGELSELNGLINLRGSLSIQQLQFCK
PIGIENAKHLEEKSGIQKLKLYWYLLERKYEIDDEDEKVLECLKPHPNLQKIVINGYGGVKLCNWFSFDYIVNLVIIDLF
NCNKLQQLPRFDQFPFLKHLKLQYLPNVEFIDNNDSVSSSLTTFFPSLEKLRIFRLPKLKEWWKRKLIDQTIPQHRRLES
LNISGVSLQVFELVMEMATTNIIVGSQDSSSSTTSISLSFLSIEDIDFEFLQFHDLFSNMTHLKSLWIINCKNIKMSSSL
DAVTWKGLGSLRELMLSSIPDLEYLPKSLQCVTTLQSLQIYNCPNLVSIESIRHLTTSLSVLEIHGCPNITFYPHEMSQL
ASLAITFQNRGWSDNYDPGEGRKEDDDQKQFGRDEQHEGTTH

>Cucsa.178360
MELCAGAIVNPIAEKIANCTVDPVFRQLDYLLHFKTNVNDLKDQGKKLVETRDFVQHSVDSAKTNGYEIEVMVTEWLGIA
DQFSEDVDRFFNEADGRSLRWWNMLSRHRFSRRATKLAVAVDKAIQGGSFERVGFRVTPQEIMTLRNNKKFEAFESRVLI
LKEIIEAVGDANARVIVVHGMAGVGKTTLVEEIARLAKEGKLFDAIAMVTVKHIPNIKKIQGEIADQLGLKFEEEKERIR
ADRLRRRLEMEKKVLVVLDDVWSRLDLEAVGISSHHKGCKILVTSRKDDLFFNDFGTQKNIYINILSKKEARDFFNKVAC
DSVESSDDTDPEMEAVATELADECGGLPLSLATVGQALKGKGLPSWNDALQGMKFPGEPSNYGVNKVAYLSLKVSYRSLN
REEARSLFLLCSLFPEDYQINIKYLLMYAMGLGLLNAMSSLAMAKWRILSLVDELKTSHLLLDGVDNDFVKMHDIVRDTA
ILIASKMKSKYLVRHGAGESLWPPMDEFKDYTAISLGCSDHSELPEFICPQLRFLLLVGKRTSLRLPEKFFAGMQELRVL
DLTGLCIQRLPPSIDQLVNLQTLCLDDCVLPDMSVVGELKKLEILSLRASDIIALPRVIGELTNLKMLNLSDCSKLKVIP
ANLLSRLIGLSELYMDNSFKHWNVGQMEGYVNARISELDNLPRLTTLHVHIPNPTILPHAFVFRKLSGYRILIGDRWDWS
GNYETSRTLKLKLDSSIQREDAIQALLENIEDLYLDELESVKNILFSLDYKGFPKLKGLRVKNNGEIVTVVNSDNMHHPH
SAFPLLESLFLKNLAELGSICRGKLPQMSFRNLKRVKVESCDRLKFVFPSSMVRGLIHLQSLEISECGIIETIVSKNKET
EMQINGDKWDENMIEFPELRSLILQHLPALMGFYCHDCITVPSTKVDSRQTVFTIEPSFHPLLSQQVSFPKLETLKLHAL
NSGKIWQDQLPSSFYGFKNLTSLSVEGCASIKYLMTITVARSLVNLERLELNDCKLMKAIIISEDQDLDNNYPSKSILQN
KDVFANLESLLISRMDALETLWVNEAASGSFTKLKKVDIRNCKKLETIFPNYMLNRVTNLERLNVTDCSSLVEIFQVKVP
VNNGNQVRDIGANHLKELKLLRLPKLKHIWSSDPHNFLRYPSLQLVHTIHCQSLLNLFPVSIAKDLIQLEVLKIQFCGVE
EIVAKRGDDGDGDDAASFLLSGLTSLTLWNLFEFKRFYPGKYTLDCPSLTALDVRHCKSFKLMEGTLENSSSISSAVEKV
LHSL

>Cucsa.178450
MNQASGSSSSSRFRWHFDVFLSFRGEDTRSNFTSHLNMALRQRGINVFIDNKLSRGEEISASLLEAIEGSKISIVIISEN
YASSRWCLNELVKIIMCNKLRGQVVLPIFYKVDPSEVRKQSGKFGEEFAKLEVRFSSEKMQAWREAMISVSHMSGWPVPK
KDDEANLIQRIVQEVWKKLNRGTREMRVPKYPVGIDRQVNNILSQVMSDEIITMVGLYGIGGIGKTTLAKALYNKIADDF
EGCCFLINVREASNQYRGLVELQKELLREILMDDSIKVSNLDIGISIIRDRLCSRKILLILDDVDTSEQLEALAGGHDWF
GPGSVVIATTRNKHLLAINEFDILQSVQGLNDDEAFELFSWHAFKMSCPSSHYLYLISKRAVSYCKGLPLALEVVGSFLY
SIEPSKLKLILDEYENQYLDKGIQDPLRISYDGLEDEVKEIFLYISCCFVGEDINKVKMKLEACGCLCLEKGTTKLMNLS
LLTIDKSNRVEMHNLIQHMGRTIHLSKTSTSHKRKRLLIKDDAMDVLNGNKEAKGVKAIKLSFPKATELDIDSRAFEKVK
NVVVLEVGNVTSSKGTDLEYLPSSLRWMNWPHFPFPSLPTTYTMENLMELKLPYSSIKHFGRGFMSGERLKEIDLSGSEF
LVEIADLSTATNLEKLNLLGCVNLVKVHDSVGSLTKLVTFSLSSNVKGFEQFPPHLKLKSLKLLSMENCRIDEWCPQFSE
EMKSSLEELLIQYSTVINQLSPTIGYLTSLKRLFIIECMKLKTLPSTIYRLRNLTFLSITNLDFLETMVHVAPALKLLDL
SGNNFCRLPSCIINFKSLKSLVTMECKLLEEIPKVPKGVVRMNATGCISLTRFPDNIPDFICCDDNVVRIIVLSHDLMIS
RVFRSYKN

>Cucsa.178620
MAEAILFNLTADIIFKLGSSALRQFGSLRGGVKDDFDKLWHSLSAIQAVLHDAEEKQFKDHAVEVWVSRLKDVLYEIDDL
IDEFSYQILRRQVLRSNRKQVRTLFSKFITNWKIGHKIKEISQRLQNINEDKIQFSFCKHVIERRDDDDEGLRKRRETHS
FILEDEVIGRNDDKEAVIDLLLNSNTKEDIAIVSIVGMPGFGKTALAQSIYNHKRIMTQFQLKIWVCVSDEFDLKITIQK
IIESATGKKPKSFLQMDPLQCELRKQIDGKKYLIVMDDVWNEKKEKWLHLKRLLMGGAKGSRILITTRSEQVAKTFDSTF
VHLLQILDASNSWLLFQKMIGLEEHSDNQEVELDQKNSNLIQIGMEIVSTLRGVPLLIRTIGGLLKDNKSERFWLSFKDK

ELYQVLGRGQDALKEIQLFLELSYKYLPSSNLKQCFLYCALFPKDYRIKKDELILLWRAQGFIQQNGNNDDNSSLVDIGE
DYFMELLSRSFFQEVEKNDFGDIITCKMHDLMHDLACSITNNECVRGLKGNVIDKRTHHLSFEKVSHEDQLMGSLSKATH
LRTLFSQDVHSRCNLEETFHNIFQLRTLHLNSYGPPKCAKTLEFIKFLPDSITKLYKLEALILDGCSNLKELPKYTKRLI
NLKRLVLYGCSALTHMPKGLSEMTNLQTLTTFVLGKNIGGELKELEGLTKLRGGLSIKHLESCTSIVDQQMKSKNSKFLQ
LKSGLQNLELQWKKLKIGDDQLEDVMYESVLDCLQPHSNLKEIRIDGYGGVNLCNWVSSNKSLGCLVTTYLYRCKRLRHL
FRLDQFPNLKYLTLQNLPNIEYMIVDNDDSVSSSTIFPYLKKFTISKMPKLVSWCKDSTSTKSPTYWHAPKLKLLQISDS
EDELNVVPLKIYENLTFLFLHNLSRVEYLPECWQHYMTSLQLLCLSKCNNLKSLPGWIRNLTSLTNLNISYCEKLAFLPE
GIQHVHNLQSIAVVDCPILKEWCKKNRREDWPKIKYYISEHIWENICSLTGSWSSRNKIFSDHFRSVIALNCSACFYLIY
ICRCYYELISSPFFIILYHVLFNE

>Cucsa.189390
MDIISPVVGPIVEYTLKPIGRQLSYLFFIRQHIQNLESQVELLKNTKESVVNKVNEAIRNAEKIESGVQSWLTKVDSIIE
RSETLLKNLSEQGGLCLNLVQRHQLSRKAVKLAEEVVVIKIEGNFDKVSSPVALSEVESSKAKNSDFVDFESRKPTIDKI
IAALMDDNVHTIGVYGMGGVGKTMLVQEISKLAMEQKLFDEVITSTVSQTPDLRRIQGQLGDKLGLRFEQETEEGRALKL
LNRLKMERQKILIVLDDVWKQIDLEKIGIPSIEDHSGCKILFTSRDNDVLFNDWRTYKNFEIKFLQEDETWNLFRKMAGE
IVETSDFKSIAVEIVRECAHLPIAITTIARALRNKPASIWKDALIQLRNPVFVNIREINKKVYSSLKLSYDYLDSEEAKS
LFLLCSMFPEDYIIDCQVLHVYAMGMGLLHGVESVAQARNRITKLVDDLISSSLLLKESNVDLVMYVKMHDIVRDVAIII
ASKDDRIFTLSYSKGLLDESWDEKKLVGKHTAVCLNVKGLHNLPQKLMLPKVQLLVFCGTLLGEHELPGTFFEEMKGMRV
LEIRSMKMPLLSPSLYSLTNLQSLHLFDCELENIDVICELNKLENLSLKGSHIIQIPATISQLTQLKVLDLSECYALKVI
PPNILVNLTKLEELYLLNFDGWESEELNQGRRNASISELSYLSQLCALALHIPSEKVMPKELFSRVFPFELNENESSYLK
YLYINYNSNFQHFIHGQNKTNLQKVLSNMERLELSYLENLESFFHGDIKDISFNNLKVIKLLSCNKLGSLFLDSNMNGML
LHLERINITDCEKVKTVILMESGNPSDPVEFTNLKRLRLNGLPQLQSFYSKIEQLSPDQEAEKDERSRNFNDGLLFNEQ

>Cucsa.237070
METVIAILGTVCEYAVAPIGRQVGYVSSYKKNINDLKDQLQNLVDTKTRLQHMVNEARSSAYNIQSDVSSWLNQVDKIIE
QSNDILYKNENESNSKYCSNKLNFIHQYQMSKKAKKMVKVISQIIEKRKLMFHQVGYPTPLSRIHGSSTSSSHGYDQILE
SRTSIAKQIRDALVDCNVNKVGVYGMGGVEKTTLLKQVTPLVMEEKLFDHVIIVNVGQTLGVEGIQAQIGDKLRLELNKK
VESKEGRASLLQNKLEMESNVLLVLDDLWKGLDLEEVGIPCRSESCEKGCKILITSRDRDVLTNEMDTQVYFEVKPLSEK
ESWEFFKNMIGEFDNKCIELIGKEMVKKCGGLPIALATIVKTLKGKEVPIWKDALKQLKNPIAVDVKGVTDVFPDDYEIS
VEDLQIYAMSLRLLNQVNTWDEARNRVIKLVDDLKASSLLLESNSRDNHVKMHDIVRDVAIYIASKEANMSTLSYGFGLS
EWQEKDRHGFYRAIFGNCHNFYNFPQNLEFPKLELLILDGHDWRGEKLQICYSFFEGMKELKVLNLSRMCFQLLRRPSIH
SLENLQTLCMSHCTFNDIDAISHLKKLQILRIDKCPITLLPKSMSQLTQLKVLQVSNCPLKVIPPNTLSSLLKLQALDIW
TSFNGWGEEVSHNNKLINNARLSELKCLPHLTNLKIHILDIKILSDLIFLKNLKLERFVIHVGELKMSQRLQGCEQYATT
LMLKIITSSSQIVSIDHHEFLSLEKMESLENIVHADVFTSPFRKLRSIKVISCKRLRYLFSFSIFKGLVDLQRVFIFDCN
MMDEILCMDSEDSTIAVEGNSIECPQLKDLTIIGAHNLKMLWHKNGLAPNFFSKLQRISINSCNTLRYRG

>Cucsa.237390
MVPSSPSSSAASSSSPSSSPSIGKWKFDVFLSFRGEDTRGGFTDHLYKALTRKGISTFRDENEIEEGEHIPSNLLASIDA
SRFAIVVVSEDYASSRWCLEELARMFECKKEVLPIFYKVDPSHVKNQSGTFEEAFVKHEKRFGRGDGKVQSWRTFLTELA
NTKAWLSQSWSHESNIIEEITTKIWKRLKPNLTVIKEDQLVGINSKINKLSSLLIPNSDDDDADDDVIFVGIHGMGGIGK
TTIARVCYERIRDEFEAHCFLSNVRENYIRTLGNLSCLQTKLLSSMFSLKNNHIMDVEEGTAMINKAIFRKKTLLVLDDV
DSSDQIKGLIPDNNSFGNGSRVIITTRNADFLSNEFGVKRIFEMDELKYEEALQLLSLSAFMKTCPKEGYLEHSKKIVKV
VGGHPLALKLLGSSLRNKNLSVWNEVIEEVGGGGNIHEKIFKCLKVSYDGLDEREREIFLDVACFFNGKRREVVEEILNG
CGFYAKTRIELLIQKSLLTLSYDNKLHMHNLLQEMGRKIVRDKHVRDRLMCHKDIKSVVTEALIQSIFFKSSSKNMVEFP
ILFSRMHQLRLLNFRNVRLKNKLEYSIPSELRYLKWKGYPLEFLPIDSSEECKLIELHMCHSNLKQFWQQEKNLVELKYI
KLNSSQKLSKTPNFANIPNLKRLELEDCTSLVNIHPSIFTAEKLIFLSLKDCINLTNLPSHINIKVLEVLILSGCSKVKK
VPEFSGNTNRLLQLHLDGTSISNLPSSIASLSHLTILSLANCKMLIDISNAIEMTSLQSLDVSGCSKLGSRKGKGDNVEL
GEVNVRETTRRRRNDDCNNIFKEIFLWLCNTPATGIFGIPSLAGLYSLTKLNLKDCNLEVIPQGIECMVSLVELDLSGNN
FSHLPTSISRLHNLKRLRINQCKKLVHFPKLPPRILFLTSKDCISLKDFIDISKVDNLYIMKEVNLLNCYQMANNKDFHR
LIISSMQKMFFRKGTFNIMIPGSEIPDWFTTRKMGSSG

>Cucsa.237450
YDVFLSHRAKDTGRSFTSYLHEALTSQGIVVFIDEEDEDNGGKPSMEKTKAVDESRSSIVVFSENYGNLVCMKEIRKIRM
CQKLGDQLVLPVFYKIDPGDVRKQEGSFEKYFNEHEVNPNISIEEVKKWRKSMNKVGNLSGWHVQDSQLTFSDDSRQLWV
SYEPREVYPYRLNKWRNLRVSFLPSCSQTKVILCGARLLYKEDLDEFVDTIIDSVLGCSINLHEFYDGVFLNGMLSLIRS
QKYDPNIEEEEEEEEDEDEALMETKGGNYASTSSRVYRFSLFLSLSCILIFEQTTKITPKMYDSRSCNNYKNST

>Cucsa.237500
MSTFDTFISFRGEDTRNTFTGHLYKALVDFGISTFMDDKKLLIGDSLSEDLIGAIEKSGSFIVVLSENYASSKWCLRELV
KIIGCMVEQKRRVLPVFYHVSPHDVRHQSGCFKKSFCEYEEILQELNDREGDKYTKEVQEWRSALTKVGELTGVVVTKDS
LEAASIDKITEQLSSTLHQQKLVNLDELTELVDIERQLCKMDKLNDLEPNVVRFIGIIGMGGIGKTTIAEVFYEKVAYKF
GKNCFLIVLDGVKEKSQLEQLVGNPNWFGQGSKIIITTRNRDVLRQPNYKDKMVEYKVEFLDNKSAMTLFCKQAFGSCDQ
FPSKNFEDFSKEIVERVKGHPQVLRQIGSSLYDKGIEIWKEQLKSLEEDYNNRIFKTLKISFDDLGKTSQEVFLDFACFF

NEKKKESVIEILKSLDYRPHSEIQLLEDRCLIEVRRDNTIFMPKCIQAMGQQIEREADKRSRIWLPKDAHDVFDEPHRVK
DIKGVVLKLEEKQEEVKLEGKVFEDMRSLKILEIGNVEVSGDFTHLSKQLRLLNWQSYPSQCLPLRFESRYLFQLLLPLS
QTRQLWNGQKGFEKLKVINVSRSKNLRETPNFTKVPNLESLDLSYCPRLWKIDSSISRLNRLTLLDVSYCINLESLPFSR
SCKSLARINYAGSGLEERGIYTYLSLW

>Cucsa.237520
MCRPPYWCWDDSWMFFDVGYTKFKIQSIANSIGDHLLRLKLQAKEENLFEMPLRLRTMKMLLGLGSNDVRFIGIVGMSGI
GKTTLAEMTYLRIFKPFVSALRKPYFLHFVGRSIVSLQQQLLDQLAFLKPIDIQVLDENHGVELIMQHLSSLKNVLIVFD
GITERSQLEMLAGSPDWFGAGSRIIITTTNKNIFHHPNFKDKVQEYNVELLSHEAAFSLFCKLAFGDHPHTQNMDDLCNE
MIEKVGRLPLALEKIAFSLYGQNIDVWEHTLKNFHQVVYDNIFSDVLKSSYEGLEAESQQIFLDLACFLNGEKVDRVIQI
LQGFGYTSPQTNLQLLVDRCLIDILDGHIQMHILILCMGQEIVHRELGNCQQTRIWLRDDARRLFHENNELKYIRGIVMD
LEEEEELVLKAKAFADMSELRILRINNVQLSEDIECLSNKLTLLNWPGYPSKYLPSTFQPPSLLELHLPGSNVERLWNGT
QNFKNLKEIDASDSKFLVETPNFSEAPKLRRLILRNCGRLNKVHSSINSLHRLILLDMEGCVSFRSFSFPVTCKSLKTLV
LSNCGLEFFPEFGCVMGYLTELHIDGTSINKLSPSITNLLGLVLLNLRNCIRLSSLPTEICRLSSLKTLILNGCKNLDKI
PPCLRYVKHLEELDIGGTSISTIPFLENLRILNCERLKSNIWHSLAGLAAQYLRSLNDLNLSDCNLVDEDIPNDLELFSS
LEILDLSSNHFERLSESIKQLINLKVLYLNDCNKLKQVPKLPKSIKYVGGEKSLGX

>Cucsa.237540
MQSSSSSSLDRPKMNYDVFISFRGRDVRHTFAGYLYDALNRLGIKAFLDNKRFLIGDDLHDLFKIIDESRSAIVVLSEDY
ASAKWCLRELTKIMDSMGTSMERVLPVFYHIDPSIVKDQSGTFKTSFDEHEANVLKEIDNQEKEKRLKELQNWKSALKKI
GNHTGVVITKNSSEVDIVNKIASQIFDAWRPKLEALNKNLVGMTSRLLHMNMHLGLGLDDVRFVAIVGMGGIGKTTIAQV
VFDCILSKFEDCCFLTLPGGDSKQSLVSLQREMLSQIFHKEDFRIWHENHGVEMIKNRLSGRKVLIVLDGIEERRQLEML
AGSIEWFGPGSRIIITTRNKGLLCHPNYDEMKVYNVEELDHDSALQLFLKHAFGSNHQNNDSFMDLSNEIVEKAKRLPLA
LRVIGSSLYGKDITVWRETLKRLIKVDERNFFDVLKISYDGLGVESQQVFLDITCFFNGKNEDRVIEILESFGYSPNSEV
QLLMQRCLIEVSHKKILVHDLILEMGREIVRKESLTQAEKQSRIWLHEDLYCRFAEKHDLMHIQGIVLSLAKEMEESIEL
DAESFSEMTKLRILEISNVELDEDIEYLSPLLRIINWLGYPSKSLPPTFQSRYLFELLLPHSHLLRIWDGKKRFPKLKLI
DVSNSEHLRVTPDFSGVPNLERLVLCNCVRLCEIHPSINSLNKLILLDLEGCGDLKHFPANIRCKNLQTLKLSGTGLEIF
PEIGHMEHLTHLHLDGSNITHFHPSIGYLTGLVFLDLSSCLGLSSLPCEIGNLKSLKTLLLKYCKKLDKIPPSLANAESL
ETLSISETSITHVPPSIIHCLKNLKTLDCEGLSHGIWKSLLPQFNINQTITTGLGCLKALNLMGCKLMDEDIPEDLHCFS
SLETLDLSYNNFTTLPDSLSHLKKLKTLNLNCCTELKDLPKLPESLQYERFRSKFDLLLHGDKIPKFFSNQSKGNMTEIK
LPQYLEKFRESIGVAVCALVVVDKKRRKLNEIIPERERYTKVVDLICKFKVDSYQIMPEHCHFTSQQKLLSEYASQFLWL
SYIPLHGFNINWHYCTQFEIALETSCDELFGVKNCGLHLIHKHERMMIDKMVMESTVPSSTSHKGKEPQIH

>Cucsa.237560
MASSTPKELSSFSSSPRFIFDVFLSFRGVDTRKNVTNRLYEALRRQGIIVFRDDDELERGKTIANTLTNSINQSRCTIVI
LSKRYADSKWCLRELVEIVKCKNTFKQLVLVVFYKIKPSDVNSPTGIFEKFFVDFENDVKENFEEVQDWRKAMEVVGGLP
PWPVNEQTETEKVQKIVKHACDLLRPDLLSHDENLVGMNLRLKKMNMLMGIGLDDKRFIGIWGMGGIGKTTIAKAVFKSV
AREFHGSCILENVKKTLKNVGGLVSLQEKLLSDTLMRGKVQIKDGDGVEMIKKNLGNQKVFVVLDGVDHFSQVKDLAGGE
EWFGCGSRIIITTRDEGLLLSLGVDIRYNVESFDDEEALQLFCHEAFGVKFPKKGYLDLCMPFIEYAEGLPLAIKALGHS
LHNRLFKSWEGAIRKLNNSLNRQVYENLKISYDALGKEERRIFLYIACFLKGQNKDQVIDTFVSFEIDAADGLLTRKNAA
DVLCIKETAADALKKLQEKSLITMLYDKIEMHNLHQKLGQEIFHEESSRKGSRLWHREDMNHALRHKQGVEAIETIVLDS
KEHGESHLNAKFFSAMTGLKVLRVHNVFLSGVLEYLSNKLRLLSWHGYPFRNLPSDFKPSELLELNLQNSCIENIWRETE
KLDKLKVINLSNSKFLLKTPDLSTVPNLERLVLNGCTRLQELHQSVGTLKHLIFLDLKDCKSLKSICSNISLESLKILIL
SGCSRLENFPEIVGNMKLVKELHLDGTAIRKLHVSIGKLTSLVLLDLRYCKNLRTLPNAIGCLTSIEHLALGGCSKLDKI
PDSLGNISCLKKLDVSGTSISHIPFTLRLLKNLEVLNCEGLSRKLCYSLFLLWSTPRNNNSHSFGLWLITCLTNFSSVKV
LNFSDCKLVDGDIPDDLSCLSSLHFLDLSRNLFTNLPHSLSQLINLRCLVLDNCSRLRSLPKFPVSLLYVLARDCVSLKE
HYNYNKEDRGPMSQAEVRVLSYPSSAKDQNSKISQLMISSMCTACENGG

>Cucsa.237410
MERRASIKSLSPPPYSISLPLPPLRNYDVFLSHRVKDTGSSFAADLHEALTNQGIVVFRDGIDDEDAEQPYVEEKMKAVE
ESRSSIVVFSENYGSFVCMKEVGKIVTCKELMDQLVLPIFYKIDPGNVRKQEGNFKKYFNDHEANPKIDIEEVENWRYSM
NQVGHLSGWHVQDSQSEEGSIINEVVKHIFNKLRPDLFRYDDKLVGISPRLHQINMLLGIGLDDVRFVGIWGMGGIGKTT
IARIIYKSVSHLFDGCYFLDNVKEALKKEDIASLQQKLLTGTLMKRNIDIPNADGATLIKRRISNIKALIIILDDVNHLSQ
LQKLAGGLDWFGSGSRVIVTTRDEHLLISHGIERRYNVEVLKIEEGLQLFSQKAFGEEHTKEEYFDVCSQVVDYAGGLPL
AIEVLGSSLRNKPMEDWINAVEKLWEVRDKEIIEKLKISYYMLEKSEQKIFLDIACFFKRKSKKQAIEILESFGFPAVLG
LEILEEKCLITTPHDKLHMHDLIQEMGQEIVRQNFLNEPEKRTRLWLREDVNLALSRDQGTEAIEGIMMDLDEEGESHLN
AKAFSEMTNLRVLKLNNVHLSKEIEYLSDQLRFLNWHGYPLKTLPSNFNPTNLLELELPNSSIHHLWTASK

>Cucsa.237440
MTSLSFPPPPPPPPYSISLPLPPLRRYDVFLSHRAKDTGCSFTSNLHEALTSQGIVVFIDKEDGGKPLTEKMKAVDESRSS
IVVFTKNYGSLVCMKEIRKIRMCQKLRDQLVLPVFYKIDPGDVRKQEGSFEKYFNEHEVNPNISIEEVKKWRKSMNKVGN
LSGWSEEGTINEVVNHIFNKLRPDLFRYDDKLVGISRRLHEINKLMGIGLDDVRLIGIWGMGGIGKTTIARIIYKSVSHL
FDGCYFLDNVKETLKKEGIASLQQKLLTGALMKRNIDIPNAEGATLIKRRMSNIKALIIILDDVDHLSQLQQLAGGSDWFG

SGSRVIVTTREEHLLISHGIKRRYNVEVLKIEEGIQLFSQKAFGEDHPKKGYFDLCSQVVDYAGGLPLAIEVLGSSLRNK
PMEDWIDAVKKLWEVRDKEIIEKLKISYYMLEKDDREIFLDIACFFKRKSKRQAIEILESFGFPAVFGLDILKEKSLITT
PHEKIQMHDLIQEMGQKIVNEKFPDEPEKRSRLWLREDITRALSHDQGTEAIKGIMMDLDEEGESHLNAKAFFSMTNLRI
LKLNNVHLSEEIEYLSDQLRFLNWHGYPLKTLPSNFNPTNLLELELPNSSIHHLWTASKVHQNNSSN

>Cucsa.237470
MLLINYASNACTYLLSIGSLITTKKRDVLHQLNYRDKVLEYKVELLSRESAYSLFSKNAFGGGPSDKDELCNEIVEKVGR
LPLALKTIGSYLHNKELDVWNETLKRLDGVEQDFCDTVLQKX

>Cucsa.237480
MGKQTNHKLVLAHKTSLVGMENQVEKACNLLDLERSKNILFVGIFGSSGIGKTTIAEVVYNTIVDEFQSGYFLYLSSKQN
SSVPLQHQMLSHLQSKETKIWDEDHGAQLIKHHMIHERNQIQRLVGSPNWFAPGSRVIITARNRDVLHELNYRDQVQEYK
VELLSRERAYSLFCENAFGDGGPSDKKDLCSEIVEKVERLPLALRTIGSYLHNKDLDVWNETWKRLDE

>Cucsa.237530
MGKQTDNKLVLSHKTSLVGMENQVKKVCNLLDLERSKDILFVGIFGSSGIGKTTIAEVVYNTIIDEFQSGCFLYLSSKQN
SLVPLQHQILSHLLSKETKIWDEDHGAQLIKHHMSNRKVVIVLDGVDERNQIEKLVGSPNWFAPGSRVIITATNRDVLHQ
LNYRDQVQEYKVELLSRESAYSLFCKNAFGDGPSDKNDLCSEIVEKVGRLPLALRTIGSYLHNKDLDVWNETLKRLDEEE
QNYFDTILKRN

>Cucsa.239860
MAEFIINVASVIVTPIGKYVIKPIGNQLGYIVFYNRNKNEIKEQLESLETTKKDLDLRVE
DAKSKAYTIFTKVSEWLVAADDEIKKSDELFNSNPPCLNFLQRHQLSRKARKRATDIRRL
KDGGNNFLEVGCPAPLPDTMNTIVPEAYQTLGSKTSMAKQIKDALAKPEVRKVGIYGMGG
VGKTYLLKEVKKLVLEEKLFDLVIDVTVGQSNDVMNMQQQIGDFLNKELPKSKEGRTSFL
RNALVEMKGNILITFDDLWNEFDIINDVGIPLSKEGCKTLVTSRFQNVLANKMNIKECFK
VTCLDDEESWKFFKKIIGDEFDAKMENIAKEVAKQCGGLPLALDIIAKTLKRSRHINYYW
EGVLSKLKNSIPVNIDVGEKVYASLKLSYEHLDGEEVKSLFLLCSVFPDDHGISVNDLQM
YVMGMGLLKMVNTWKEARAEAHYLVEDLTSSSLLQRLKNRDVKMHDIVRDVAIYIGPDFN
MSTLYYGYSTSSKGLDEDKCRSYRAIFVDCKKFCNLLPNLKLPKLELLILSFPFWGKDRN
IDIMDAYFEGMENLKVLDIEGTSFLQPFWTPLKNLRTLCMSYCWCEDIDTIGHLKQLEIL
RISNCRGITELPTSMSELKQLKVLVVSHCFKLVVIHTNIISSMTKLEELDIQDCFKEWGE
EVRYKNTWIPNAQLSELNCLSHLSILRVRVLKLTILSEALSSQMLKNLREFFIYVGTHEP
KFHPFKSWSSFDKYEKNMSFNMKSQIVSVNGTKLSILLEGTKRLMILNDSKGFANDIFKA
IGNGYPLLKCLEIHDNSETPHLRGNDFTSLKRLVLDRMVMLESIIPRHSPINPFNKLKFI
KIGRCEQLRNFFPLSVFKGLSNLRQIEIYECNMMEEIVSIEIEDHITIYTSPLTSLRIER
VNKLTSFCSTKSSIQQTIVPLFDERRVSFPELKYLSIGRANNLEMLWHKNGSSFSKLQTI
EISDCKELRCVFPSNIATSLVFLDTLKIYGCELLEMIFEIEKQKTSGDTKVVPLRYLSLG
FLKNLKYVWDKDVDDVVAFPNLKKVKVGRCPKLKIIFPASFTKYMKEIEELEMVEPFNYE
IFPVDEASKLKEVALFQSLETLRMSCKQAVKERFWVMSKFFKLKSLELFGCEDGKMISLP
MEMNEVLYSIEELTIRGCLQLVDVIGNDYYIQRCANLKKLKLYNLPKLMYVLKNMNQMTA
TTFSKLVYLQVGGCNGMINLFSPSVAKNLANLNSIEIYDCGEMRTVVAAKAEEEEENVEI
VFSKLTGMEFHNLAGLECFYPGKCTLEFPLLDTLRISKCDDMKIFSYGITNTPTLKNIEI
GEHNSLPVLPTQGINDIIHAFFTIEFTSICGVHTILSEKE

>Cucsa.248810
MADFLWTFAVEEMLKNVLKVAGEQTGLAWGFQEHLSNLQKWLLNAQAFLRDINTRKLHLHSVSIWVDHLQFLVYQAEDLL
DEIVYEHLRQKVQTTEMKVCDFFSLSTDNVLIFRLDMAKKMMTLVQLLEKHYNEAAPLGLVGIETVRPEIDVISQYRETI
SELEDHKIAGRDVEVESIVKQVIDASNNQRTSILPIVGMGGLGKTTLAKLVFNHELVRQRFDKTVWVCVSEPFIVNKILL
DILKNVKGAYISDGRDSKEVLLRELQKEMLGQSYFLVLDDVWNETFFLWDDLKYCLLKITGNSNNSILVTTRSAEVAKIM
GTCPSHLLSKLSDDQCWSLFKESANAYGLSMTSNLGIIQKELVKKIGGVPLAARVLGRAVKFEGDVERWEEMLKNVLTTP
LQEENFVLSILKLSVDRLPSSSVKQCFAYCSIFPKDFVFEKQELIQMWMAQGFLQPQQGRYNNTAMENVGDIYFNILLSR
CLFEFEDANKTRIRDMIGDYETREEYKMHDLVHDIAMETSRSYKDLHLNPSNISKKELQKEMINVAGKLRTIDFIQKIPH
NIDQTLFDVEIRNFVCLRVLKISGDKLPKSIGQLKHLRYLEILSYSIELKLPESIVSLHNLQTLKFVYSVIEEFPMNFTN
LVSLRHLELGENADKTPPHLSQLTQLQTLSHFVIGFEEGFKITELGPLKNLKRCLCVLCLEKVESKEEAKGADLAGKENL
MALHLGWSMNRKDNDLEVLEGLQPNINLQSLRITNFAGRHLPNNIFVENLREIHLSHCNSCEKLPMLGQLNNLKELQICS
FEGLQVIDNEFYGNDPNQRRFFPKLEKFEISYMINLEQWKEVITNDESSNVTIFPNLKCLKIWGCPKLLNIPKAFDENNM
QHLESLILSCCNKLTKLPDGLQFCSSIEGLTIDKCSNLSINMRNKPKLWYLIIGCVTQIPEQLQHLTALQFLSIQHFRCI
EALPEWLGNYVCLQTLNLWNCKKLKKLPSTEAMLRLTKLNKLHDLNICLSPSFSSLLCFLIN

>Cucsa.249360
NLKRLLRVAKHLVEIDSQLREIEESVSHIGSEGIGKTTLAKDLYNKIATQFERCCFLQDVRREASKQYGLVQLHETLLCE
ILKEDLKVVNCDKGINIIRSRLCLKKVLIVFDDVDHHRQLEALVGELDWFGRGSKIIMLTRNGHLLSSHGFDEKHKIHEL

DQDHALVLFSLSESATNYCKGLSVALVVLGSFLRGRDQTRWSCILDEFENSLPKDIKDVLQLSFDGLEDKANDIFLDISL
HVIRPLDFGIMILMDLSLIMIESDRVQMHGLIQQMGCSIVRNESSQPEKRSRLWLVQDIGEVFVNKSVRNSLPKLKHVDL
SYSTLLEKILDFSAASNLEELYLTNCTNLGMLDKSILSLNKLTVLNFEGCSNLKMLSRGYFMLSSLKELRPSYYKKLEKI
PDLSAASNLKRLYLQECTNLRVIHKSVGSLDKLGLLDLSQCTNLVKLPSYLRLKSLYTLYLSGCCKLESFPTIAENMKYL
EELYLNFTAI

>Cucsa.251930
MADFIWTFALQEILKKTLHLATQQIRLASGFNHDLSKLLHSLLFFEAILRDVDRTKSDLQSVKIWVTKLQDLVLDAEVVL
DELSYEDLRREVDVNGNSKKRVRDFFSFSNPLMFRLKMARKIRTITQVLNEIKGEASAVGAIPTGGSDEIVADNGHIPET
DSFLDEFEVVGRRADISRIVNVVVDNATHERITVIPIVGMGGLGKTTLAKAVFNHELVIAHFDETIWVCVTATFDEKKIL
RAILESLTNFPSGLDSKDAILRRLQKELEGKRYFLVLDDVWNENVKLWNNFKSLLLKITNSIGNRVLVTTRSEEAGKIME
TFPSHHVEKLSDDECWSIFKERASANGLPLTPELEVIKNVLAEQFGGIPLVAKVLGGAVQFKKRTETWLMSTLETLIMNP
LQNENDVSSILRLSVDHLPNSSLKQCFAYFSNFPKGFNFEKEQLIQFWMAEGFIQPSDKVNPETMEDIGDKYFNILLARS
LFQDIVKDENGKITHCKMHHLLHDLAYSVSKCEALGSNLNGLVDDVPQIRRLSLIGCEQNVTLPPRRSMVKLRSLFLDRD
VFGHKILDFKRLRVLNMSLCEIQNLPTSIGRLKHLRYLDVSNNMIKKLPKSIVKLYKLQTLRLGCFRGEAPKKFIKLISL
RHFYMNVKRPTTRHMPSYLGRLVDLQSLPFFVVGTKKGFHIEELGYLRNLRGKLKLYNLELVRNKEEAMRADLVKKDKVY
KLKLVWSEKRENNNNHDISVLEGLQPHINLQYLTVEAFMGELFPNLTFVENLVQISLKNCSRCRRIPTFGHLPNLKVLEI
SGLHNLKCIGTEFYGNEYGEGSLFPKLKRFHLSDMNNLGRWEEAAVPTEVAVFPCLEELKILDCPRLEIAPDYFSTLRTL
EIDDVNNPISQITLQTFKLLGIIHSGNLSGLPEELRGNLSSLEEFKVWYYLHLKSFPTIQWLTDILKGKTGYDTKWTNIQ
SHGLESYTSVNELSIVGHSDLTSTPDIKALYNLSSLTISGLKKLPKGFHCLTCLKSLSIGGFMEGFDFRPLLHLKSLENL
AMIDFILAESTLPDELQHLTGLKHLKIVGFQGIESLPEWLGNLNSLESLHIESCRKLRELPEAMGCLAKLEEVRSFNCPE
LRVYQDESEWAKISYIPRFISFNYWVDEDQQRIQFKVCHKPS

>Cucsa.252030
MAGSTGANSSSNSSPAPPPPKILLAKPGLVPGGPINSKIGRGAGADDEPTSIRSRLPSLGSLNLLSDSWDLHIDRFLPFL
TENTEFKVVGIIGPPGVGKSTIMNEIYGYDGSSPGMLPPFPILSEDVRAMARHCTLGIEPRISSERIILLDTQPVFSPSV
LAEIMRPDGSSTVSVINGESPSAELAHELMSIQLGILLASICNIVLVISEGVHDLNMWHLMLTVDLLKHGLPDPSSPISS
HAQNSNVASEKEYKEKTSTSEEYMATPIFVHAKVQDRDLVPQNILQLKRAFAYYFKTSSFMGDKFEKVHSEQLLSSVVPD
TRNLDVDGEDRRLLLIPNRNKDDSTRGQYESFNLALWKLRDQVLSMNGASFPRTVSERDWLKNSVKIWESVKSSPIVMEY
ARTLQSSGMFRR

>Cucsa.275630
QSSSSCSSNLKWSYDVFLSFRGEDTRNNFTSHLDRALREKGVNFFIDDKLERGGQISESLLKSIDGSKISIIIFSKNYAS
STWCLDELVKIVQCMKSMGHIVFPVFYKVDPSEVRKQTGGFGEALAKHEANELMTNKVQPWKEALTTAASLSGWDLATRK
NEADLIHDLVKEVLSILNQTQLLHVAKHPVGIDSQLRAVEELASHDVPDGVNMVGIHGMGGIGKTTLAKALYNKIAYQFE
ACCFLSNVRETLEQFKDLVQLQEKLLSEILKDNAWKVGNVHKGKNIIRDRLCSKKVLIILDDVDKDEQLDALVGERDWFG
RGSKIIATTRDRHLLENHSFDIVYPIQLLDPKKSLELFSLHAFKQNHPSSNYVDLSKFAVSYCKGLPLALVILGSLLHKR
ERKIWKSKLHELENSLEPSVEAVFQIGFKELHERVKEIFLDISCFFVGEDINYSKDVLKACDLNPDYGIIILMDLSLVTV
EDGKIQMHDLIQQMGQTIVRHESFEPAKRSRLWEAEGAIKILKEKSVSDFRQCFYLLIAKDIYSEAFRNMKNLRLLILQR
VAYFPRNIFEYLPNSLKWIEWSTFYVNQSSSISFSETPNFFATLNLEKLYLRGCTSLKGMRPSFRKFPSHLKFKSLKVLN
LRDCLNLEEITDFSMASNLEILDLNTCFSLRIIHESIGSLDKLITLQLDLCHNLEKLPSSLKLKSLDSLSFTNCYKLEQL
PEFDENMKSLRVMNLNGTAIRVLPSSIGYLIGLENLNLNDCANLTALPNEIHWLKSLEELHLRGCSKLDMFPPRSSLNFS
QESSYFKLTVLDLKNCNISNSDFLETLSNVCTSLEKLNLSGNKFSCLPSLQNFKSLRFLELRNCKFLQNIIKLPHHLARV
NASGSELLAIRPDCIADMIYLHANDRHHIKVLFPNTTSKFVSKRFKSANVIMRTQGVYMVDRRFSCYT

>Cucsa.277260
MAEFLWTFAVQEVLKKIVNFGAEQISLAWGLEKELSHLKKWLLKAQTILADINTKKSHHHSVGLWVEELHDIIYEADDLL
DEIVYEQIRQTVEQTGKLRKVRDSISPSKNSFLFGLKMAKKMKKITKTLYEHYCEASPLGLVGDESPTESEAALNQIRET
TSILDFEVEGREAEVLEILKLVIDSTDEDHISVISIVGMGGLGKTTLAKMVFNHDAIKGHFDKTVWVCVSKPFIVMKILE
AIFQGLTNTSSGLNSREALLNRLREEMQGKKYFLVLDDVWDKENCLWDELIGNLKYIAGKSGNSIMVTTRSVEVATMVKT
VPIYHLKKLSDDHCWALLKKSANANQLQMNSKLENTKNILVRKIGGVPLIAKVLGGAVKFEEGGSESWMAKIESFARNIS
IEDKDFVLSILKLSVESLPHSALKQCFAYCSNFPQDYEFDKDEAIQMWIAEGFIQPEQERENLTMENIGEEYLNFLLSRS
LFEDAIKYDGRIVTFKIHDLMHDIACAISNHHKMDSNPISWNGKSTRKLRTLICENEEAFHKIQTDIICLRVLVLKWFDT
NTLSTIMAKLIHLRYLDISNCNINKLLRDSICALYNLQTLKLGYIECDLPKNLRNLVNLRHLEFKKFFDMGQMPSHMGNM
IHLQTLSEFVVGLEKGCKIDELGPLKDLKGTLTLKNLQNVQNKDEAMAAKLVEKKYLRHLIFQWFLNLYDRGEYDEDDNK
QVLEGLQPHKNVQSLDIRGFQGRVLNNNIFVENLVEIRLVDCGRCEVLPMLGQLPNLKKLEIISMNSVRSIGSEFYGVDC
NDRNSSAFPQLNKFHICGLKKLQQWDEATVFASNRFGCLKELILSGCHQLAKLPSGLEGCYSIEYLAIDGCPNLMLNVQN
LYNLYHLDIRGLKRLPDEFGKLTNLKKLRIGGCMQNYEFSPFIHLSSQLVELELTDDGSSGSETTQLPQQLQHLTNLKVL
KIADFDDIEVLPEWLGNLTCLATLVFLECKNLKELPSREAIQRLTKLDDLVIDGCPKLLLGEGDQERAKLSHLPSKCGLG
LKSVMINLLPKKSF

>Cucsa.292710
MGSSSVNGAESSSSCSSNSKWSYDVFLSFRGEDTRDKFISHLDLALRREGVNFFIDDKLD

RGKQISKSLLKSIEGSRISIIIFSQNYASSTWCLDEVVKIIECMRSKKQTVLPVFYNVSP
SEVVKQTGIFGEAFAKYETNPLMTNKIQPWKEALTTAATLSGWDLGNYWKNNEAHLIQDL
VKKVSILKQTQLLNVAKHPVAIDSQLKAIEELASHGVSDNGVNMVGIHGMGGIGKTTLAK
ALYNKITYQFEACCFLSNVRETSEQFNGLVQLQEKLLNEIFKDNNLKVDNVDKGMNIIKD
RLCSRKVLMVLDDVDKDDQLDALVGGRDWFGRGSKIIVTTRDRHLLETYSFDKIHPIQLL
DCDKSLELFCWHAFKQSHPSRNYSELPELVRYCNGLPLALVILGSLLCKRDQIIWKSKLD
ELKNFPEPGIEAVFQISFKRLPENPPVKEIFLDICCFFVGEDVSYSKNVLKACDPYLESR
IIILMDLSLVTVEDGKIQMHDLIRQMGQMIVRRKSFKPEKRSRLWVAKEAVKMLIEKSGT
HKVKAIKLDLRNNGSLIVEAEAFRNMENLRLLILQNAAKLPTNIFKYLPNIKWIEYSSSS
VRWYFPISFVVNGGLVGLVINGVSNKHPGIIFEDCKMLKHVDLSYWRLLEETPDFSAALN
LEKLYLLSCKRLKMIHGSVASLSKLVTLDLEGCENLEKLPSSFLMLKSLEVLNLSGCIKL
KEIPDLSASSNLKELHLRECYHLRIIHDSAVGRFLDKLVILDLEGCKILERLPRYISNSK
SIEVMNLDSCRKIEQLFDNYFEKFPSHLKFESLKVLNLSYCQNLKEITDFSIASNLEIFD
LRGCFSLRTIHKSVGSLDQLIALKLDFCHQLEELPSCLRLKSLDSLSLTNCYKIEQLPEF
DENMKSLREMNLKGTAIRKLPTSIRYLIGLENLILSYCTNLISLPSEIHLLKSLKELDLR
ECSRLDMLPSGSSLNFPQRSLCSNLTILDLQNCNISNSDFLENLSNFCTTLKELNLSGNK
FCCLPSLKNFTSLRLLELRNCKFLRNIVKIPHCLKRMDASGCELLVISPDYIADMMFRNQ
DLKLRNFKRELIVTYSEIPKFCNNQTTETYDAYYGKLVWVKIRAYVVTKNSSITVDMLE

>Cucsa.303290
MEMNIWDLEKSLHGSKPSLLRFANWLRAEMEVHGMSCFVSDRAKCRNSRKHRVIERAMDASSFGVVILTKKSFQNPYTIE
ELRFFSGKKNLVPIFFDLSPGDCLARDIVEKRGDLWEKHGGDLWILYGGLEKEWKEAIEGLCRVDEWKFEAQNGNWRDCI
LKAVMLLAMRLGRRSVVEHLTKWREKVEKEEFPFPRNENFIGRKKELSELEFILFGNIAGDSERDYFELKARPRRKNLTL
GWSKSSSLEEKQRELPLEVRNKKGKEPIVWKESEKEIEMQSIEFPQRHRRLKTKSGERYAKRKRTAKILYGKGIACISGD
SGIGKTELLLEFAYRNHQKYKMVLWIGGESRYIRQNYLNLGSFLEVDVGFGNFSGKSKIKNFEEQEEAAISRIRTELMRN
VPFLLIIDNLECEKDWWDHKLVMDLLPRFGGETHIIISTRLPRVMNLEPLKLSYLSGAEAMCLMQGSLRDYSMAEIDVLR
VIEEKVGRLTLGLAIIGAILSELPITPTRLLDTTNRMPFKDQSWSGREAHVFRRNTFLMQLFEVCFSIFDHADGPRSLAT
RMALASGWFGPAAIPISQLTLAAHKIPEKRQRTRLWRKLLRSMACGLTSSYIKKSEAEATSMLLRFNMARSSTKQGCLHF
NDLVKLYARKRGVNGFAQAMVQAVMNRPFIIHHSEHIWAACFLLFGFGRDPVVVELKVSELLYLIKEVVLPLAIRTFLTF
SQCTTALELLRLCTNALEAADQAFVTPVEKWFDKSLCWRPIQTNAQLNPYLWQELALCRATLLETRARLMLRGGQFDIGD
DLIRKAIFIRTSISGEDHPDTISARETLSKLNRLIANFHVHSSP

>Cucsa.318890
LVFNHELVRQHFDKTVWVCVSEPFIVNKILLDILQNLKGTISNGGDSKEVLLRELQKKMHGQRYFLVLDDVWNENSFLWD
ELKYCLLKITGNSKNSIVVTTRSAEVAKIMGTCSGHLLSKLSDDHCWSLFKESANAYGLSMTSNLEIIQKELVKKIGGIP
LAARVLGRAVKFEGDVERWEEMLKNVLSTPLKEENFILSILKLSVDRLPSSALKQCFSYCSIFPKDFVFEKQELIHMWMA
QGFLQPQEGRNMTMETVGDIYFKILLSHCLFEDAHETKTEEYEIPDLLEFETRPEEYKMHDLVHDIAIEISRDQNLQLNP
SNISKKELQKEIKKVACKLRMVDFIRRIPCNIGQLTFFDVEIRNFVCLRVLKLSTLPSDKLPKSIGQLKHLRYLEIACYL
GRLKFPESIVSLHNLQTLKFLYSYVEKFPMNFTNLTLSHFVIGFEEGCKITELGPLKNLQGCLSLLCLEKVESKEEANGT
NLAEKEKLKDLHLSWSNERKDNNNYNDLEVLEGLQPNQNLQSLGIYNFAERRLPNKIFVENLSVIGLYGCNNCEKLPMLG
QLNNLKKLEIYSFHGVQIIDNEFYGNDLNQRRFFPKLEIFVMCDMINLEQWKEVMTNDASSNVTIFSNLKCLEIRGCPKL
TKLPNGLHFCSSIRRHLLSLKKITLVEDELSNNSVTQISEQLQHLTALEFLSIENFGGIEALPEWLGNFVCLQTLSLYNC
KNLKKLPSTKAMLRLTKLNQLYACKCPMLLLEEGDPERAKLSHFPNMLVQRNGYLKCI

>Cucsa.326910
KIPILHQLDYLFHYKQNIKEVEKKVEALGTAKGNAVFDGVSKWLTIVKDVLEIAQQNENPSCFNFVERYQLSRKAKKRVE
NIIELINEGNGFNKDNVGYPVPSPDTNSPTLPTDYQIIASRTSIVEEIKEALANPNVDTVGVCGMGGVGKTALLNEVKKL
VLEKNLFDRVIQVEVGESKSVFNIQEQIKDELNMELNIECEEVRACRLRTHIAERKENMLFMLDDIWKEHDVEKEFGIPF
KHVLNNEMNTEKTFEVNSLTNEESRNFFVTIVGESSCVEDGHNIQQIAEDVVKECGGLPLALKILGKALKGKRVQIWKDA
LKSLKNPVTVTISGVSEQLYSCLQFSYDSTEDEAEQVLLLCSVFPDDYKIEVKDLQMYAMGMGLVKHINTWEDAGNRVIK
LVDDLKSCYLLQDEQSKKGSDDCVQMHDVVHDFAKYVASKKDKMTSLTYRSGQRLEYWQEEDDDMHESYKAIYADCAKYC
VYLPPKVGVSEPSIGNKIRIPTAFFERMKALRVLSVETMSISFEPSSWASINNLEALYELIKLKVLHVLKCDDFNPSEFP
PNIIESMTQLEELKFDGFKMNELSELNRLTRLFSLELRIQNVEILLNELSVEKAEKLEEFSFCVDSVGFTNLLCNNNHTV
PYGNYNWYPRLKELQIYIHNNQNQYLDMPRGIENNPCILIFSCNKLTYVFPSHMLTLLVFLNTLEVHHCKLVERIFEIEE
WSGSGGAGDVNQVLVPFTILHLSFLPNLKHVWNTDPNPTTSDLLPQCKESGDYKVSDA

>Cucsa.328080
MAESILCSLAGSIITKLGSFALQDLGLLWGFHDELDKLKGTVSALEAVLLDAEEKQSKSRAVKDWILKLKDTFYDIDDLL
DVFSYESLKRQVMTKHRTNNTKKVRIFFSKSNQIAFRLKMSQKIKRVREKLDAIAMDKTQFNLYENTREIQDDESTKRLE
TTSFIREGEIIGRDDDKKSIIHYLLDTNIHEDSVAVIAIIGMGGLGKTALVQSIYGDEKVKKHFELTMWVCISEEFDVKV
IIEKIIESLTKKKREPDLQLDTLQSMVREKIDGKRYLLVMDDVWNVNRAKWISLKRYLMGGAKGSRILITTRTHQVAQTF
ETILSHHLKELDEEKSWKLFRKMAFSNESEVLENSKLVVIGKEIVTKLKGSPLAIRVIGSYLYSKKSEKDWLSFKDHELD
TIMQQENEIQSILKISFNHLSSSLKHCFTYCALFSKDYHYEIRKNDLIKQWMAQGFIQPHNKKAMEDVGDDYFEELLGRS

FFQDIRKNKWGEIKKFKMHDIIHDLACSVVENDCVLANDDTKSIDKRTRHVSISAFNSMTRWKLITKSLIEAKNLRTLNY
ARRHHIDLSNHLRLRTLNLEFHFVPKCIGKMKHLRYINITYCYIDFLPKAVTKLYHLETLIIRGCLELRELSSDIKNLIN
LRHLDIKDFKHVWSYMPKGMGSMTTLQTMNLFILGENKGGELSELNGLVNLRGSLSIQQLQFCKPIGLENVKYLEEKSRI
QKLELHWKTYQRESKIDDEDERVLESLKPHSNLQKIRIEGYRGLKLCNWFSFDSIVNLVFIKLFNCEKLQQLPRFDRFPF
LKHLHLEDLPSIEYIAINNYVSSSMTTFFPSLENLSIIKLPNLKEWWKGESIDQNTSFPTILRHLSQLKIHYCRQLASIP
QHGPLQSLDIRDISLQLFELVIKMTATNIIFLPNDLFSNVTHLQSLVIGRCFNLKMSFDDDNVRWKELGSLRTLRLCFIP
KLEYLPKGFQYLKALEHLELLWCENLACILGIEHLTSLSRLEISNCPNLTSLPEGMTQLISLTCLIIDDCPNLSTLPEGL
HHLLNTPRYAPLIFSH

>Cucsa.337180
MACCIYEQAENILIELKKFPMYLRRMQYTMLSLKTILKDAEKEEYRHCLNDWLQKLQSVFLQIEELLYESNREVKKQEAT
GKWVFLPSFNFSQIDQTKKMMKLCDDLDEIASHMYGFNLTNMETTHSFLSATEVSTRLMKPSWQLLYSLTNAPKVFQDKR
YHNFLDHFKKSTHGLFHIVGEPGIGKTTLAKFFYNNLVNTFPSRLWICVKEEFDPQRLIKEMLSFSHCQVTCDNLTEKQL
CFAVQQFLRDKKFLIVFQDISIKNLGNCSIFKSLLGMGNRGSKIIVTTQNEKIADAVGLKKLYKNESQVVPSPEATKPSD
VNKDNMKHQTIFKVERLSKENSLSLFKVHAFTETQEAQIPNLTKIQEVIEQKCHGVPLAIKCLGGLLSKTSIAEWNGVID
KLWEHEEEEDGNKSILPTLRLCYDQMPSHLQRCFLYCSQLKKDRILSSNDVIQLWIASDLLPKENYLSLEKIGENYFKEL
CSRCFLQELEEYGFGYWFKLHPLIEKLARLLTQKQVFEVTKTQSIAFTIRDKVPPSAFLANACIDKFKYLRLLHLGNANL
QGIPSAVENLVQLRYLDLQGNKKIKRLPNSIFKLKNLQTLILASCSALKELPNDIRQLTNLRYLWVTANNLRLHKNGVGT
MTSLRFLAIGGCKKLTLTLKGVEFRLQRFTIRELPIVKKLPEWTQRFTETLRVLEIIDCPIEWNDDVLKSYKSLERFSIH
GAVRTKNQIGGYNIDYRNFVRSRKVKKEVKTCVYY

>Cucsa.337190
MAYCIYYRAENILSELKNLPNYPRRIEYTMLSLKSILMDAEEKQEQSRGLQNWLEELQNVFSQIEGFIDEHKEEAYEGIG
KQVLAPFSCSSNQIARTWKMEKLFDHLNEVAAKMYEFNLTERHTGAIKTETTNSFLTATEVSTRLMKPSWKVLYPLTNAP
KFYQDERYRKILNDFKNPTLGFFHIVGEAGIGKSTLAKFIYNDPEVEGMFPSRLWVCVKEEFDTQRLMKEILNFSYSPAT
CDNLTTKLCPTDQYLRERTFLLVFQDLSIKNLDNCSLFTSLLMMGKPGSKIIVTTQNEEIANAIELTMIYKVGQQSEQNR
SQTALDTVTKETANVNNADQFVQANPLGKIDQSIPSQTIFKVKRLSEKDSLSLFKDYASTYEGNEKDIMKTLKKCNGIPL
AIKCLGSMLSLGPPATKWMEDNERQKGDNESSSTFSILKLCYNEMPSHLKRCFLYCSQLPNDSILSSNDVIQLWMANGLL
RSRQENYLSLEDIGEIYFKELCSRCFLQDVEEYGLGYWFKMHPLIRELARLVQKRTKDLISIKPVTNVTSIAFPVRDEVP
SSSFLAEKCISKFQHLRLLYLGHTDLQEIPNTIETLNHLTYLDLQGNKNIKRLPNAICNLQHLQTLILASCSALEELPKD
ICKLSNLRYLWVTSNKLRLHKNGVGTMTSLRFLAIGGCDKLQDLFERPSCLVRLETLMIYDCNSLQLLPNEMGSLISLQN
LVIWSCKQLTLKGLEKVDFSLQRFTIRELPEVNKLPEWLQRSTETLRVLEIIDCPIKVEEEGIKIHHWEFGTA

>Cucsa.337200
MAHCIYTQAENILTKLKDSPPYQKRIEYAMSSLKAVLLDAEEKQEQNQRLQNWLKELQNVFYQVEDSIDEFKWEIFKQKD
IGKQVLAPFSCSNQISANKLKQKCKRKQVCDELNNIATRMYEFHLKVKHIDSISMETTHTFPSASEISIRHLKPSWQLLY
PLIDASRTYDEIYDGILNVFNECTHVFHIVGEAGIGKSTVARFLYNHNNVVGKYTSRYWVCVEEGFNKHRLVKEVYSHAD
NKEICEDLTTEQLLSKVKRLLKVETFLLVFQDLSITNLKDRSPLVLNELLEMGQHGSKIIVTTQTEEIANYLQDRGYKTE
RRSKENLANGDRVSAHNQSLTKNTQNQAVPGSQIEETADAIANKIYEHSLSLTEDAQNLEVPIPQVTEDTNIGLGINPDI
PPIKQDDTEYQTIFKLEKLSKQSSSRLFKEYAFRNRQEVENPELTKIVDQLLEKCMGVPLAIKCLGSLLSSETSIAKWKN
IEEKLSLQEKKENGILHVLRVCYDQMPSQLKPCFLHCSQLPNDRIISSNDMIQLWMANGLLHSPEEKNSTMENIGEKYFM
ELWSRYFIQEIEEHGLGYWIKLHPLIQKLAHKITQEQSEGSGGNNHPKEVTEIRSIAFQERNMVLPNASLTEKCIWKYKG
LRLLYLSNADLQEIPNSIGTLKYLRYLDLHGNTKIKHLPNSICNLQSLQTLILGSCSALEDLPKDIRNLISLRYLWVTTN
KLRLDKNGVGTMNSLRFLAIGGCNNLENLFERPDCLAGLETLMIYNCTTLKLLPDEMRYLKSLQNLMIWSCKQLTLNLKE
VEFKLQRFTIKELPRVERLPQWLENSAETLRTLQIINCPIRIMERQGIEKYEAVENTIIYGAVRFEMAPPGYDFEHRNLA
VRNGNEEMHIYP

>Cucsa.338100
MAEIGTFVVQEVLKRIVKYGAEQIVVAWELENEVSLLKDKLHDADTILEDINRKKSHPGN
SVKRWVEKLEDIVHEADDLLDELVYEHLRRTVEHTEKFSKVSDSISSSINSFLFRRKMAK
KIKNITDTLNQHYCAASAFGLVGVETVTEIELALNQIRETTSILDFQVEGREAEVLELLK
LAIDSTNEHHMSVISIVGMGGLGKTTLAKMIFNHREIEGHFDKTIWVCVSKPFIVTKILE
KIFQGLTKTCSGLESNKEALLGRLRKEMQDKNYFLVLDDVWDNEKHLWDELRGCLKHIAG
KPGNTIVMTTRNEEVATMVEPISIYRLKKLSNDQCWALFKESANANQLPMNSKLEIMKKE
LVKKMGGVPLVAKVLGGAVKFEETELEEEDHEISWMTKVESIVRNISLEDKDFVLSILKL
SVDSLPNPVLKQCVAYCSNFSQDYDFQKDDLIKMWIAQGFIQPGQGRDKNLLMEDIGEQY
FNFLLSRSIFQDVTRDANKRIVGFKMHDLMHDIACAISSHQNVESNPNNLSGKSVRKLRT
LICNDEVINYLNQKDIVCLRVLKVIFQSHTDLWIPIDKLIHLRYLDISECSINKLLLESL
SLLYNLQTLKLGQSGLPKNLRKLVNLRHLEFKMFGDTAMPSDMGNLIHLQSLSGFLVGFE
KGCKIEELGPLKNLKGKLTLTNLWRVQNKDEAMAAKLVEKKNLRHLNLWFFETDKRGEDD
EDGIVQVLEGLQPHKNLQSLEILGFRGKVLPTGIFVENLVKIRLGHFERCEVLPMLGQLP
NLKELEIMYMESVRSIGNEFYGVDSSHQNSVAFPQLKKVSIYEMMNLEQWDEATVVLASN
LFGCLKEVRIRRCNPLAKLPSGLEGCHSLEYLSIRGCFNLMLNVQNLHKLYHLEIDGLKR

LPKGMDGLTRLKELKIGGCMQNYEFSSVIHLASQLVELELSGRYGSVDTQLPQQLQHLTN
LQVLKITQFDCIEALPEWIGNLISLKTLKCSYCFKLKELPSREAILRLTKLENLDIFECP
KLLVGEGDQERAKLSHLPSKCVHKSE

>Cucsa.338190
MVGLLDSVAGNLLGRIIEAADRLEFRAIQSELKNLETDVLNLKARLRDAEEKQASNCELNELLKNLKNVFSRADIAIEEL
ECDYLKWRVQNRKNDVDDKGCQFSSCFSSNFLISPFNTGSKFQEDLKIITSELRSIEKAMSKFSLVEDEDEYIKKLKGEM
TLRTSITGSHAFARLLRLRREAILSNVDSIFGRDKIQESIIKELVNDEQKSPRILSIQGDGGMGKTALAKLVYNADEVFD
HFDKRMWVCVSEDFDIRRILREVLMSATGENVTTVALTESRLRIRLQRYFFGKKILLVLDDFGNLDPERVSELKKIVKMG
VGGSKIMITTRSDETLNVATTHKIDKLDETISMQIFEDTYGSEGLSEGLRDDLYLKNLVAECGGAPLAIKCLAGLLSSKP
SDGAKSPNVKDLSEKWKQEEANNGGGVLCALRLSYDLMPSYLKPCFLCFSVLPKDNVFFSFELIQLWMAQGILPSGTKDN
PEEVGEKYFKEFRDRRLLVDVEEHTLGYWFKIHSLVHDLAVQKATEQKNLGNFHMLSFVDCDSIPSSTNYDNTRFISIPV
VGGAGPNINSDLFKCITQFRQLRFLYLCNSSLEEIPTSIDTLKHLRCLDLRGSQRLKRLPESICKLQSLQTLVLAFCSEL
EELPRNIKNLISLRFLWIQTKQARLEKDEIGSLTSLRFLAIGRSENLTHLFEDINKLNSLKTLIIYECKSLLTLPKGLEN
MKSICNMGIWECDRLRFTFSLASLHLKKLILRELTAVSTLPNWLSNLDGTLEVLEIGEFPTLRKLPIWLLNFWELRILGI
SNCPKLKHDSFPPELNYFCDKIEELRITFCGSLSKSLLKKSMKEIEPESRVIFYIHTIYVDSKRMTPPVESTDEPKEAET
KQDDAYNNASPPGTEQPSKTKHDDANNNMSHPGIGLLSESKQEHTNNNINEIETVKVCLGDNDHAEAHQAM

>Cucsa.338650
MADELRPQHGNWTYDVFLSFRGEDTRKNFTDHLYYAFKDAGINVFRDDPELERGEDISSELERAIEGSKVAVVVFSERYA
ESGWCLEELVKIMECRRTLRQLVFPIFYNVDPSCVRKQKGEFEEAFVKHEVRYFRDIDRVLKWRMALTEAANLSGWDLRN
IANGHEAKFIRLIVEKVSKEVNSKYLFIALYPVGIESRLKLLLSHLHIGSNDVRFVGILGMGGLGKTTVAKALYNQLYHN
FEAKCFLSNIKAETSNLIHLQKQLLSSITNSTNINLGNIDQGIAVLQERLRCKRLLLILDDVDDLSQLTALATSRDLFAS
GSRIIITTRDRHLLNQLEVDEICSIDEMDDDEALELFSWHAFRNSYPSETFHQLSKQVVTYCGGLPLALEVLGSFLFGRS
REEWEDTLKKLKKIPNDQIQKKLKISFDGLNDHTYKDIFLDVSCFFIGMERNYVEQILDGCGFFPRIGISVLLQRCLLTI
GDKNRLMMHDLLRDMGREIVRENFPKYPERHSRLFLHEEVLSVLTRQKGTDATEGLSLKLPRFSKQKLSTKAFNEMQKLR
LLQLNFVDVNGDFKHISEEIRWVCWHGFPLKFLPKEFHMDKLVAMDLRYSQIRFFWKESKFLKNLKFLNLGHSHYLTHTP
NFSKLPNLEILSLKDCKNLIELHPTIGELKALISLNLKDCKSLNSLPNSFSNLKSLQTLIISGCSKLNSLPEDLGEITSL
ITLIADNTPIQKIPNTIINLKNLKYLSLCGCKGSPSKSSFSSMIWSWISPKKLSQNYTSILLPSSLQGLNSLRKLCLKNC
NLSNNTIPKDIGSLSSLRELDLSENLFHSLPSTISGLLKLETLLLDNCPELQLIPNLPPHLSSLYASNCTSLERTSDLSN
VKKMGSLSMSNCPKLMEIPGLDKLLDSIRVIHMEGCSNMSNSFKDTILQGWTVSGFGGVCLPGKEVPDWFAYKDEGHSIF
LELPQYNNSNLEGFIVCIVYCSCFNNTVSTDLPSLSVINYTKSSITTNKPLTNDVIMSTQDHLWQGHLSNKAFKMEPGDE
VEIIVDFGAEITVKKIGISLVFDKYVDQTMLEFASTCNDDDVVVDNQDETVSEKDGEVGSKRGFDENDDEGLKNSYQIPK
RLKCEIDSNMKIDEE

>Cucsa.338660
MANEFQAQHGDWTYDVFLSFRGEDTRKNFTDHLYYALKDAGINVFRDDPELQRGEDISSGLERAIEGSKVAVIVFSERYA
ESGWCLEELVKIMECRRTLRQMVLPVFYNVDPSCVRKQKGEFEEAFVKHEKGKDIDKVRRWRMALTEAANVAGLGLTQNA
NGHEAEFIRSIVKMISKEVKSNYLFIALYPVGIESRIKLVLPHLHIGSNDDVKFVGILGIGGLGKTTIAKALYNQLHHNF
EAACFLANIKQTPNQPNGLVHLQKQLLSSITNSSNINFENMDRGIVVLQESLRRKKLLLILDDVDKISQLTALATRRECF
GSGSRIVITTRHRRLLNQIEVDGICSIDVMDDAEALQLFSWHAFHNSYPSETFHQLSKRVVNYCGGLPLALQVLGCFLFG
RSREEWQDTLKNLKKILDDQIQIKPKITFDTHNDHTCKDIYLVNQMLDGWGSFPRIGDINRLVTSDLLRDHTQLFLPKEV
RLSVLGPKVRK

>Cucsa.368510
MLAFTVESVGSLIVGSSFVLKLDTALELLSWYALKDKSPSSSYLELAEVAVGYCQGLPLALVLLDELVISLEKGVQDIFQ
ISYEGLEYRLKELFVDICCLFVGDDVYCMKSMLKARDLNPDYGITVLNDLSLISIENGKVQMHDLIPQMGHTIVRGESFD
LENRSRLWVAKEGTDAVKVIKLDLSNDLSPTSIDAQAFKNMKNLKLLILRNITFSSHMFEYGLNKFKWVLSSFGIRFSSL
PASFLVKEGLVGLDMQHCCIKYLGNRFENCERLKHVDLSYCELLEEIPDLSVAVNLEYLYLRGCISLKTIHESVSSLNKL
ITLDLEGCVNLEKLPSYLMLKSLDSFCLSDCRKLQRVPEFDENMKSLTRMILDYTAIEELPSSIEHLAKLEFLSLKGCAN
LVALPSEIYLLRSLVELRLPGCSKLHMFPCPTTEQIFLLSKLTILDLKNCNLSNIDFLEPLSTDRLNDSGSREFILMNGM
IPEWFNHQTTTTSSISVSLQHCPQKTQVLAACVIFKVDGDSCEAKASIKYDIFIDGESLKYFEITVRPSSKSEYMWLITT
PLTFSSVSMSCQVSCTINKTYDNARATIRSLGVHIDVRGQQRQTX
