# Supplementary material for: A 1,681-locus consensus genetic map of cultivated cucumber including 67 NB-LRR resistance gene homolog and ten gene loci
Source: BMC Plant Biol. 2013 Mar 25;13:53. doi: 10.1186/1471-2229-13-53 (PMC3626583; doi:10.1186/1471-2229-13-53)
Supplement: Additional file 3 — Supplemental data file including three supplemental figures (Figure S1 to Figure S3). Figure S1. Distribution of 70 NB domain-containing RGHs across seven cucumber chromosomes in the Gy14 genome. Figure S2. Graphic view of colinearity of common markers among three cucumber linkages used for consensus map construction. MapA = Gy14 × PI 183967 RIL (CSH-RIL) [42]; MapB = Gy14 × 9930 F2 (CSS-F2 map) (Additional file 2: Table S4, this study); MapC = 9110Gt × 9930 RIL (CSS-RIL) [45]. The graphs were drawn with the Circos software package (http://circos.ca/) [70]. Figure S3. Graphic view of consensus bin map of cucumber, which was developed from integration of three individual maps of Gy14 × 9930 F2 (CSS-F2) (Additional file 2: Table S4, this study), Gy14 × PI 183967 RIL (CSH-RIL) [42] and 9110Gt × 9930 RIL (CSS-RIL) [45] with JoinMap 3.0. Numbers on top of the map are linkage groups (LG) (1 through 7) which also correspond to chromosomes numbers. Cumulative map distance (cM) is shown to the left of each linkage group and marker designation is on the right. [file 1471-2229-13-53-S3.pdf]

Figure S1

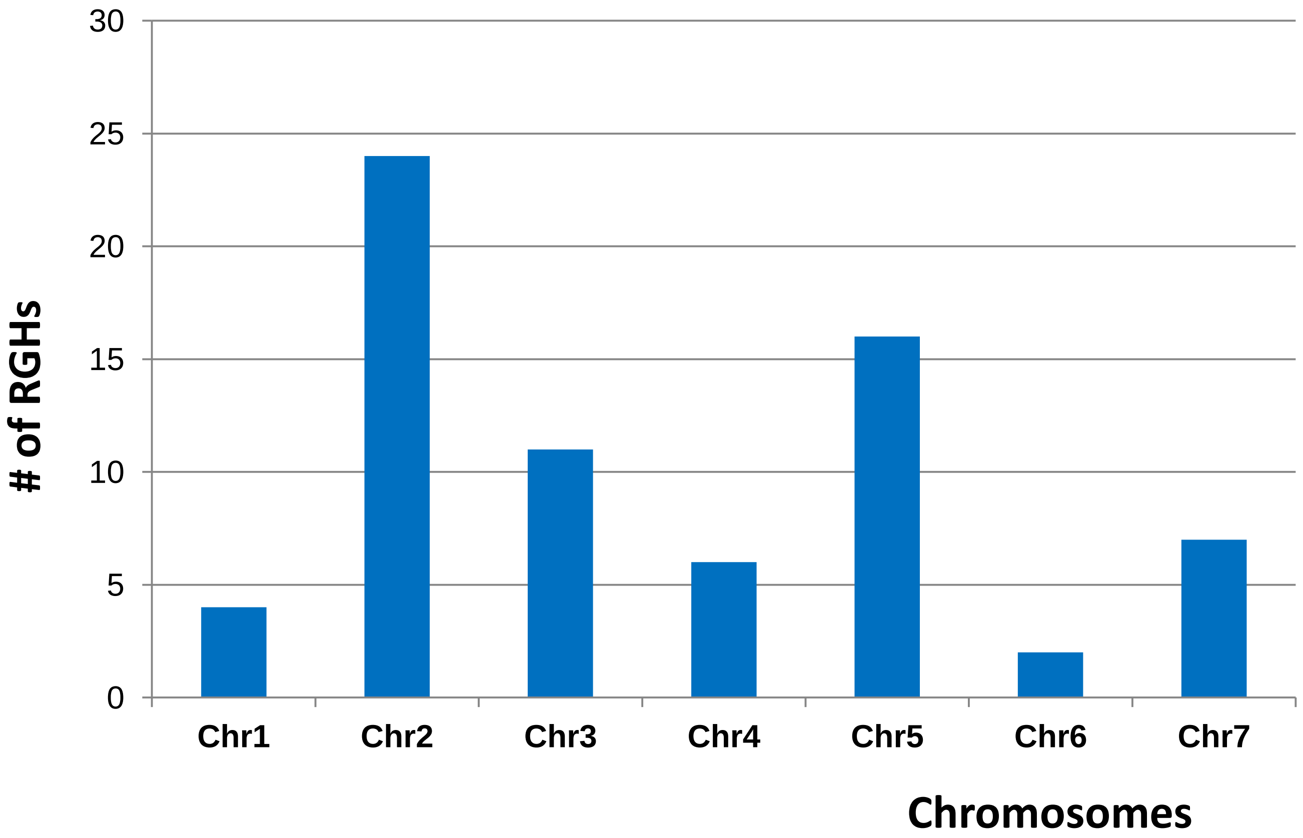

# of RGHs
30
25
20
15
10
5
0
Chr1
Chr2
Chr3
Chr4
Chr5
Chr6
Chr7
Chromosomes

Figure S2

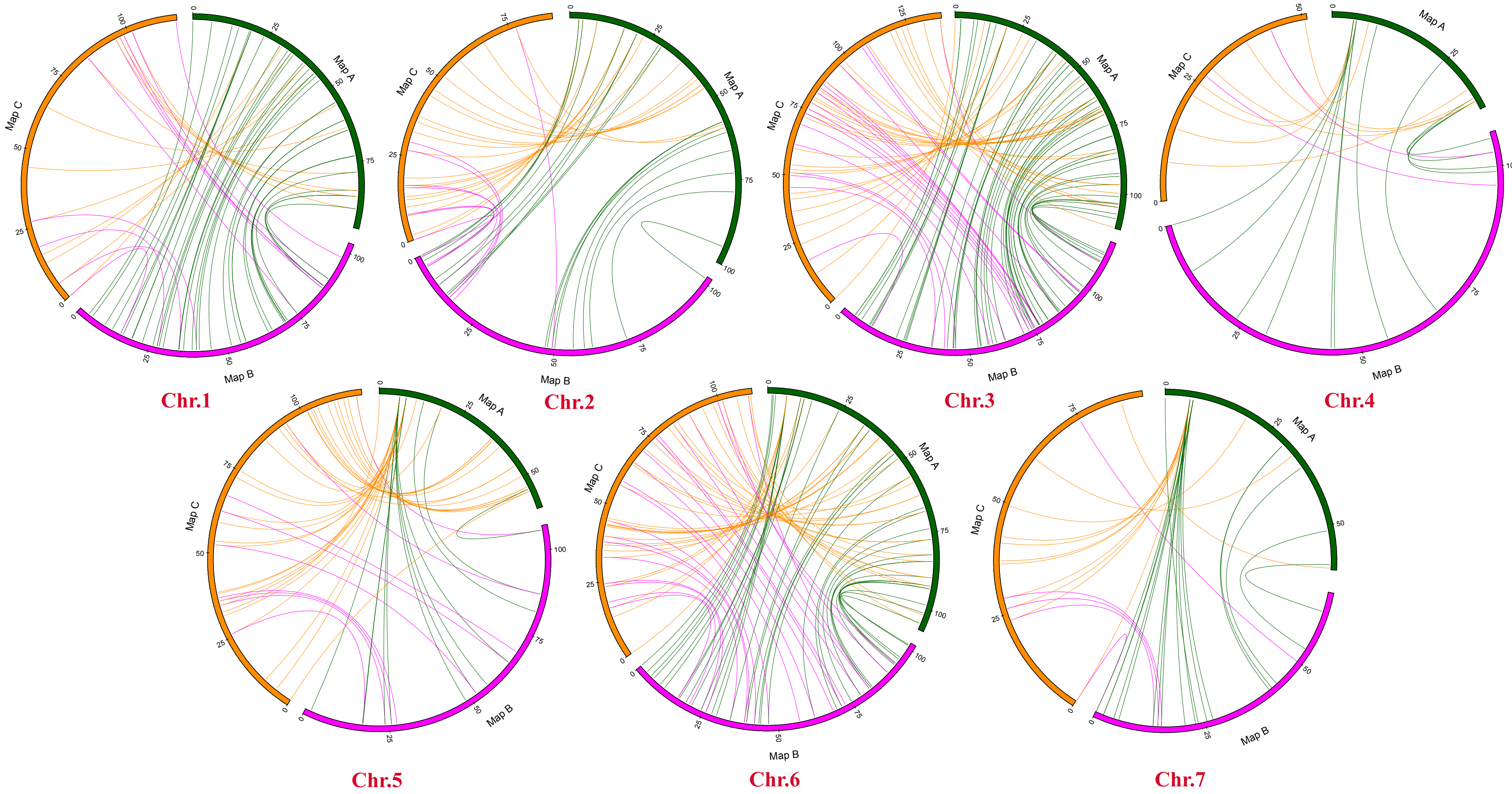

Figure S3

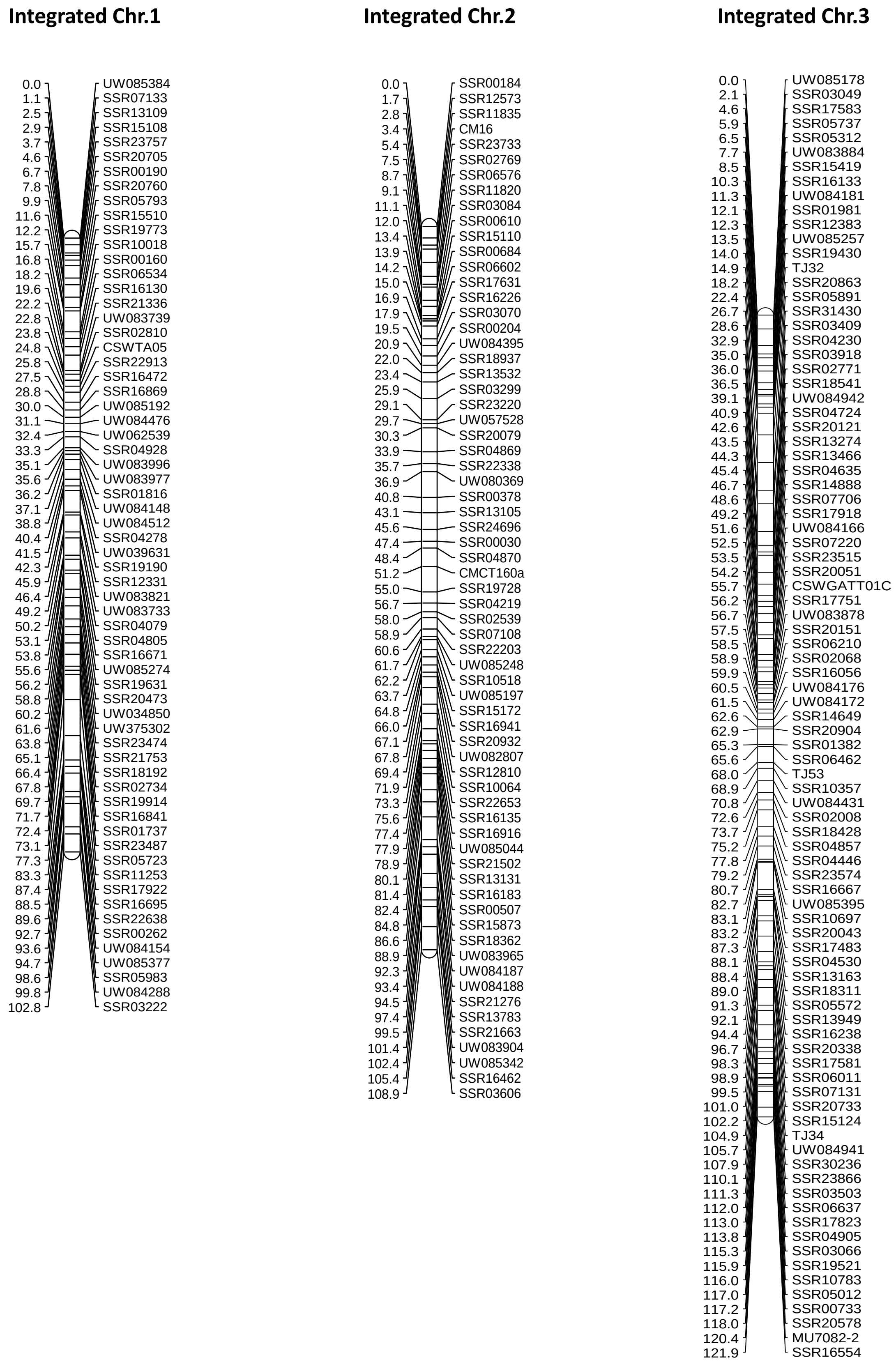
Integrated Chr.1
0.0 UW085384
1.1 SSR07133
2.5 SSR13109
2.9 SSR15108
3.7 SSR23757
4.6 SSR20705
6.7 SSR00190
7.8 SSR20760
9.9 SSR05793
11.6 SSR15510
12.2 SSR19773
15.7 SSR10018
16.8 SSR00160
18.2 SSR06534
19.6 SSR16130
22.2 SSR21336
22.8 UW083739
23.8 SSR02810
24.8 CSWTA05
25.8 SSR22913
27.5 SSR16472
28.8 SSR16869
30.0 UW085192
31.1 UW084476
32.4 UW062539
33.3 SSR04928
35.1 UW083996
35.6 UW083977
36.2 SSR01816
37.1 UW084148
38.8 UW084512
40.4 SSR04278
41.5 UW039631
42.3 SSR19190
45.9 SSR12331
46.4 UW083821
49.2 UW083733
50.2 SSR04079
53.1 SSR04805
53.8 SSR16671
55.6 UW085274
56.2 SSR19631
58.8 SSR20473
60.2 UW034850
61.6 UW375302
63.8 SSR23474
65.1 SSR21753
66.4 SSR18192
67.8 SSR02734
69.7 SSR19914
71.7 SSR16841
72.4 SSR01737
73.1 SSR23487
77.3 SSR05723
83.3 SSR11253
87.4 SSR17922
88.5 SSR16695
89.6 SSR22638
92.7 SSR00262
93.6 UW084154
94.7 UW085377
98.6 SSR05983
99.8 UW084288
102.8 SSR03222
Integrated Chr.2
0.0 SSR00184
1.7 SSR12573
2.8 SSR11835
3.4 CM16
5.4 SSR23733
7.5 SSR02769
8.7 SSR06576
9.1 SSR11820
11.1 SSR03084
12.0 SSR00610
13.4 SSR15110
13.9 SSR00684
14.2 SSR06602
15.0 SSR17631
16.9 SSR16226
17.9 SSR03070
19.5 SSR00204
20.9 UW084395
22.0 SSR18937
23.4 SSR13532
25.9 SSR03299
29.1 SSR23220
29.7 UW057528
30.3 SSR20079
33.9 SSR04869
35.7 SSR22338
36.9 UW080369
40.8 SSR00378
43.1 SSR13105
45.6 SSR24696
47.4 SSR00030
48.4 SSR04870
51.2 CMCT160a
55.0 SSR19728
56.7 SSR04219
58.0 SSR02539
58.9 SSR07108
60.6 SSR22203
61.7 UW085248
62.2 SSR10518
63.7 UW085197
64.8 SSR15172
66.0 SSR16941
67.1 SSR20932
67.8 UW082807
69.4 SSR12810
71.9 SSR10064
73.3 SSR22653
75.6 SSR16135
77.4 SSR16916
77.9 UW085044
78.9 SSR21502
80.1 SSR13131
81.4 SSR16183
82.4 SSR00507
84.8 SSR15873
86.6 SSR18362
88.9 UW083965
92.3 UW084187
93.4 UW084188
94.5 SSR21276
97.4 SSR13783
99.5 SSR21663
101.4 UW083904
102.4 UW085342
105.4 SSR16462
108.9 SSR03606
Integrated Chr.3
0.0 UW085178
2.1 SSR03049
4.6 SSR17583
5.9 SSR05737
6.5 SSR05312
7.7 UW083884
8.5 SSR15419
10.3 SSR16133
11.3 UW084181
12.1 SSR01981
12.3 SSR12383
13.5 UW085257
14.0 SSR19430
14.9 TJ32
18.2 SSR20863
22.4 SSR05891
26.7 SSR31430
28.6 SSR03409
32.9 SSR04230
35.0 SSR03918
36.0 SSR02771
36.5 SSR18541
39.1 UW084942
40.9 SSR04724
42.6 SSR20121
43.5 SSR13274
44.3 SSR13466
45.4 SSR04635
46.7 SSR14888
48.6 SSR07706
49.2 SSR17918
51.6 UW084166
52.5 SSR07220
53.5 SSR23515
54.2 SSR20051
55.7 CSWGATT01C
56.2 SSR17751
56.7 UW083878
57.5 SSR20151
58.5 SSR06210
58.9 SSR02068
59.9 SSR16056
60.5 UW084176
61.5 UW084172
62.6 SSR14649
62.9 SSR20904
65.3 SSR01382
65.6 SSR06462
68.0 TJ53
68.9 SSR10357
70.8 UW084431
72.6 SSR02008
73.7 SSR18428
75.2 SSR04857
77.8 SSR04446
79.2 SSR23574
80.7 SSR16667
82.7 UW085395
83.1 SSR10697
83.2 SSR20043
87.3 SSR17483
88.1 SSR04530
88.4 SSR13163
89.0 SSR18311
91.3 SSR05572
92.1 SSR13949
94.4 SSR16238
96.7 SSR20338
98.3 SSR17581
98.9 SSR06011
99.5 SSR07131
101.0 SSR20733
102.2 SSR15124
104.9 TJ34
105.7 UW084941
107.9 SSR30236
110.1 SSR23866
111.3 SSR03503
112.0 SSR06637
113.0 SSR17823
113.8 SSR04905
115.3 SSR03066
115.9 SSR19521
116.0 SSR10783
117.0 SSR05012
117.2 SSR00733
118.0 SSR20578
120.4 MU7082-2
121.9 SSR16554

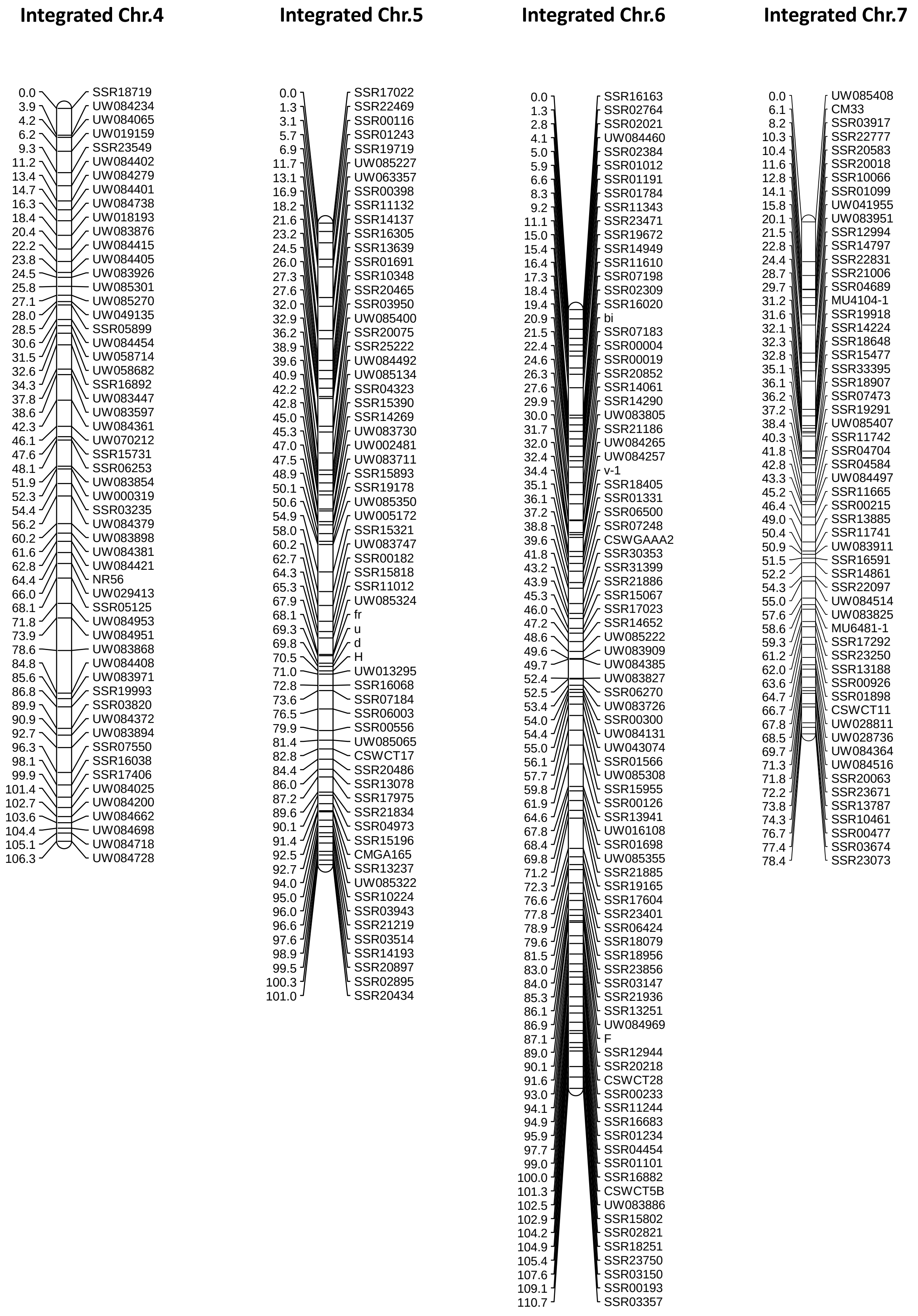
Integrated Chr.4
0.0 SSR18719
3.9 UW084234
4.2 UW084065
6.2 UW019159
9.3 SSR23549
11.2 UW084402
13.4 UW084279
14.7 UW084401
16.3 UW084738
18.4 UW018193
20.4 UW083876
22.2 UW084415
23.8 UW084405
24.5 UW083926
25.8 UW085301
27.1 UW085270
28.0 UW049135
28.5 SSR05899
30.6 UW084454
31.5 UW058714
32.6 UW058682
34.3 SSR16892
37.8 UW083447
38.6 UW083597
42.3 UW084361
46.1 UW070212
47.6 SSR15731
48.1 SSR06253
51.9 UW083854
52.3 UW000319
54.4 SSR03235
56.2 UW084379
60.2 UW083898
61.6 UW084381
62.8 UW084421
64.4 NR56
66.0 UW029413
68.1 SSR05125
71.8 UW084953
73.9 UW084951
78.6 UW083868
84.8 UW084408
85.6 UW083971
86.8 SSR19993
89.9 SSR03820
90.9 UW084372
92.7 UW083894
96.3 SSR07550
98.1 SSR16038
99.9 SSR17406
101.4 UW084025
102.7 UW084200
103.6 UW084662
104.4 UW084698
105.1 UW084718
106.3 UW084728
Integrated Chr.5
0.0 SSR17022
1.3 SSR22469
3.1 SSR00116
5.7 SSR01243
6.9 SSR19719
11.7 UW085227
13.1 UW063357
16.9 SSR00398
18.2 SSR11132
21.6 SSR14137
23.2 SSR16305
24.5 SSR13639
26.0 SSR01691
27.3 SSR10348
27.6 SSR20465
32.0 SSR03950
32.9 UW085400
36.2 SSR20075
38.9 SSR25222
39.6 UW084492
40.9 UW085134
42.2 SSR04323
42.8 SSR15390
45.0 SSR14269
45.3 UW083730
47.0 UW002481
47.5 UW083711
48.9 SSR15893
50.1 SSR19178
50.6 UW085350
54.9 UW005172
58.0 SSR15321
60.2 UW083747
62.7 SSR00182
64.3 SSR15818
65.3 SSR11012
67.9 UW085324
68.1 fr
69.3 u
69.8 d
70.5 H
71.0 UW013295
72.8 SSR16068
73.6 SSR07184
76.5 SSR06003
79.9 SSR00556
81.4 UW085065
82.8 CSWCT17
84.4 SSR20486
86.0 SSR13078
87.2 SSR17975
89.6 SSR21834
90.1 SSR04973
91.4 SSR15196
92.5 CMGA165
92.7 SSR13237
94.0 UW085322
95.0 SSR10224
96.0 SSR03943
96.6 SSR21219
97.6 SSR03514
98.9 SSR14193
99.5 SSR20897
100.3 SSR02895
101.0 SSR20434
Integrated Chr.6
0.0 SSR16163
1.3 SSR02764
2.8 SSR02021
4.1 UW084460
5.0 SSR02384
5.9 SSR01012
6.6 SSR01191
8.3 SSR01784
9.2 SSR11343
11.1 SSR23471
15.0 SSR19672
15.4 SSR14949
16.4 SSR11610
17.3 SSR07198
18.4 SSR02309
19.4 SSR16020
20.9 bi
21.5 SSR07183
22.4 SSR00004
24.6 SSR00019
26.3 SSR20852
27.6 SSR14061
29.9 SSR14290
30.0 UW083805
31.7 SSR21186
32.0 UW084265
32.4 UW084257
34.4 v-1
35.1 SSR18405
36.1 SSR01331
37.2 SSR06500
38.8 SSR07248
39.6 CSWGAAA2
41.8 SSR30353
43.2 SSR31399
43.9 SSR21886
45.3 SSR15067
46.0 SSR17023
47.2 SSR14652
48.6 UW085222
49.6 UW083909
49.7 UW084385
52.4 UW083827
52.5 SSR06270
53.4 UW083726
54.0 SSR00300
54.4 UW084131
55.0 UW043074
56.1 SSR01566
57.7 UW085308
59.8 SSR15955
61.9 SSR00126
64.6 SSR13941
67.8 UW016108
68.4 SSR01698
69.8 UW085355
71.2 SSR21885
72.3 SSR19165
76.6 SSR17604
77.8 SSR23401
78.9 SSR06424
79.6 SSR18079
81.5 SSR18956
83.0 SSR23856
84.0 SSR03147
85.3 SSR21936
86.1 SSR13251
86.9 UW084969
87.1 F
89.0 SSR12944
90.1 SSR20218
91.6 CSWCT28
93.0 SSR00233
94.1 SSR11244
94.9 SSR16683
95.9 SSR01234
97.7 SSR04454
99.0 SSR01101
100.0 SSR16882
101.3 CSWCT5B
102.5 UW083886
102.9 SSR15802
104.2 SSR02821
104.9 SSR18251
105.4 SSR23750
107.6 SSR03150
109.1 SSR00193
110.7 SSR03357
Integrated Chr.7
0.0 UW085408
6.1 CM33
8.2 SSR03917
10.3 SSR22777
10.4 SSR20583
11.6 SSR20018
12.8 SSR10066
14.1 SSR01099
15.8 UW041955
20.1 UW083951
21.5 SSR12994
22.8 SSR14797
24.4 SSR22831
28.7 SSR21006
29.7 SSR04689
31.2 MU4104-1
31.6 SSR19918
32.1 SSR14224
32.3 SSR18648
32.8 SSR15477
35.1 SSR33395
36.1 SSR18907
36.2 SSR07473
37.2 SSR19291
38.4 UW085407
40.3 SSR11742
41.8 SSR04704
42.8 SSR04584
43.3 UW084497
45.2 SSR11665
46.4 SSR00215
49.0 SSR13885
50.4 SSR11741
50.9 UW083911
51.5 SSR16591
52.2 SSR14861
54.3 SSR22097
55.0 UW084514
57.6 UW083825
58.6 MU6481-1
59.3 SSR17292
61.2 SSR23250
62.0 SSR13188
63.6 SSR00926
64.7 SSR01898
66.7 CSWCT11
67.8 UW028811
68.5 UW028736
69.7 UW084364
71.3 UW084516
71.8 SSR20063
72.2 SSR23671
73.8 SSR13787
74.3 SSR10461
76.7 SSR00477
77.4 SSR03674
78.4 SSR23073
